# Supplementary material for: In-silico-assisted derivatization of triarylboranes for the catalytic reductive functionalization of aniline-derived amino acids and peptides with H2
Source: Nat Commun. 2024 May 7;15:3708. doi: 10.1038/s41467-024-47984-0 (PMC11076482; doi:10.1038/s41467-024-47984-0)
Supplement: Supplementary file 1 — Supplementary Information [file 41467_2024_47984_MOESM1_ESM.pdf]

Supplementary Information

In-Silico-Assisted Derivatization of Triarylboranes for the Catalytic Reductive Functionalization of Aniline-Derived Amino Acids and Peptides with H_2

Yusei Hisata,[1] Takashi Washio,[2] Shinobu Takizawa,[3] Sensuke Ogoshi,[1] Yoichi Hoshimoto*[1,4]

[1]Department of Applied Chemistry, Faculty of Engineering, Osaka University Suita, Osaka, 565-0871, Japan
[2]Department of Reasoning for Intelligence and Artificial Intelligence Research Center, SANKEN, Osaka University, Ibaraki, Osaka 567-0047, Japan
[3]Department of Synthetic Organic Chemistry and Artificial Intelligence Research Center, SANKEN, Osaka University, Ibaraki, Osaka 567-0047, Japan
[4]Center for Future Innovation (CFi), Division of Applied Chemistry, Faculty of Engineering, Osaka University, Suita, Osaka 565-0871, Japan

E-mail to the corresponding author: hoshimoto@chem.eng.osaka-u.ac.jp (to YH)

Table of Contents

Supplementary Methods

[1] General considerations p. S3

[2] Materials p. S3

Supplementary Notes

[3] Preparation of reagents pp. S3−19

Supplementary Discussion

[4] Collection of experimental data p. S20

[5] Comparison of Lewis acidity among B^{xy} by Gutmann-Beckett method p. S20

[6] Effect of solvents p. S21

[7] Robustness screening with FGE kits pp. S22−S23

[8] Reductive alkylation of 1 with 2 for preparation of 3 pp. S23−28

[9] Monitoring the formation of 3aa p. S29

[10] Gaussian process regression for Model I to V p. S30

[11] Theoretical studies pp. S31−S38

[12] NMR spectra pp. S39−S123

Supplementary References pp. S124−S125

Supplementary Methods

[1] General considerations

Unless otherwise noted, all manipulations were conducted under a N_2 atmosphere using standard Schlenk line or grove box (GB) techniques. ^{1}H, ^{11}B, ^{13}C, ^{19}F, and ^{31}P NMR spectra were recorded on a Bruker AVANCE III 400 or at 25 °C. The chemical shifts in the ^{1}H NMR spectra were recorded relative to Me_4Si or residual protonated solvent ($CHCl_3$ (*δ* 7.26), $CDCl_2H$ (*δ* 5.32), C_6D_5H (*δ* 7.16)). The chemical shifts in the ^{11}B NMR spectra were recorded relative to BF_3. The chemical shifts in the ^{13}C spectra were recorded relative to Me_4Si or deuterated solvent ($CDCl_3$ (*δ* 77.16), CD_2Cl_2 (*δ* 53.84), C_6D_6 (*δ* 128.06)). The chemical shifts in the ^{19}F NMR spectra were recorded relative to α,α,α-trifluorotoluene (*δ* −65.64). The chemical shifts in the ^{31}P NMR spectra were recorded relative to 85% H_3PO_4 as an external standard. Assignment of the resonances in ^{1}H and ^{13}C NMR spectra was based on ^{1}H−^{1}H COSY, HMQC, and HMBC experiments. High resolution mass spectrometry (HRMS) and elementary analyses were performed at the Instrumental Analysis Center, Faculty of Engineering, Osaka University. Exact mass spectra were recorded on a double-focusing mass spectrometer (JMS-700; JEOL). X-ray crystal data were collected with Rigaku XtaLAB Synergy equipping with the HyPix-6000HE detector.

[2] Materials

All commercially available reagents including super-dehydrated solvents (*n*-hexane, toluene, tetrahydrofuran, and Et_2O), were purchased from Sigma Aldrich, TCI, and Wako Pure Chemical Industries, and used as received. Benzene-*d*$_6$ was distilled from sodium benzophenone ketyl prior to use. CD_2Cl_2 was once degassed by several freeze-pump-thaw cycles and stored inside GB over molecular sieves (4 Å). 4-Methyltetrahydropyran (MTHP) was provided from Kuraray Co., Ltd., and used after distillation using sodium benzophenone ketyl. Compounds **A0**−**A21** (functional group evaluation kit) shown in Figure 6 was provided by Oshima and Morimoto et al. (Graduate School of Pharmaceutical Sciences, Kyushu University), and used after dried in vacuo overnight.[1] Metrical data for the solid-state structures are available from Cambridge Crystallographic Data Centre: CCDC 2295627 (**B**^{1f}), 2295628 (**B**^{2b}), 2295633 (**B**^{2c}), 2295634 (**B**^{2e}), 2295629 (**B**^{3b}), 2295635 (**B**^{3c}), 2295631 (**B**^{3s}), 2295632 (**B**^{4b}), 2295630 (**B**^{4e}).

Supplementary Notes

[3] Preparation of reagents

3-1. Preparation of trifluoro(2,3,5,6-tetrachlorophenyl)-λ^{4}-borane, potassium salt

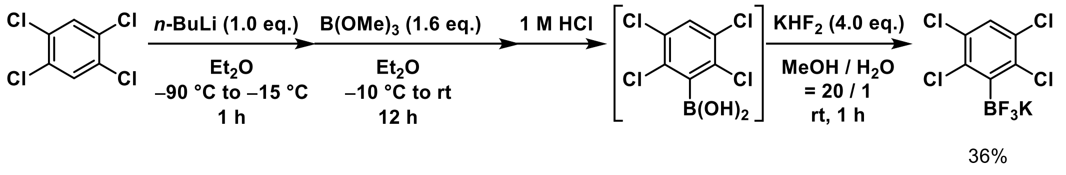

At −90 °C, *n*-BuLi (17.9 mL in *n*-hexane, 1.58 M, 27.8 mmol) was added to 1,2,4,5-tetrachlorobenzene (6.00 g, 27.8 mmol) in Et_2O (50 mL). The mixture was slowly allowed to warm to −10 °C for 1 h. When the solution became clear, $B(OMe)_3$ (4.96 mL, 44.5 mol) was added to this reaction mixture. The mixture was allowed to warm to rt, and stirred for 12 h. Then, HCl (1M 50 mL) was added to the resultant mixture at rt, stirred for 15 min, and the resultant organic layer was extracted

with Et_2O. The combined organic layer was washed with brine, dried over anhydrous Na_2SO_4, and all volatiles were removed in vacuo after filtration. The resultant solid was then washed with *n*-hexane and dried in vacuo, giving a pale yellow solid. A methanol solution of the pale-yellow solid (100 mL) was mixed with H_2O (5 mL) and KHF_2 (6.67 g, 111.0 mmol) and stirred at rt in a PE-bottle. After 12 h of stirring, extraction with acetone through a glass filter and the following removal of all volatiles furnished a white solid. After extraction with acetone, filtration, and evaporation yielded a solid that was purified via the chromatography on a silica gel (*n*-hexane/EtOAc = 1/2) and removal of all volatiles in vacuo afforded trifluoro(2,3,5,6-tetrachlorophenyl)-λ^4-borane, potassium salt as a white solid (3.21 g, 9.99 mmol, 36%) **^{1}H NMR** (400 MHz, DMSO-d_6, rt, δ/ppm): 7.68 (s, 1H, Ar-*H*). **^{11}B NMR** (128 MHz, DMSO-d_6, rt, δ/ppm): 1.68 (q, $^{1}J_{B,F}$ = 29.4 Hz). **^{13}C{^{1}H} NMR** (100 MHz, DMSO-d_6, rt, δ/ppm): 135.1, 130.8, 128.3. A signal of the ipso-carbon with respect to the boron atoms was not identified. **^{19}F NMR** (376 MHz, DMSO-d_6, rt, δ/ppm): −135.7 (q, $^{2}J_{F,F}$ = 41.0 Hz, 3F). HRMS (FAB$^-$): m/z Calculated for $C_6HBCl_4F_3$ ([M]$^-$) 280.8883, found 280.8860.

3-2. Preparation of trifluoro(perchlorophenyl)-λ^4-borane, potassium salt

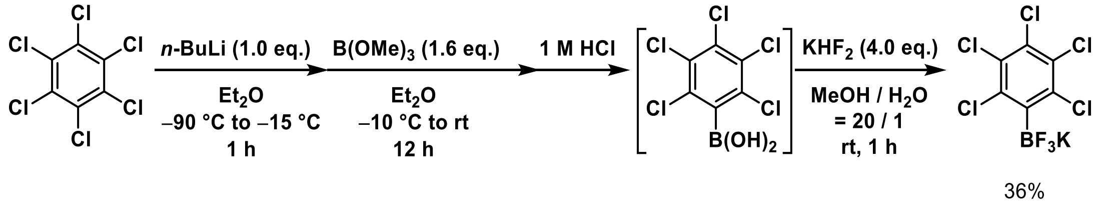

At −90 °C, *n*-BuLi (17.9 mL in *n*-hexane, 1.58 M, 21.1 mmol) was added to perchlorobenzene (6.00 g, 21.1 mmol) in Et_2O (50 mL). The mixture was slowly allowed to warm to −15 °C for 1 h. When the solution became clear, the reaction mixture was treated with $B(OMe)_3$ (3.76 mL, 33.8 mmol). The mixture was allowed to warm to rt, and stirred for 12 h. Then, HCl (1M 50 mL) was added to the resultant mixture at rt, stirred for 15 min, and the resultant organic layer was extracted with Et_2O.The combined organic layer was washed with brine, dried over anhydrous Na_2SO_4, and all volatiles were then washed with hexane and dried in vacuo, giving a pale yellow solid. A methanol solution of the pale-yellow solid (100 mL) was mixed with H_2O (5 mL) and KHF_2 (5.07 g, 84.3 mmol) in a PE-bottle, and stirred at rt. After 12 h of stirring, extraction with acetone through a glass filter and the following removal of all volatiles furnished a white solid. After extraction with acetone, filtration, and evaporation yielded a solid that was purified via the chromatography on a silica gel (*n*-hexane/EtOAc = 1/1) and removal of all volatiles in vacuo afforded trifluoro(perchlorophenyl)-λ^4-borane, potassium salt as a pale yellow solid (2.71 g, 7.61 mmol, 36%). **^{11}B NMR** (128 MHz, C_6D_6, rt, δ/ppm): 1.62 (q, $^{1}J_{B,F}$ = 29.1 Hz). **^{13}C{^{1}H} NMR** (100 MHz, DMSO-d_6, rt, δ/ppm): 136.2, 131.9 (d, J = 23.0 Hz), 130.4, 129.7 (d, J = 21.0 Hz). **^{19}F NMR** (376 MHz, DMSO-d_6, rt, δ/ppm): −132.5 (q, $^{2}J_{F,F}$ = 42.6 Hz, 3F). HRMS (FAB$^-$): m/z Calculated for $C_6BCl_5F_3$ ([M]$^-$) 314.8493, found 314.8488.

3-3. Preparation of 2,6-dichloro-3,5-bis(trifluoromethyl)aniline and 2,4,6-trichloro-3,5-bis(trifluoromethyl)aniline[2]

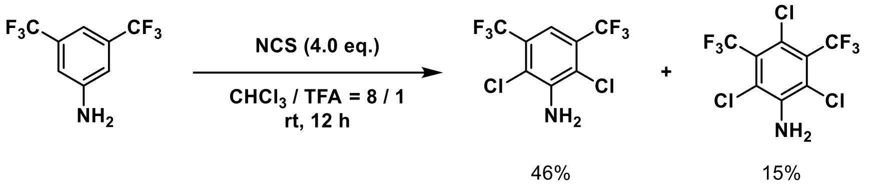

This reaction was carried out under air. To a solution of trifluoroacetic acid (45.7 mL) and N-chlorosuccinimide (77.98 g, 584.8 mmol, 4.0 eq.) in $CHCl_3$ (365 mL) was added 3,5-bis(trifluoromethyl)aniline (33.5 g, 146.2 mmol) at rt. After stirring 12 h, sat. $NaHCO_3$ aq. was added followed by addition of sat. $Na_2S_2O_3$ aq. Then the organic layer was extracted with $CHCl_3$ and washed with brine, dried over anhydrous Na_2SO_4, and all volatiles were removed in vacuo after filtration. Then, distillation (0.2 mmHg, 90 °C) of the resultant mixture was carried out to afford 2,6-dichloro-3,5-bis(trifluoromethyl)aniline as a fraction that eventually became a white solid (19.9 g, 66.8 mmol, 46%). The residue was purified by the chromatography on a silica gel (100% hexane) followed by removal of all volatiles in vacuo, resulting in isolation of 2,4,6-trichloro-3,5-bis(trifluoromethyl)aniline as a pale-yellow solid (7.31 g, 22.0 mmol, 15%).

Identification of 2,6-dichloro-3,5-bis(trifluoromethyl)aniline:

^{1}H NMR (400 MHz, $CDCl_3$, rt, δ/ppm): 7.39 (s, 1H, Ar-*H*), 5.02 (brs, 2H, N*H*). **^{13}C{^{1}H} NMR** (100 MHz, $CDCl_3$, rt, δ/ppm): 143.4, 127.5 (q, $^2J_{C,F}$ = 32.3 Hz), 122.3 (q, $^1J_{C,F}$ = 274 Hz), 120.3, 113.5 (t, $^3J_{C,F}$ = 5.6 Hz). **^{19}F NMR** (376 MHz, $CDCl_3$, rt, δ/ppm): −66.2 (s, 6F). HRMS (EI$^+$): m/z Calculated for $C_8H_3Cl_2F_6N$ ([M]$^+$) 296.9547, found 296.9551.

Identification of 2,4,6-trichloro-3,5-bis(trifluoromethyl)aniline:

^{1}H NMR (400 MHz, $CDCl_3$, rt, δ/ppm): 5.15 (s, 2H, N*H*). **^{13}C{^{1}H} NMR** (100 MHz, $CDCl_3$, rt, δ/ppm): 142.2, 127.0 (q, $^2J_{C,F}$ = 30.1 Hz), 122.4 (q, $^1J_{C,F}$ = 276 Hz), 121.3, 118.3. **^{19}F NMR** (376 MHz, $CDCl_3$, rt, δ/ppm): −57.8 (s, 6F). HRMS (EI$^+$): m/z Calculated for $C_8H_2Cl_3F_6N$ ([M]$^+$) 330.9157, found 330.9164.

3-4. Preparation of 2,4-Cl_2-3-I-1,5-$(CF_3)_2$-C_6H[3]

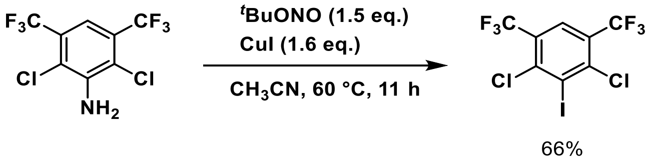

In CH_3CN (65 mL), a mixture of CuI (14.1 g, 78.9 mmol, 1.6 eq.) and 2,6-dichloro-3,5-bis(trifluoromethyl)aniline (14.7 g, 49.3 mmol) was stirred at 60 °C. To this solution, ^{t}BuONO (8.80 mL, 74.2 mmol, 1.5 eq.) was added slowly and the resultant mixture was stirred for 11 h at 60 °C. The reaction was then cooled to rt, diluted with *n*-hexane/EtOAc (v/v = 5/1), and filtered through a celite pad. To the resultant solution, sat. $Na_2S_2O_3$ aq. was added, and washed with brine, dried over anhydrous Na_2SO_4, and all volatiles were removed in vacuo after filtration. The resultant mixture was purified by the chromatography on a silica gel (hexane/EtOAc = 99/1). Removal of all volatiles in vacuo afforded

2,4-Cl_2-3-I-1,5-$(CF_3)_2$-C_6H as a colorless crystal (13.3 g, 32.5 mmol, 66%). **^{1}H NMR** (400 MHz, $CDCl_3$, rt, δ/ppm): 8.05 (s, 1H, Ar-*H*). **^{13}C{^{1}H} NMR** (100 MHz, $CDCl_3$, rt, δ/ppm): 142.8, 127.7 (q, $^2J_{C,F}$ = 32.6 Hz), 126.2−126.0 (m, *C*-H), 121.6 (q, $^1J_{C,F}$ = 274 Hz), 113.0. **^{19}F NMR** (376 MHz, $CDCl_3$, rt, δ/ppm): −66.8 (s, 6F). HRMS (EI^+): m/z Calculated for $C_8HCl_2F_6I$ ($[M]^+$) 407.8404, found 407.8403.

3-5. Preparation of 2,4-F_2-3-I-1,5-$(CF_3)_2$-C_6H[4]

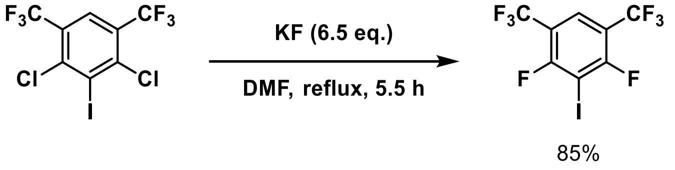

In DMF (6.0 mL), 2,4-Cl_2-3-I-1,5-$(CF_3)_2$-C_6H (14.1 g, 34.5 mmol) and KF (13.0 g, 224.1 mmol, 6.5 eq.) were mixed, and the resultant suspension was stirred at 160 °C for 5.5 h. Then the mixture was allowed to cool to rt. Followed by addition of H_2O (50 mL), and the organic layer was extracted with *n*-hexane/EtOAc (v/v = 5/1) and washed with brine, dried over anhydrous Na_2SO_4, and all volatiles were removed in vacuo after filtration. The resultant mixture was purified by the chromatography on a silica gel (100% hexane). Removal of all volatiles in vacuo afforded 2,4-F_2-3-I-1,5-$(CF_3)_2$-C_6H as a colorless solid (11.0 g, 29.2 mmol, 85%). **^{1}H NMR** (400 MHz, $CDCl_3$, rt, δ/ppm): 7.92 (t, $^4J_{H,F}$ = 7.2 Hz, Ar-*H*). **^{13}C{^{1}H} NMR** (100 MHz, $CDCl_3$, rt, δ/ppm): 162.5 (dd, $^1J_{F,F}$ = 261 Hz, $^4J_{C,F}$ = 5.9 Hz), 126.5 (m, 2H), 121.1 (q, $^2J_{C,F}$ = 273 Hz), 116.2−114.8 (m), 75.3 (t, $^2J_{F,F}$ = 29.6 Hz). **^{19}F NMR** (376 MHz, $CDCl_3$, rt, δ/ppm): −64.7 (d, $^4J_{F,F}$ = 14.4 Hz, 6F), −86.3 (m, 4F). HRMS (EI^+): m/z Calculated for $C_8Cl_3F_6I$ ($[M]^+$) 407.8404, found 407.8403.

3-6. Preparation of 1,3,5-Cl_3-2-I-4,6-$(CF_3)_2$-C_6[4]

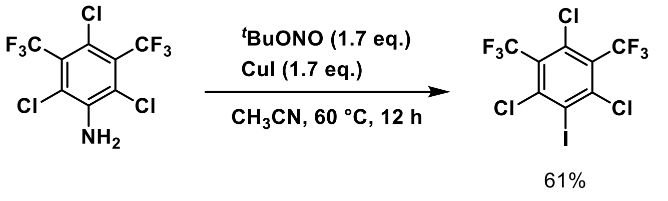

Under N_2 atmosphere, CuI (5.94 g, 31.2 mmol, 1.7 eq.) and 2,4,6-trichloro-3,5-bis(trifluoromethyl)aniline (6.10 g, 18.3 mmol) were dissolved in CH_3CN (40 mL) and stirred at 60 °C. Then, ^{t}BuONO (3.69 mL, 31.2 mmol, 1.7 eq.) was added slowly and the mixture was stirred for 12 h while maintained at 60 °C. The reaction mixture was then cooled to rt, diluted with *n*-hexane/EtOAc (v/v = 5/1), and filtered through a celite pad. To the resultant solution, sat. $Na_2S_2O_3$ aq. was added, and the organic layer was washed with brine, dried over anhydrous Na_2SO_4, and all volatiles were removed in vacuo after filtration. The resultant mixture was purified by the chromatography on a silica gel (hexane/EtOAc = 99/1). Removal of all volatiles in vacuo afforded 1,3,5-Cl_3-2-I-4,6-$(CF_3)_2$-C_6 as a colorless solid (4.91 g, 11.1 mmol, 61%). **^{13}C{^{1}H} NMR** (100 MHz, $CDCl_3$, rt, δ/ppm): 143.5, 136.1, 127.6 (q, $^2J_{C,F}$ = 30.5 Hz), 121.7 (q, $^1J_{C,F}$ = 276 Hz), 113.4. **^{19}F NMR** (376 MHz, $CDCl_3$, rt, δ/ppm): −58.3 (s, 6F). HRMS (EI^+): m/z Calculated for $C_8Cl_3F_6I$ ($[M]^+$) 441.8014, found 441.8024.

3-7. Preparation of (2,6-dichloro-3,5-bis(trifluoromethyl)phenyl)trifluoro-λ[4]-borane

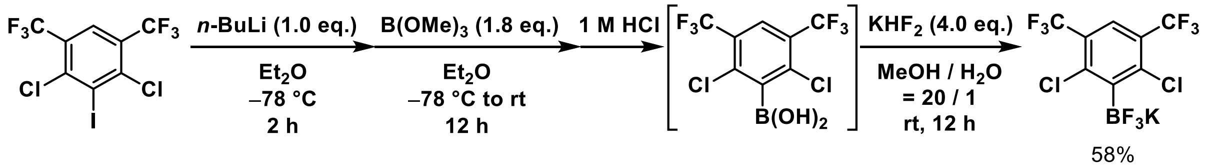

At −78°C, *n*-BuLi (6.06 mL in *n*-hexane, 1.58 M) (9.6 mmol) was added to 2,4-Cl_2-3-I-1,5-$(CF_3)_2$-C_6H (3.92 g, 9.59 mmol) in Et_2O (40 mL) over 15 min. The reaction solution was stirred at −78 °C for 2 h, then $B(Ome)_3$ (1.92 mL, 17.2 mmol, 1.8 eq.) was added to the reaction mixture. The mixture was allowed to warm to rt, and stirred for 12 h. Then, HCl (1M 50 mL) was added to the resultant mixture at rt, stirred for 15 min, and the resultant organic layer was extracted with Et_2O. The combined organic layer was washed with brine, dried over anhydrous Na_2SO_4, and all volatiles were removed in vacuo after filtration. The resultant solid was then washed with hexane and dried in vacuo, giving a pale yellow solid. A methanol solution of the pale yellow solid (100 mL) was mixed with H_2O (5 mL) and KHF_2 (2.30 g, 38.3 mmol, 4.0 eq.) in a PE-bottle, and stirred at rt. After 12 h of stirring, extraction with acetone through a glass filter and the following removal of all volatiles furnished a white solid. After extraction with acetone, filtration, and evaporation yielded a solid that was washed with *n*-hexane followed by dried in vacuo, which afforded (2,6-dichloro-3,5-bis(trifluoromethyl)phenyl)trifluoro-λ[4]-borane, potassium salt as a white solid (2.15 g, 5.53 mmol, 58%) **^{1}H NMR** (400 MHz, DMSO-*d*$_6$, rt, *δ*/ppm): 7.83 (s, 1H, Ar-*H*). **^{11}B NMR** (128 MHz, DMSO-d$_6$, rt, *δ*/ppm): 1.70 (q, $^1J_{B,F}$ = 44.2 Hz). **^{13}C{^{1}H} NMR** (100 MHz, DMSO-*d*$_6$, rt, *δ*/ppm): 141.3, 125.6 (q, $^2J_{C,F}$ = 30.3 Hz), 124.1−123.9 (m), 122.8 (q, $^1J_{C,F}$ = 271 Hz). A signal of the ipso-carbon with respect to the boron atoms was not identified. **^{19}F NMR** (376 MHz, DMSO-*d*$_6$, rt, *δ*/ppm): −64.3 (s, 6F), −134.8 (q, $^2J_{F,F}$ = 41.2 Hz, 3F). HRMS (FAB$^-$): m/z Calculated for $C_8HBF_9Cl_2$ ([M]$^-$) 348.9410, found 348.9398.

3-8. Preparation of trifluoro(2,4,6-trichloro-3,5-bis(trifluoromethyl)phenyl)-λ[4]-borane, potassium salt

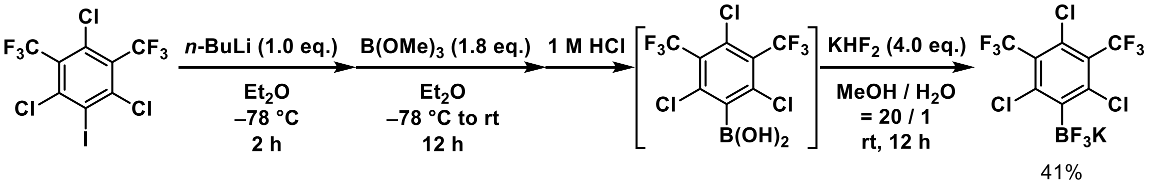

At −78°C, *n*-BuLi (4.64 mL in *n*-hexane, 1.58 M, 7.20 mmol) was added to 1,3,5-Cl_3-2-I-4,6-$(CF_3)_2$-C_6 (3.19 g, 7.20 mmol) in Et_2O (35 mL) over 15 min. The reaction solution was stirred at −78 °C for 2 h, then $B(OMe)_3$ (1.44 mL, 13.0 mmol, 1.8 eq.) was added to the reaction mixture over 5 min. The mixture was allowed to warm to rt, and stirred for 12 h. Then, HCl (1M 50 mL) was added to the resultant mixture at rt, stirred for 15 min, and the resultant organic layer was extracted with Et_2O. The combined organic layer was washed with brine, dried over anhydrous Na_2SO_4, and all volatiles were removed in vacuo after filtration. The resultant solid was washed with *n*-hexane and dried in vacuo, giving a brownish solid. A methanol solution of the solid (100 mL) was mixed with H_2O (5 mL) and KHF_2 (1.73 g, 28.8 mmol, 4.0 eq.) in a PE-bottle, and stirred at rt. After 12 h of stirring, extraction with acetone, filtration, and evaporation yielded a solid that was washed with *n*-hexane followed by dried in vacuo, giving trifluoro(2,4,6-trichloro-3,5-bis(trifluoromethyl)phenyl)-λ[4]-borane, potassium salt as a pale yellow solid (1.26 g, 29.8 mmol, 41%). **^{11}B NMR** (128 MHz, DMSO-*d*$_6$, rt, *δ*/ppm): 1.60 (q, $^1J_{B,F}$ = 42.5 Hz). **^{13}C{^{1}H} NMR** (100 MHz, DMSO-*d*$_6$, rt, *δ*/ppm): 142.9, 131.1, 125.0, 122.7 (q, $^1J_{C,F}$ = 277 Hz). A signal of the ipso-carbon with respect to the boron atoms was not identified. **^{19}F NMR** (376 MHz,

DMSO-d_6, rt, δ/ppm): −56.0 (s, 6F), −134.2 (q, $^2J_{F,F}$ = 41.8 Hz, 3F). HRMS (FAB$^-$): m/z Calculated for $C_8BF_9Cl_3$ ($[M]^-$) 382.9020, found 382.9037.

3-9. Preparation of (2,6-dibromo-4-fluorophenyl)trifluoro-λ^4-borane, potassium salt

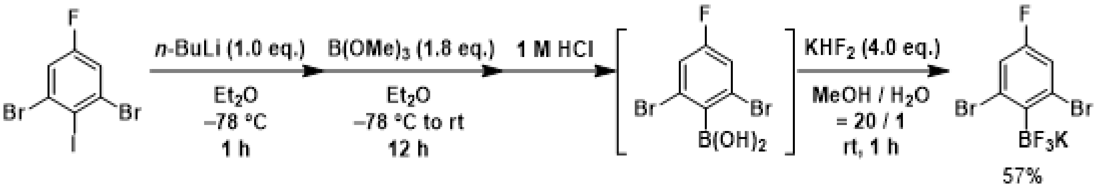

At −78 °C, *n*-BuLi (8.67 mL in *n*-hexane, 1.52 M) (13.2 mmol) was added to 1,3-Br_2-5-F-2-I-C_6H_2 (5.00 g, 13.2 mmol) in Et_2O (40 mL) over 15 min. The reaction solution is stirred at −78 °C for 1 h, then $B(OMe)_3$ (2.64 mL, 23.7 mmol, 1.8 eq.) was added to the reaction mixture. The mixture was allowed to warm to rt and stirred for 12 h. Then, HCl (1M 50 mL) was added to the resultant mixture at rt, stirred for 15 min, and the resultant organic layer was extracted with Et_2O. The combined organic layer was washed with brine, dried over anhydrous Na_2SO_4, and all volatiles were removed in vacuo after filtration. The resultant solid was then washed with hexane and dried in vacuo, giving a pale yellow solid. A methanol solution of the solid (100 mL) was mixed with H_2O (5 mL) and KHF_2 (3.16 g, 52.6 mmol, 4.0 eq.) in a PE-bottle, and stirred at rt. After 12 h of stirring, extraction with acetone, filtration, and evaporation yielded a solid that was washed with *n*-hexane followed by dried in vacuo, giving (2,6-dibromo-4-fluorophenyl)trifluoro-λ^4-borane, potassium salt as a white solid (2.70 g, 7.51 mmol, 57%) **^{1}H NMR** (400 MHz, DMSO-d_6, rt, δ/ppm): 7.30 (d, $^3J_{H,F}$ = 8.8 Hz, 2H, Ar-*H*). **^{11}B NMR** (128 MHz, DMSO-d_6, rt, δ/ppm): 1.71 (q, $^1J_{B,F}$ = 44.7 Hz). **^{13}C{^{1}H} NMR** (100 MHz, DMSO-d_6, rt, δ/ppm): 159.4 (d, $^1J_{C,F}$ = 247 Hz), 127.7 (d, $^3J_{C,F}$ = 9.0 Hz), 119.1 (d, $^2J_{C,F}$ = 22.0 Hz). A signal of the ipso-carbon with respect to the boron atoms was not identified. **^{19}F NMR** (376 MHz, DMSO-d_6, rt, δ/ppm): −119.9 (t, $^2J_{F,H}$ = 8.6 Hz, 1F), −135.7 (q, $^2J_{F,F}$ = 43.4 Hz, 3F). HRMS (FAB$^-$): m/z Calculated for $C_6H_2BF_4Br_2$ ($[M]^-$) 318.8558, found 318.8566.

3-10. Preparation of methyl (4-((tert-butoxycarbonyl)amino)benzoyl)alaninate[5]

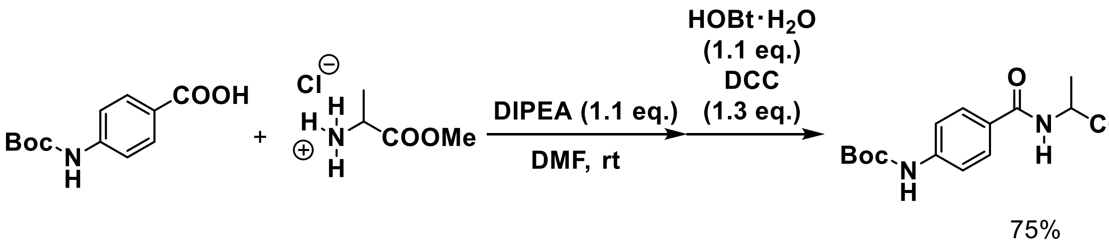

Methyl DL-alaninate hydrochloride (848 mg, 6.32 mmol, 2.0 eq.) was suspended in dry DMF (32 mL) and solubilized by addition of dry DIPEA (1.10 mL, 6.3 mmol, 2.0 eq.). To this solution,. 4-((tert-butoxycarbonyl)amino)benzoic acid (750 mg, 3.16 mmol), HOBt·H_2O (533 mg, 348 mmol, 1.1 eq.), and DCC (848 mg, 4.11 mmol, 1.3 eq.) were added. The solution was stirred 16 h at rt, and then all volatiles were removed in vacuo. The residue was dissolved in EtOAc and washed with brine. The organic layer was dried over anhydrous Na_2SO_4, and the crude product was purified by a flash column chromatography on a silica gel (*n*-hexane/EtOAc = 1/1). Removal of all volatiles in vacuo afforded methyl (4-((tert-butoxycarbonyl)amino)benzoyl)-D-alaninate as a colorless solid (759.1 mg, 2.36 mmol,

75%). **^{1}H NMR** (400 MHz, $CDCl_3$, rt, *δ*/ppm): δ 7.74 (d, *J* = 8.6 Hz, 2H, Ar-*H*), 7.43 (d, *J* = 8.5 Hz, 2H, Ar-*H*), 6.58−6.80 (m, 2H, Ar-*H*), 4.79 (q, *J* = 7.2 Hz, 1H, C*H*CH_3), 3.79 (s, 3H, COOCH_3), 1.52 (s, 9H, C(CH_3)$_3$).

3-11. Preparation of methyl (4-((tert-butoxycarbonyl)amino)benzoyl)alaninate[5]

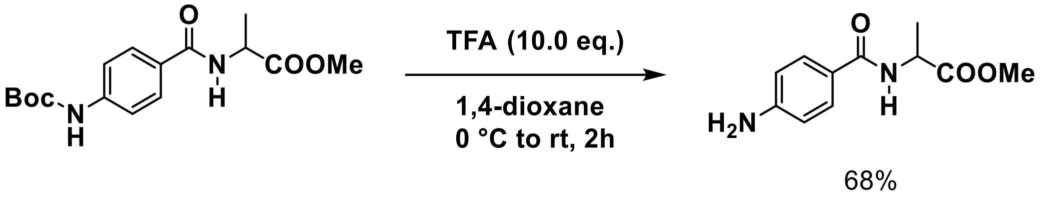

Trifluoroacetic acid (475 μL, 6.2 mmol, 10.0 eq.) was slowly added to a solution of methyl (4-((tert-butoxycarbonyl)amino)benzoyl)alaninate (200 mg, 0.62 mmol) in 1,4-dioxane (10 mL) at 0 °C. The reaction mixture was allowed to warm to rt and stirred for 2 h. After addition of H_2O (2 mL), the solution was evaporated in vacuo to give the crude mixture. The crude products were purified by a flash column chromatography on a silica gel (*n*-hexane/EtOAc = 7/3) afforded methyl (4-aminobenzoyl)alaninate as a white solid (94.2 mg, 0.42 mmol, 68%). **^{1}H NMR** (400 MHz, $CDCl_3$, rt, *δ*/ppm): 7.64 (dd, *J* = 7.1, *J* = 1.9 Hz, 2H, Ar-*H*), 6.67 (d, *J* = 8.6 Hz, 2H, Ar-*H*), 6.53 (m, 1H, Ar-*H*), 4.79 (p, *J* = 7.2 Hz, 1H, C*H*CH_3), 3.78 (s, 3H, COOCH_3), 1.50 (d, *J* = 7.2 Hz, 2H, CHCH_3). A resonance of NH_2 was not identified.

3-12. Preparation of methyl (4-((tert-butoxycarbonyl)amino)benzoyl)methioninate[6]

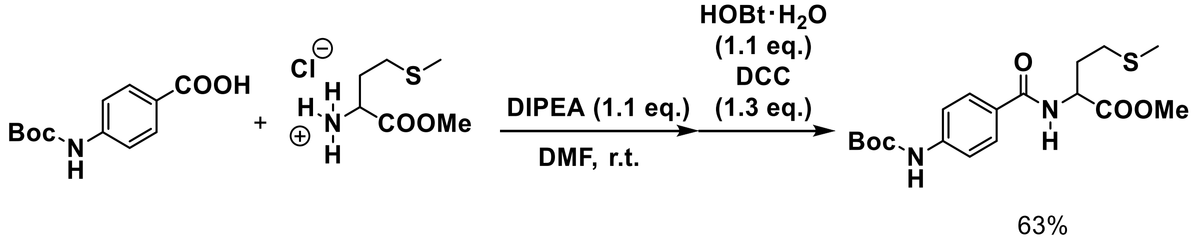

Methyl methioninate hydrochloride (1.80 g, 8.44 mmol, 2.0 eq.) was suspended in dry DMF (30 mL) and solubilized by addition of dry DIPEA (1.47 mL, 8.44 mmol, 2.0 eq.). To this solution, 4-((tert-butoxycarbonyl)amino)benzoic acid (1.0 g, 4.22 mmol), HOBt·H_2O (710 mg, 4.6 mmol, 1.1 eq.) and DCC (1.13 g, 5.48 mmol, 1.3 eq.) were added. The solution was stirred 16 h at rt, and then all volatiles were removed in vacuo. The residue was dissolved in EtOAc and washed with brine. The organic layer was dried over anhydrous Na_2SO_4, and the crude products were purified by a flash column chromatography on a silica gel (*n*-hexane/EtOAc = 1/1). Removal of all volatiles in vacuo afforded methyl (4-((tert-butoxycarbonyl)amino)benzoyl)methioninate as a white solid (1.01 g, 2.64 mmol, 63%). **^{1}H NMR** (400 MHz, $CDCl_3$, rt, *δ*/ppm): δ 7.76 (d, *J* = 8.7 Hz, 2H, Ar-*H*), 7.44 (d, *J* = 8.6 Hz, 2H, Ar-*H*), 6.88 (d, *J* = 7.5 Hz, 1H, Ar-*H*), 6.70 (s, 1H, CON*H*), 4.91 (m, 1H, CON*H*), 3.79 (s, 3H, COOCH_3), 2.52−2.65 (m, 2H, CH_2-S), 2.11 (s, 3H, S-CH_3), 2.05−2.34 (m, 2H, CH-CH_2-CH_2).

3-13. Preparation of methyl (4-aminobenzoyl)methioninate[7]

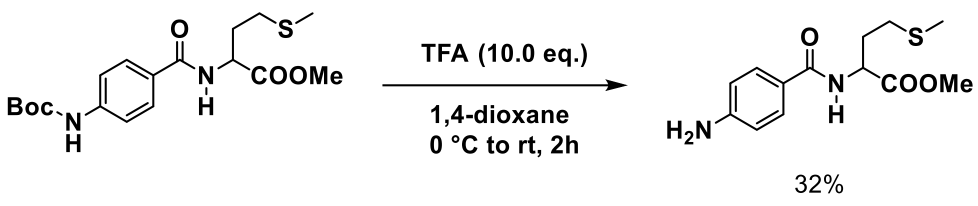

Trifluoroacetic acid (2.0 mL, 26.1 mmol, 10 eq.) was slowly added to a solution of methyl (4-((tert-butoxycarbonyl)amino)benzoyl)methioninate (1.0 g, 2.62 mmol) in 1,4-dioxane (40 mL) at 0 °C. The reaction mixture was allowed to warm to rt and stirred for 2 h. After addition of H_2O (2 mL), the solution was evaporated in vacuo to give the crude mixture. The crude product was purified by a flash column chromatography on a silica gel (*n*-hexane/EtOAc = 1/1), affording methyl (4-aminobenzoyl)alaninate as a white solid (238 mg, 0.84 mmol, 32%). **^{1}H NMR** (400 MHz, $CDCl_3$, rt, δ/ppm): 7.65 (d, J = 8.6 Hz, 2H, Ar-*H*), 6.73 (d, J = 7.7 Hz, 1H, N*H*), 6.67 (d, J = 8.6 Hz, 2H, Ar-*H*), 4.86–4.95 (m, 1H, C*H*CH_2), 3.79 (s, 3H, COOCH_3), 2.52–2.61 (m, 2H, CH_2S), 2.11 (s, 3H, SCH_3), 2.03–2.33 (m, 2H, CHC$H_2$$CH_2$). A resonance of NH_2 was not identified.

3-14. Preparation of **B^{1f}**

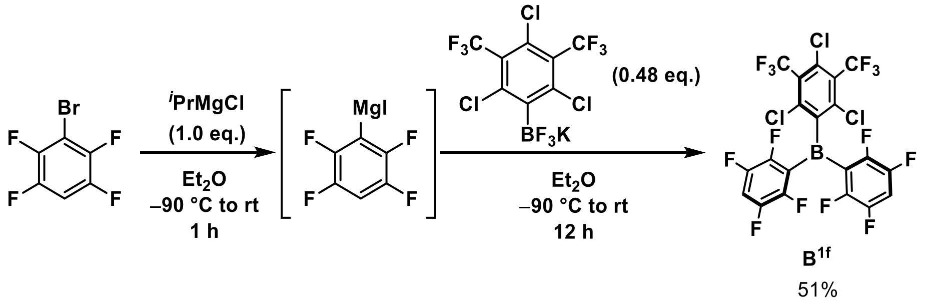

A solution of *p*-C_6F_4HBr (314 μL, 2.56 mmol, 2.29 eq., 0.17 M in Et_2O) was slowly treated with ^{i}PrMgCl (2.56 mL, 2.29 eq., 1.0 M in Et_2O) at −90 °C, and stirred for 1 h (−90 °C to rt).The resultant solution was transferred into a suspension of trifluoro(2,4,6-trichloro-3,5-bis(trifluoromethyl)phenyl)-λ^4-borane, potassium salt (475 mg, 1.12 mmol, 0.22 M in Et_2O) at −90 °C. The reaction mixture was then allowed to warm to rt, and stirred for another 12 h. After the removal of all volatiles in vacuo, the residue was dissolved in *n*-hexane and then passed through a celite pad, concentrated in vacuo and washed with *n*-hexane (cooled to −20 °C prior to use) to afford **B^{1f}** as a white solid (396 mg, 0.57 mmol, 51%) after removal of all volatiles in vacuo. A single crystal of **B^{1f}** was obtained from a saturated solution of **B^{1f}** in toluene layered by *n*-hexane at −20 °C. **^{1}H NMR** (400 MHz, C_6D_6) , rt, δ/ppm): 6.23–6.14 (m, 2H, Ar-*H*). **^{11}B NMR** (128 MHz, C_6D_6, rt, δ/ppm): 63.4 (brs). **^{13}C{^{1}H} NMR** (100 MHz, C_6D_6, rt, δ/ppm): 150.1–149.0 (m), 147.6–147.0 (m), 145.3–144.7 (m), 137.4, 137.0, 127.2, 126.6, 122.5 (q, $^1J_{C,F}$ = 276 Hz), 119.7, 112.6. A signal of the ipso-carbon with respect to the boron atoms was not identified. **^{19}F NMR** (376 MHz, C_6D_6, rt, δ/ppm): −58.0 (s, 6F), −131.7–−131.8 (m, 4F), −140.3–−140.4 (m, 4F). X-ray data for (**B^{1f}**)$_{1/2}$: M = 312.69, colorless, monoclinic, *C*2/c (#15), a = 14.0805(6) Å, b = 11.5258(4) Å, c = 13.9271(6) Å, α = 90°, β = 110.209(5)°, γ = 90°, V = 2121.07(16) Å^3, Z = 8, *Dcalcd* = 1.958 g/cm^3 , T = 173 K, R_1 (wR_2) = 0.0409 (0.1136).

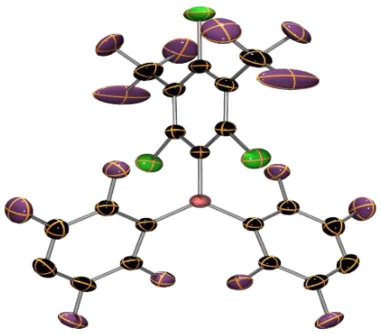

Supplementary Figure 1. Molecular structure of $\mathbf{B^{1f}}$ with ellipsoids set at 50% probability. H atoms are omitted for clarity.

3-15. Preparation of $\mathbf{B^{2b}}$

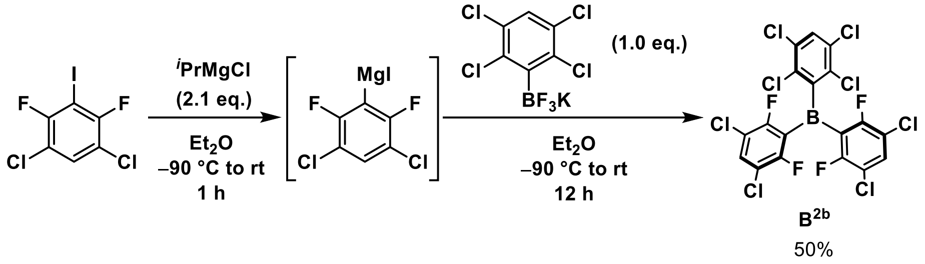

A solution of 1,5-Cl_2-2,4-F_2-3-I-C_6H (1.51 g, 4.9 mmol, 2.1 eq., 0.16 M in Et_2O) was slowly treated with ^{i}PrMgCl (4.90 mL, 2.1 eq., 1.0 M in Et_2O) at −90 °C. After stirred for 1 h (−90 °C to rt), the resultant solution was transferred into a suspension of trifluoro(2,3,5,6-tetrachlorophenyl)-λ^4-borane, potassium salt (750 mg, 2.33 mmol, 0.46 M in Et_2O) at −90 °C. The reaction mixture was then allowed to warm to rt, and stirred for another 12 h. After the removal of all volatiles in vacuo, the residue was dissolved in a small amount of toluene and then passed through a celite pad, then concentrated in vacuo and washed with *n*-hexane (cooled to −20 °C prior to use) to afford $\mathbf{B^{2b}}$ as a white solid (683 mg, 1.16 mmol, 50%) after removal of all volatiles in vacuo. A single crystal of $\mathbf{B^{2b}}$ was obtained from a saturated solution of $\mathbf{B^{2b}}$ in toluene layered by *n*-hexane at rt. **^{1}H NMR** (400 MHz, C_6D_6, rt, δ/ppm): 6.91 (s, 1H, Ar-*H*), 6.82 (t, $^4J_{H,F}$ = 7.6 Hz, 2H, Ar-*H*). **^{11}B NMR** (128 MHz, C_6D_6, rt, δ/ppm): 65.9 (brs). **^{13}C{^{1}H} NMR** (100 MHz, C_6D_6, rt, δ/ppm): 159.3 (dd, $^1J_{C,F}$ = 256 Hz, $^4J_{C,F}$ = 9.8 Hz), 143.9, 136.9 (d, J = 4.8 Hz), 132.3 , 131.9 (d, J = 4.2 Hz), 130.5 , 118.3−117.6 (m), 117.7−117.4 (m). **^{19}F NMR** (376 MHz, C_6D_6, rt, δ/ppm): −103.5 (d, $^4J_{F,F}$ = 6.8 Hz, 4F). X-ray data for $\mathbf{B^{2b}}$: M = 589.61, colorless, monoclinic, $P2_1/c$ (#14), a = 12.7067(3) Å, b = 10.6167(2) Å, c = 16.0053(4), α = 90°, β = 100.320(2)°, γ = 90°, V = 2124.24(8) Å^3, Z = 4, $Dcalcd$ = 1.844 g/cm^3 , T = 123 K, R_1 (wR_2) = 0.0388 (0.1002).

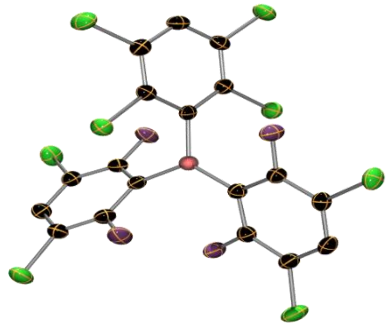

Supplementary Figure 2. Molecular structure of $\mathbf{B^{2b}}$ with ellipsoids set at 50% probability. H atoms are omitted for clarity.

3-16. Preparation of $\mathbf{B^{2c}}$

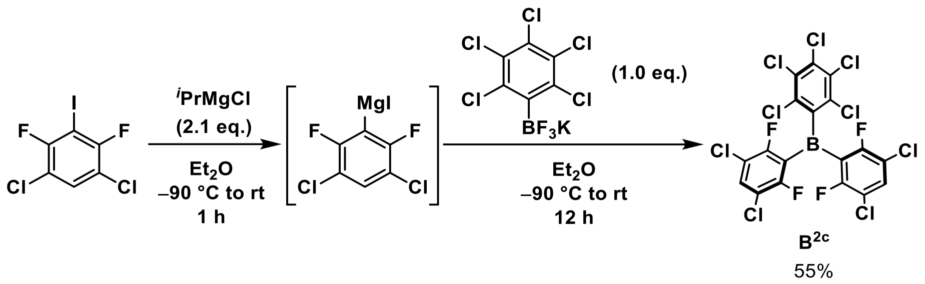

A solution of 1,5-Cl_2-2,4-F_2-3-I-C_6H (1.37 mg, 4.42 mmol, 2.1 eq., 0.15 M in Et_2O) was slowly treated with ^{i}PrMgCl (4.50 mL, 2.1 eq., 1.0 M in Et_2O) at −90 °C, and stirred for 1 h (−90 °C to rt). The resultant solution was transferred into a suspension of trifluoro(perchlorophenyl)-λ^4-borane, potassium salt (750 mg, 2.11 mmol, 0.42 M in Et_2O) at −90 °C. The reaction mixture was then allowed to warm to rt, and stirred for another 12 h. After the removal of all volatiles in vacuo, the residue was dissolved in toluene (10 mL × 4) and then passed through a celite pad, then concentrated in vacuo and washed with *n*-hexane to afford $\mathbf{B^{2c}}$ as a white solid (716 mg, 1.15 mmol, 55%) after removal of all volatiles in vacuo. A single crystal of $\mathbf{B^{2c}}$ was obtained from a saturated solution of $\mathbf{B^{2c}}$ in toluene layered by *n*-hexane at −20 °C. **^{1}H NMR** (400 MHz, C_6D_6, rt, δ/ppm): 6.80 (t, $^{4}J_{H,F}$ = 7.6 Hz, 1H, Ar-*H*). **^{11}B NMR** (128 MHz, C_6D_6, rt, δ/ppm): 61.3 (brs). **^{13}C{^{1}H} NMR** (100 MHz, C_6D_6, rt, δ/ppm): 159.2 (dd, $^{1}J_{C,F}$ = 256 Hz, $^{4}J_{C,F}$ = 9.8 Hz), 141.4 (brs), 137.0 (d, J = 5.3 Hz), 135.2, 132.4, 131.2, 118.3−117.9 (m), 117.6−117.4 (m). **^{19}F NMR** (376 MHz, C_6D_6, rt, δ/ppm): −103.5 (d, $^{4}J_{F,F}$ = 7.5 Hz, 4F). X-ray data for $\mathbf{B^{2c}}$: M = 624.06, colorless, monoclinic, $P2_1/c$ (#14), a = 8.5443(2) Å, b = 19.4719(5) Å, c = 13.7797(3) Å, α = 90°, β = 104.919(2)°, γ = 90°, V = 2215.30(9) Å^3, Z = 4, *Dcalcd* = 1.871 g/cm^3 , T = 123 K, R_1 (wR_2) = 0.0583 (0.1666).

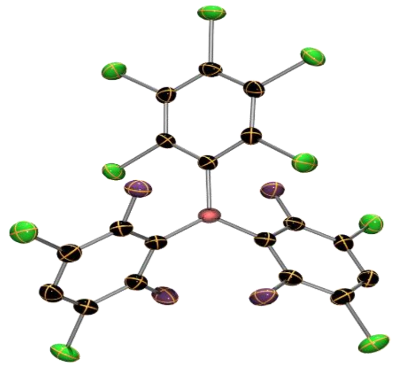

Supplementary Figure 3. Molecular structure of $\mathbf{B^{2c}}$ with ellipsoids set at 50% probability. H atoms are omitted for clarity.

3-17. Preparation of **B^{2e}**

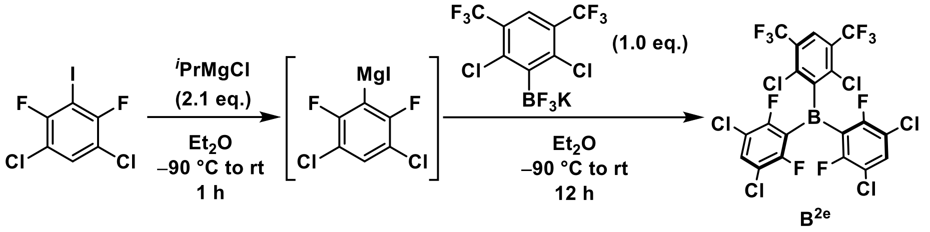

A solution of 1,5-Cl_2-2,4-F_2-3-I-C_6H (833 mg, 2.70 mmol, 2.1 eq., 0.18 M in Et_2O) was slowly treated with ^{i}PrMgCl (2.70 mL, 2.1 eq., 1.0 M in Et_2O) at −90 °C, and stirred for 1 h (−90 °C to rt). The resultant solution was transferred into a suspension of (2,6-dichloro-3,5-bis(trifluoromethyl)phenyl)trifluoro-λ^4-borane, potassium salt (500 mg, 1.29 mmol, 0.16 M in Et_2O) at −90 °C. The reaction mixture was then allowed to warm to rt, and stirred for another 12 h. After the removal of all volatiles in vacuo, the residue was dissolved in toluene (5 mL × 4) and then passed through a celite pad, then concentrated in vacuo and washed with *n*-pentane to afford **B^{2e}** as a white solid (411 mg, 0.57 mmol, 44%) after removal of all volatiles in vacuo. A single crystal of **B^{2e}** was obtained from a saturated solution of **B^{2e}** in toluene layered by *n*-hexane at −20 °C. **^{1}H NMR** (400 MHz, C_6D_6) , rt, *δ*/ppm): 7.47 (s, 1H, Ar-*H*), 6.82−6.78 (m, 2H, Ar-*H*). **^{11}B NMR** (128 MHz, C_6D_6, rt, *δ*/ppm): 63.2 (brs). **^{13}C{^{1}H} NMR** (100 MHz, C_6D_6, rt, *δ*/ppm): 159.2 (dd, $^1J_{C,F}$ = 255 Hz, $^4J_{C,F}$ = 9.7 Hz), 146.5, 137.3, 136.2, 127.4−127.3 (m), 126.3, 122.3 (q, $^1J_{C,F}$ = 272 Hz), 118.4−118.0 (m), 117.5−117.1 (m). **^{19}F NMR** (376 MHz, C_6D_6, rt, *δ*/ppm): −65.3 (s, 6F), −103.7 (s, 4F). X-ray data for **B^{2e}**: M = 656.73, colorless, orthorhombic, *P*bca (#61), a = 15.5239(4) Å, b = 11.5011(3) Å, c = 26.6249(6) Å, α = 90°, β = 90°, γ = 90°, V = 4753.7(2) Å^3, Z = 8, *Dcalcd* = 1.835 g/cm^3, T = 123 K, R_1 (wR_2) = 0.0478 (0.1363).

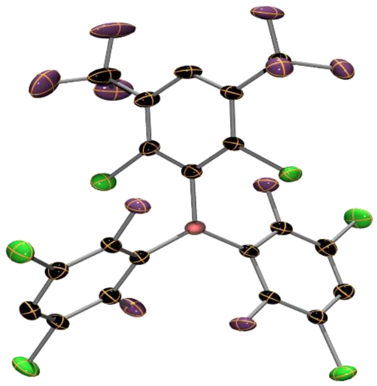

Supplementary Figure 4. Molecular structure of **B^{2e}** with ellipsoids set at 50% probability. H atoms are omitted for clarity.

3-18. Preparation of **B**[3b]

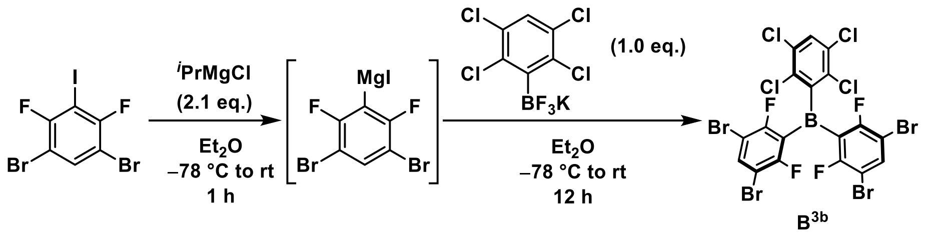

A solution of 1,5-Br$_2$-2,4-F$_2$-3-I-C$_6$H (1.30 g, 3.26 mmol, 2.1 eq., 0.10 M in Et$_2$O) was slowly treated with ^{i}PrMgCl (3.32 mL, 2.1 eq., 1.0 M in Et$_2$O) at −78 °C, and stirred for 1 h (−78 °C to rt). The resultant solution was transferred into a suspension of trifluoro(2,3,5,6-tetrachlorophenyl)-λ^4-borane, potassium salt (500 mg, 1.55 mmol, 0.31 M in Et$_2$O) at −90 °C. The reaction mixture was then allowed to warm to rt, and stirred for another 12 h. After the removal of all volatiles in vacuo, the residue was dissolved in PhCF$_3$ and then passed through a celite pad, then concentrated in vacuo and washed with *n*-pentane to afford **B**^{3b} as a white solid (491 mg, 0.64 mmol, 41%) after removal of all volatiles in vacuo. A single crystal of **B**^{3b} was obtained from a saturated solution of **B**^{3b} in toluene layered by *n*-hexane at −20 °C. **^{1}H NMR** (400 MHz, C$_6$D$_6$) , rt, δ/ppm): 7.21 (t, J = 7.2 Hz, 2H, Ar-*H*), 6.87 (s, 1H, Ar-*H*). **^{11}B NMR** (128 MHz, C$_6$D$_6$, rt, δ/ppm): 58.1 (brs). **^{13}C{^{1}H} NMR** (100 MHz, C$_6$D$_6$, rt, δ/ppm): 160.5 (dd, $^1J_{C,F}$ = 253 Hz, $^4J_{C,F}$ = 9.9 Hz), 143.5, 141.8, 131.9, 131.4 (d, J = 4.3 Hz), 130.1, 117.3, 105.4 (d, J = 26.4 Hz). **^{19}F NMR** (376 MHz, C$_6$D$_6$, rt, δ/ppm): −94.6 (d, $^4J_{F,F}$ = 7.0 Hz, 4F) X-ray data for **[(B^{3b})$_2$·C$_6$H$_{14}$]**: M = 1621.0, colorless, monoclinic, $P2_1$/c (#14), a = 26.6350(3) Å, b = 10.24640(1) Å, c = 19.2265(2) Å, α = 90°, β = 101.1980(10)°, γ = 90°, V = 5147.26(1) Å^3, Z = 4, $Dcalcd$ = 2.092 g/cm^3 , T = 123 K, R_1 (wR_2) = 0.0368 (0.0969).

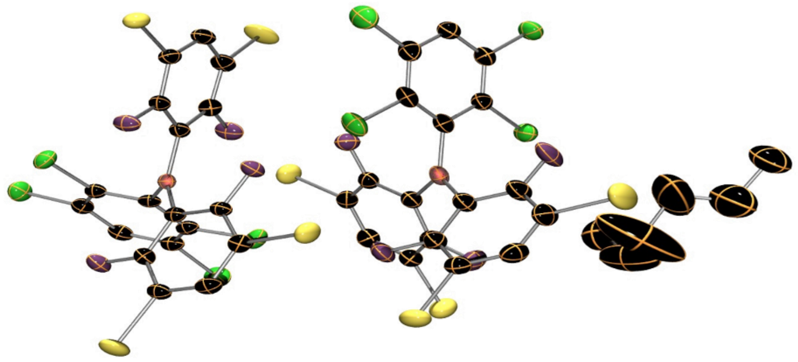

Supplementary Figure 5. Molecular structure of **[(B^{3b})$_2$·C$_6$H$_{14}$]** with ellipsoids set at 50% probability. H atoms are omitted for clarity.

3-19. Preparation of $\mathbf{B^{3c}}$

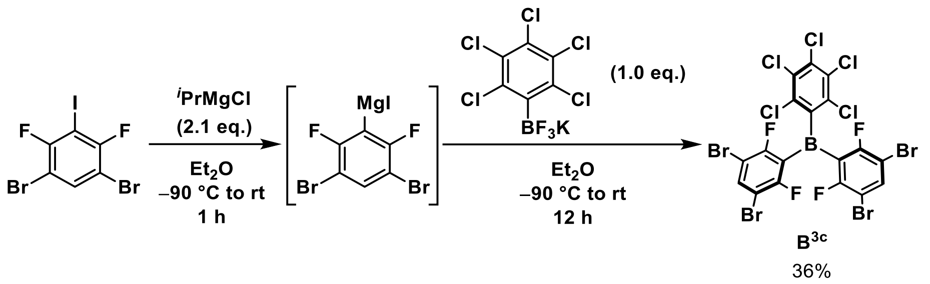

A solution of 1,5-Br_2-2,4-F_2-3-I-C_6H (1.17 g, 2.95 mmol, 2.1 eq., 0.20 M in Et_2O) was slowly treated with ^{i}PrMgCl (3.32 mL, 2.1 eq., 1.0 M in Et_2O) at −78 °C, and stirred for 1 h (−78 °C to rt). The resultant solution was transferred into a suspension of trifluoro(perchlorophenyl)-λ^4-borane, potassium salt (500 mg, 1.40 mmol, 0.28 M in Et_2O) at −90 °C. The reaction mixture was then allowed to warm to rt, and stirred for another 12 h. After the removal of all volatiles in vacuo, the residue was dissolved in $PhCF_3$ (10 mL × 5) and then passed through a celite pad, then concentrated in vacuo and washed with *n*-hexane to afford $\mathbf{B^{3c}}$ as a white solid (411 mg, 0.51 mmol, 36%) after removal of all volatiles in vacuo. A single crystal of $\mathbf{B^{3c}}$ was obtained from a saturated solution of $\mathbf{B^{3c}}$ in toluene layered by *n*-hexane at −20 °C. **^{1}H NMR** (400 MHz, C_6D_6, rt, δ/ppm): 7.19 (t, J = 7.3 Hz, 2H, Ar-H). **^{11}B NMR** (128 MHz, C_6D_6, rt, δ/ppm): 63.9 (brs). **^{13}C{^{1}H} NMR** (100 MHz, C_6D_6, rt, δ/ppm): 160.5 (dd, $^1J_{C,F}$ = 254 Hz, $^4J_{C,F}$ = 9.9 Hz), 141.8, 134.7, 132.0, 130.9, 117.4−117.3 (m), 105.6−105.2 (m). A signal of the ipso-carbon with respect to the boron atoms was not identified. **^{19}F NMR** (376 MHz, C_6D_6, rt, δ/ppm): −94.7 (d, $^4J_{F,F}$ = 7.1 Hz, 4F). X-ray data for $\mathbf{B^{3c}}$: M = 801.86, colorless, monoclinic, $P2_1/c$ (#14), a = 8.61989(3) Å, b = 19.8121(6) Å, c = 14.3343(6) Å, α = 90°, β = 106.628(4)°, γ = 90°, V = 2345.59(15) Å^3, Z = 4, $Dcalcd$ = 2.271 g/cm^3 , T = 123 K, R_1 (wR_2) = 0.0504 (0.1406).

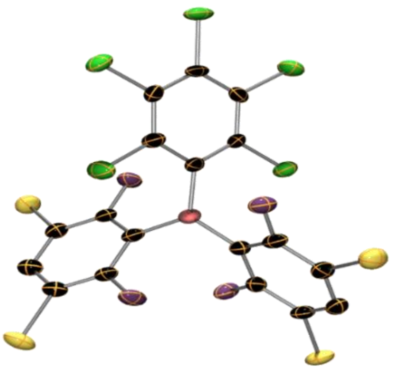

Supplementary Figure 6. Molecular structure of $\mathbf{B^{3c}}$ with ellipsoids set at 50% probability. H atoms are omitted for clarity.

3-20. Preparation of **B**^{3s}

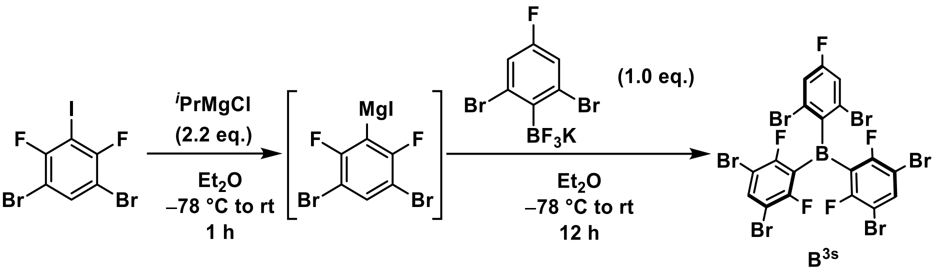

A solution of 1,5-Br_2-2,4-F_2-3-I-C_6H (2.38 g, 5.98 mmol, 2.2 eq., 0.24 M in Et_2O) was slowly treated with ^{i}PrMgCl (5.98 mL, 2.2 eq., 1.0 M in Et_2O) at −78 °C, and stirred for 1 h (−78 °C to rt). The resultant solution was transferred into a suspension of (2,6-dibromo-4-fluorophenyl)trifluoro-λ^4-borane, potassium salt (1.0 g, 2.78 mmol, 0.56 M in Et_2O) at −78 °C. The reaction mixture was then allowed to warm to rt, and stirred for another 12 h. After the removal of all volatiles in vacuo, the residue was dissolved in toluene (10 mL × 4) and then passed through a celite pad, then concentrated in vacuo and washed with *n*-hexane to afford **B**^{3s} as a white solid (1.71 g, 2.12 mmol, 76%) after removal of all volatiles in vacuo. A single crystal of **B**^{3s} was obtained from a saturated solution of **B**^{3s} in toluene layered by *n*-hexane at −20 °C. **^{1}H NMR** (400 MHz, C_6D_6, rt, δ/ppm): 7.27 (t, J = 7.2 Hz, 2H, Ar-*H*), 6.66 (d, J = 7.6 Hz, 2H, Ar-*H*). **^{11}B NMR** (128 MHz, C_6D_6, rt, δ/ppm): 67.0 (brs). **^{13}C{^{1}H} NMR** (100 MHz, C_6D_6, rt, δ/ppm): 163.0 (d, $^{1}J_{C,F}$ = 255 Hz), 160.6 (dd, $^{1}J_{C,F}$ = 253 Hz, $^{4}J_{C,F}$ = 9.9 Hz), 141.6, 141,2, 128.6, 123.3 (d, J = 9.8), 119.0–118.4 (m), 105.8–105.4 (m). **^{19}F NMR** (376 MHz, C_6D_6, rt, δ/ppm): −94.4 (d, $^{4}J_{F,F}$ = 7.2 Hz, 4F), −110.7 (t, $^{3}J_{F,H}$ = 8.1 Hz, 1F). X-ray data for (**B**^{3s})$_2$ M = 1610.96, colorless, triclinic, P-1 (#2), a = 8.4152(2) Å, b = 15.7132(4) Å, c = 16.5779(4) Å, α = 80.300(2)°, β = 84.953(2)°, γ = 81.608(2)°, V = 2133.10(9) Å^3, Z = 2, $Dcalcd$ = 2.508 g/cm^3 , T = 123 K, R_1 (wR_2) = 0.0391 (0.0805).

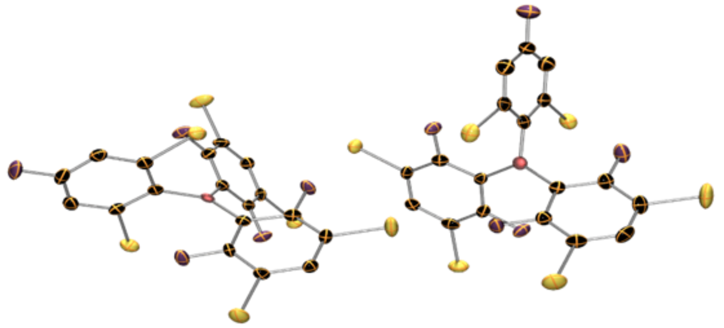

Supplementary Figure 7. Molecular structure of (**B**^{3s})$_2$ with ellipsoids set at 50% probability. H atoms are omitted for clarity.

3-21. Preparation of **B^{4b}**

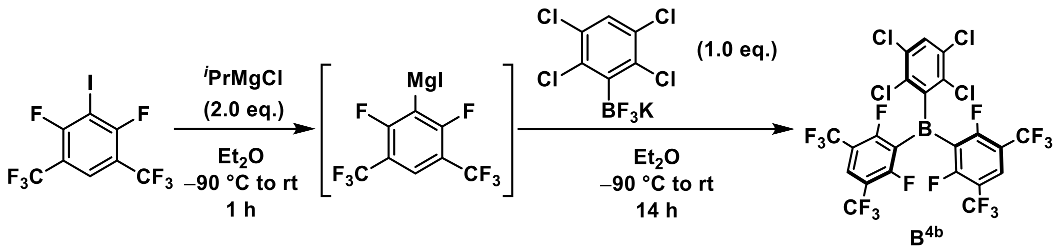

A solution of 2,4-F_2-3-I-1,5-$(CF_3)_2$-C_6H (4.58 g, 12.2 mmol, 2.0 eq., 0.24 M in Et_2O) was slowly treated with ^{i}PrMgCl (12.2 mL, 2.0 eq., 1.0 M in THF) at −90 °C, and stirred for 1 h (−90 °C to rt). The resultant solution was transferred into a suspension of trifluoro(2,3,5,6-tetrachlorophenyl)-λ^4-borane, potassium salt (1.92 mg, 5.97 mmol, 1.20 M in Et_2O) at −90 °C. The reaction mixture was then allowed to warm to rt, and stirred for another 12 h. After the removal of all volatiles in vacuo, the residue was dissolved in toluene and then passed through a celite pad, then concentrated in vacuo. Recrystallization from *n*-hexane at −20 °C afforded **B^{4b}** as a colorless crystal (2.62 g, 3.62 mmol, 61%) after removal of all volatiles in vacuo. **^{1}H NMR** (400 MHz, C_6D_6, rt, δ/ppm): 7.45 (t, $^4J_{H,F}$ = 7.4 Hz, 2H, Ar-*H*), 6.79 (s, 1H, Ar-*H*). **^{11}B NMR** (128 MHz, C_6D_6, rt, δ/ppm): 60.1 (brs). **^{13}C{^{1}H} NMR** (100 MHz, C_6D_6, rt, δ/ppm): 165.1 (dd, $^1J_{C,F}$ = 326 Hz, $^3J_{C,F}$ = 10.7 Hz), 142.3, 132.6 (d, J = 5.8 Hz) , 132.2, 130.1, 124.5, 121.3 (q, $^1J_{C,F}$ = 271 Hz), 117.3−117.1 (m) 116.2−115.2 (m). **^{19}F NMR** (376 MHz, C_6D_6, rt, δ/ppm): −64.5 (d, J = 12.9 Hz, 6F), −95.3 (d, $^4J_{F,F}$ = 5.6 Hz, 4F). X-ray data for **B^{4b}**: M = 723.85, colorless, monoclinic, $P2_1/c$ (#14), a = 8.64150(1) Å, b = 10.1745(2) Å, c = 29.3722(4) Å, α = 90°, β = 94.7370(1)°, γ = 90°, V = 2573.67(7) Å^3, Z = 4, $Dcalcd$ = 1.868 g/cm^3 , T = 173 K, R_1 (wR_2) = 0.0490 (0.1391).

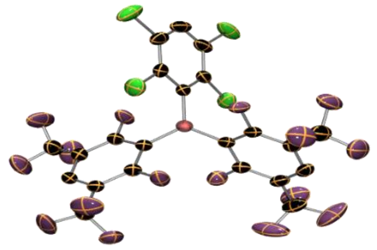

Supplementary Figure 8. Molecular structure of **B^{4b}** with ellipsoids set at 50% probability. H atoms are omitted for clarity.

3-22. Preparation of **B**[4c]

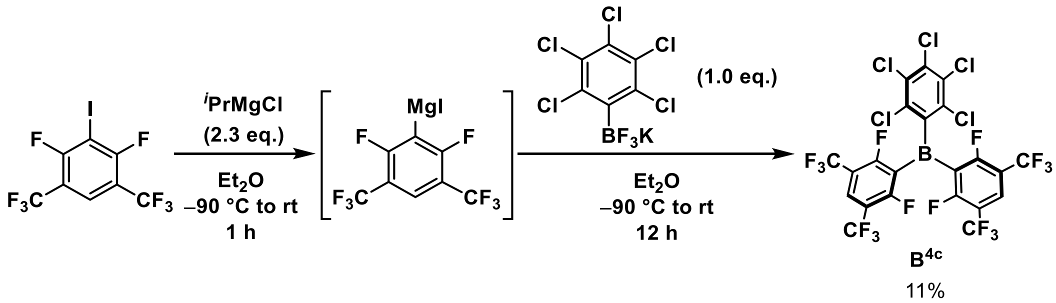

A solution of 2,4-F_2-3-I-1,5-$(CF_3)_2$-C_6H (2.38 g, 6.32 mmol, 2.3 eq., 0.42 M in Et_2O) was slowly treated with ^{i}PrMgCl (6.32 mL, 2.3 eq., 1.0 M in Et_2O) at −90 °C, and stirred for 1 h (−90 °C to rt). The resultant solution was transferred into a suspension of trifluoro(perchlorophenyl)-λ^4-borane, potassium salt (1.00 g, 2.81 mmol, 0.56 M in Et_2O) at −90 °C. The reaction mixture was then allowed to warm to rt, and stirred for another 12 h. After the removal of all volatiles in vacuo, the residue was dissolved in $PhCF_3$ (5 mL × 4) and then passed through a celite pad, then concentrated in vacuo. Recrystallization from *n*-hexane at −20 °C afforded **B**[4c] as a colorless crystal (240 mg, 0.30 mmol, 11%) after removal of all volatiles in vacuo. **^{1}H NMR** (400 MHz, C_6D_6, rt, *δ*/ppm): 7.46 (t, $^4J_{H,F}$ = 7.4 Hz, 2H, Ar-*H*). **^{11}B NMR** (128 MHz, C_6D_6, rt, *δ*/ppm): 64.5 (brs). **^{13}C{^{1}H} NMR** (100 MHz, C_6D_6, rt, *δ*/ppm): 165.1 (d, $^1J_{C,F}$ = 278 Hz), 136.2, 132.8, 132.3, 130.8, 121.5 (q, $^1J_{C,F}$ = 272 Hz), 116.3−115.4 (m). Signals of the ipso-carbon with respect to the boron atoms were not identified. **^{19}F NMR** (376 MHz, C_6D_6, rt, *δ*/ppm): −64.5 (d, *J* = 12.8 Hz, 6F), −95.3 (d, $^4J_{F,F}$ = 5.6 Hz, 4F).

3-23. Preparation of **B**[4e]

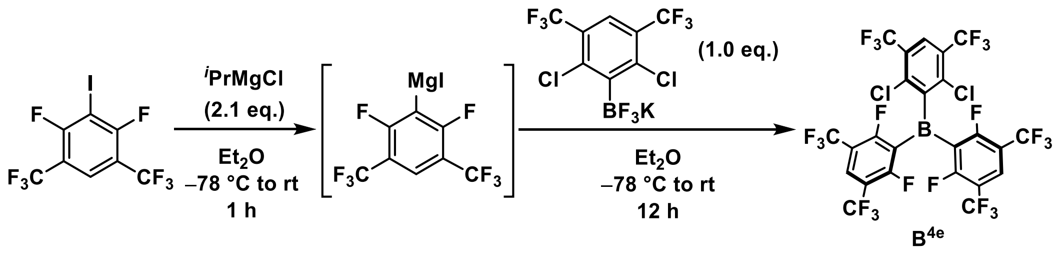

A solution of 2,4-F_2-3-I-1,5-$(CF_3)_2$-C_6H (3.40 g, 9.04 mmol, 2.1 eq., 0.18 M in Et_2O) was slowly treated with ^{i}PrMgCl (9.05 mL, 2.1 eq., 1.0 M in Et_2O) at −78 °C, and stirred for 1 h (−78 °C to rt). The resultant solution was transferred into a suspension of (2,6-dichloro-3,5-bis(trifluoromethyl)phenyl)trifluoro-λ^4-borane, potassium salt (1.42 mg, 4.41 mmol, 0.88 M in Et_2O) at −78 °C. The reaction mixture was then allowed to warm to rt, and stirred for another 12 h. After the removal of all volatiles in vacuo, the residue was dissolved in toluene (5 mL × 4) and then passed through a celite pad, and then concentrated in vacuo. Recrystallization of the resultant solids with *n*-pentane at −20 °C afforded **B**[4e] as a colorless crystal (suitable for XRD analysis), which included trace unidentified impurities. While tried to remove them by continuous extraction and recrystallization from *n*-pentane, we failed to obtain **B**[4e] as an analytically pure sample. Therefore, we used **B**[4e] including trace impurities for the following experiments. **^{1}H NMR** (400 MHz, C_6D_6, rt, *δ*/ppm): 7.53 (t, $^4J_{H,F}$ = 7.8 Hz, 2H, Ar-*H*), 7.44 (s, 1H, Ar-*H*). **^{19}F NMR** (376 MHz, C_6D_6, rt, *δ*/ppm): −64.6–−64.8 (m, 12F), −65.5–−65.7 (s, 6F), −95.5 (s, 4F). Due to a limited amount of a solid, we failed to collect sufficient quality of

^{13}C NMR spectrum. X-ray data for **B^{4e}** : M = 790.97, colorless, orthorhombic, Pbcn (#60), a = 16.8886(5) Å, b = 12.9910(4) Å, c = 12.8554(4)Å, α = 90°, β = 90°, γ = 90°, V = 2820.47(15) Å^3, Z = 4, $Dcalcd$ = 1.863 g/cm^3, T = 193 K, R_1 (wR_2) = 0.0578 (0.1670).

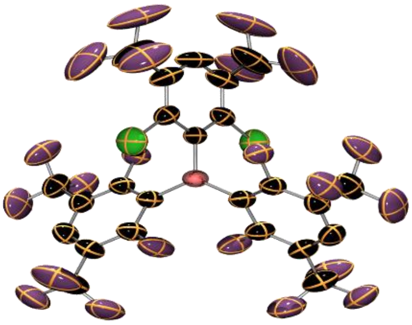

Supplementary Figure 9. Molecular structure of **B^{4e}** with ellipsoids set at 50% probability. H atoms are omitted for clarity.

Supplementary Discussion

[4] Collection of experimental data

A 30 mL autoclave was charged with **1a** (0.40 mmol), **2a** (0.40 mmol), triarylborane (0.02 mmol, 5 mol%), 4Å MS (100 mg) and THF (8 mL). Once sealed, the vessel was pressurized with H_2 (80 atm) and heated at 100 °C for 6 h. The reaction mixture was then cooled to rt, degassed, extracted with acetone, and passed through a glass filter. Subsequently, the filtrate was concentrated in vacuo to give crude mixture. The yield of **3aa** was determined by NMR analysis using 1,3,5-trimethoxybenzen as an internal standard.

[5] Comparison of Lewis acidity among B^{xy} by Gutmann-Beckett method

A J. Young tube was charged with $\mathbf{B^{xy}}$ (0.03 mmol), $Et_3P{=}O$ (1.3 mg, 0.01 mmol, 0.33 eq.), and CD_2Cl_2 (0.5 mL). Then, the ^{31}P NMR analyses were conducted. The acceptor number (AN) was calculated according to the literature and summarized in Supplementary Table 1.[S7]

Supplementary Table 1. Comparison of Lewis acidity based on the Gutmann-Beckett method.

B^{xy}	B^{1a}	B^{1f}	B^{1v}	B^{1w}	B^{2a}	B^{2b}	B^{2c}	B^{2e}	B^{3a}
δ_P	75.0	76.5	71.6	76.1	74.8	75.7	76.0	76.2	74.7
AN	75	79	68	78	75	77	77	78	75

B^{xy}	B^{3b}	B^{3c}	B^{3s}	B^{4b}	B^{4c}	B^{4e}	B^{5a}	B^{6a}	$B(C_6F_5)_3$
δ_P	75.6	75.9	75.3	76.7	76.9	77.2	72.0	73.4	77.7
AN	77	77	76	79	80	80	69	72	81

[6] Effect of solvents

A 30 mL autoclave was charged with **1a** (0.40 mmol), **2a** (0.40 mmol), **B**[4b] (0.02 mmol), 4Å MS (100 mg) and MTHP (8 mL). Once sealed, the vessel was pressurized with H_2 (40 or 80 atm) and heated at 100 °C for 6–24 h. The reaction mixture was then cooled to rt, degassed, extracted with acetone, and passed through a glass filter. Subsequently, the filtrate was concentrated in vacuo to give crude mixture. The yield of **3aa** was determined by NMR analysis using 1,3,5-trimethoxybenzen as an internal standard. Results are summarized in Supplementary Figure 10.

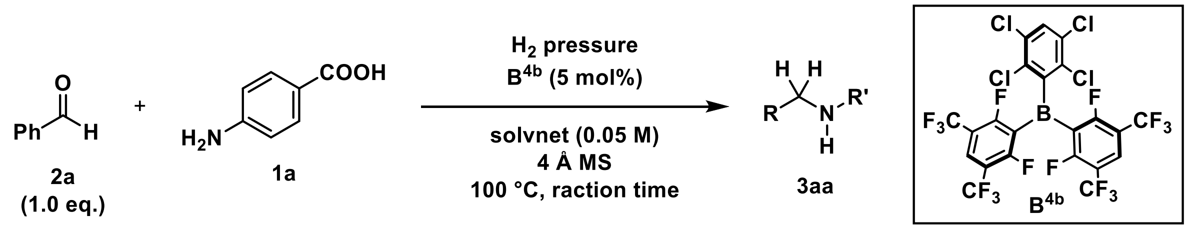

entry	H_2 (atm)	solvent	raction time (h)	MS (mg)	3aa (%)
1	80	THF	6	100	>99
2	40	THF	6	100	50
3	40	MTHP	6	100	59
4	40	1,4-dioxane	6	100	24
5	40	CPME	6	100	18
6	40	2-MeTHF	6	100	38
7	40	2,2,4-Trimethyl-1,3-dioxolane	6	100	-
8	40	MTHP	24	100	72
9	40	MTHP	24	none	42

Supplementary Figure 10. Effect of solvents

[7] Robustness screening with FGE kit

7-1. Exploring functional-group compatibility

A 30 mL autoclave was charged with **1a** (0.40 mmol), **2a** (0.40 mmol), **B^{4b}** (0.02 mmol, 5 mol%), 4 Å MS (100 mg), and MTHP (8 mL). Once sealed, the vessel was pressurized with H_2 (40 or 60 atm) and the reaction mixture was stirred at 100 °C for 24 h. Then degassed at rt followed by addition of acetone, the resultant mixture was filtered to remove MS and other solids when generated. The yield of **3aa** was determined by NMR analysis using 1,3,5-trimethoxybenzen as an internal standard. Results are summarized in Supplementary Table 2.

Supplementary Table 2. Results on robustness screen

Entry	3aa (%)	Additive remaining (%)	Imine (%)	Note
A0-1	72	>99	29	
A0-2	71	>99	27	
A1-1	40	>99	57	
A1-2	31	>99	54	
A2-1	42	>99	52	
A2-2	42	99	51	
A3-1	66	95	27	
A3-2	71	85	20	
A4-1	69	>99	27	
A4-2	63	88	30	
A5-1	0	>99	72	
A5-2	0	>99	81	
A6-1	72	>99	28	
A6-2	70	99	27	
A7-1	75	>99	21	
A7-2	73	98	25	
A8-1	72	>99	30	
A8-2	72	>99	25	
A9-1	69	99	22	
A9-2	71	99	26	
A10-1	53	>99	45	
A10-2	52	>99	44	
A11-1	16	86	62	3-(4-chlorophenoxy)propanal was observed.
A11-2	11	85	55	3-(4-chlorophenoxy)propanal was observed.
A12-1	66	>99	33	
A12-2	65	>99	34	
A13-1	60	87	25	
A13-2	63	87	20	
A14-1	57	99	41	
A14-2	49	>99	43	
A15-1	73	99	26	
A15-2	72	>99	29	
A16-1	70	>99	26	
A16-2	72	95	28	
A17-1	71	89	20	
A17-2	72	94	23	
A18-1	59	>99	38	
A18-2	54	>99	41	
A19-1	33	45	9	
A19-2	32	53	12	
A20-1	67	>99	23	
A20-2	75	99	21	
A21-1	48	>99	44	
A21-2	51	>99	41	

7-2. **B^{4b}**-catalyzed ring opening of **A11**

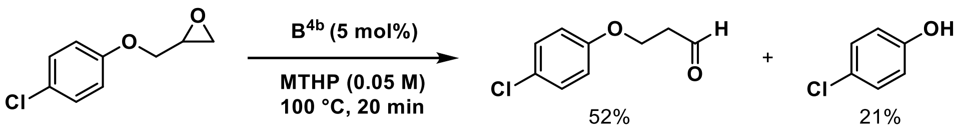

In MTHP (21.6 mL), **A11** (200 mg, 1.08 mmol) and **B^{4b}** (39.2 mg, 54.1 µmol, 5 mol%) was mixed and the solution was stirred at 100 °C for 20 min. Then, H_2O was added, and the organic layer was extracted with EtOAc, washed with brine, dried over anhydrous Na_2SO_4, and filtered. Removal of all volatiles gave a crude mixture which was further purified by a flash column chromatography on a silica gel (hexane/EtOAc = 9/1), giving 3-(4-chlorophenoxy)propanal as colorless liquid (103.5 mg, 0.56 mmol, 52%) and 4-chlorophenol as a colorless solid (29.2 mg, 0.23 mmol, 21%).

Identification of 3-(4-chlorophenoxy)propanal:

^{1}H NMR (400 MHz, $CDCl_3$, rt, δ/ppm): 9.86 (t, J = 1.4 Hz, 1H, C*H*O), 7.23−7.21 (m, 2H, Ar-*H*), 6.85−6.80 (m, 2H, Ar-*H*), 4.27 (t, J = 6.0 Hz, 2H, CH_2), 2.90 (dt, J = 6.0 Hz, J = 1.4 Hz, 2H, CH_2). **^{13}C{^{1}H} NMR** (100 MHz, $CDCl_3$, rt, δ/ppm): 200.0 (d, J = 6.0 Hz), 157.2, 129.5, 126.2, 115.9, 62.0, 43.3. HRMS (EI^+): m/z Calculated for $C_9H_9ClO_2$ ($[M]^+$) 184.0291, found 184.0290.

Identification of 4-chlorophenol[8]

^{1}H NMR (400 MHz, $CDCl_3$, rt, δ/ppm): 7.18 (d, J = 8.8 Hz, 2H, Ar-*H*), 6.77 (d, J = 8.8 Hz, 2H, Ar-*H*), 5.48 (brs, 1H, O*H*).

[8] Reductive alkylation of 1 with 2 for preparation of 3

8-1. Reactions with aniline derived amino acids

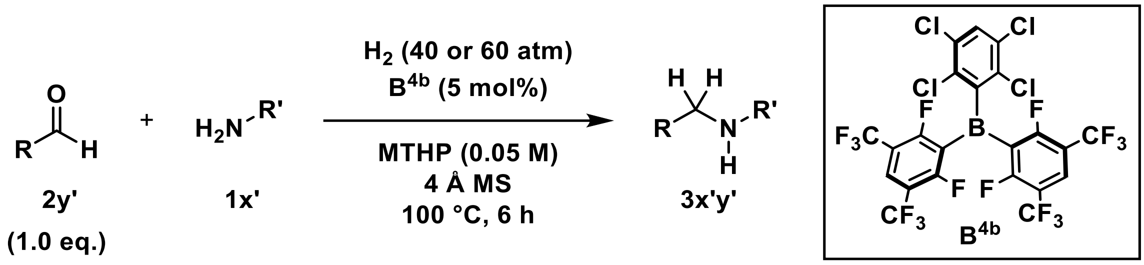

General: A 30 mL autoclave was charged with **1x′** (0.40 mmol), **2y′** (0.40 mmol), **B^{4b}** (0.02 mmol), 4 Å MS (100 mg), and MTHP (8 mL). Once sealed, the vessel was pressurized with H_2 (40 or 60 atm) and the reaction mixture was stirred at 100 °C for 24 h. Then degassed at rt followed by addition of acetone, the resultant mixture was filtered to remove MS and other solids when generated. Subsequently, all volatiles were removed in vacuo to give **3x′y′** that was purified by a flash column chromatography on silica gel.

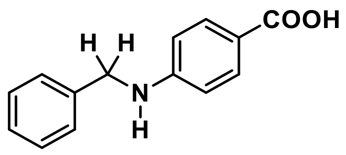

4-(Benzylamino)benzoic acid (3aa):[9] The general procedure was followed with **2a** (42.4 mg, 0.40 mmol), **1a** (54.9 mg, 0.40 mmol), **B^{4b}** (14.5 mg, 0.02 mmol), and H_2 (60 atm). Purification by a flash column chromatography on silica gel (*n*-hexane/EtOAc = 2/1) afforded **3aa** as a colorless solid (85.4 mg, 0.38 mmol, 95%). **^{1}H NMR** (400 MHz, $CDCl_3$, rt, δ/ppm): 7.92 (d, J = 8.8 Hz, 2H, Ar-*H*), 7.39−7.28 (m, 5H, $C_6H_5CH_2$), 6.61 (d, J = 8.8 Hz, 2H, Ar-*H*), 4.41 (s, 2H, CH_2). Resonances of COOH and NH were not identified.

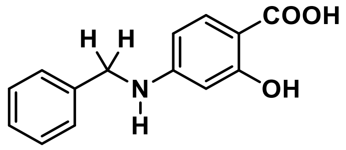

4-(Benzylamino)-2-hydroxybenzoic acid (3ba):[9] The general procedure was followed with **2a** (42.4 mg, 0.40 mmol), **1b** (61.3 mg, 0.40 mmol), **B^{4b}** (14.5 mg, 0.02 mmol), and H_2 (40 atm). Purification by a flash column chromatography on silica gel (*n*-hexane/EtOAc = 1/1) afforded **3ba** as a brown solid (87.1 mg, 0.36 mmol, 90%). **^{1}H NMR** (400 MHz, DMSO-d_6, rt, δ/ppm): δ 12.6 (brs, 1H, COO*H*), 7.43 (d, J = 8.8 Hz, 1H, Ar-*H*). 7.34–7.32 (m, 4H, Ar-*H*), 7.27–7.22 (m, 1H, Ar-*H*), 7.08 (brs, J = 5.6 Hz, 1H, N-*H*), 6.19 (d, J = 8.8, J = 2.4 Hz, 1H, Ar-*H*), 5.93 (d, J = 2.0 Hz, 1H, Ar-*H*), 4.31 (d, J = 6.0 Hz, 2H, CH_2). Resonances of OH were not identified.

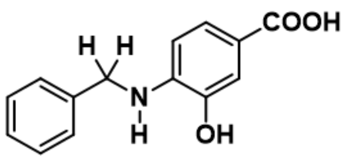

4-(Benzylamino)-3-hydroxybenzoic acid (3ca):[9] The general procedure was followed with **2a** (42.4 mg, 0.40 mmol), **1c** (61.3 mg, 0.40 mmol), **B[4b]** (14.5 mg, 0.02 mmol), and H_2 (40 atm). Purification by a flash column chromatography on silica gel (*n*-hexane/EtOAc = 1/1) afforded **3ca** as a pink solid (88.9 mg, 0.37 mmol, 93%). **^{1}H NMR** (400 MHz, DMSO-d_6, rt, δ/ppm): δ 12.0 (brs, 1H, COO*H*), 7.63 (d, *J* = 8.7 Hz, 2H, Ar-*H*), 7.36−7.28 (m, 4H, Ar-*H*), 7.26−7.20 (m, 1H, Ar-*H*), 7.02 (t, *J* = 6.0 Hz, 1H, N*H*), 6.59 (d, *J* = 8.8 Hz, 2H, Ar-*H*), 4.33 (d, *J* = 6.0 Hz, 2H, CH_2). A resonance of OH was not identified.

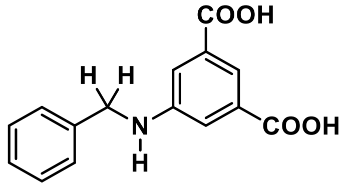

5-(Benzylamino)isophthalic acid (3da): The general procedure was followed with **2a** (42.4 mg, 0.40 mmol), **1d** (72.5 mg, 0.40 mmol), **B[4b]** (14.5 mg, 0.02 mmol), and H_2 (40 atm). Purification by a flash column chromatography on silica gel (*n*-hexane/EtOAc = 1/3) afforded **3da** as a colorless solid (104.1 mg, 0.38 mmol, 95%). **^{1}H NMR** (400 MHz, DMSO-d_6, rt, δ/ppm): 12.9 (brs, 2H, COO*H*), 7.69−7.66 (m, 1H, Ar-*H*), 7.28−7.38 (m, 6H, Ar-*H*), 7.19−7.26 (m, 1H, Ar-*H*), 6.85 (t, *J* = 5.4 Hz, 1H, N*H*), 4.34 (d, *J* = 5.2 Hz, 2H, CH_2). **^{13}C{^{1}H} NMR** (100 MHz, DMSO-d_6, rt, δ/ppm): 167.1, 149.0, 139.4, 131.7, 128.4, 127.1, 126.8, 117.5, 116.6, 46.3. HRMS (EI$^+$): m/z Calculated for $C_{15}H_{13}NO_4$ ($[M]^+$) 271.0845, found 271.0850.

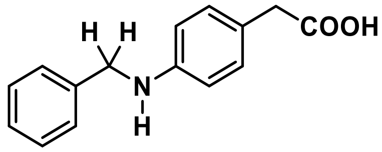

2-(4-(Benzylamino)phenyl)acetic acid (3ea): The general procedure was followed with **2a** (42.4 mg, 0.40 mmol), **1e** (60.5 mg, 0.40 mmol), **B[4b]** (14.5 mg, 0.02 mmol), and H_2 (40 atm). Purification by a flash column chromatography on silica gel (*n*-hexane/EtOAc = 1/3) afforded **3ea** as a pink solid (88.8 mg, 0.37 mmol, 93%).**^{1}H NMR** (400 MHz, DMSO-d_6, rt, δ/ppm): 12.1 (brs, 1H, COO*H*), 7.37−7.15 (m, 5H, C_6H_5-CH_2), 6.91 (d, *J* = 8.4 Hz, 2H, Ar-*H*), 6.50 (d, *J* = 8.4 Hz, 2H, Ar-*H*), 6.15 (brs, 1H, N*H*), 4.24 (s, 2H, NHCH_2), 3.32 (s, 2H, CH_2COOH, (overlap with a H_2O peak)). **^{13}C{^{1}H} NMR** (100 MHz, DMSO-d_6, rt, δ/ppm): 173.3, 147.4, 140.3, 129.7, 128.2, 127.1, 126.6, 121.9, 112.2, 46.5. HRMS (EI$^+$): m/z Calculated for $C_{15}H_{15}NO_2$ ($[M]^+$) 241.1103, found 241.1093.

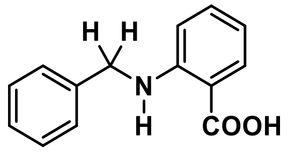

2-(Benzylamino)benzoic acid (3fa):[10] The general procedure was followed with **2a** (42.4 mg, 0.40 mmol), **1f** (54.9 mg, 0.40 mmol), **B[4b]** (14.5 mg, 0.02 mmol), and H_2 (40 atm). Purification by a flash column chromatography on silica gel (*n*-hexane/EtOAc = 1/1) afforded **3fa** as a colorless solid (77.01 mg, 0.34 mmol, 85%).**^{1}H NMR** (400 MHz, DMSO-d_6, rt, δ/ppm): 8.30 (brs, 1H, COO*H*), 7.80 (d, *J* = 7.8 Hz, 2H, Ar-*H*), 7.19−7.38 (m, 6H), 6.66 (d, *J* = 8.5 Hz, 1H, Ar-*H*), 6.55 (t, *J* = 7.5 Hz, 1H, N*H*), 4.45 (s, 2H).

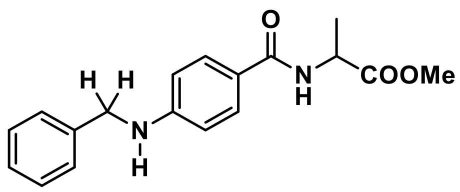

Methyl(4-(benzylamino)benzoyl)-D-alaninate (3ia): The general procedure was followed with **2a** (42.4 mg, 0.40 mmol), **2i** (88.9 mg, 0.40 mmol), **B[4b]** (14.5 mg, 0.02 mmol), and H_2 (40 atm). Purification by a flash column chromatography on silica gel (*n*-hexane/EtOAc = 99/1)

afforded **3ia** as a white solid (117.2 mg, 0.38 mmol, 95%). ^{1}**H NMR** (400 MHz, $CDCl_3$, rt, δ/ppm): 7.65 (dd, J = 6.9, J = 1.9 Hz, 1H, Ar-H), 7.39–7.26 (m, 5H), 6.61 (dd, J = 6.9, J = 1.9 Hz, 1H, Ar-H), 6.52 (d, J = 7.0 Hz, 1H, CONH), 4.78 (q, J = 7.2 Hz, 1H, CHCH$_3$), 4.42 (brs, 1H, NH), 4.38 (s, 2H, CH_2), 3.78 (s, 3H, COOCH_3), 1.49 (d, J = 7.2 Hz, 3H, CHCH_3). A resonance of NH was not identified. ^{13}**C{^{1}H} NMR** (100 MHz, DMSO-d_6, rt, δ/ppm): 174.0, 166.6, 150.9, 138.6, 128.8, 128.7, 128.4 (d, J = 7.5 Hz), 122.2, 111.9, 52.5 (d, J = 8.2 Hz), 48.3 (d, J = 2.2 Hz), 47.7, 18.8 (d, J = 4.9 Hz). HRMS (EI$^+$): m/z Calculated for $C_{18}H_{20}N_2O_3$ ([M]$^+$) 312.1474, found 312.1468.

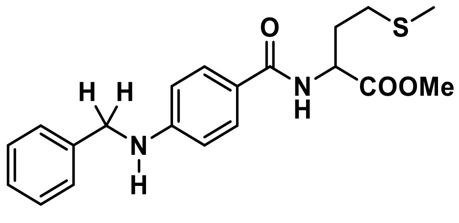

Methyl (4-(benzylamino)benzoyl)methioninate (3ja): The general procedure was followed with **2a** (42.4 mg, 0.40 mmol), **1j** (112.9 mg, 0.40 mmol), **B**^{4b} (14.5 mg, 0.02 mmol), and H_2 (40 atm). Purification by a flash column chromatography on silica gel (n-hexane/EtOAc = 7/3) afforded **3ja** as a white solid (81.2 mg, 0.22 mmol, 55%). ^{1}**H NMR** (400 MHz, $CDCl_3$, rt, δ/ppm): 7.56 (d, J = 8.7 Hz, 2H, Ar-H), 7.31–7.18 (m, 5H, Ar-H), 6.67 (d, J = 7.6 Hz, 1H, CONH), 6.52 (d, J = 8.8 Hz, 2H, Ar-H), 4.75–4.84 (m, 1H, CHCH$_2$), 4.30 (s, 2H, C_6H_5CH_2), 3.68 (s, 3H, COOCH_3), 2.55–2.40 (m, 2H, CH_2S), 2.23–1.93 (m, 2H, CHCH_2), 2.01 (s, 3H, SCH_3). A resonance of NH was not identified. ^{13}**C{^{1}H} NMR** (100 MHz, $CDCl_3$, rt, δ/ppm): 173.0, 167.1, 151.1, 138.6, 129.0, 128.9, 127.6, 127.5, 122.0, 112.0 (d, J = 4.8 Hz), 52.7 (d, J = 9.3 Hz), 52.0 (d, J = 4.7 Hz), 47.8 (t, J = 5.8 Hz), 31.9, 30.2, 15.6. HRMS (EI$^+$): m/z Calculated for $C_{20}H_{24}N_2O_3NaS$ ([M]$^+$) 395.1400, found 395.1381.

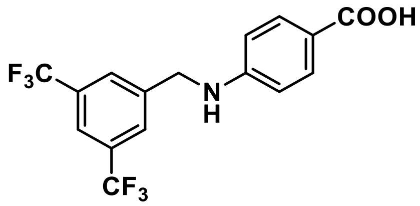

4-((3,5-Bis(trifluoromethyl)benzyl)amino)benzoic acid (3ab): The general procedure was followed with **1a** (54.9 mg, 0.40 mmol), 3,5-bis(trifluoromethyl)benzaldehyde **2b** (96.8 mg, 0.40 mmol), **B**^{4b} (14.5 mg, 0.02 mmol), and H_2 (40 atm). Purification by a flash column chromatography on silica gel (n-hexane/EtOAc = 9/1) afforded **3ab** as a white solid (135.3 mg, 0.37 mmol, 93%). ^{1}**H NMR** (400 MHz, $CDCl_3$, rt, δ/ppm): 7.94 (d, 2H, Ar-H), 7.81 (m, 3H, Ar-H), 6.60 (d, J = 8.6 Hz, 2H, Ar-H), 4.71 (brs, 1H, NH), 4.56 (s, 2H, CH_2). Resonances of COOH were not identified. ^{13}**C{^{1}H} NMR** (100 MHz, $CDCl_3$, rt, δ/ppm): 171.8, 151.7, 141.4, 132.6, 132.3 (d, $^2J_{C,F}$ = 33.1 Hz), 127.3, 123.3 (q, 2C, $^1J_{C,F}$ = 272 Hz), 121.8, 119.0, 112.1, 47.1. ^{19}**F NMR** (376 MHz, $CDCl_3$, rt, δ/ppm): −66.1 (s, 6F). HRMS (EI$^+$): m/z Calculated for $C_{16}H_{10}F_6NO_2$ ([M]$^+$) 362.0610, found 362.0621.

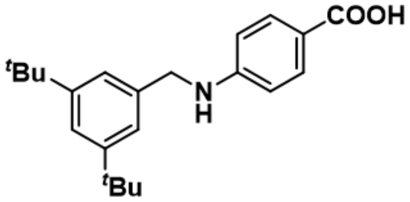

4-((3,5-Bis(trifluoromethyl)benzyl)amino)benzoic acid (3ac): The general procedure was followed with **1a** (54.9 mg, 0.40 mmol), **2c** (87.3 mg, 0.40 mmol), **B**^{4b} (14.5 mg, 0.02 mmol), and H_2 (40 atm). Purification by a flash column chromatography on silica gel (n-hexane only) afforded **3ac** as a white solid (50.8 mg, 0.15 mmol, 38%). ^{1}**H NMR** (400 MHz, $CDCl_3$, rt, δ/ppm): 7.98 (d, J = 8.4 Hz, 2H, Ar-H), 7.41 (s, 1H, NH), 7.22 (d, J = 1.6 Hz, 2H, Ar-H), 6.66 (d, J = 8.8 Hz, 2H, Ar-H), 4.38 (s, 2H, CH_2), 1.35 (s, 18H, C(CH_3)$_3$). Resonances of COOH were not identified. ^{13}**C{^{1}H} NMR** (100 MHz, $CDCl_3$, rt, δ/ppm): 172.6, 152.7, 151.6, 137.3, 132.5, 122.2, 121.9, 117.7, 111.7, 48.6, 35.0, 31.6 (d, J = 4.9 Hz). HRMS

(EI$^+$): m/z Calculated for $C_{22}H_{28}NO_2$ ([M]$^+$) 338.2115, found 338.2123.

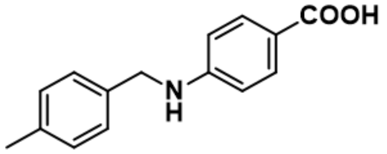

4-((4-Methylbenzyl)amino)benzoic acid (3ad): The general procedure was followed with **1a** (54.9 mg, 0.40 mmol), **2d** (48.1 mg, 0.40 mmol), **B^{4b}** (14.5 mg, 0.02 mmol), and H_2 (40 atm). Purification by a flash column chromatography on silica gel (*n*-hexane/EtOAc = 9/1) afforded **3ad** as a pink solid (74.1 mg, 0.32 mmol, 80%). **^{1}H NMR** (400 MHz, $CDCl_3$, rt, δ/ppm): 7.92 (d, J = 8.8 Hz, 2H, Ar-*H*), 7.26−7.15 (m, 4H, Ar-*H*), 6.60 (d, J = 8.8 Hz, 2H, Ar-*H*), 4.36 (s, 2H, C*H₂*), 2.35 (s, 3H, C*H₃*). Resonances of COOH were not identified. **^{13}C{^{1}H} NMR** (100 MHz, $CDCl_3$, rt, δ/ppm): 172.0, 152.6, 137.5, 135.3, 132.5, 129.6, 127.6, 117.7, 111.8, 47.6, 21.2. HRMS (EI$^+$): m/z Calculated for $C_{15}H_{14}NO_2$ ([M]$^+$) 240.1019, found 240.1030.

8-2. Reactions with substituted anilines

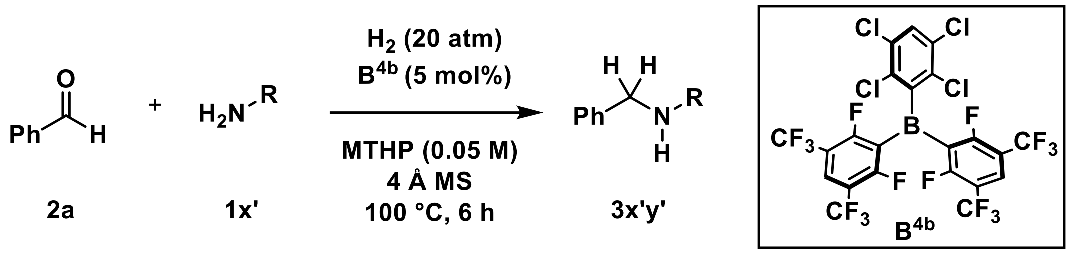

General: A 30 mL autoclave was charged with **1x'** (0.40 mmol), **2y'** (0.40 mmol), **B^{4b}** (0.02 mmol), 4 Å MS (100 mg), and MTHP (8 mL). Once sealed, the vessel was pressurized with H_2 (20 atm) and the reaction mixture was stirred at 100 °C for 6 h. Then degassed at rt followed by addition of acetone, the resultant mixture was filtered to remove MS and other solids when generated. Subsequently, all volatiles were removed in vacuo to give **3x'y'** that was purified by a flash column chromatography on silica gel.

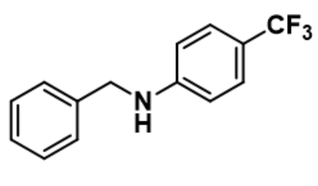

***N*-Benzyl-4-(trifluoromethyl)aniline (3ka):**[9] The general procedure was followed with **2a** (42.4 mg, 0.40 mmol), **1k** (64.4 mg, 0.40 mmol), **B^{4b}** (14.5 mg, 0.02 mmol), and H_2 (20 atm). Purification by a flash column chromatography on silica gel (*n*-hexane only) afforded **3ka** as a white solid (93.1 mg, 0.37 mmol, 93%). **^{1}H NMR** (400 MHz, $CDCl_3$, rt, δ/ppm): 7.41–7.28 (m, 7H, Ar-*H*), 6.63 (d, J = 8.8 Hz, 2H, Ar-*H*), 4.38 (brs, 3H, C*H*₂ and N*H*).

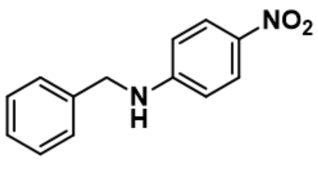

***N*-Benzyl-4-nitroaniline (3la):**[9] The general procedure was followed with **2a** (42.4 mg, 0.40 mmol), **1l** (55.2 mg, 0.40 mmol), **B^{4b}** (14.5 mg, 0.02 mmol), and H_2 (20 atm). Purification by a flash column chromatography on silica gel (*n*-hexane/EtOAc = 1/1) afforded **3la** as a yellow solid (90.3 mg, 0.40 mmol, >99%). **^{1}H NMR** (400 MHz, $CDCl_3$, rt, δ/ppm): 8.11–8.07 (m, 2H, Ar-*H*), 7.40–7.30 (m, 5H, Ar-*H*), 6.60–6.56 (m, 2H, Ar-*H*), 4.84 (brs, 1H, N*H*), 4.43 (d, J = 5.6 Hz, 2H, C*H*₂).

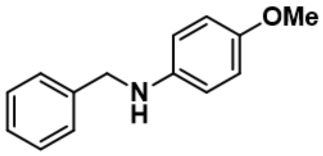

***N*-Benzyl-4-methoxyaniline (3ma):**[9] The general procedure was followed with **2a** (42.4 mg, 0.40 mmol), **1m** (49.3 mg, 0.40 mmol), **B**[4b] (14.5 mg, 0.02 mmol), and H_2 (20 atm). Purification by a flash column chromatography on silica gel (*n*-hexane only) afforded **3ma** as a white solid (76.0 mg, 0.36 mmol, 90%). **^{1}H NMR** (400 MHz, $CDCl_3$, rt, δ/ppm): 7.47–7.42 (m, 4H, Ar-*H*), 7.38–7.35 (m, 1H, Ar-*H*), 6.88 (d, *J* = 8.8 Hz, 2H, Ar-*H*), 6.68 (d, *J* = 8.8 Hz, 2H, Ar-*H*), 4.35 (s, 2H, CH_2), 3.81 (brs, 4H, OCH_3 and N*H*).

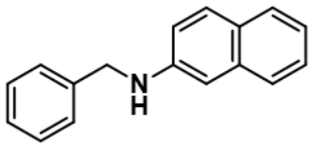

***N*-Benzylnaphthalen-2-amine (3na):**[9] The general procedure was followed with **2a** (42.4 mg, 0.40 mmol), **1n** (87.3 mg, 0.40 mmol), **B**[4b] (14.5 mg, 0.02 mmol), and H_2 (20 atm). Purification by a flash column chromatography on silica gel (*n*-hexane only) afforded **3na** as a brownish solid (92.6 mg, 0.40 mmol, >99%). **^{1}H NMR** (400 MHz, $CDCl_3$, rt, δ/ppm): 7.69–7.58 (m, 3H, Ar-*H*), 7.44–7.28 (m, 6H, Ar-*H*), 7.22–7.18 (m, 1H, Ar-*H*), 6.94–6.91 (m, 1H, Ar-*H*), 6.85 (d, *J* = 2.0 Hz, 1H, Ar-*H*), 4.45 (s, 2H, CH_2), 4.21 (brs, 1H, N*H*).

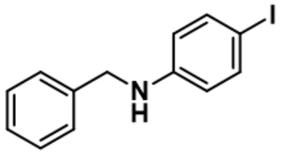

***N*-Benzyl-4-iodoaniline (3oa):**[9] The general procedure was followed with **2a** (42.4 mg, 0.40 mmol), **1o** (87.3 mg, 0.40 mmol), **B**[4b] (14.5 mg, 0.02 mmol), and H_2 (20 atm). Purification by a flash column chromatography on silica gel (*n*-hexane only) afforded **3oa** as a pale yellow solid (120.3 mg, 0.39 mmol, 98%). **^{1}H NMR** (400 MHz, $CDCl_3$, rt, δ/ppm): 7.43–7.39 (m, 2H, Ar-*H*), 7.37–7.27 (m, 5H, Ar-*H*), 6.43–6.40 (m, 2H, Ar-*H*), 4.30 (d, *J* = 5.2 Hz, 2H, CH_2), 4.10 (brs, 1H, N*H*).

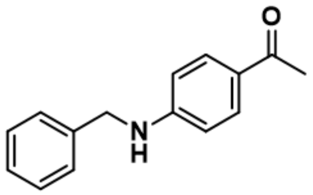

1-(4-(Benzylamino)phenyl)ethan-1-one (3pa):[9] The general procedure was followed with **2a** (42.4 mg, 0.40 mmol), **1p** (54.1 mg, 0.40 mmol), **B**[4b] (14.5 mg, 0.02 mmol), and H_2 (20 atm). Purification by a flash column chromatography on silica gel (hexane/EtOAc = 1/1) afforded **3pa** as a pale yellow solid (83.1 mg, 0.37 mmol, 93%). **^{1}H NMR** (400 MHz, $CDCl_3$, rt, δ/ppm): 7.84–7.81 (m, 2H, Ar-*H*), 7.39–7.28 (m, 5H, Ar-*H*), 6.62–6.58 (m, 2H, Ar-*H*), 4.58 (brs, 1H, N*H*), 4.41 (d, *J* = 5.6 Hz, 2H, CH_2), 2.49 (s, 3H, CH_3).

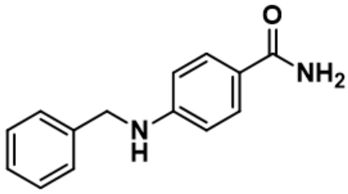

4-(Benzylamino)benzamide (3qa):[9] The general procedure was followed with **2a** (42.4 mg, 0.40 mmol), **1q** (54.5 mg, 0.40 mmol), **B**[4b] (14.5 mg, 0.02 mmol), and H_2 (20 atm). Purification by a flash column chromatography on silica gel (EtOAc) afforded **3qa** as a white solid (86.7 mg, 0.38 mmol, 95%). **^{1}H NMR** (400 MHz, DMSO-d_6, rt, δ/ppm): 7.59 (d, *J* = 8.8 Hz, 2H, Ar-*H*), 7.51 (brs, 1H, one of NH_2), 7.35–7.30 (m, 4H, Ar-*H*), 7.25–7.21 (m, 1H, Ar-*H*), 6.83 (brs, 1H, other peak of NH_2), 6.78 (t, *J* = 6.0 Hz, 1H, N*H*), 6.55 (d, *J* = 8.8 Hz, 2H, Ar-*H*), 4.31 (d, *J* = 6.0 Hz, 2H, CH_2). Resonances of N*H* were not identified.

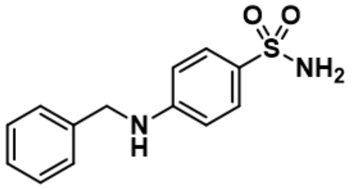

4-(Benzylamino)benzenesulfonamide (3ra):[9] The general procedure was followed with **2a** (42.4 mg, 0.40 mmol), **1r** (68.9 mg, 0.40 mmol), **B**[4b] (14.5 mg, 0.02 mmol), and H_2 (20 atm). Purification by a flash column chromatography on silica gel (*n*-hexane only) afforded **3ra** as a pale yellow solid (103.1 mg, 0.39 mmol, 98%). **^{1}H NMR** (400 MHz, DMSO-d_6, rt, δ/ppm): 7.47 (d, J = 8.8 Hz, 2H, Ar-*H*), 7.33–7.32 (m, 4H, Ar-*H*), 7.26–7.21 (m, 1H, N*H*), 6.99 (t, J = 6.0 Hz, 1H, Ar-*H*), 6.88 (brs, 2H, NH_2), 6.62 (d, J = 8.8 Hz, 2H, Ar-*H*). 4.33 (d, J = 6.0 Hz, 2H, CH_2).

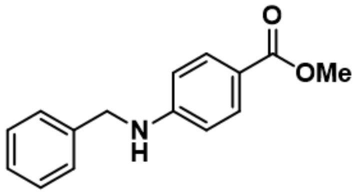

Methyl 4-(benzylamino)benzoate (3sa):[9] The general procedure was followed with **2a** (42.4 mg, 0.40 mmol), **1s** (60.5 mg, 0.40 mmol), **B**[4b] (14.5 mg, 0.02 mmol), and H_2 (20 atm). Purification by a flash column chromatography on silica gel (*n*-hexane only) afforded **3sa** as a white solid (92.2 mg, 0.38 mmol, 95%). **^{1}H NMR** (400 MHz, $CDCl_3$, rt, δ/ppm): 7.87–7.84 (m, 2H, Ar-*H*), 7.38–7.27 (m, 5H, Ar-*H*), 6.61–6.57 (m, 2H, Ar-*H*), 4.49 (brs, 1H, N*H*), 4.39 (d, J = 5.2 Hz, 2H, CH_2), 3.85 (s, 3H, COOCH_3).

[9] Monitoring the formation of 3aa

A MTHP solution of **1a** (137.1 mg, 1.0 mmol, [**1a**] = 0.050 M), **2a** (106.1 mg, 1.0 mmol, [**2a**] = 0.050 M), **B**^{4b} (36.2 mg, 0.050 mmol), was prepared. Then, this reaction mixture was equally divided into 10 autoclave reactors (V = 10 mL), and 4 Å molecular sieves (25 mg) were added to each solution, affording a 2.0 mL of MTHP solution ([**1a**] = [**2a**] = 0.050 M). Once sealed, each vessel was pressurized with H_2 (40 atm) and the reaction mixture was stirred at 100 °C. After heating for the certain reaction period, a resultant crude mixture was filtered to remove MS, dried in vacuo. The yield of **3aa** was calculated by ^{1}H NMR analyses using 1,3,5-trimethoxybenzene as an internal standard. The obtained time-concentration profile was shown in Supplementary Figure 11, showing obvious similarity with the **B**^{1a}-catalyzed reductive alkylation of amines with aldehydes.[9] We thus calculated TOFs used in GPR analysis based on the following equation:

$$\text{TOF (h}^{-1}\text{) = yield of } \mathbf{3aa} \text{ (\%)/ reaction time (h)}$$

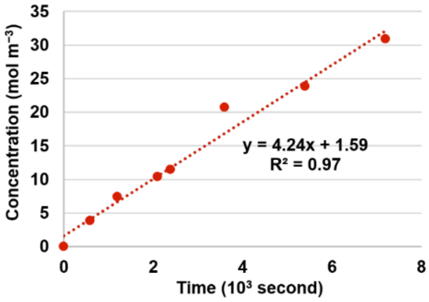

Supplementary Figure 11. Concentration vs. time profiles of the production of **3aa**

[10] Gaussian process regression for Model I to V

The code and .csv folder for all theoretical values used in this work can be found in the Zenode repository, see DOI: 10.5281/zenodo.8420295.

Supplementary Table 3. ***Q***^{2} **values of Model I – VIII**

Model No.	I	II	III	IV
3D surface	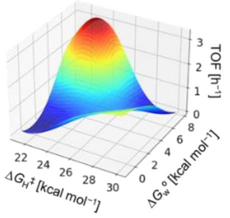	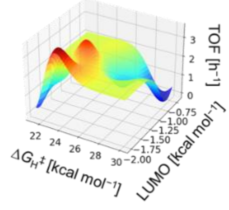	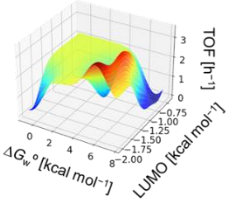	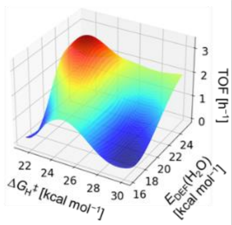
Theoretical value combinations	$\Delta G_H^‡$ vs ΔG_w°	$\Delta G_H^‡$ vs LUMO	ΔGw° vs LUMO	$\Delta G_H^‡$ vs $E_{DEF}(H_2O)$
Q^2 (Leave one out)	0.78	0.23	0.23	0.59

Model No.	V	VI	VII	VIII
3D surface	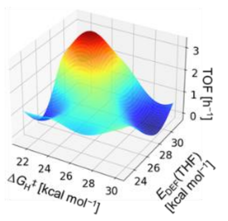	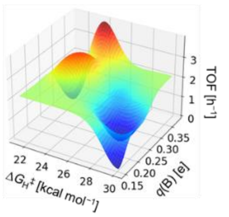	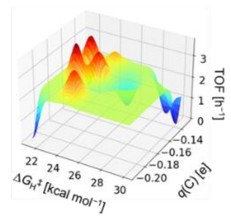	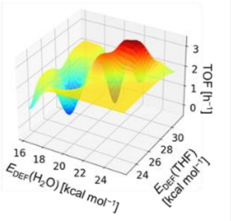
Theoretical value combinations	$\Delta G_H^‡$ vs E_{DEF}(THF)	$\Delta G_H^‡$ vs q(B)	$\Delta G_H^‡$ vs q(C)	$E_{DEF}(H_2O)$ vs E_{DEF}(THF)
Q^2 (Leave one out)	0.51	0.09	-0.31	0.16

[11] Theoretical studies

11-1. Computational details

The density functional theory (DFT) calculations were performed with Gaussian 16 (Revision C.01) software.[11] All the structures were optimized at the ωB97X-D[12] level of theory with the 6-31G(d,p) basis sets. Frequency calculations were performed to verify that intermediates have no imaginary frequency, whereas the transition state structures have only one imaginary frequency. For selected transition state structures, the appropriateness of the connections between each reactant and product via the transition state was confirmed using intrinsic reaction coordinate (IRC).[13] Single-point energy calculations were carried out at ωB97X-D/6-311+G(d,p)/gas-phase level of theory otherwise noted. The reported Gibbs free energies were calculated at 298.15 K. These calculations involve a certain margin of error. The coordinates of the optimized structures are given in Source_data.xlsx.

For the calculation of deformation energies (E_{DEF}), the ORCA 5.0.3 program package was applied.[14] Gas phase equilibrium structures for **H_2O−B^{xy}** and **THF–B^{xy}** adducts have been obtained on the PBEh-3c/Def2-SVPS6 level of theory, which was also applied for the frequency analysis to verify that these structures have no imaginary frequency.

E_{DEF} values were obtained by computing the single point energies (RI-DSDPBEP86-D3BJ/ma-Def2-QZVPP) of B^{xy} units in the structures of each **H_2O−B^{xy}** and **THF–B^{xy}** adducts, and subtraction from the energies in the relaxed states.[15]

11-2. Comparison of geometrical parameters in **B^{xy}** and **H_2O−B^{xy}** adducts

From the optimized geometrical parameters of B^{xy} at the PBEh-3c/Def2-SVPS6 level, we obtained the following four indices: (i) q(B) (the Mulliken charge on boron atom in B^{xy}); (ii) q(C) (the averaged value of the Mulliken charge on *ipso*-carbon atoms; (iii) Mayer's bonded valence on B; and (iv) the averaged value of the Mayer's bonded valence of *ipso*-carbon atoms. These results are summarized in Supplementary Table 3.

Supplementary Table 3. Comparison of theoretical parameters in $\mathbf{B}^{xy}$

B^{xy}	B^{1a}	B^{1b}	B^{1c}	B^{1d}	B^{1e}	B^{1f}	B^{1g}	B^{1h}	B^{1i}	B^{1v}
**q(B)**	**0.3118**	**0.2793**	**0.2539**	**0.2739**	**0.2765**	**0.3077**	**0.2685**	**0.2751**	**0.2724**	**0.15**
**q(C)**	**-0.166**	**-0.147**	**-0.125**	**-0.143**	**-0.147**	**-0.166**	**-0.149**	**-0.162**	**-0.139**	**-0.137**
**Mayer bond order on B**	**3.218**	**3.305**	**3.328**	**3.307**	**3.296**	**3.232**	**3.313**	**3.307**	**3.298**	**3.42**
**Mayer bond order on ipso-C**	**3.767**	**3.774**	**3.753**	**3.768**	**3.767**	**3.771**	**3.775**	**3.786**	**3.758**	**3.742**

B^{xy}	B^{1w}	B^{2a}	B^{2b}	B^{2c}	B^{2d}	B^{2e}	B^{2f}	B^{2g}	B^{2h}	B^{2i}
**q(B)**	**0.252**	**0.265**	**0.2975**	**0.3326**	**0.2163**	**0.2691**	**0.2843**	**0.2607**	**0.2875**	**0.2645**
**q(C)**	**-0.157**	**-0.14**	**-0.194**	**-0.202**	**-0.139**	**-0.172**	**-0.184**	**-0.175**	**-0.203**	**-0.172**
**Mayer bond order on B**	**3.368**	**3.279**	**3.237**	**3.159**	**3.398**	**3.301**	**3.277**	**3.318**	**3.268**	**3.302**
**Mayer bond order on ipso-C**	**3.754**	**3.736**	**3.765**	**3.758**	**3.757**	**3.76**	**3.749**	**3.769**	**3.779**	**3.755**

B^{xy}	B^{2j}	B^{2k}	B^{2l}	B^{2m}	B^{2n}	B^{2o}	B^{2p}	B^{2q}	B^{2r}	B^{2t}
**q(B)**	**0.2252**	**0.2825**	**0.2312**	**0.2163**	**0.2484**	**0.2607**	**0.2448**	**0.2842**	**0.2488**	**0.2465**
**q(C)**	**-0.141**	**-0.191**	**-0.161**	**-0.146**	**-0.185**	**-0.183**	**-0.173**	**-0.208**	**-0.172**	**-0.141**
**Mayer bond order on B**	**3.352**	**3.257**	**3.348**	**3.399**	**3.359**	**3.313**	**3.341**	**3.26**	**3.33**	**3.346**
**Mayer bond order on ipso-C**	**3.741**	**3.772**	**3.756**	**3.787**	**3.759**	**3.758**	**3.771**	**3.777**	**3.764**	**3.793**

B^{xy}	B^{2u}	B^{3a}	B^{3b}	B^{3c}	B^{3d}	B^{3e}	B^{3f}	B^{3g}	B^{3h}	B^{3i}
**q(B)**	**0.2465**	**0.3179**	**0.2843**	**0.2829**	**0.2469**	**0.2542**	**0.3673**	**0.2596**	**0.2662**	**0.2681**
**q(C)**	**-0.157**	**-0.157**	**-0.17**	**-0.159**	**-0.137**	**-0.156**	**-0.189**	**-0.158**	**-0.171**	**-0.17**
**Mayer bond order on B**	**3.345**	**3.169**	**3.331**	**3.248**	**3.327**	**3.34**	**3.23**	**3.319**	**3.313**	**3.289**
**Mayer bond order on ipso-C**	**3.883**	**3.747**	**3.757**	**3.743**	**3.762**	**3.768**	**3.715**	**3.774**	**3.786**	**3.763**

B^{xy}	B^{3s}	B^{4a}	B^{4b}	B^{4c}	B^{4d}	B^{4e}	B^{4f}	B^{4g}	B^{4h}	B^{4i}
**q(B)**	**0.2569**	**0.2859**	**0.2659**	**0.2618**	**0.2648**	**0.3718**	**0.2772**	**0.2564**	**0.2873**	**0.2537**
**q(C)**	**-0.146**	**-0.167**	**-0.157**	**-0.149**	**-0.147**	**-0.17**	**-0.159**	**-0.168**	**-0.165**	**-0.145**
**Mayer bond order on B**	**3.31**	**3.241**	**3.304**	**3.307**	**3.305**	**3.245**	**3.28**	**3.306**	**3.244**	**3.322**
**Mayer bond order on ipso-C**	**3.766**	**3.768**	**3.772**	**3.755**	**3.766**	**3.715**	**3.761**	**3.776**	**3.779**	**3.765**

B^{xy}	B^{4j}	B^{5a}	B^{6a}	BCF
**q(B)**	**0.2208**	**0.2443**	**0.258**	**0.3749**
**q(C)**	**-0.119**	**-0.13**	**-0.147**	**-0.213**
**Mayer bond order on B**	**3.351**	**3.317**	**3.306**	**3.184**
**Mayer bond order on ipso-C**	**3.751**	**3.757**	**3.756**	**3.655**

From the optimized geometrical parameters of H_2O−B^{xy} adducts, we obtained the following two indices for the evaluation of the degree of geometrical deviation from the ideal tetrahedral geometry around the boron center: (i) $\tau_\delta(B) = \{360 - (\alpha + \beta)/141 \times \beta/\alpha\}$, where α and β are the largest and second largest C−B−C angles;[16] (ii) a tetrahedral character (THC),[17] calculated by the following equation (θ = X−B−C, X = O or C; n = 1 − 6):

$$\mathrm{THC} = \left[1 - \frac{\sum_{n=1-6}|109.5-\theta_n|}{27}\right] \times 100$$

When the boron center adopts ideal tetrahedral geometry, both $\tau_\delta(B)$ and THC provide each 1.0 and 100 as their maximum, and smaller values thus suggest that the boron atom adopts more distorted tetrahedral geometry. Results are summarized in Supplementary Table 4.

Supplementary Table 4. Comparison of geometrical parameters in $H_2O{-}B^{xy}$ adducts

B^{xy}	B^{1a}	B^{1b}	B^{1c}	B^{1d}	B^{1e}	B^{1f}	B^{1g}	B^{1h}	B^{1i}	B^{1v}
τ_δ(B)	0.88	0.87	0.87	0.86	0.87	0.86	0.9	0.86	0.88	0.9
O−B	1.63	1.65	1.65	1.64	1.63	1.64	1.65	1.64	1.64	1.67
av. B−C_{Ar}	1.63	1.63	1.63	1.63	1.63	1.63	1.63	1.63	1.63	1.63
THC (%)	91.6	88.7	88.6	90.1	91.5	90.2	90.8	90.1	90.2	87.6

B^{xy}	B^{1w}	B^{2a}	B^{2b}	B^{2c}	B^{2d}	B^{2e}	B^{2f}	B^{2g}	B^{2h}	B^{2i}
τ_δ(B)	0.87	0.88	0.87	0.87	0.87	0.87	0.88	0.89	0.87	0.87
O−B	1.69	1.64	1.65	1.65	1.65	1.64	1.64	1.65	1.64	1.63
av. B−C_{Ar}	1.63	1.63	1.63	1.63	1.63	1.63	1.63	1.63	1.63	1.63
THC (%)	83.5	90.1	90.1	90	90	90.7	89.8	89.8	90.7	89.7

B^{xy}	B^{2j}	B^{2k}	B^{2l}	B^{2m}	B^{2n}	B^{2o}	B^{2p}	B^{2q}	B^{2r}	B^{2t}
τ_δ(B)	0.88	0.88	0.89	0.88	0.9	0.89	0.89	0.87	0.87	0.89
O−B	1.65	1.65	1.65	1.65	1.66	1.66	1.65	1.64	1.65	1.64
av. B−C_{Ar}	1.63	1.64	1.63	1.63	1.63	1.63	1.63	1.63	1.63	1.63
THC (%)	89.2	89	88.6	89.3	87.8	87.4	89.1	89.5	89.8	89.9

B^{xy}	B^{2u}	B^{3a}	B^{3b}	B^{3c}	B^{3d}	B^{3e}	B^{3f}	B^{3g}	B^{3h}	B^{3i}
τ_δ(B)	0.89	0.89	0.88	0.88	0.88	0.88	0.87	0.88	0.89	0.87
O−B	1.65	1.65	1.65	1.65	1.65	1.65	1.65	1.65	1.64	1.64
av. B−C_{Ar}	1.63	1.63	1.63	1.63	1.63	1.63	1.63	1.63	1.63	1.63
THC (%)	89.1	89.8	89.7	89.7	89.6	89.3	89.5	88.6	89.7	90.6

B^{xy}	B^{3s}	B^{4a}	B^{4b}	B^{4c}	B^{4d}	B^{4e}	B^{4f}	B^{4g}	B^{4h}	B^{4i}
τ_δ(B)	0.87	0.88	0.87	0.86	0.88	0.88	0.91	0.91	0.87	0.88
O−B	1.65	1.66	1.64	1.64	1.65	1.65	1.65	1.65	1.64	1.64
av. B−C_{Ar}	1.63	1.63	1.63	1.63	1.63	1.63	1.63	1.63	1.63	1.63
THC (%)	89.7	88.8	89.2	91.1	89.8	88.7	88.4	89.3	91.5	90.5

B^{xy}	B^{4j}	B^{5a}	B^{6a}	BCF
τ_δ(B)	0.88	0.88	0.88	0.92
O−B	1.66	1.66	1.65	1.64
av. B−C_{Ar}	1.63	1.62	1.63	1.62
THC (%)	87.3	89.3	90.1	92.5

11-3. Selected frontier molecular orbitals (FMOs) for $\mathbf{B}^{xy}$

FMOs including the p orbitals of the boron, calculated at the ωB97X-D/6-311+G(d,p)//ωB97X-D/6-31G(d,p) level, are shown in Supplementary Figure 12.

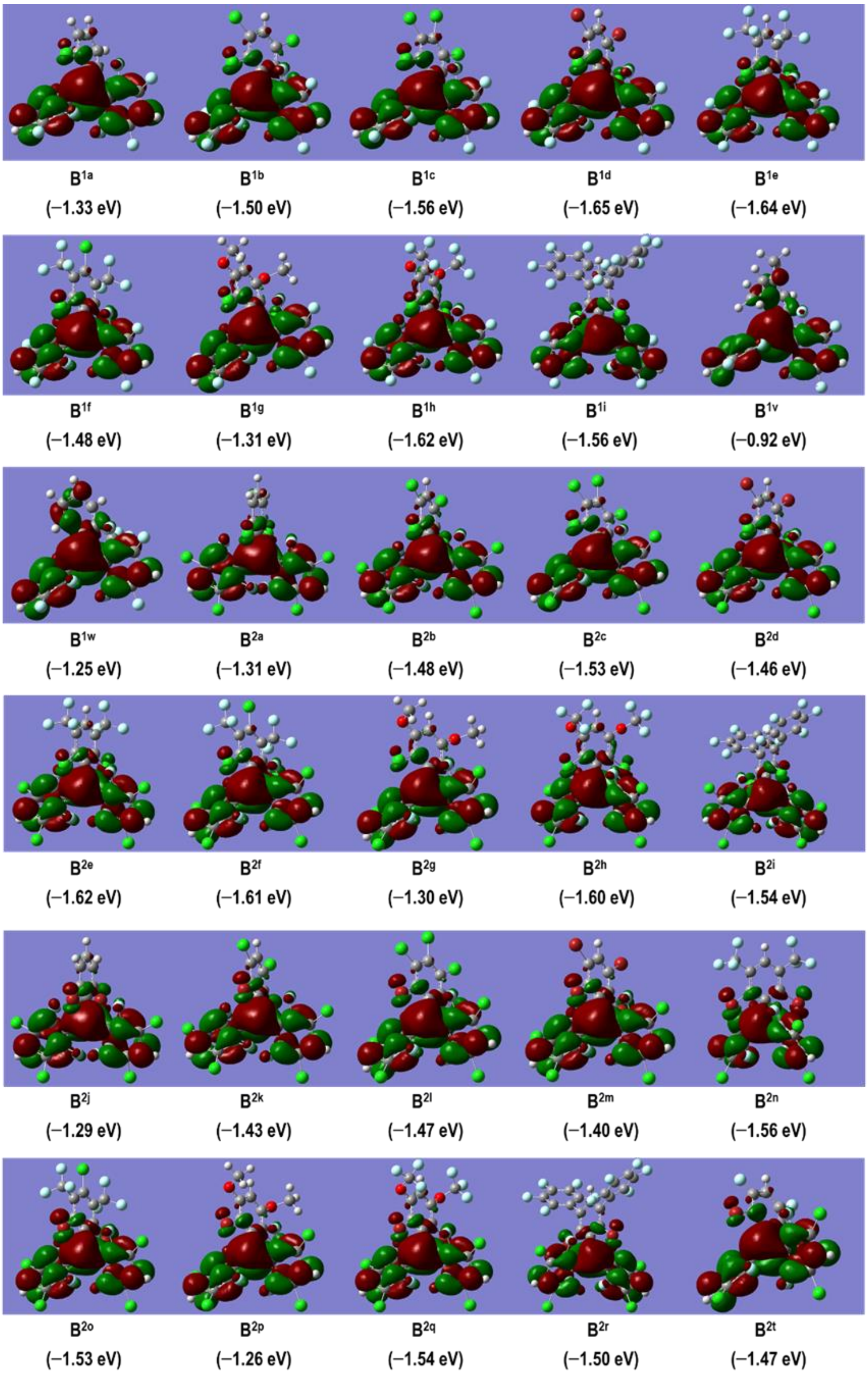

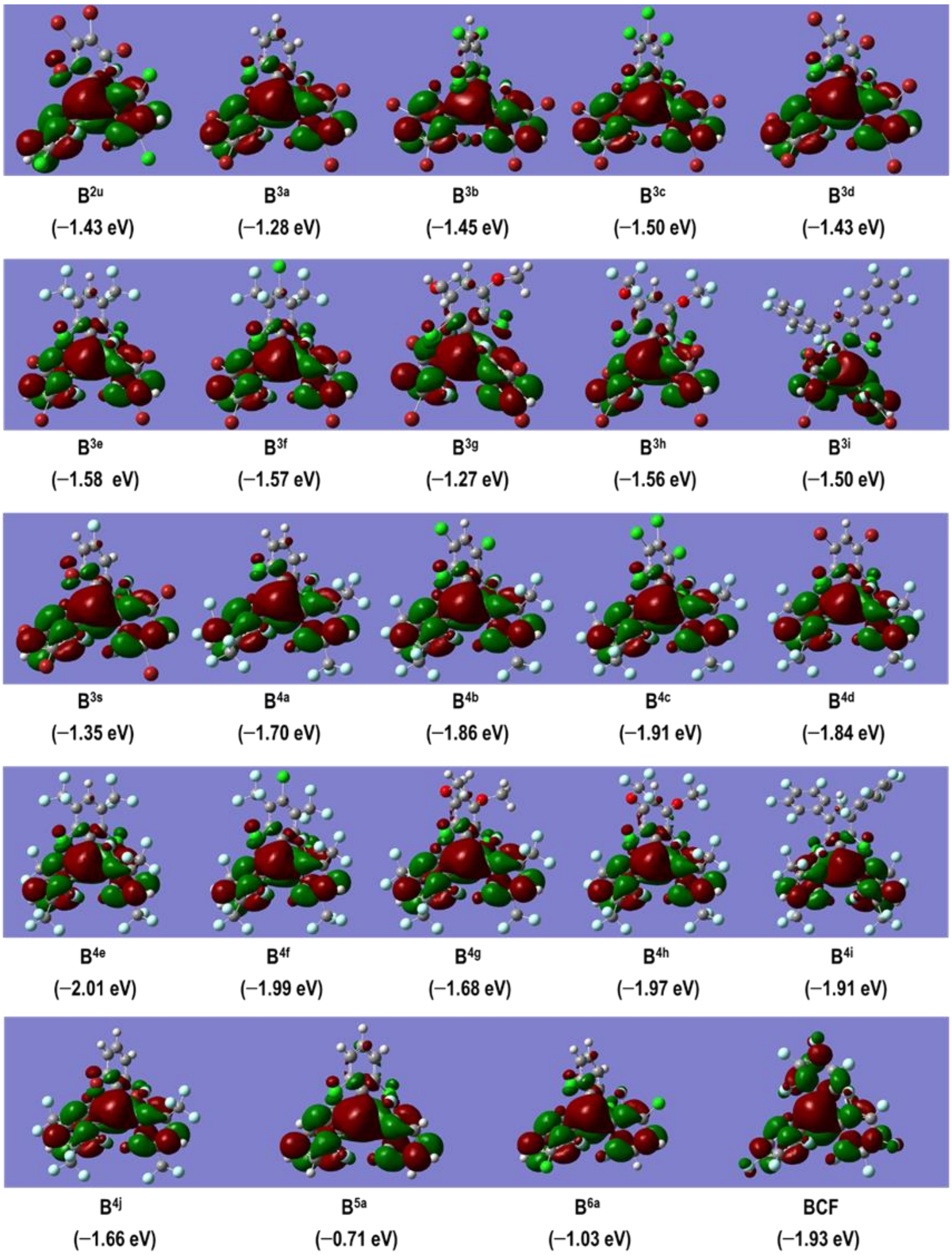

Supplementary Figure 12. Selected FMOs (LUMO in most cases) for $\mathbf{B}^{xy}$

11-4. Results of AIM analysis

The AIM calculation for TS_{MTHP} was carried out using AIMAll program (Version 19.10.12),[18] in which wave functional files were prepared based on the SCF density at the aforementioned level of theory (Supplementary Figure 13 and Supplementary Table 5).

The AIM bond paths (white lines), bond critical points (BCP; $e\ r_{Bohr}^{-3}$; green dots; their numbers are also shown).

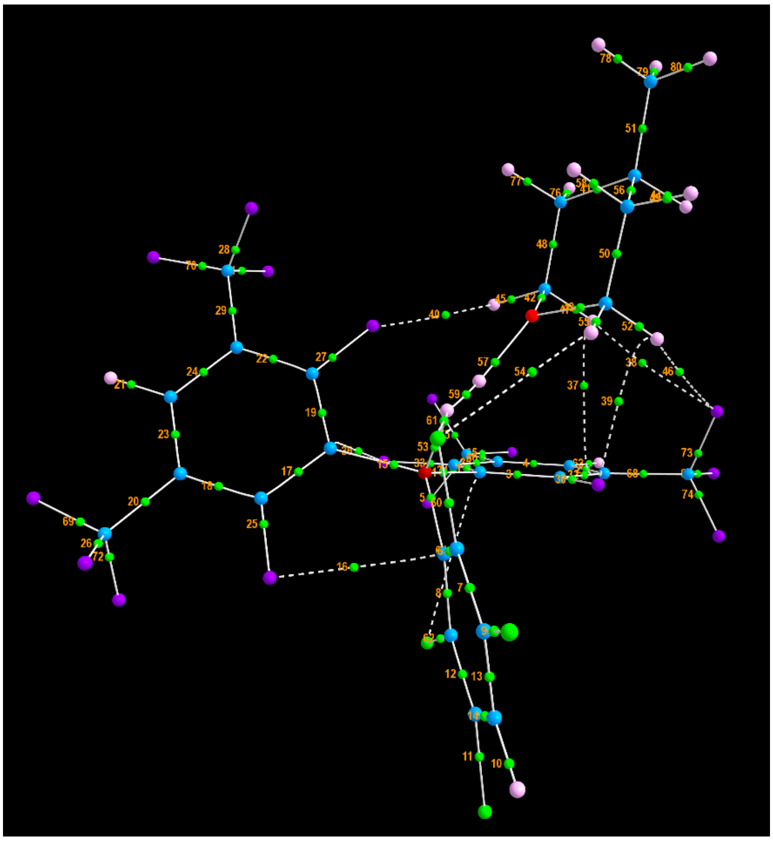

Supplementary Figure 13. Selected AIM results for TS_{MTHP}. NCIs are shown in dashed white lines.

Supplementary Table 5. Selected AIM parameters for TS_{MTHP}

BCP #	*Atoms*	*Rho*	*DelSqRho*	*DI(A|B)*	*G*	*V*
1	C39 - C40	0.31064	-0.872215	1.313351	0.108375	-0.434803
2	C1 - C3	0.308379	-0.851426	1.352646	0.104611	-0.422078
3	C38 - C40	0.311195	-0.873115	1.314894	0.11105	-0.440378
4	C2 - C3	0.309371	-0.8565	1.36354	0.105145	-0.424415
5	C4 - B32	0.158987	-0.131622	0.468655	0.120762	-0.274429
6	C4 - C5	0.299061	-0.791262	1.348826	0.103678	-0.405171
7	C5 - C7	0.306155	-0.834229	1.314593	0.10023	-0.409016
8	C4 - C6	0.29849	-0.790667	1.328525	0.101827	-0.401322
9	C7 - Cl66	0.201781	-0.306528	1.116421	0.067615	-0.211863
10	C9 - H35	0.283391	-0.989694	0.926677	0.034697	-0.316818
11	C8 - Cl67	0.20246	-0.308815	1.118565	0.067846	-0.212896

12	C6 - C8	0.307415	-0.840735	1.319861	0.101085	-0.412354
13	C7 - C9	0.312872	-0.88084	1.354102	0.107073	-0.434356
14	C8 - C9	0.311935	-0.876592	1.345696	0.10574	-0.430628
15	C10 - B32	0.159275	-0.096169	0.466036	0.129069	-0.28218
16	C4 - F16	0.012244	0.048313	0.041486	0.010718	-0.009358
17	C10 - C11	0.310338	-0.868787	1.331531	0.108517	-0.434231
18	C11 - C13	0.311035	-0.875057	1.282576	0.102104	-0.422972
19	C10 - C12	0.311909	-0.87901	1.321812	0.111147	-0.442047
20	C13 - C43	0.267043	-0.69597	0.890759	0.059355	-0.292702
21	C15 - H37	0.286189	-1.01143	0.910493	0.033453	-0.319764
22	C12 - C14	0.314646	-0.893078	1.301642	0.106271	-0.435812
23	C13 - C15	0.310053	-0.859738	1.369829	0.105765	-0.426464
24	C14 - C15	0.308246	-0.851838	1.351468	0.104228	-0.421416
25	C11 - F16	0.26331	0.212207	0.822398	0.406292	-0.759532
26	C43 - F49	0.278238	-0.242389	0.697554	0.329099	-0.718796
27	C12 - F17	0.252922	0.229359	0.801062	0.390441	-0.723543
28	C44 - F45	0.276378	-0.244356	0.694471	0.324696	-0.710481
29	C14 - C44	0.267762	-0.700142	0.892702	0.060055	-0.295145
30	F18 - C38	0.256581	0.208177	0.809046	0.392679	-0.733313
31	B32 - C40	0.156459	-0.078992	0.455046	0.129087	-0.277921
32	C1 - C38	0.312969	-0.884089	1.286575	0.104446	-0.429914
33	F19 - C39	0.263238	0.217947	0.819929	0.4074	-0.760313
34	C41 - F55	0.279748	-0.239787	0.699097	0.332896	-0.725738
35	C2 - C39	0.31178	-0.878491	1.284524	0.102773	-0.425169
36	C10 - F19	0.011967	0.047838	0.040173	0.010441	-0.008923
37	C1 - H23	0.007599	0.023011	0.017925	0.004739	-0.003725
38	H23 - F51	0.00524	0.019774	0.015257	0.004205	-0.003467
39	F18 - H26	0.008384	0.03625	0.018069	0.007745	-0.006428
40	F17 - H24	0.010454	0.044296	0.029192	0.00947	-0.007866
41	C57 - C59	0.241527	-0.545887	0.961434	0.056133	-0.248737
42	O20 - C21	0.235168	-0.238496	0.807561	0.246988	-0.5536
43	O20 - C22	0.2361	-0.246077	0.81482	0.24665	-0.554819
44	C57 - H58	0.273178	-0.904965	0.921413	0.042758	-0.311757
45	C21 - H24	0.287117	-1.010442	0.895638	0.034324	-0.321259
46	H26 - F51	0.00564	0.021329	0.018171	0.004538	-0.003743
47	C21 - H23	0.280653	-0.958771	0.892506	0.03672	-0.313132
48	C21 - C59	0.251465	-0.601381	0.966286	0.05836	-0.267065
49	C25 - H28	0.273152	-0.907715	0.938973	0.042059	-0.311046

50	C22 - C25	0.251396	-0.601236	0.967259	0.058082	-0.266472
51	C57 - C62	0.243315	-0.557879	0.984019	0.056237	-0.251943
52	C22 - H26	0.282229	-0.969869	0.893733	0.035863	-0.314193
53	H30 - B32	0.103927	0.16877	0.257519	0.12025	-0.198308
54	H27 - Cl33	0.005414	0.016203	0.03026	0.003268	-0.002485
55	C22 - H27	0.284563	-0.993078	0.905021	0.034726	-0.317723
56	C25 - C57	0.241359	-0.544957	0.961003	0.056128	-0.248496
57	O20 - H31	0.101652	0.101265	0.23731	0.068999	-0.112682
58	C25 - H29	0.273255	-0.906542	0.932077	0.04207	-0.310775
59	H30 - H31	0.182147	-0.548597	0.425402	0.024519	-0.186187
60	C5 - Cl33	0.194556	-0.281643	1.083614	0.065706	-0.201822
61	H30 - Cl33	0.019899	0.066945	0.091872	0.014681	-0.012627
62	C6 - Cl34	0.199058	-0.295167	1.095549	0.067247	-0.208286
63	C3 - H36	0.286053	-1.01027	0.910389	0.033474	-0.319516
64	Cl34 - C40	0.013628	0.046576	0.071868	0.009987	-0.008329
65	C41 - F54	0.275133	-0.222841	0.690078	0.328037	-0.711784
66	C2 - C41	0.267081	-0.695885	0.889959	0.059224	-0.292419
67	C42 - F52	0.277385	-0.21626	0.693527	0.33436	-0.722786
68	C1 - C42	0.268142	-0.701741	0.895113	0.060773	-0.296982
69	C43 - F50	0.274885	-0.222863	0.689287	0.327546	-0.710809
70	C44 - F47	0.276263	-0.219748	0.691933	0.33118	-0.717297
71	C44 - F46	0.27795	-0.244301	0.696846	0.328001	-0.717077
72	C43 - F48	0.278913	-0.242029	0.698318	0.330603	-0.721713
73	C42 - F51	0.269689	-0.261432	0.685694	0.306104	-0.677566
74	C42 - F53	0.281379	-0.23396	0.70221	0.337794	-0.734078
75	C41 - F56	0.277855	-0.244844	0.696376	0.327676	-0.716564
76	C59 - H61	0.273087	-0.90734	0.938758	0.042068	-0.310971
77	C59 - H60	0.273554	-0.90861	0.93041	0.041646	-0.310445
78	C62 - H64	0.271525	-0.896548	0.953923	0.04304	-0.310216
79	C62 - H65	0.272522	-0.90359	0.95837	0.042882	-0.311662
80	C62 - H63	0.272474	-0.903232	0.95853	0.042915	-0.311637

[12] NMR spectra

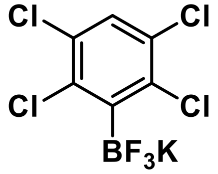

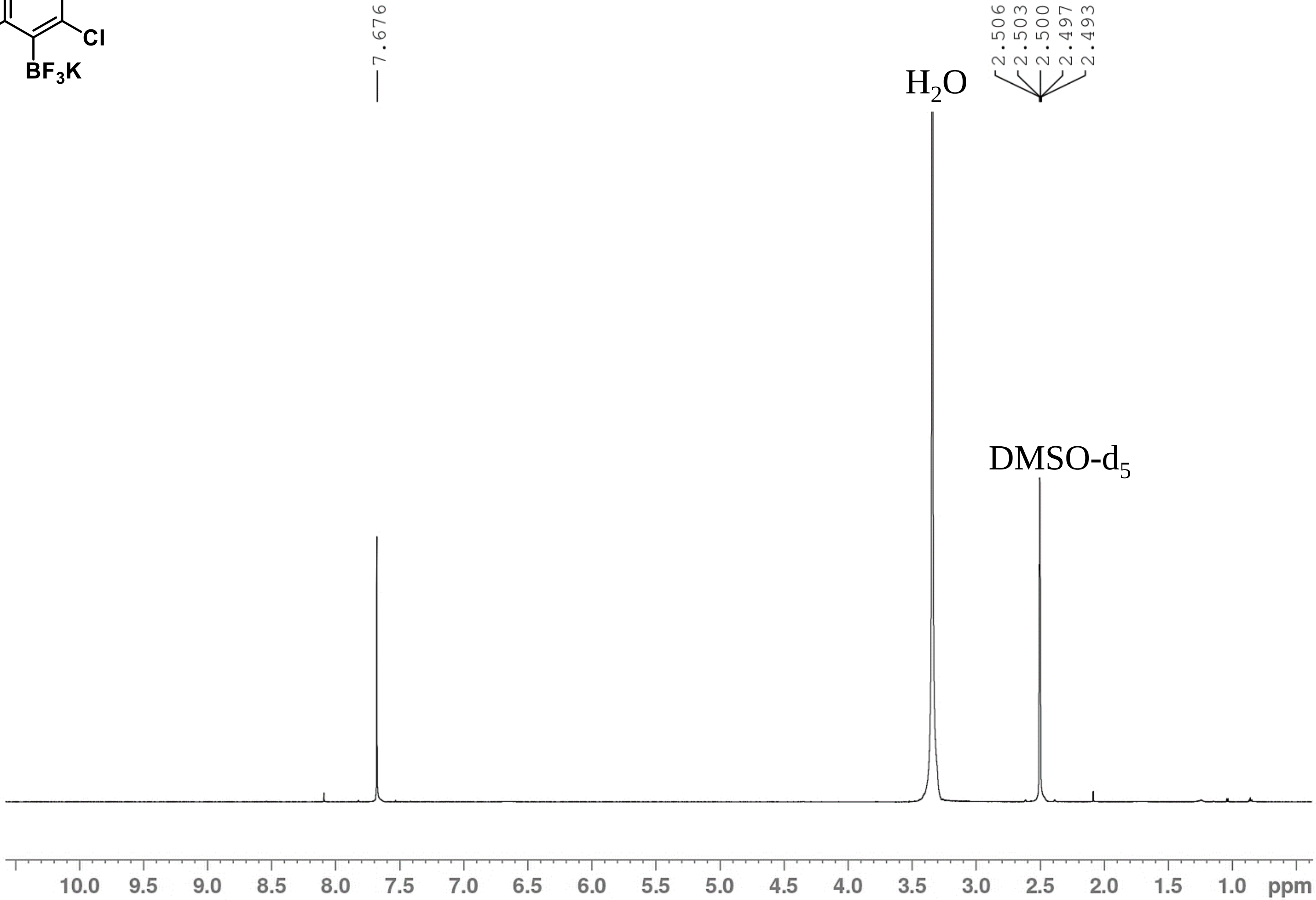

^{11}B, DMSO-d_6 (128 MHz)

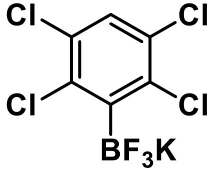

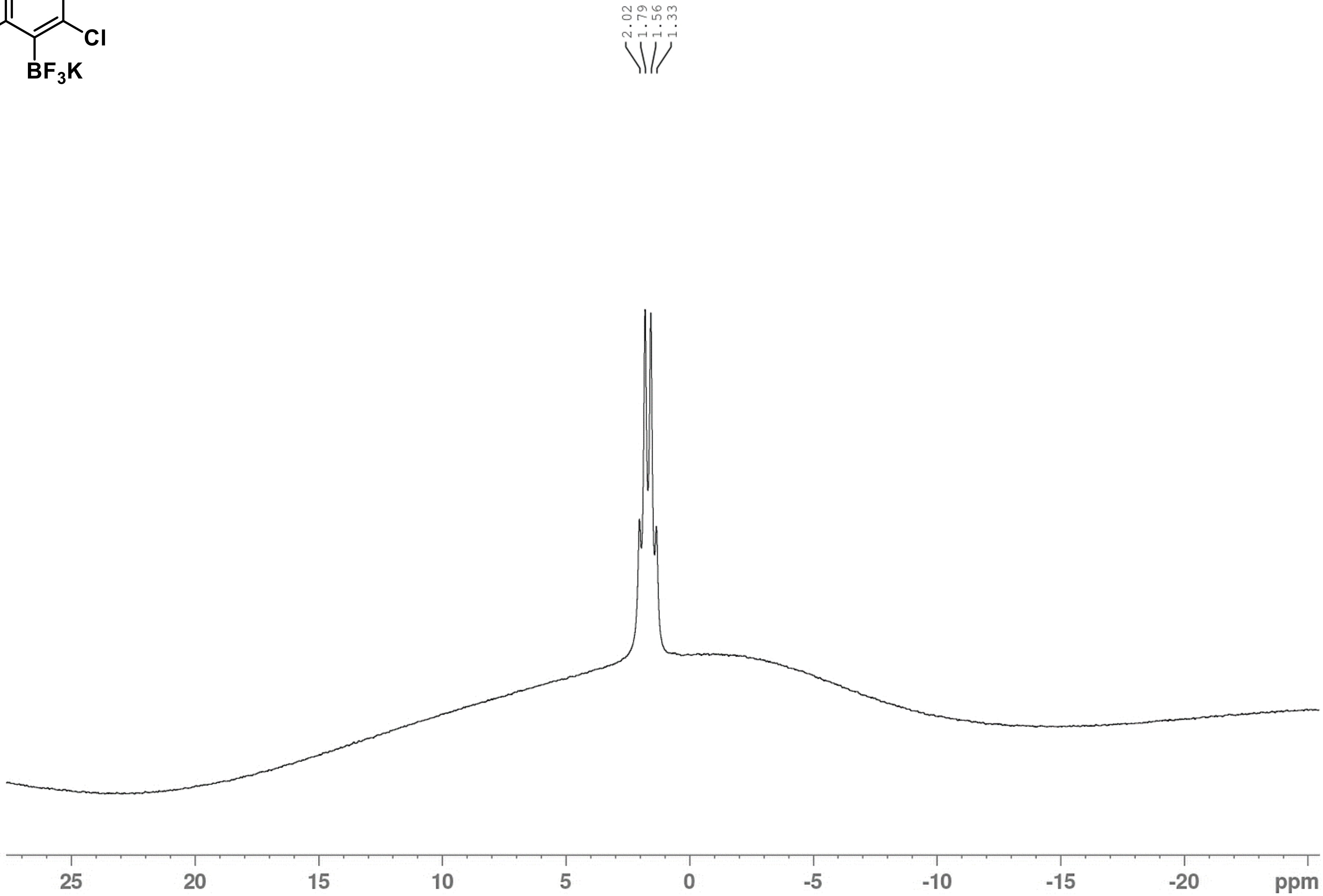

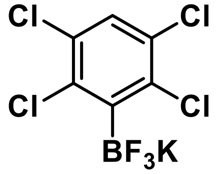

^{13}C, DMSO-d_6 (100 MHz)

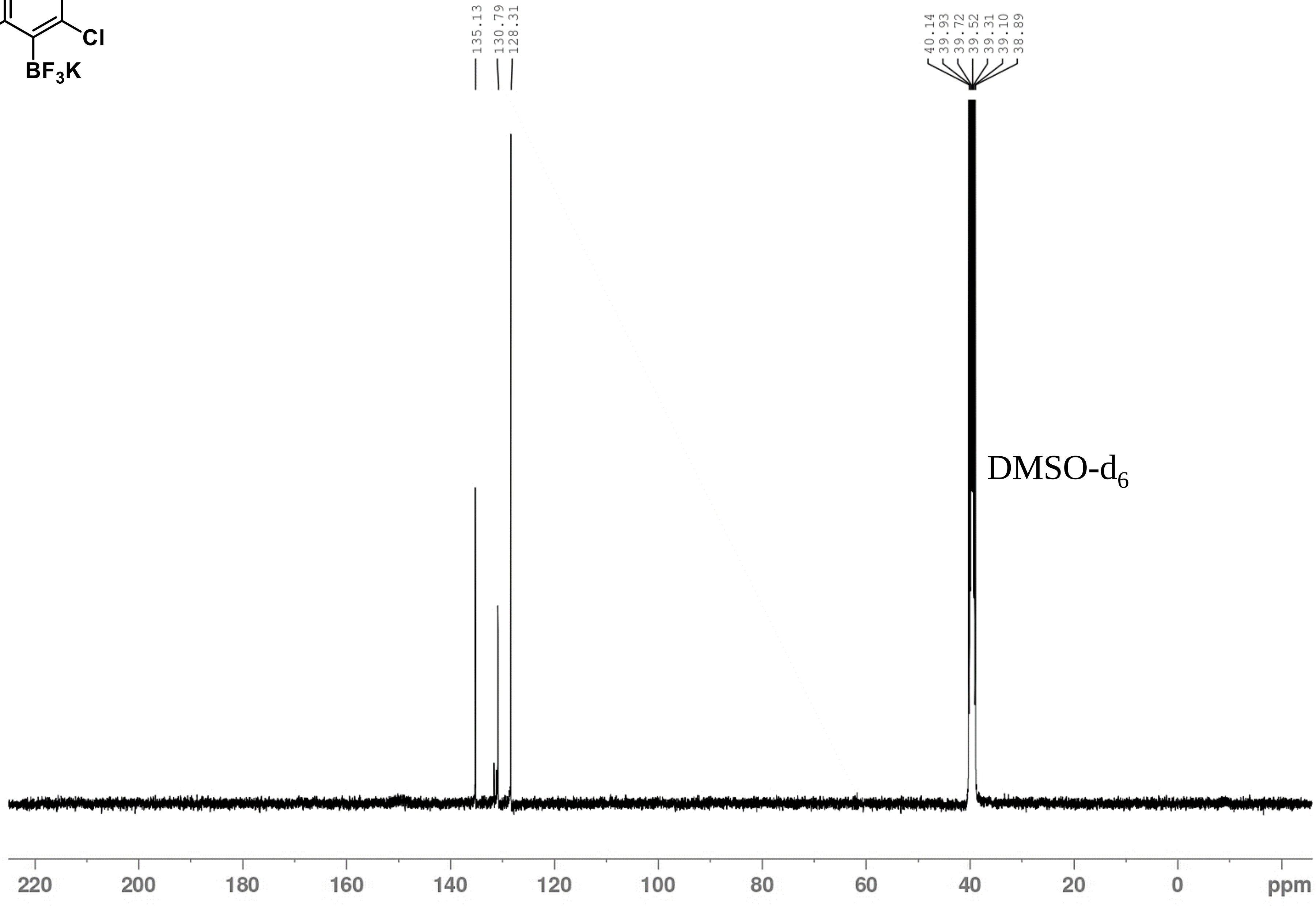

^{19}F, DMSO-d_6 (376 MHz)

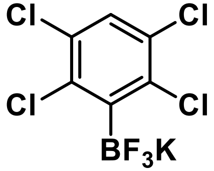

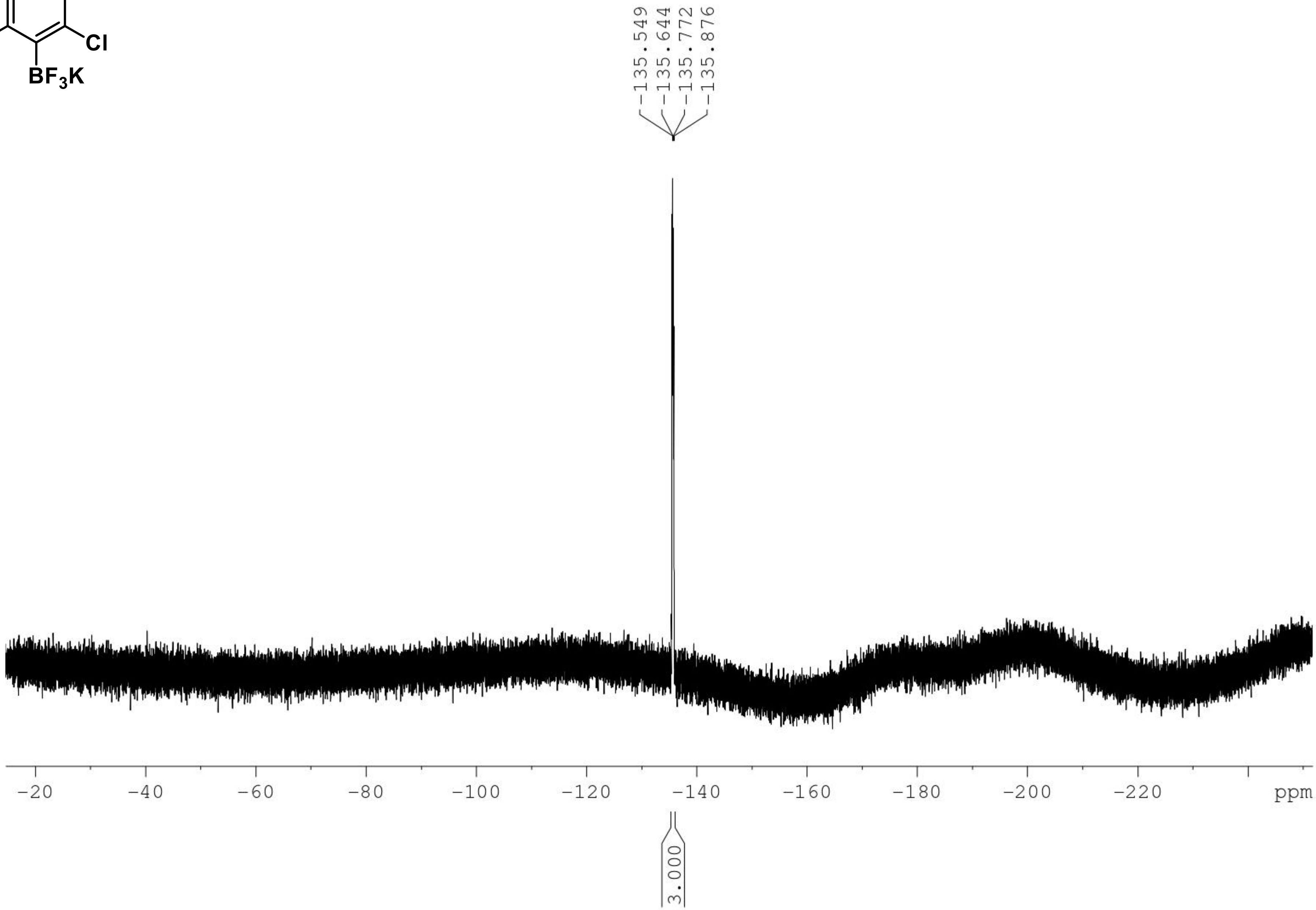

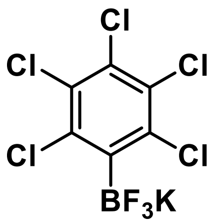

^{11}B, DMSO-d_6 (128 MHz)

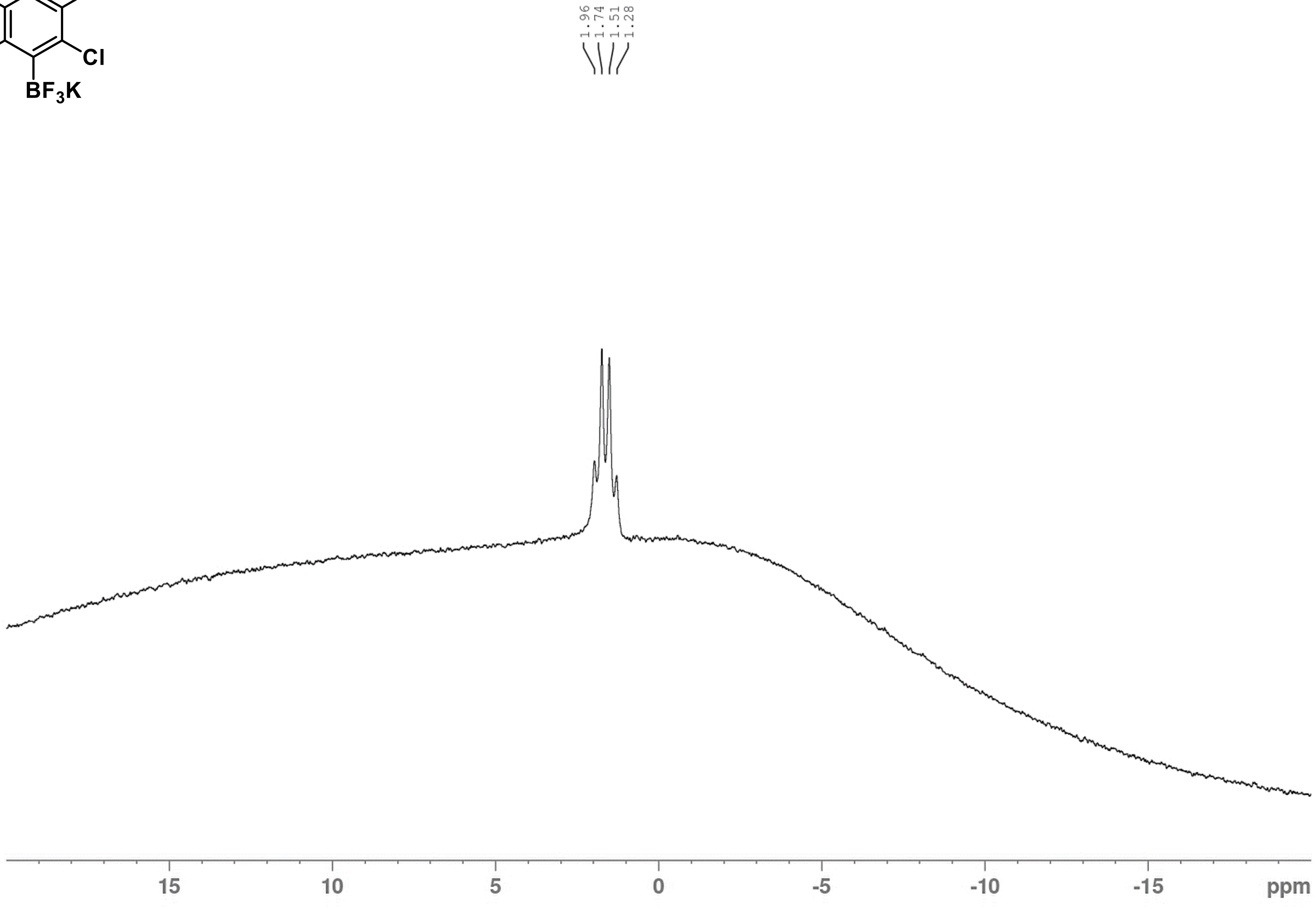

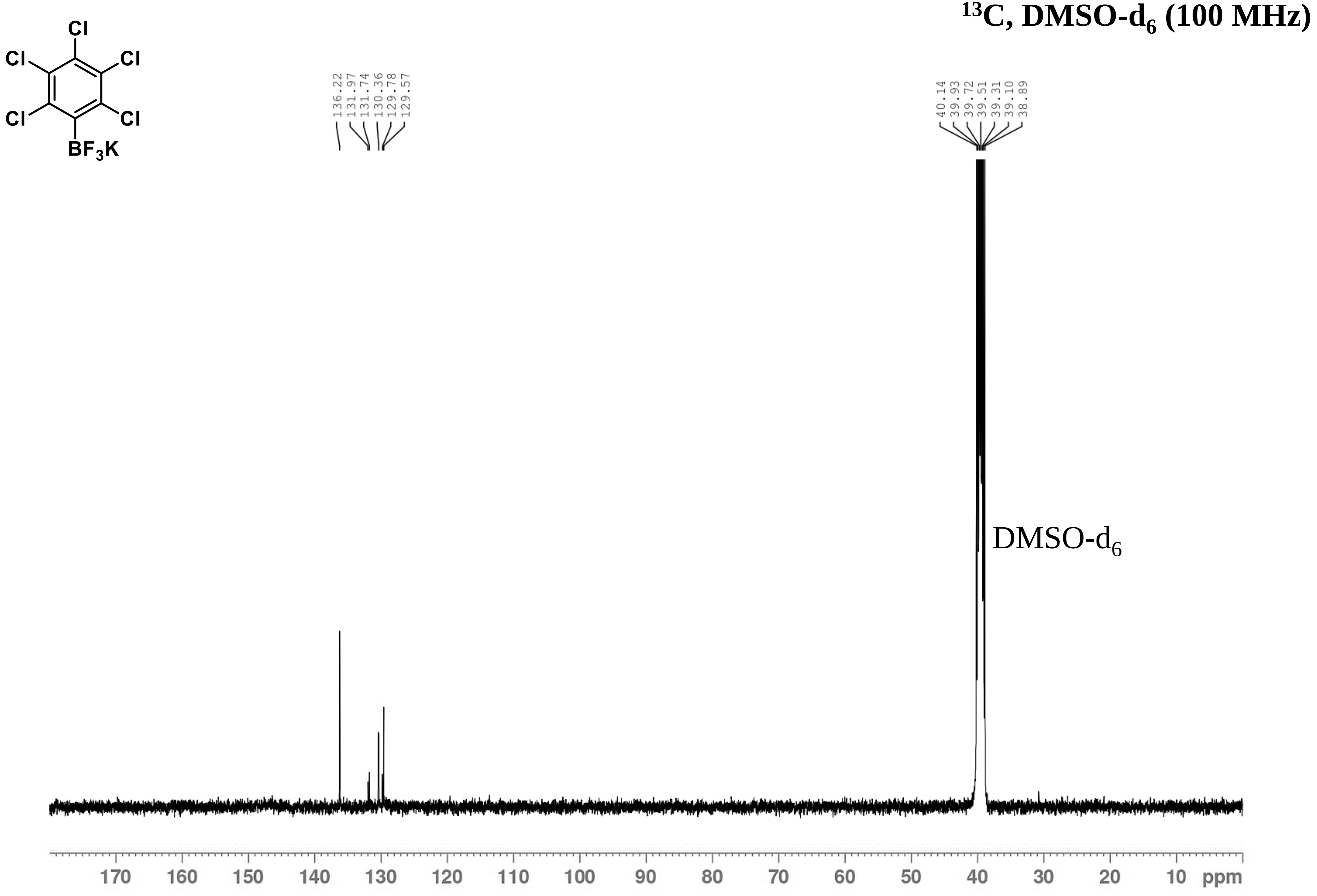
Cl
Cl
Cl
Cl
Cl
BF3K
13C, DMSO-d6 (100 MHz)
136.22
131.97
131.74
130.36
129.78
129.57
40.14
39.93
39.72
39.51
39.31
39.10
38.89
DMSO-d6
170
160
150
140
130
120
110
100
90
80
70
60
50
40
30
20
10
ppm

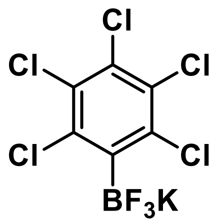

^{19}F, DMSO-d_6 (376 MHz)

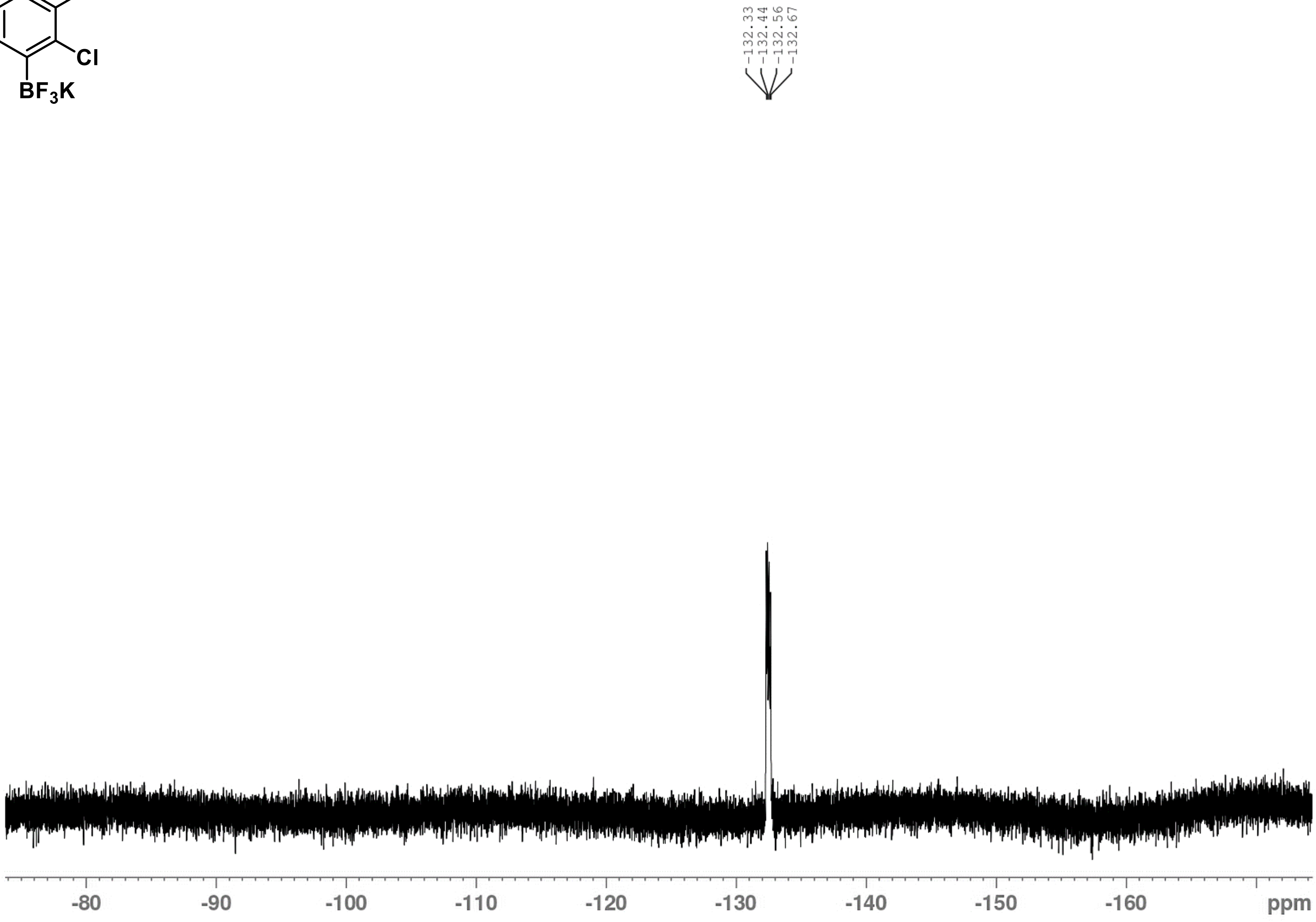

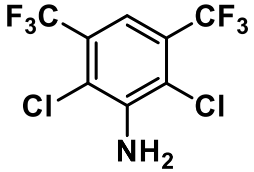

^{1}H, $CDCl_3$ (400 MHz)

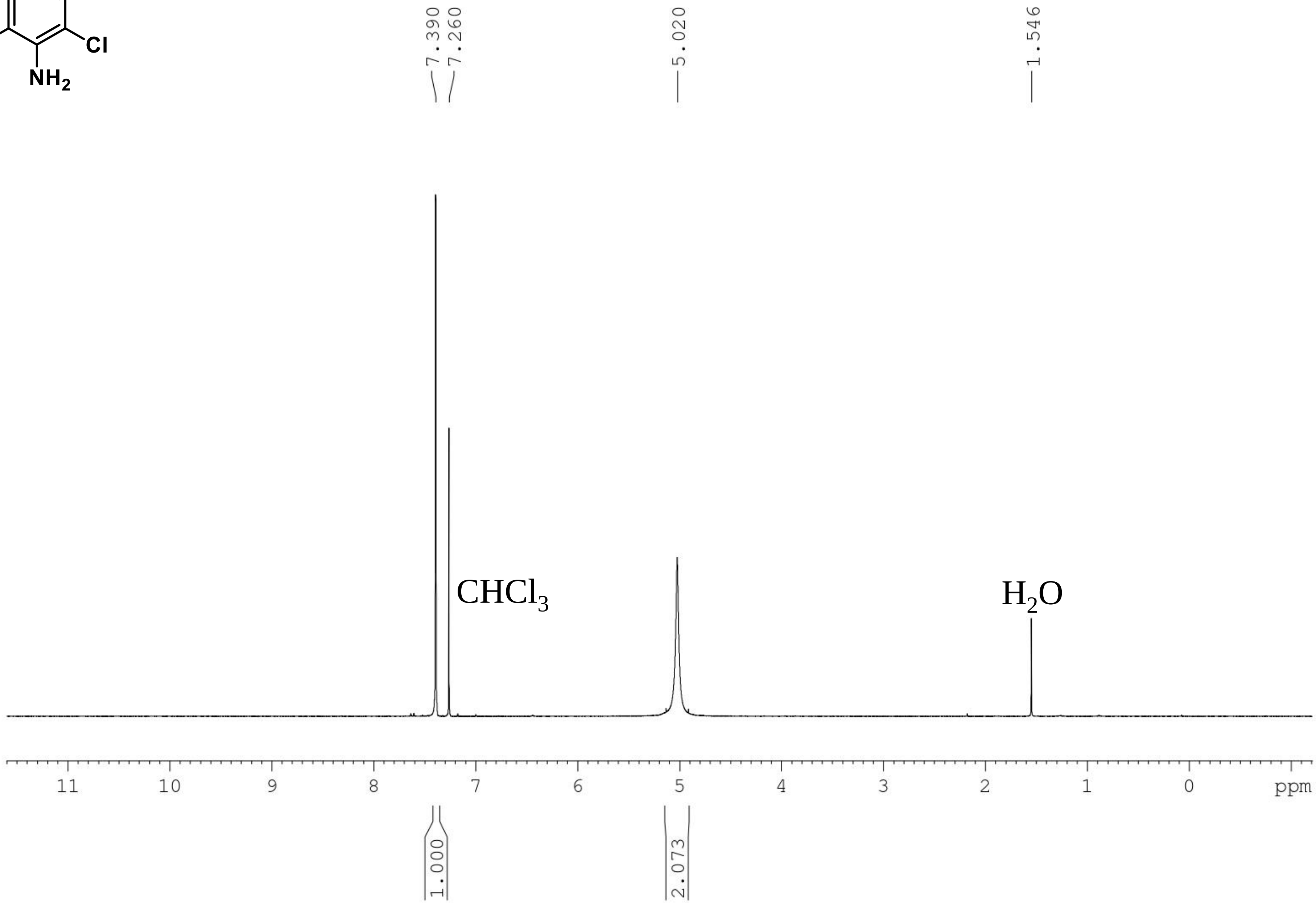

^{13}C, $CDCl_3$ (100 MHz)

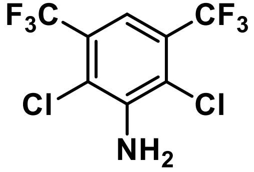

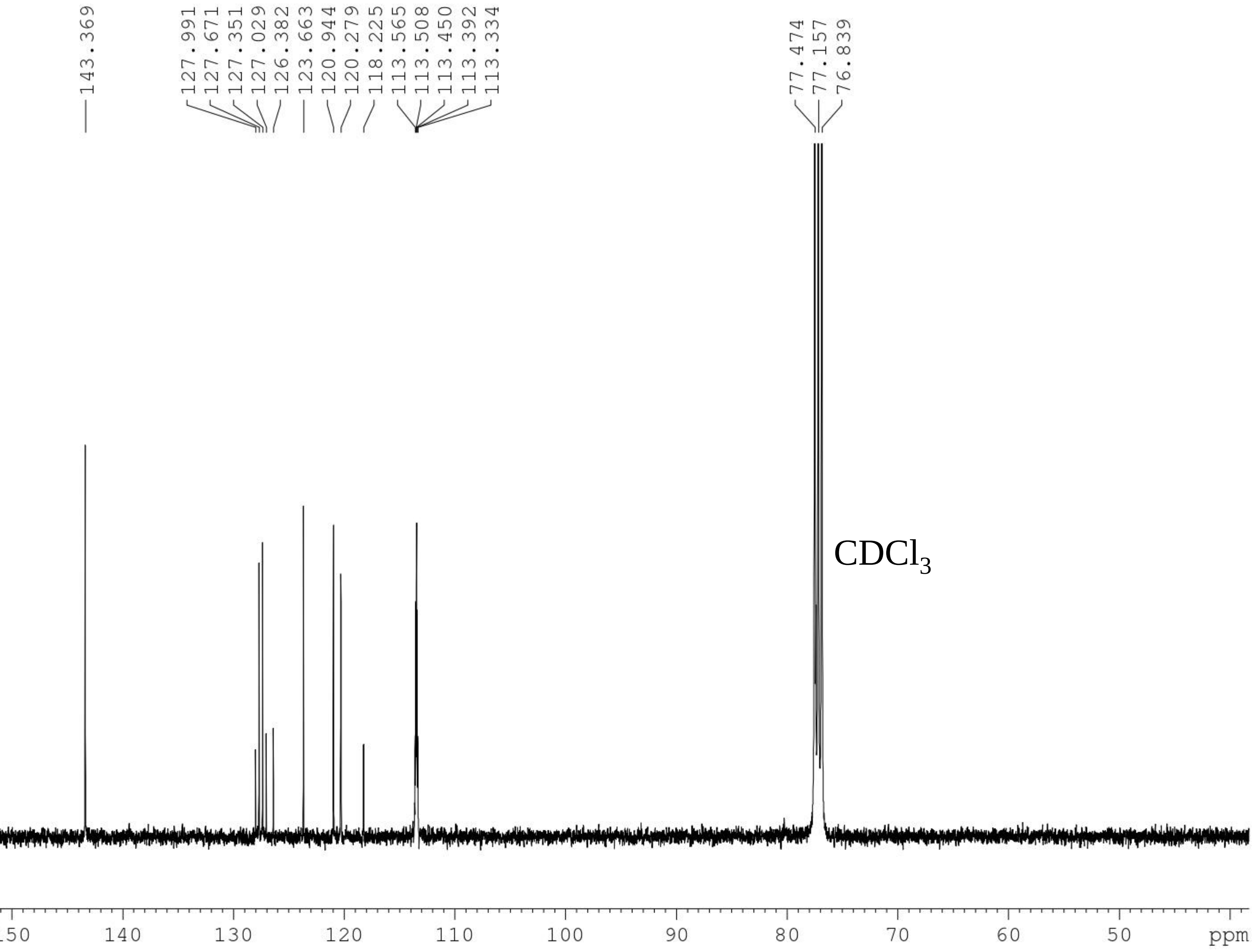

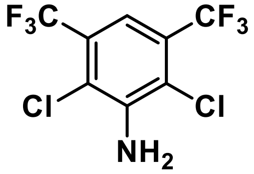

^{19}F, $CDCl_3$ (376 MHz)

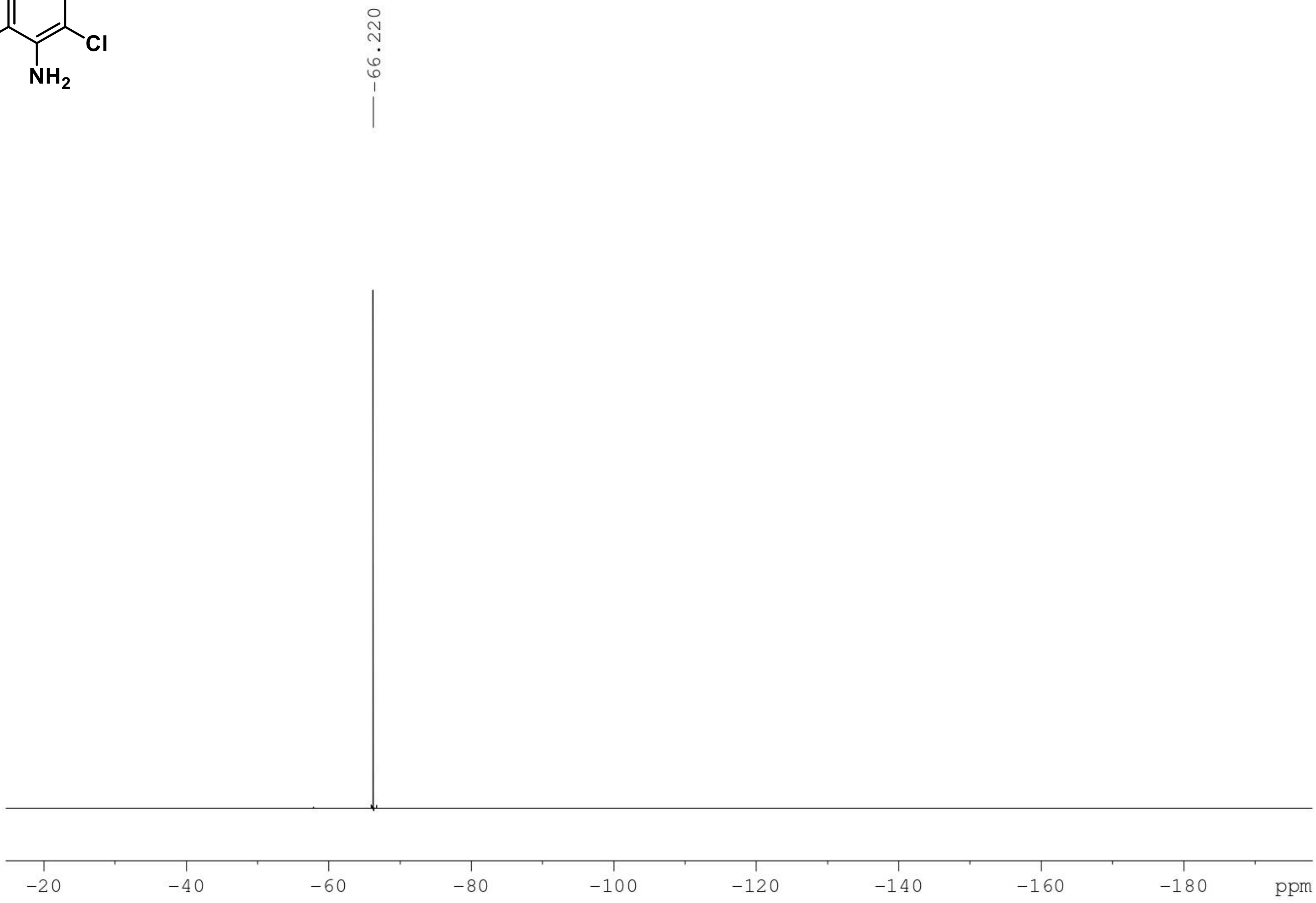

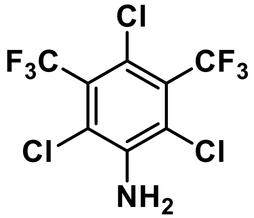

^1H, $CDCl_3$ (400 MHz)

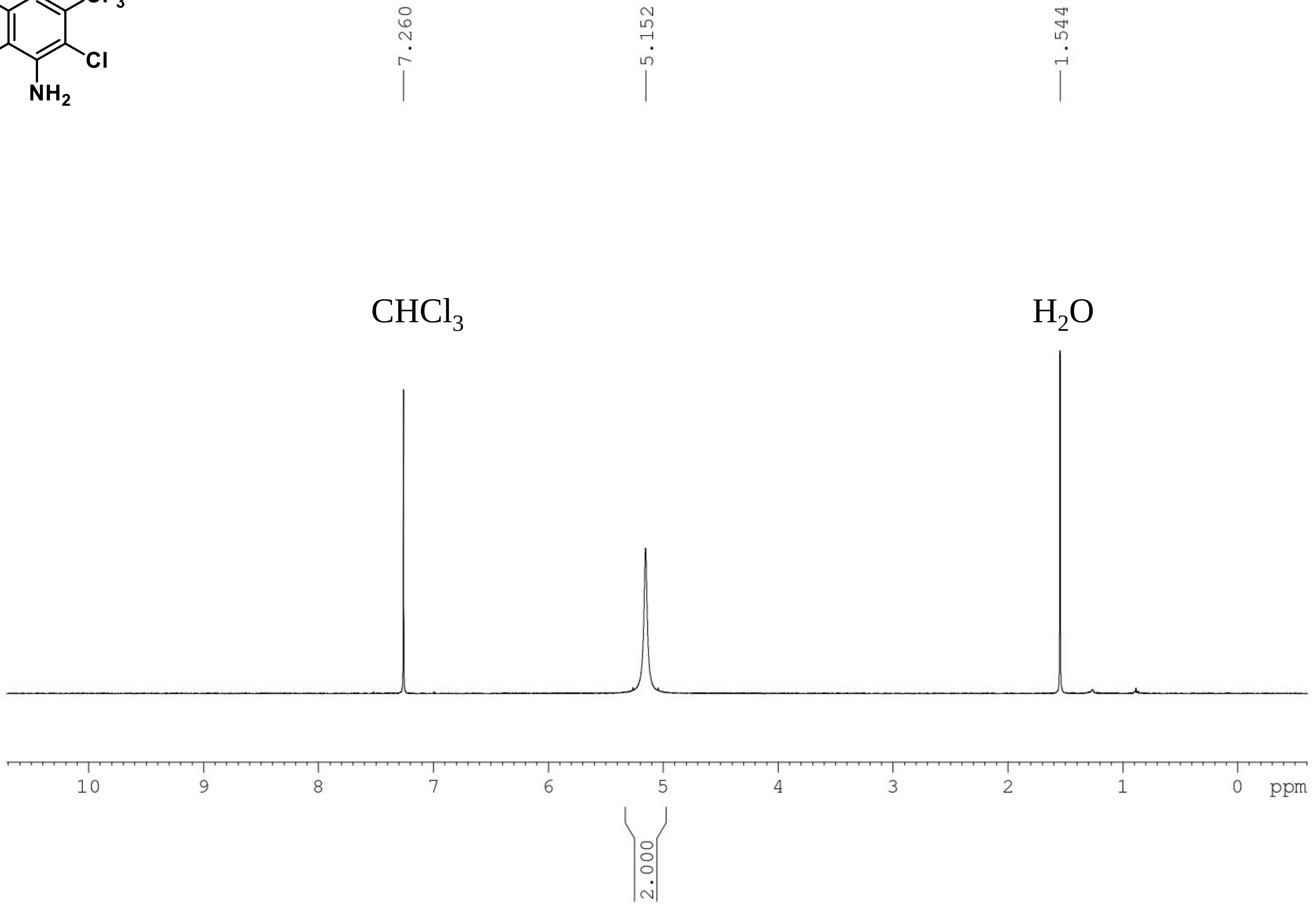

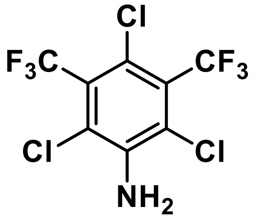

^{13}C, $CDCl_3$ (100 MHz)

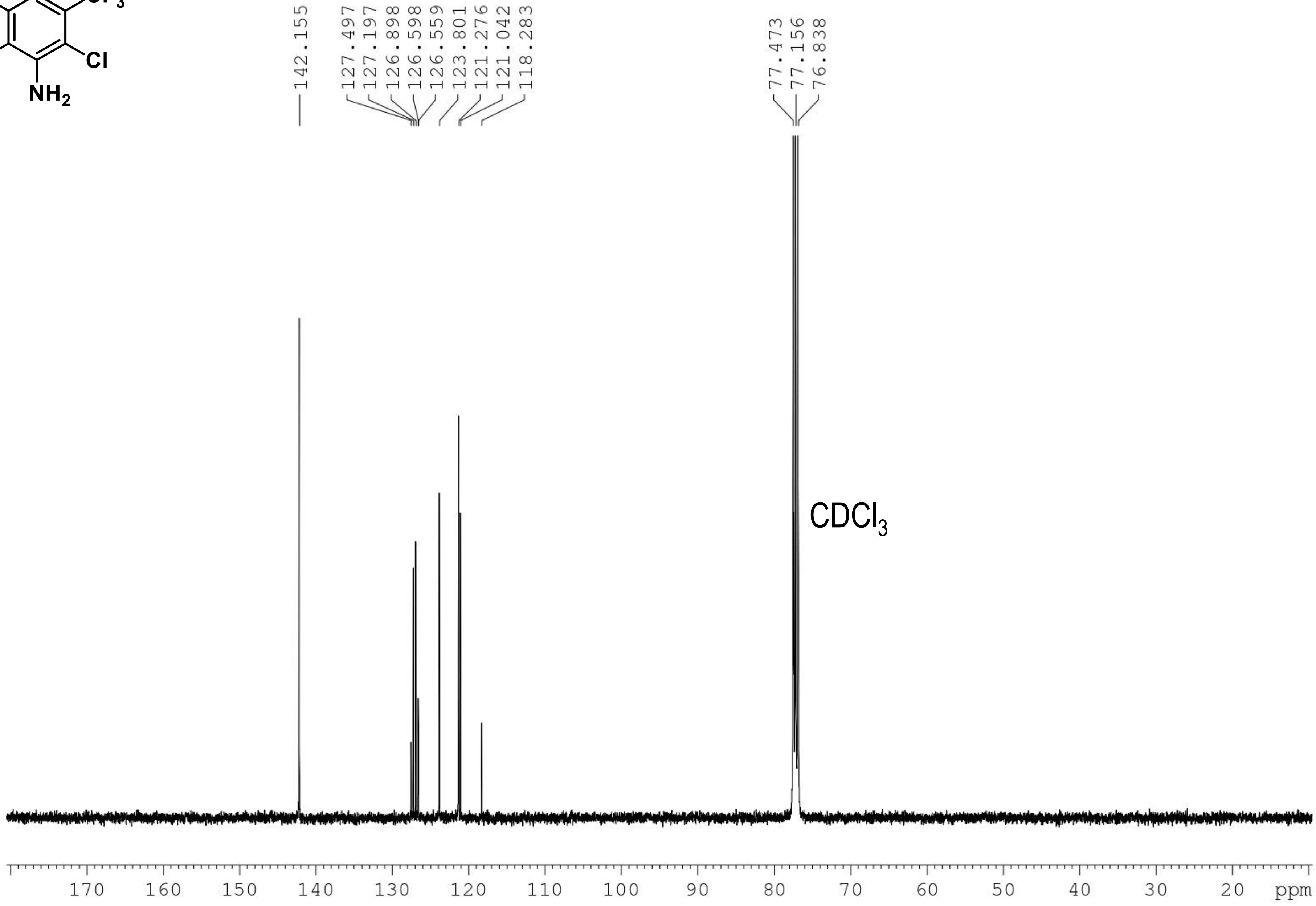

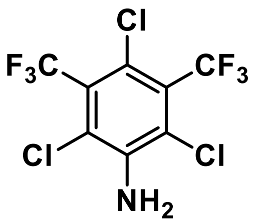

^{19}F, $CDCl_3$ (376 MHz)

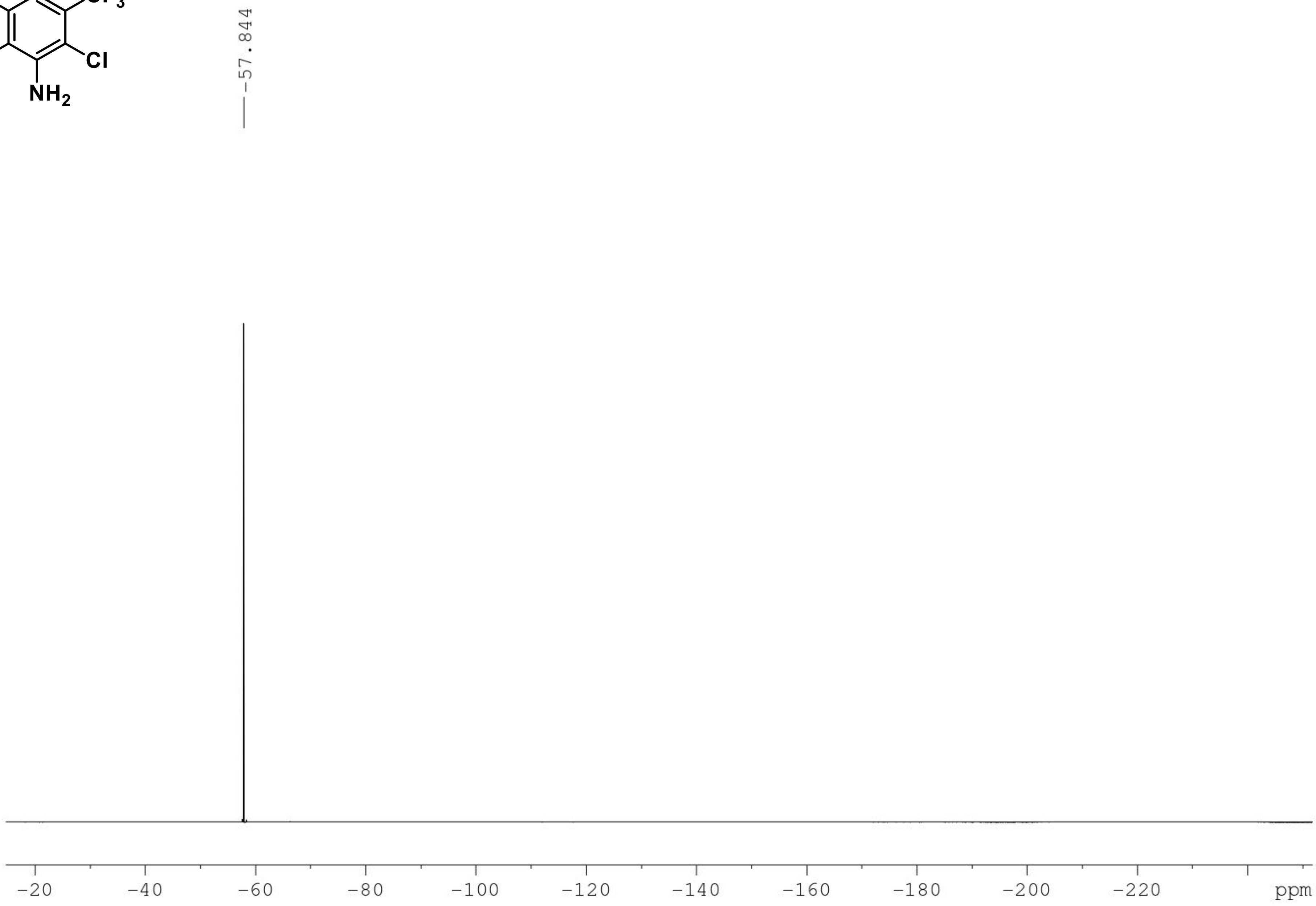

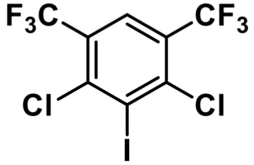

^1H, $CDCl_3$ (400 MHz)

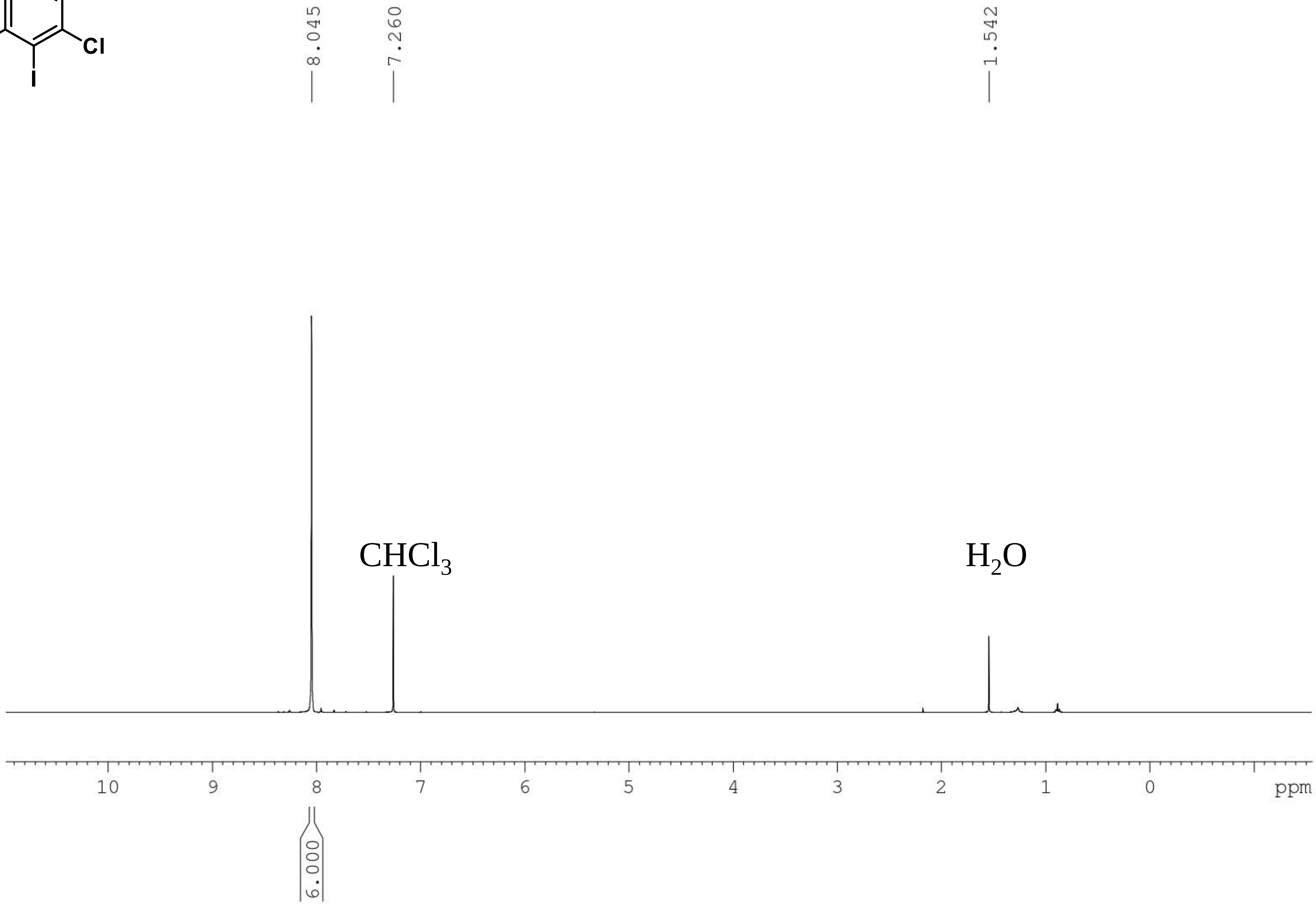

^{13}C, $CDCl_3$ (100 MHz)

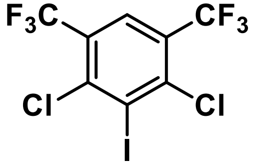

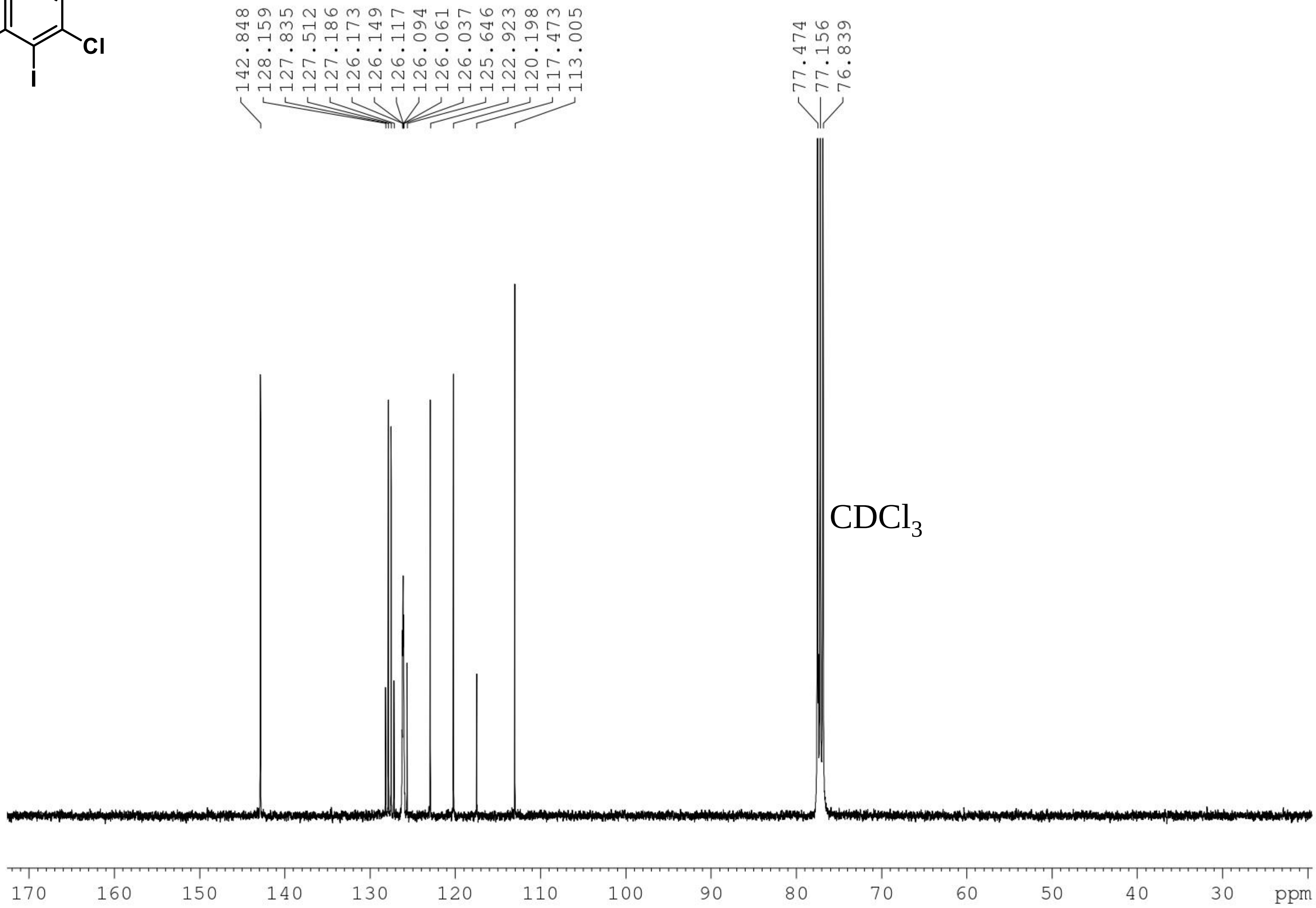

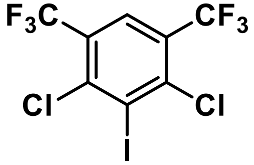

^{19}F, $CDCl_3$ (376 MHz)

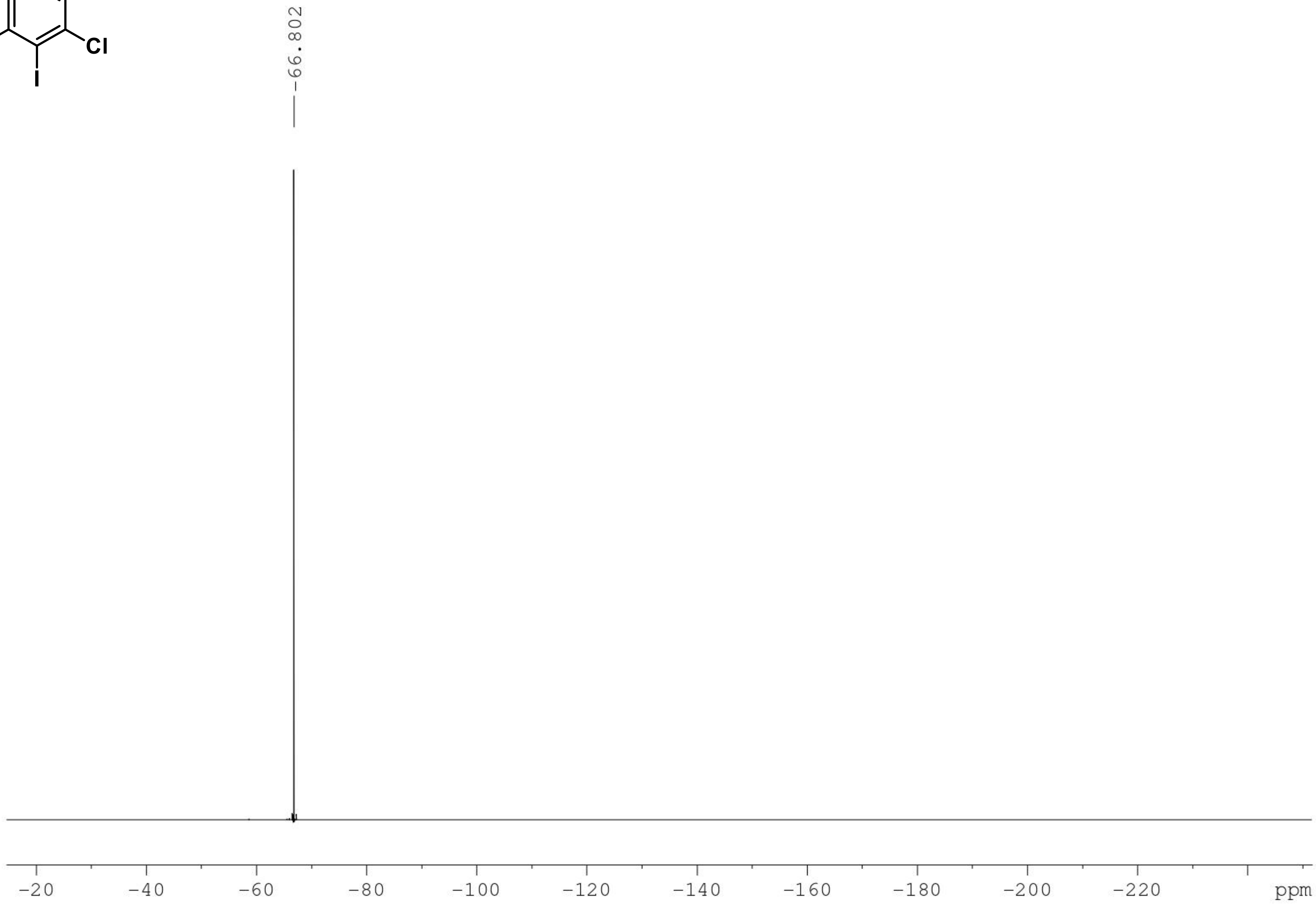

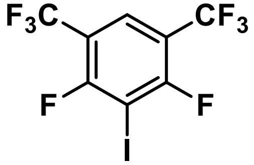

^1H, $CDCl_3$ (400 MHz)

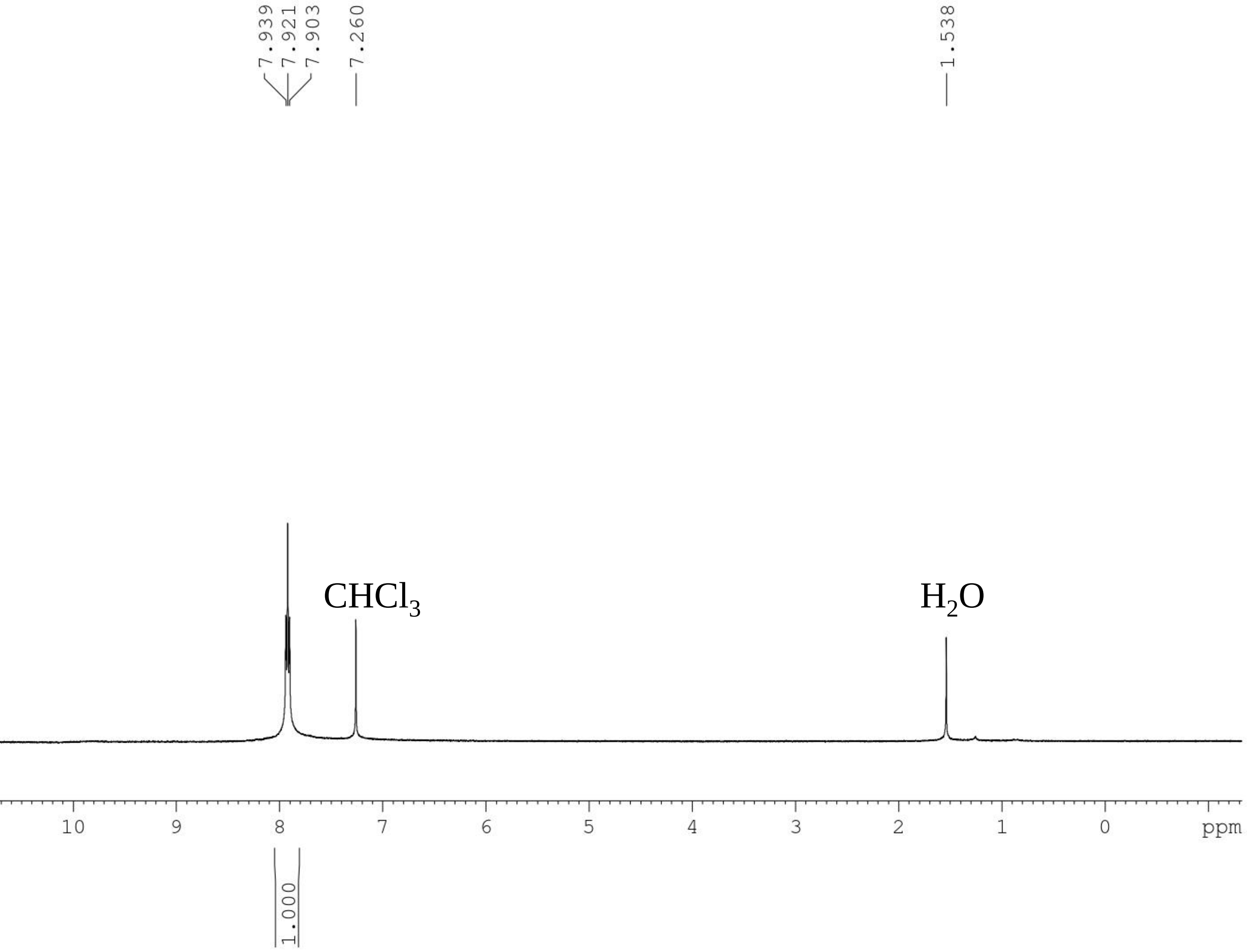

^{13}C, $CDCl_3$ (100 MHz)

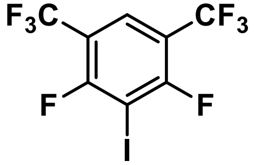

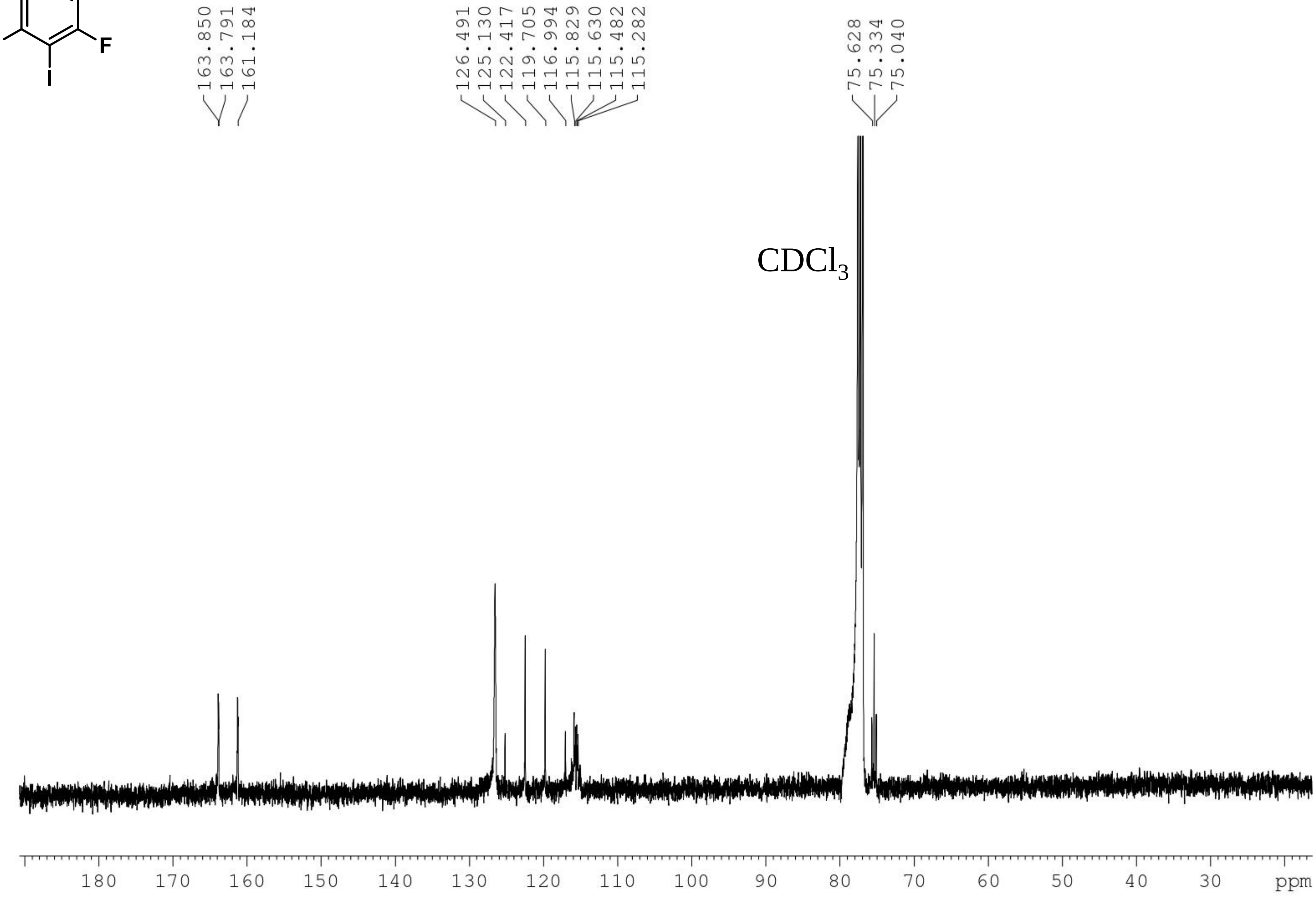

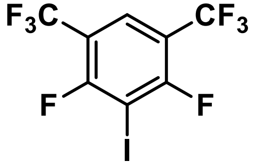

^{19}F, $CDCl_3$ (376 MHz)

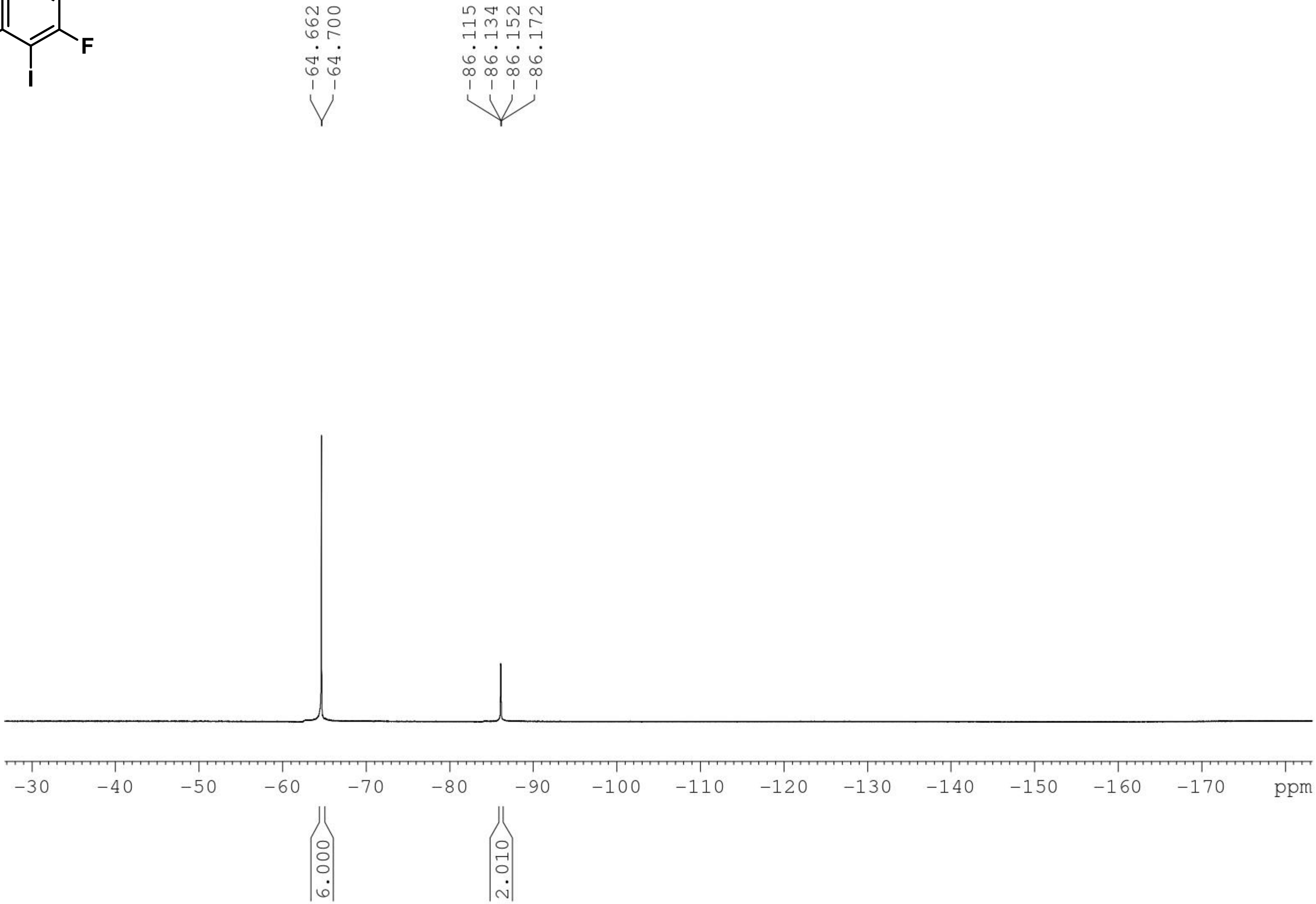

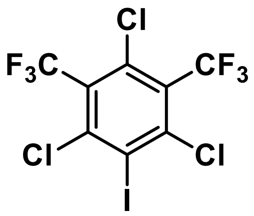

^{13}C, $CDCl_3$ (100 MHz)

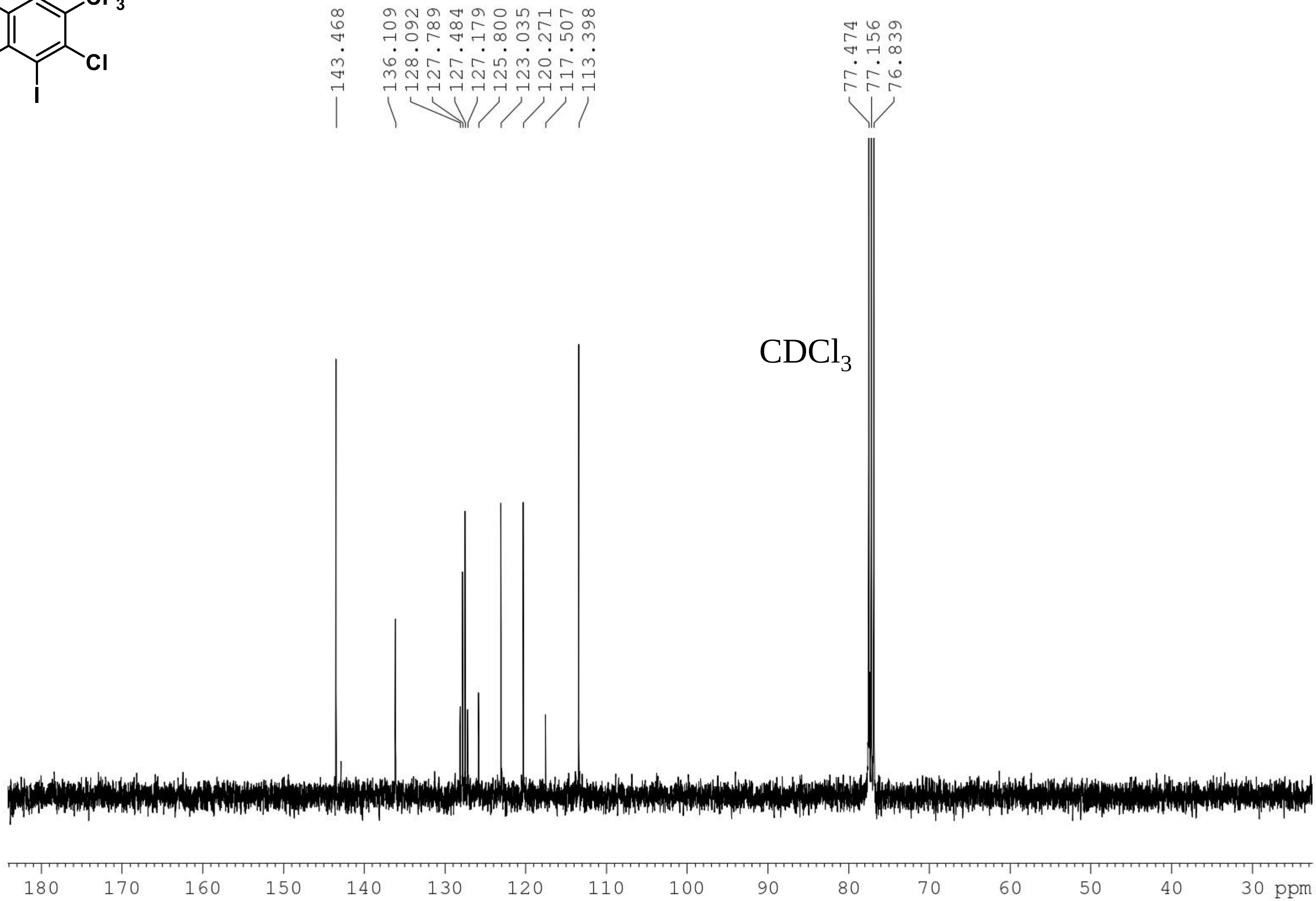

^{19}F, $CDCl_3$ (376 MHz)

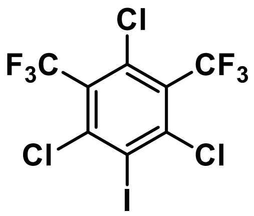

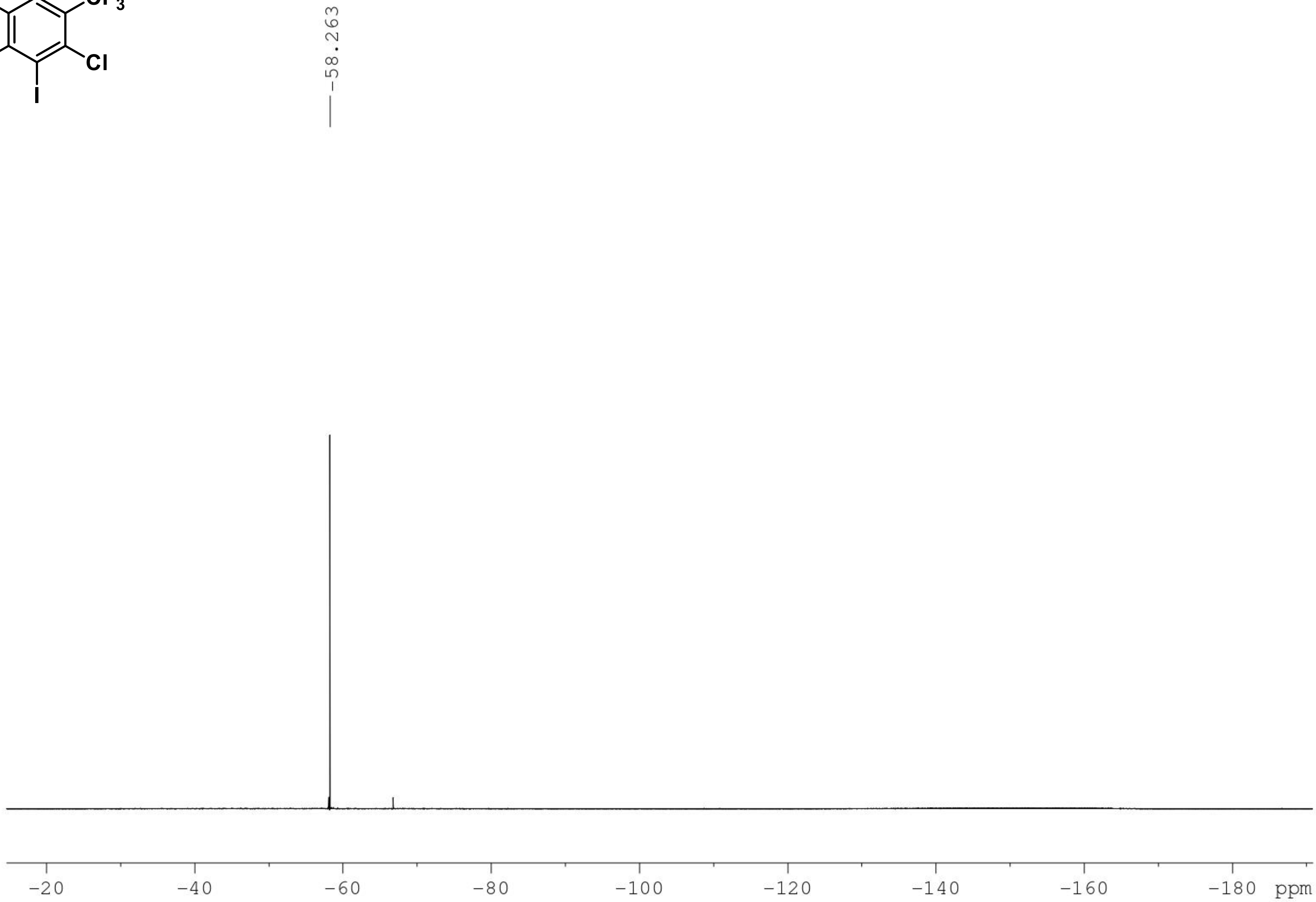

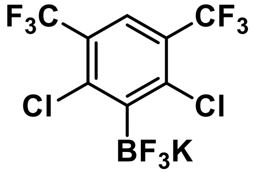

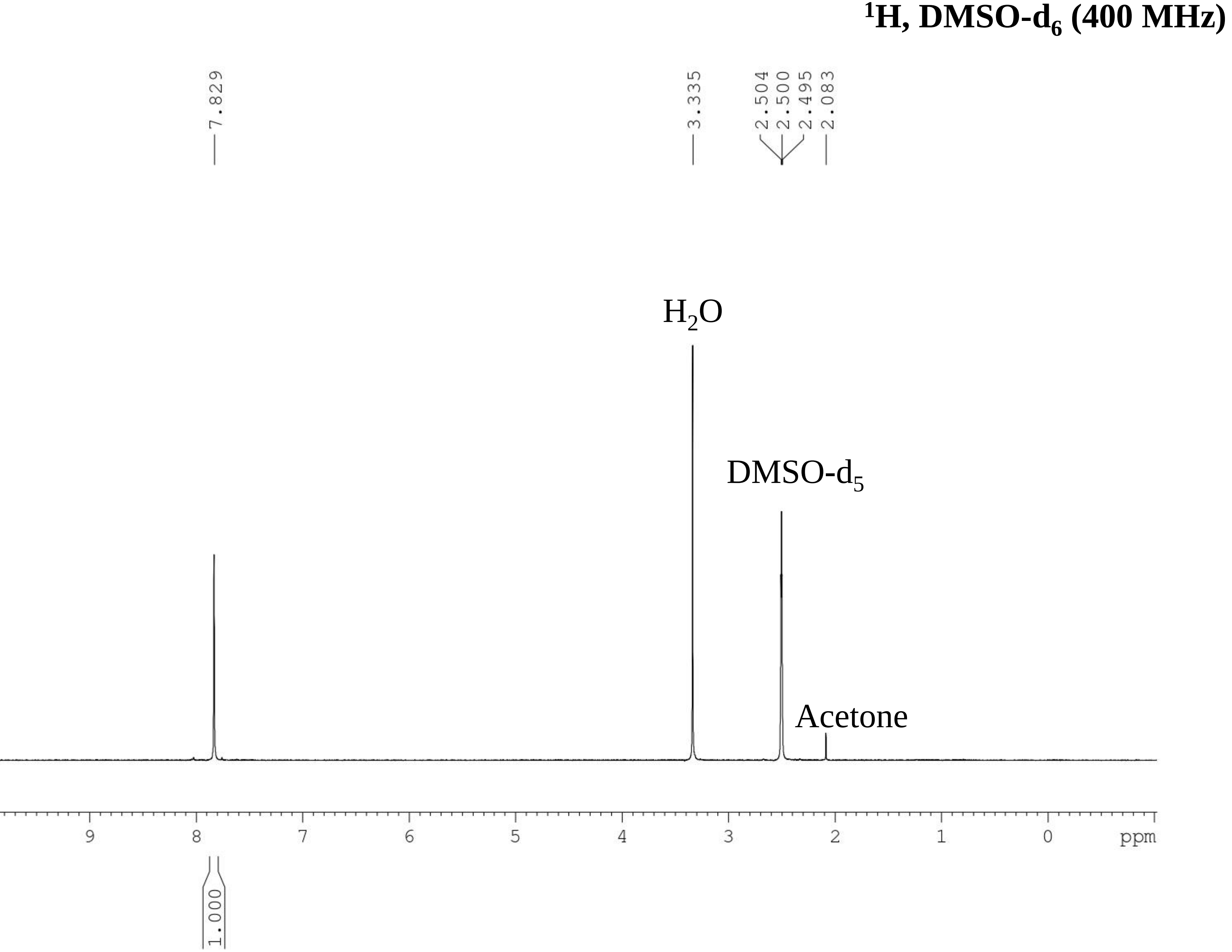

1H, DMSO-d6 (400 MHz)
7.829
3.335
2.504
2.500
2.495
2.083
H2O
DMSO-d5
Acetone
10
9
8
7
6
5
4
3
2
1
0
ppm
1.000

^{11}B, DMSO-d$_6$ (128 MHz)

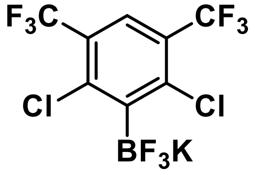

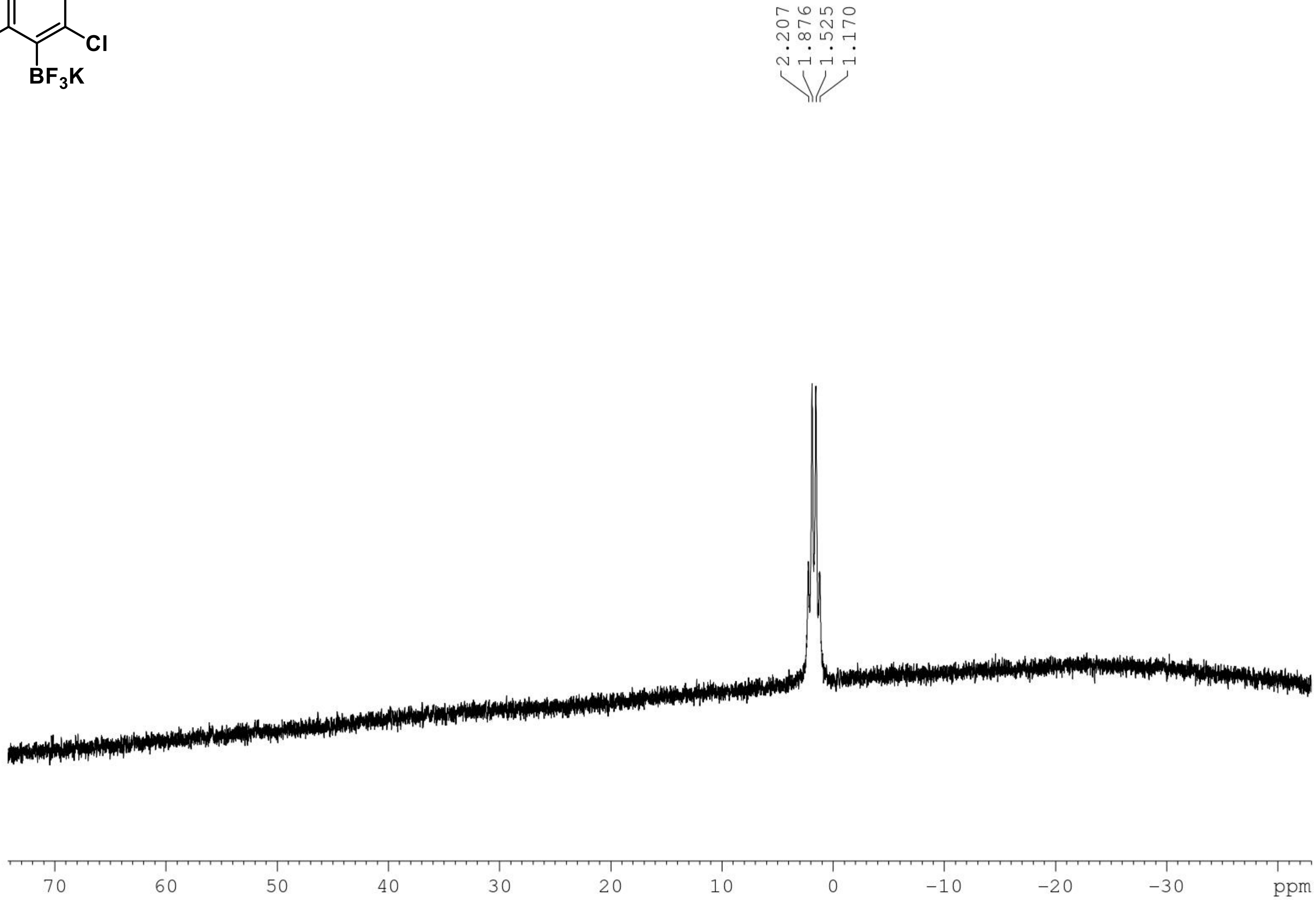

^{13}C, DMSO-d_6 (100 MHz)

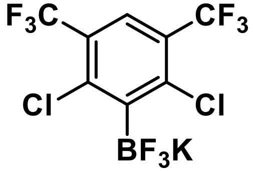

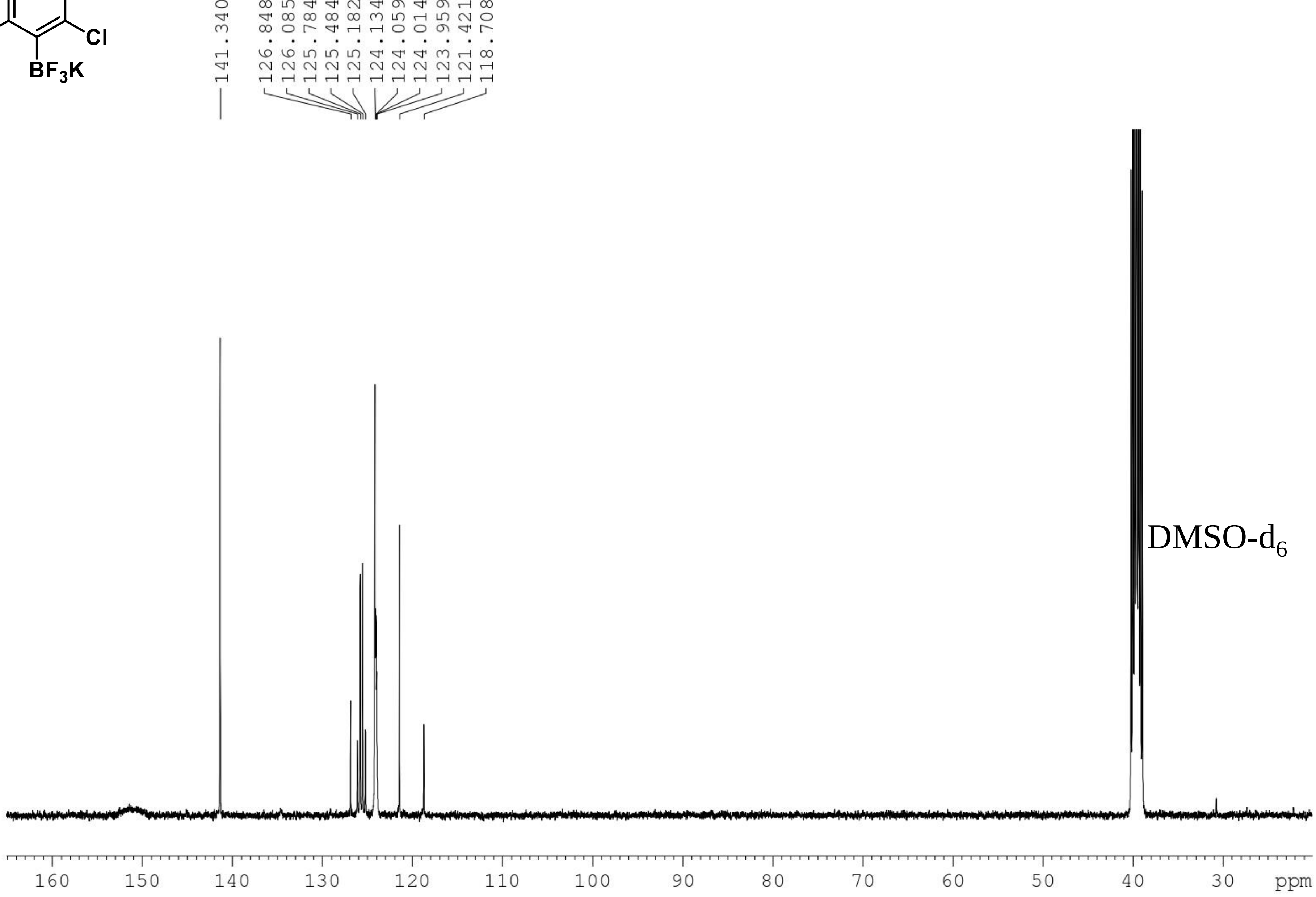

^{19}F, DMSO-d$_6$ (376 MHz)

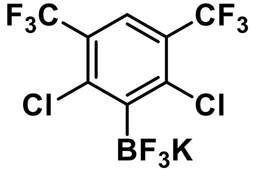

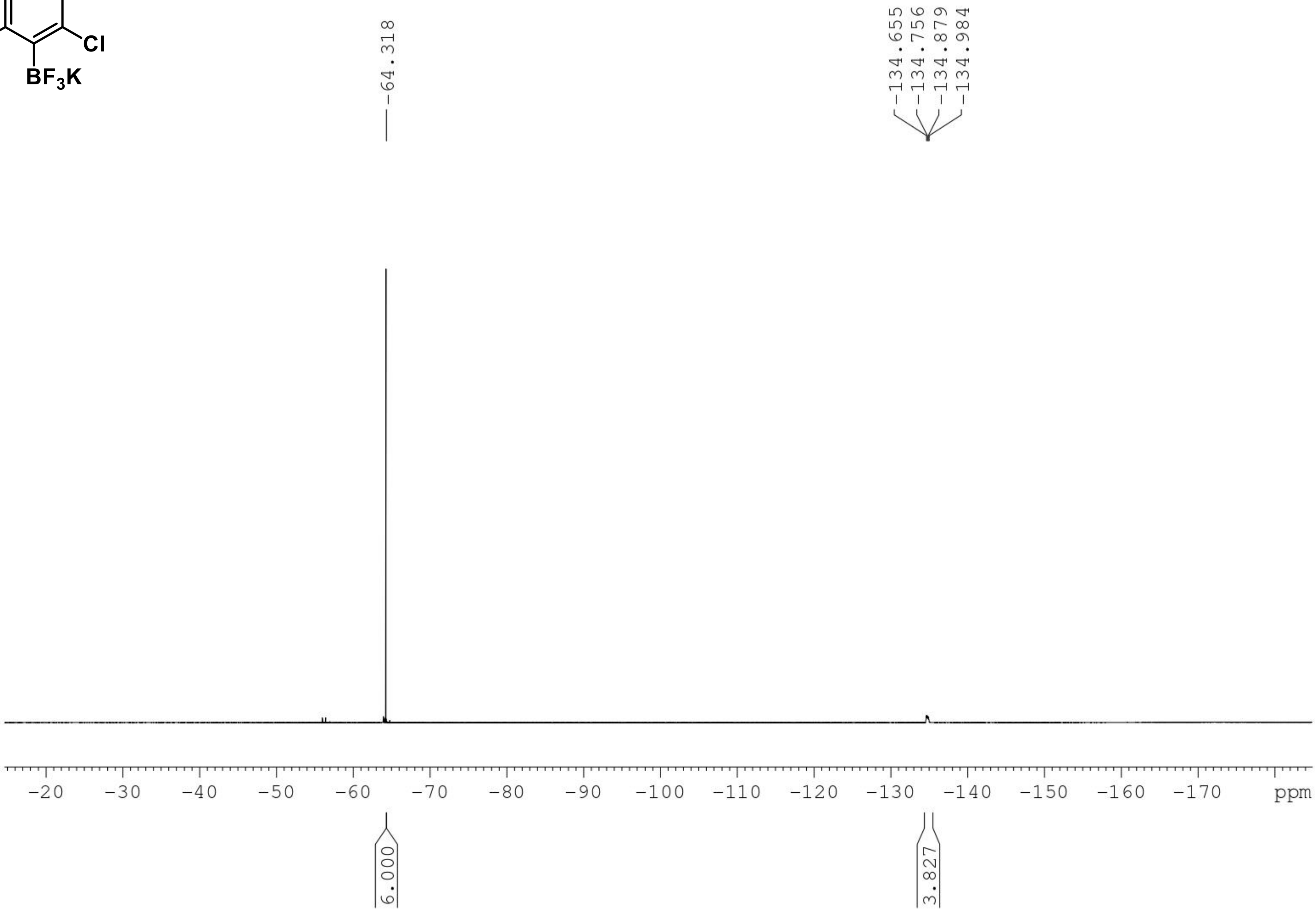

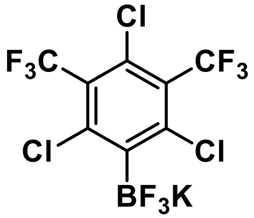

^{11}B, DMSO-d$_6$ (128 MHz)

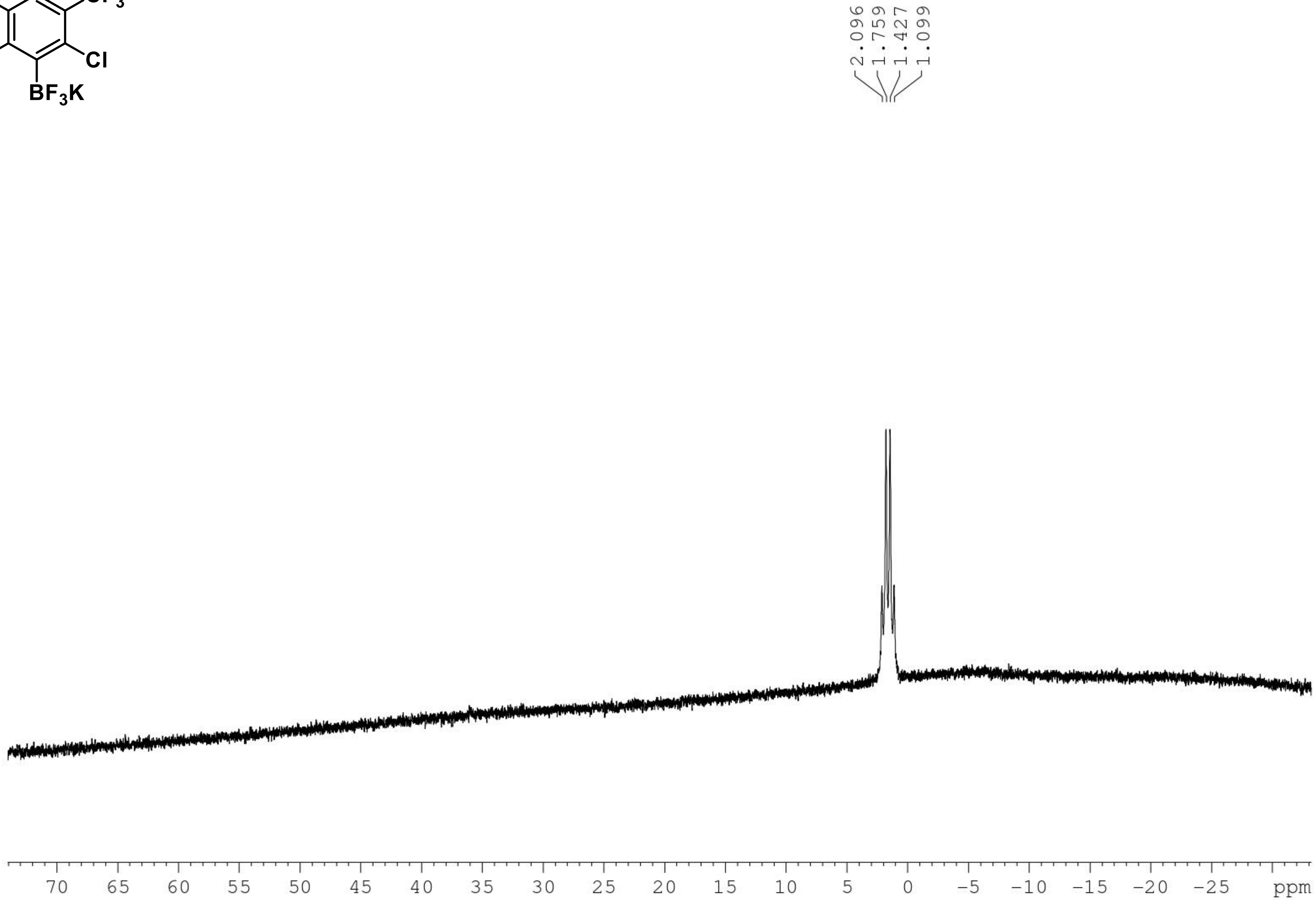

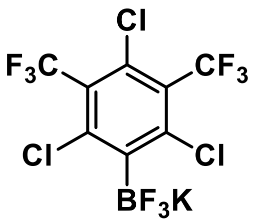

^{13}C, DMSO-d_6 (100 MHz)

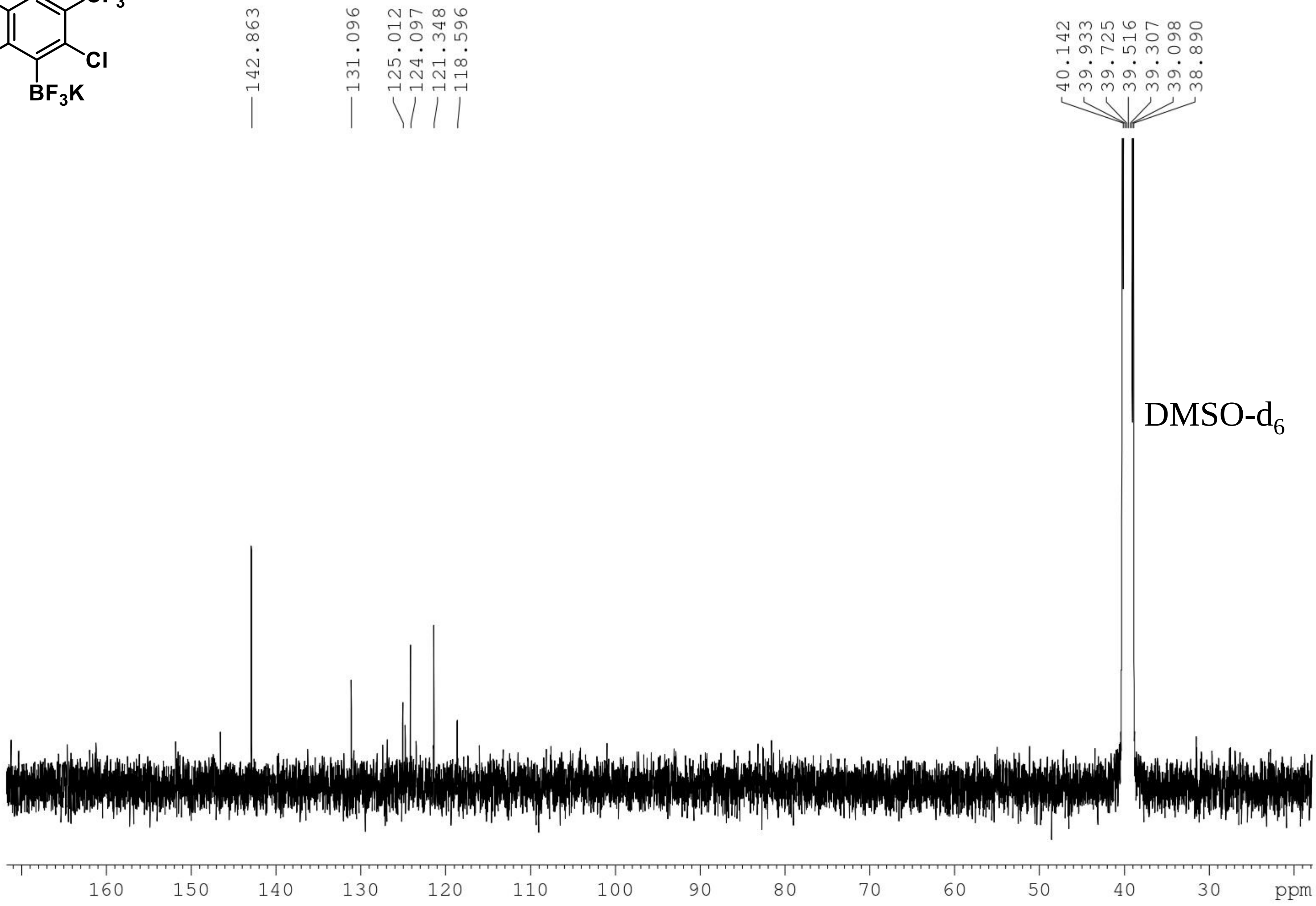

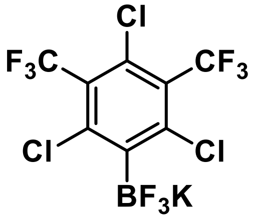

^{19}F, DMSO-d_6 (376 MHz)

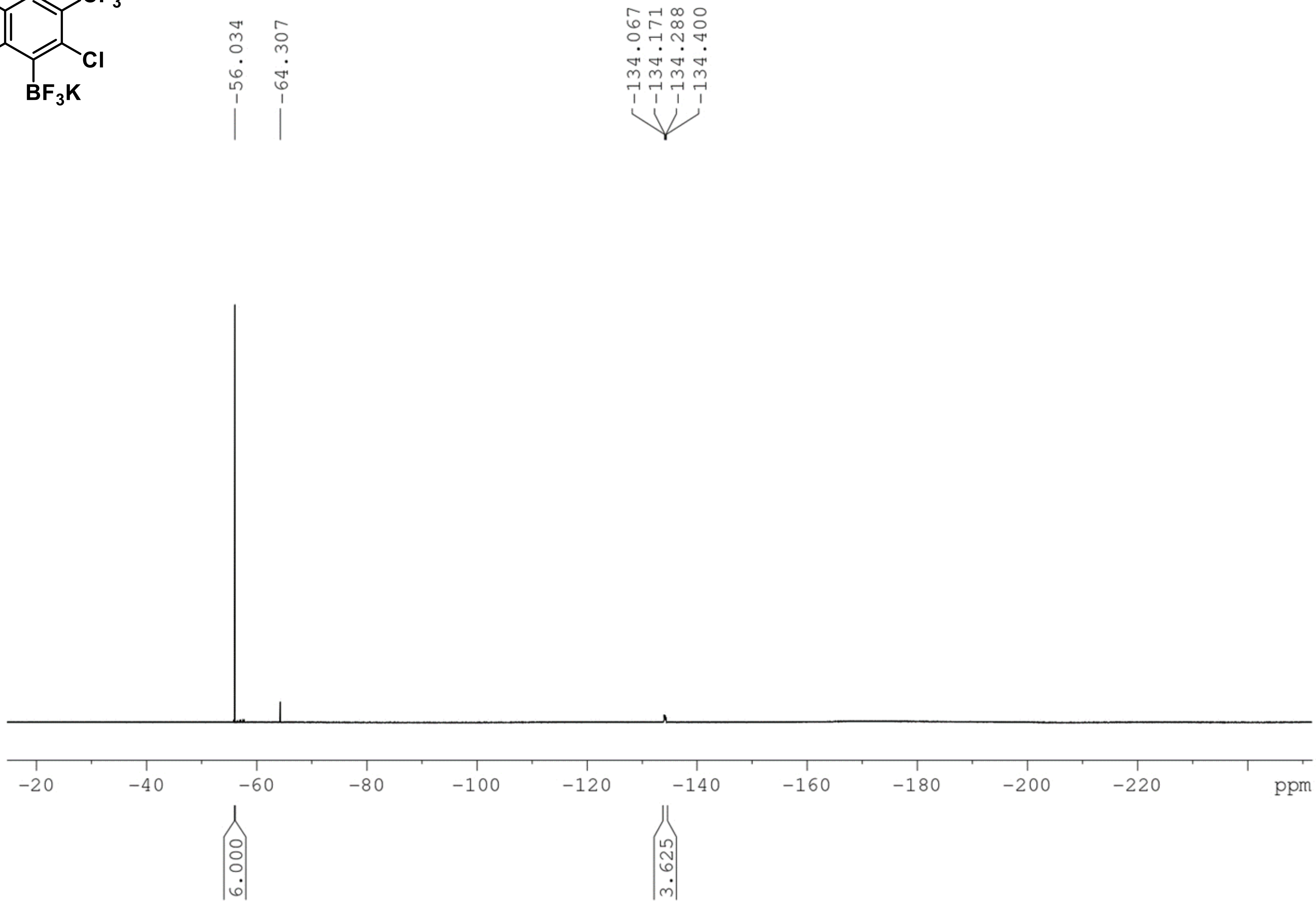

^1H, DMSO-d_6 (400 MHz)

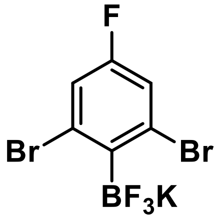

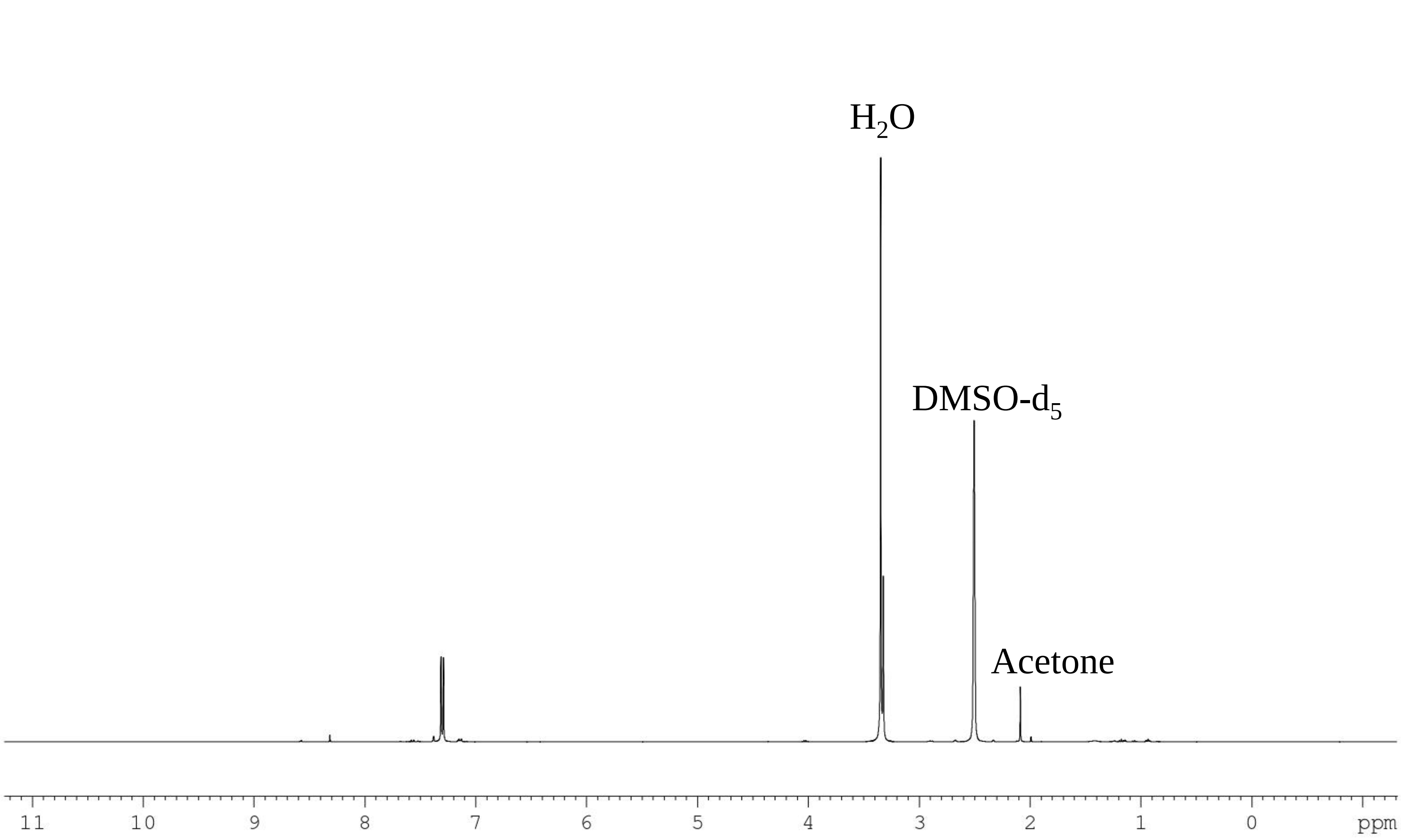

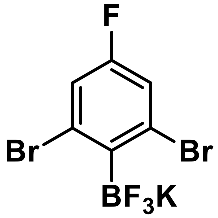

^{11}B, DMSO-d$_6$ (128 MHz)

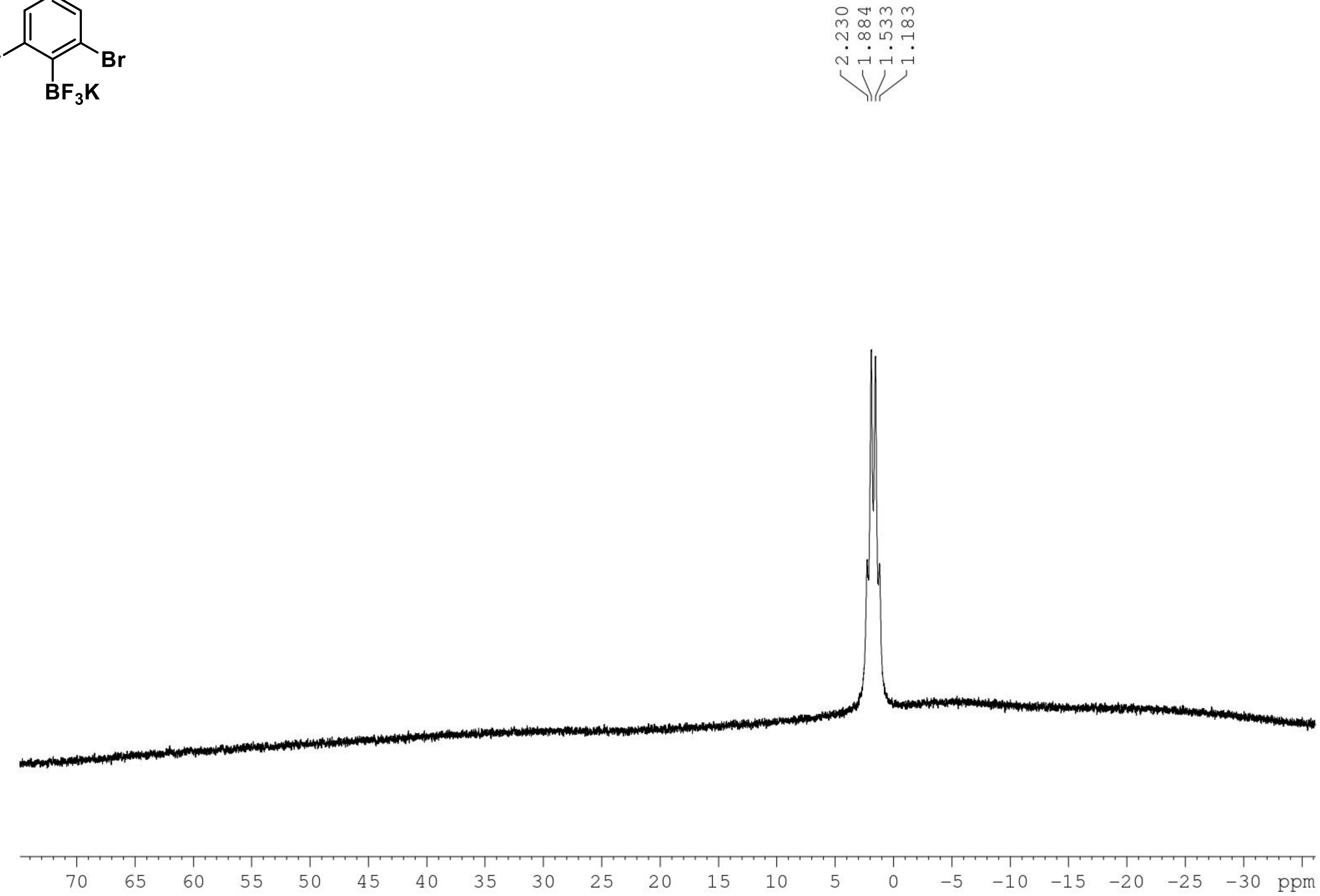

^{13}C, DMSO-d_6 (100 MHz)

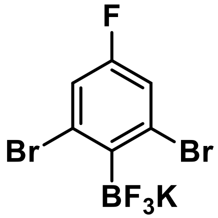

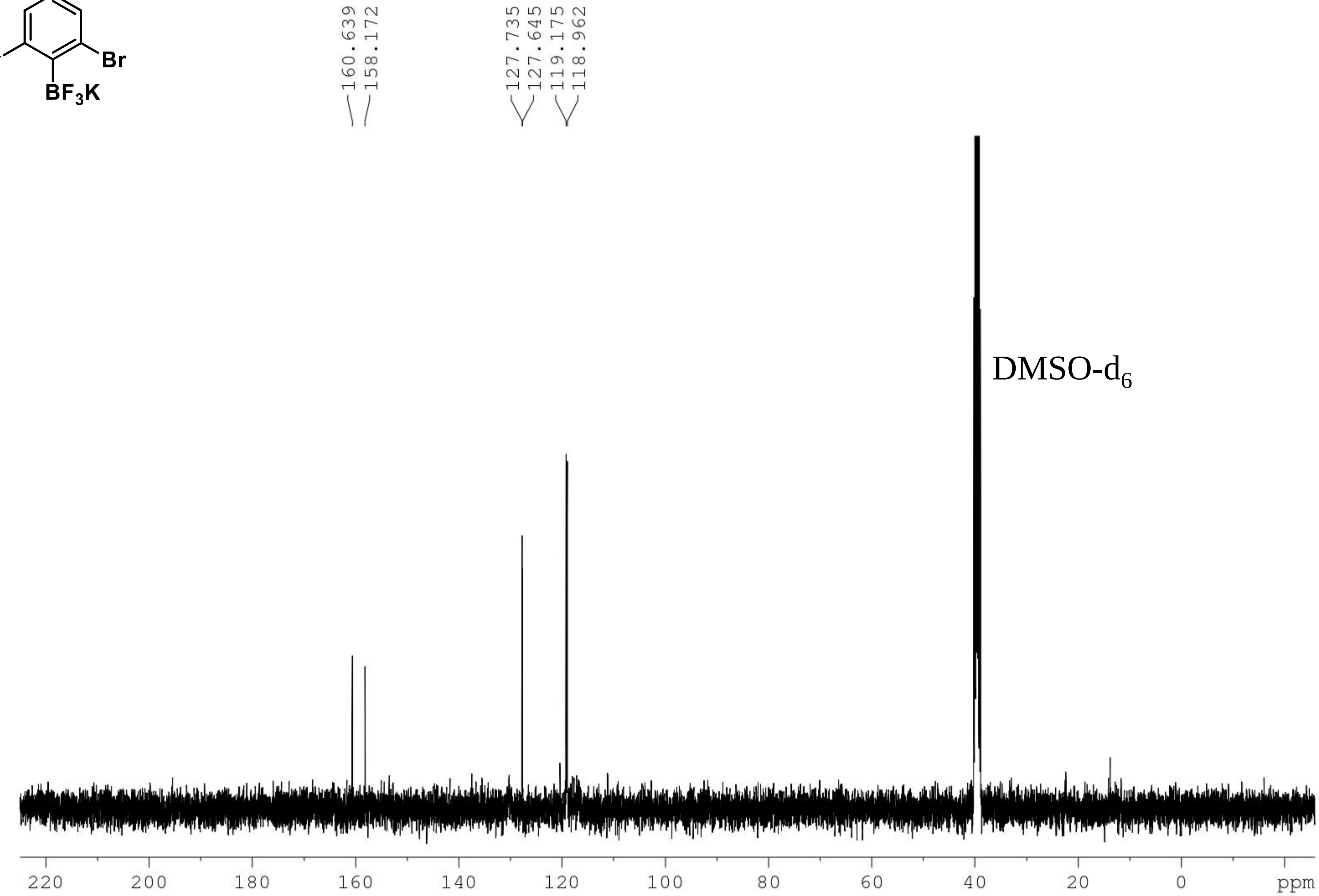

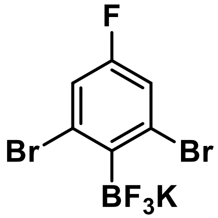

^{19}F, DMSO-d_6 (376 MHz)

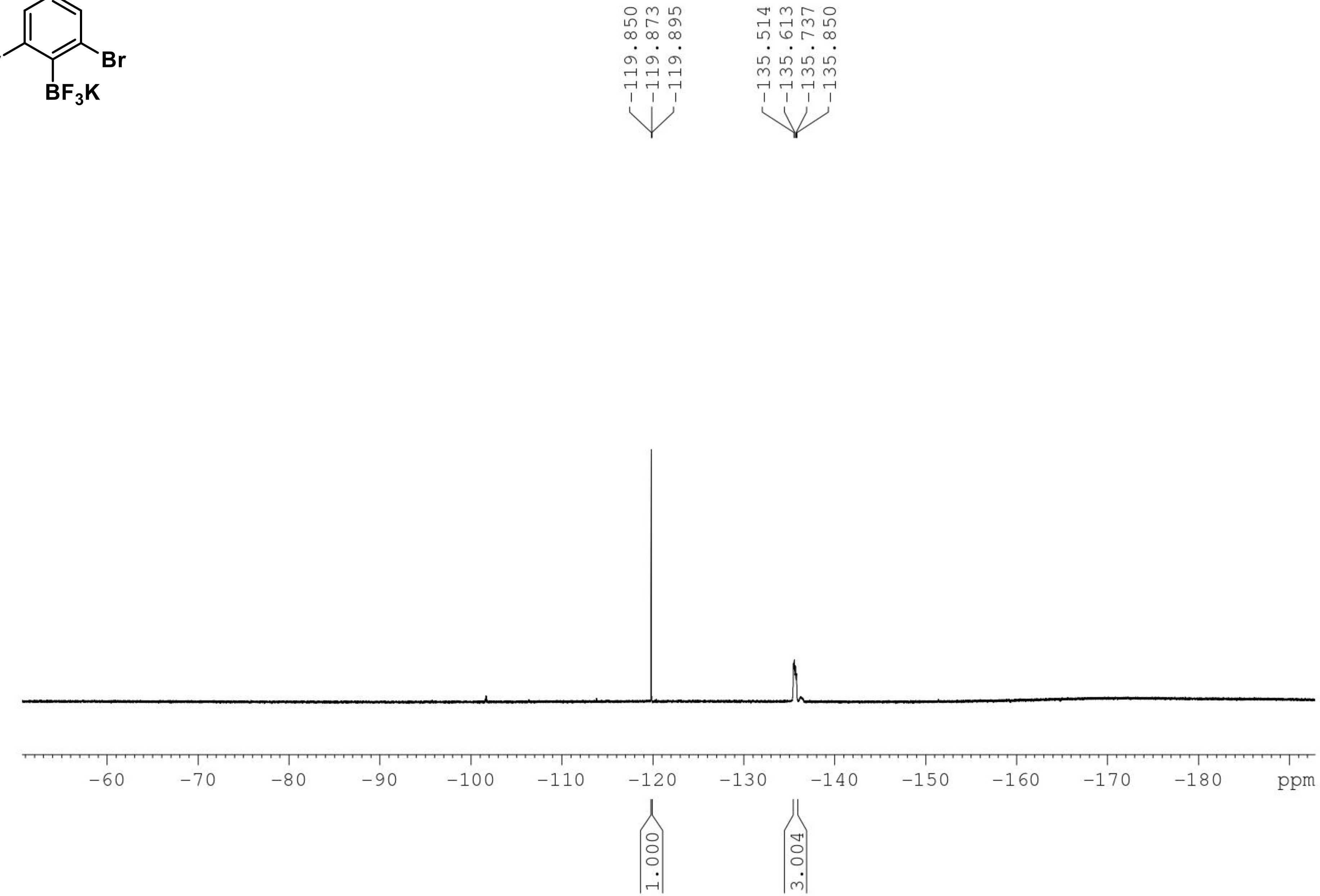

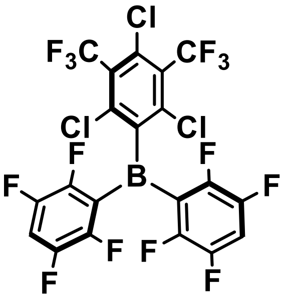

^{1}H, C_6D_6 (400 MHz)

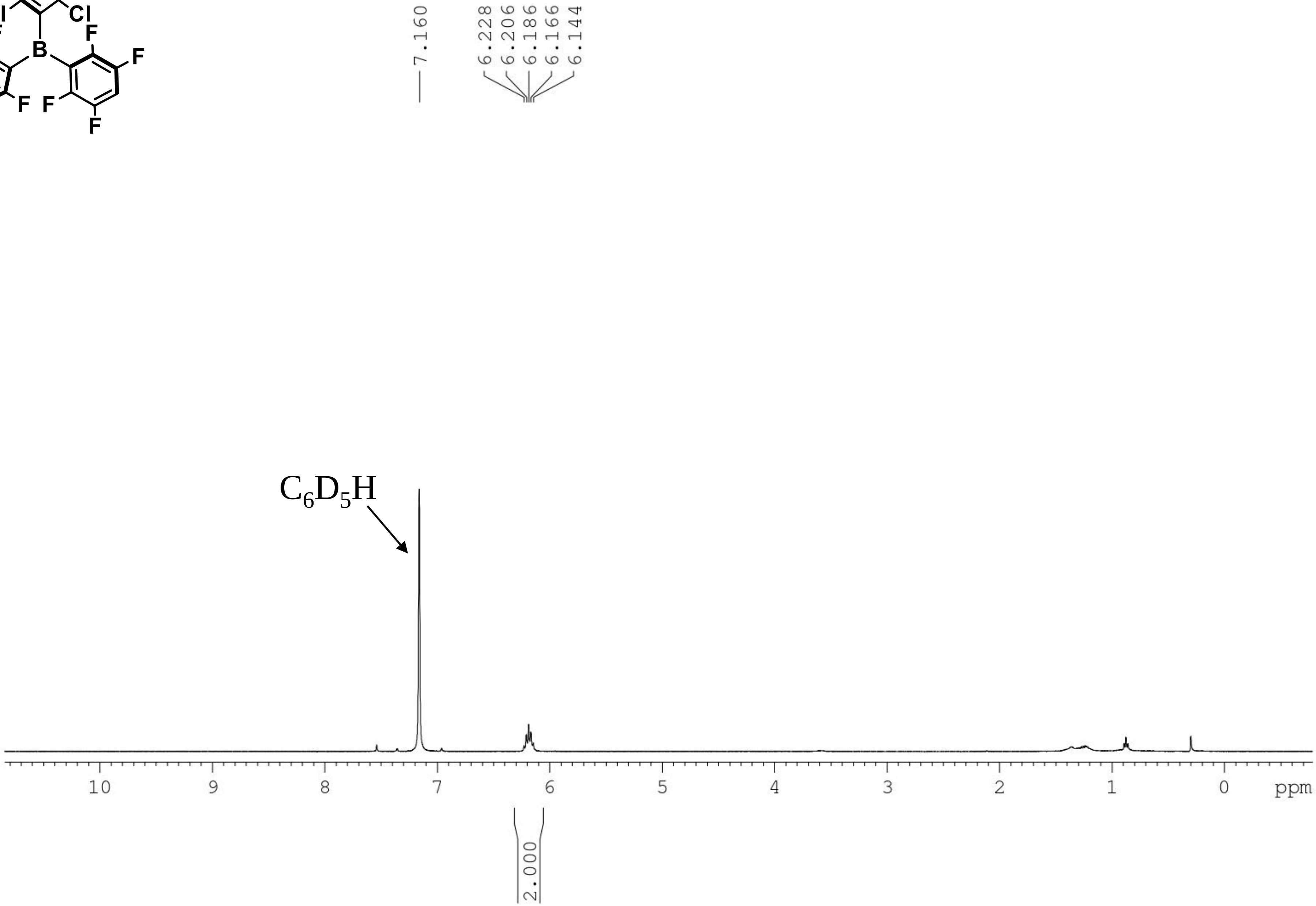

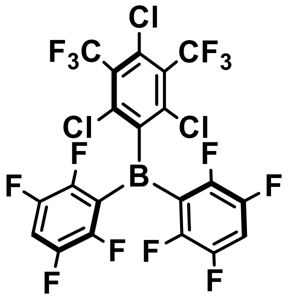

^{11}B, C_6D_6 (128 MHz)

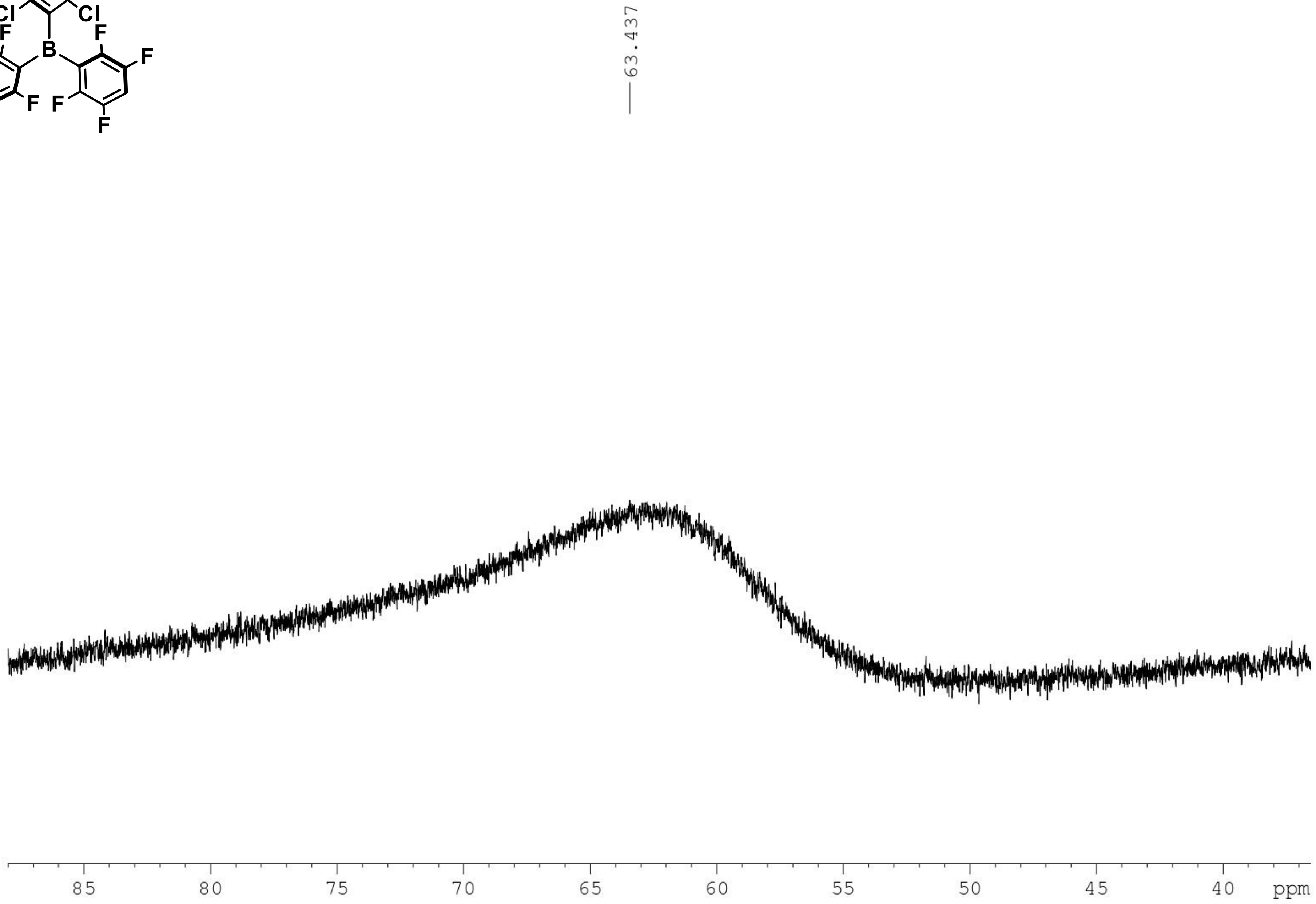

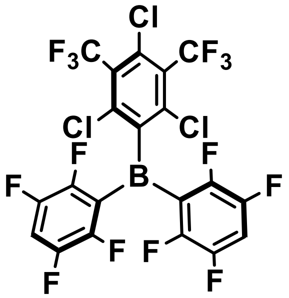

^{13}C, C_6D_6 (100 MHz)

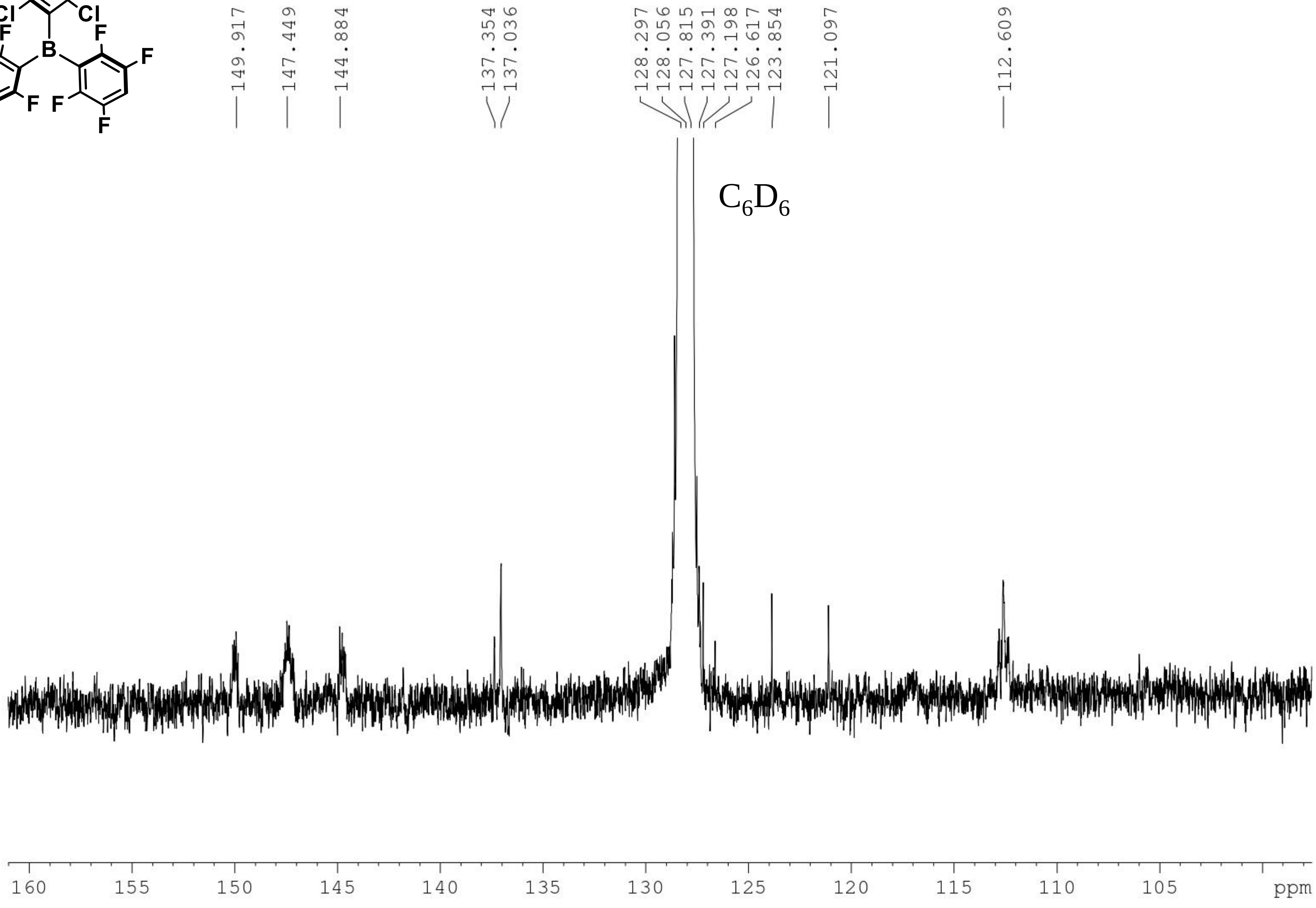

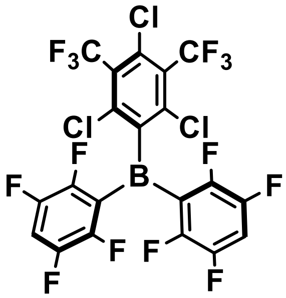

^{19}F, C_6D_6 (376 MHz)

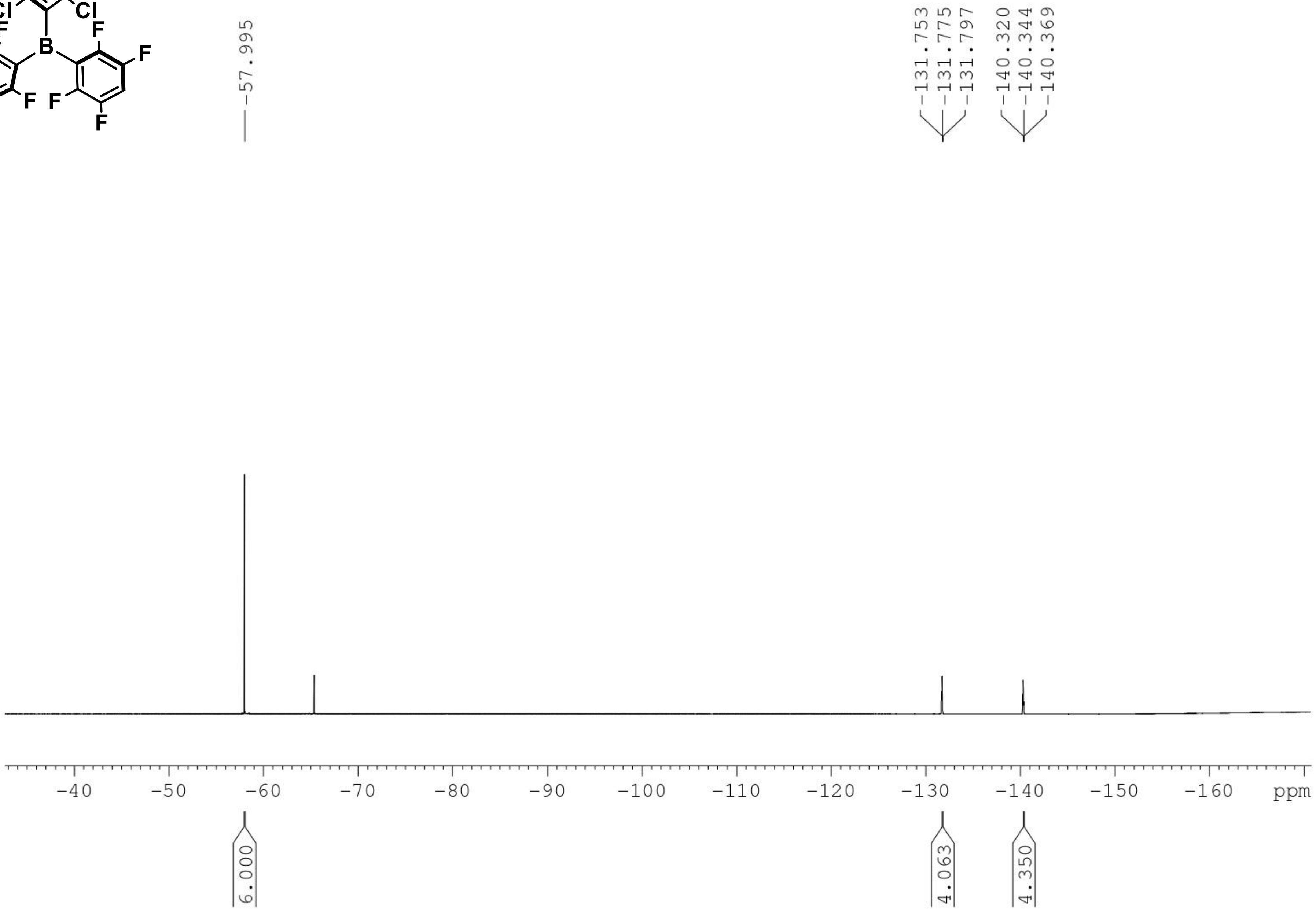

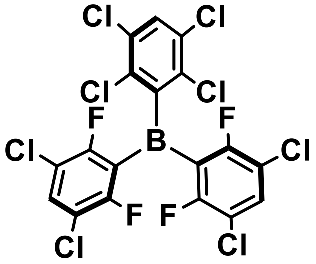

^1H, C_6D_6 (400 MHz)

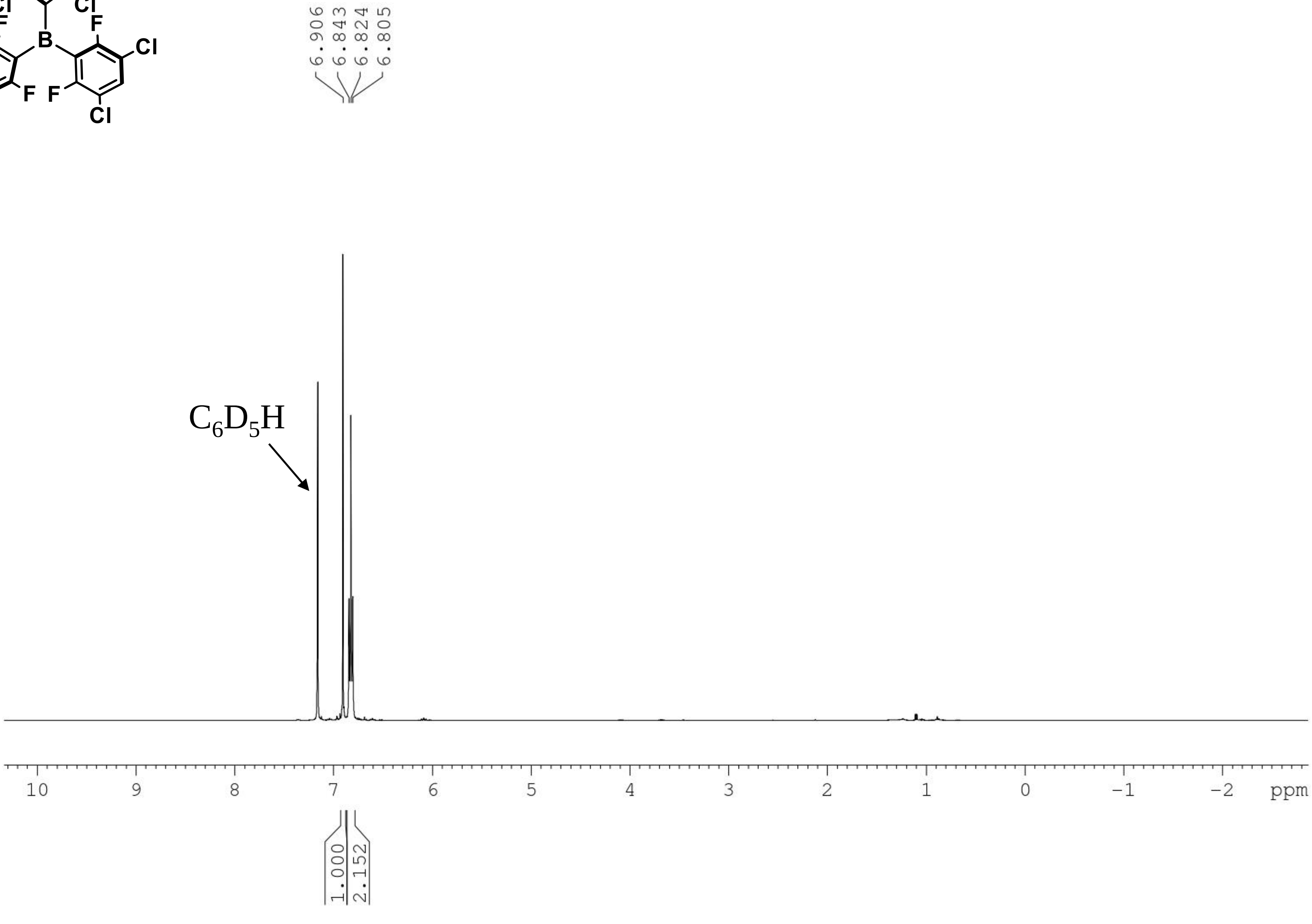

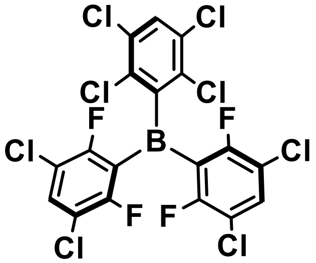

^{11}B, C_6D_6 (128 MHz)

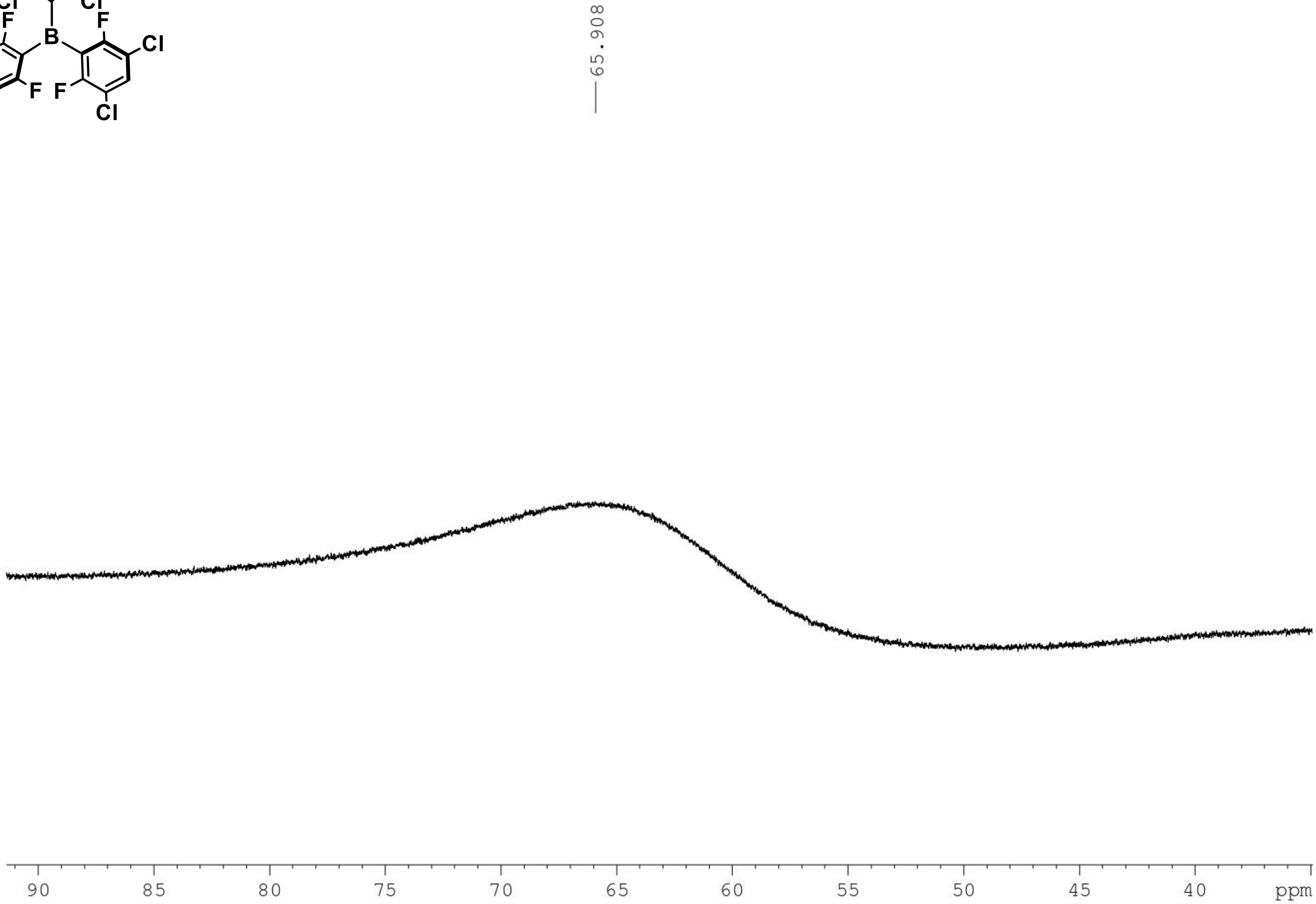

^{13}C, C_6D_6 (100 MHz)

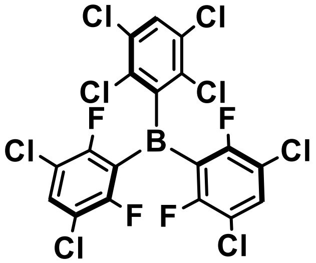

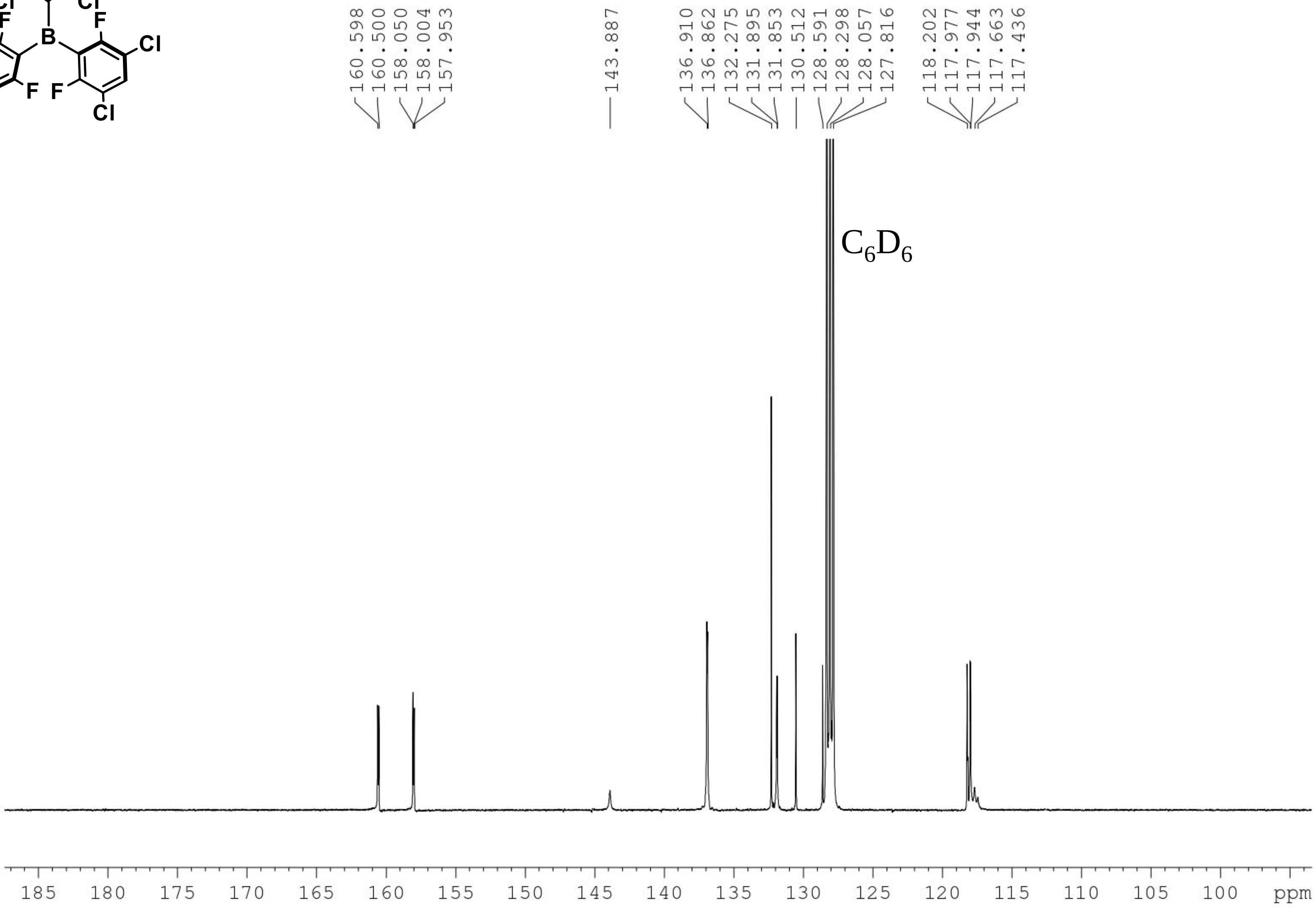

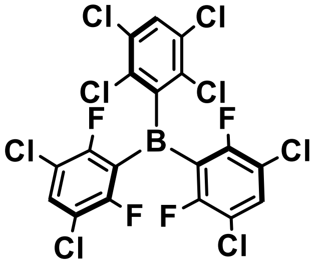

^{19}F, C_6D_6 (376 MHz)

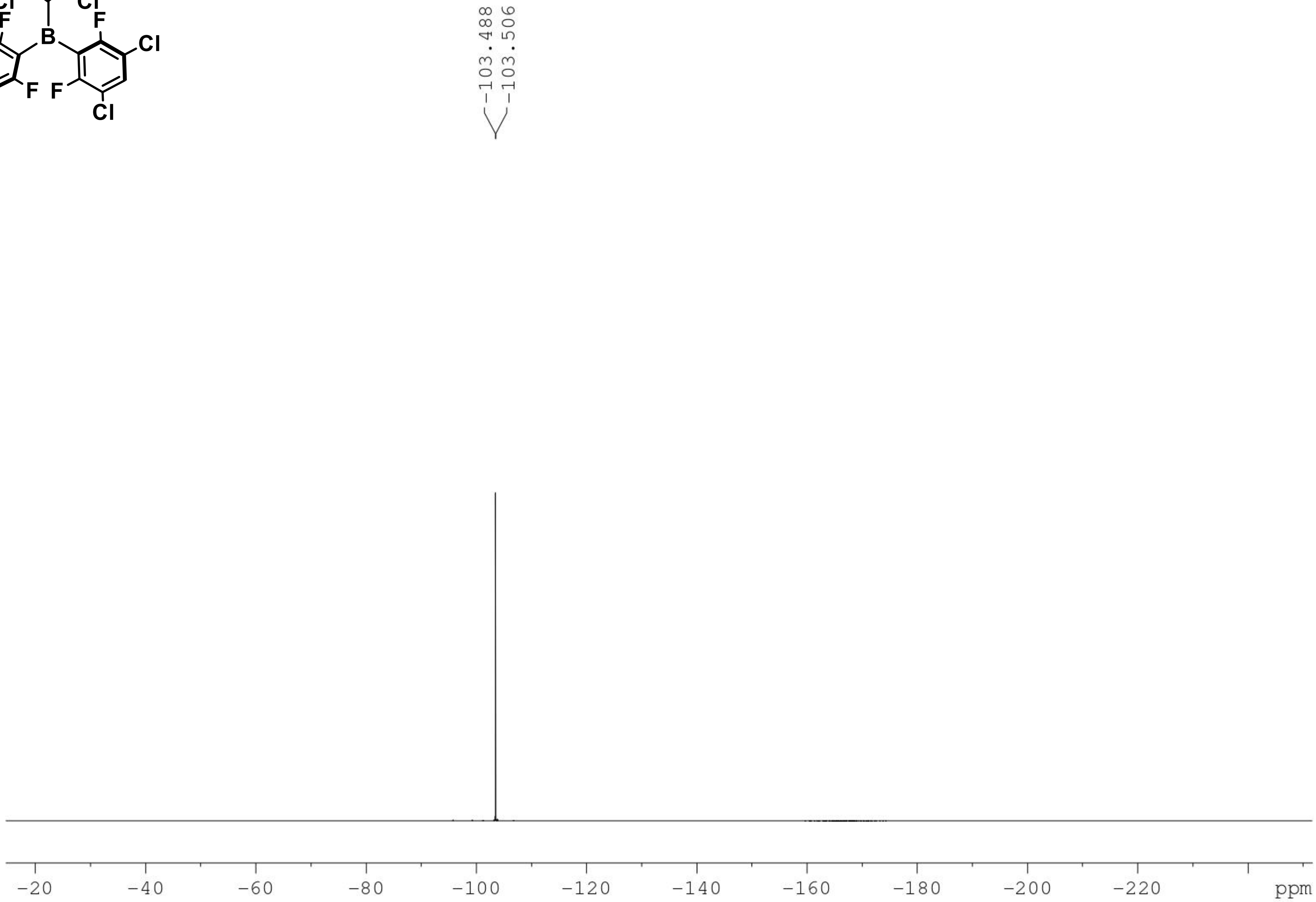

^{1}H, C_6D_6 (400 MHz)

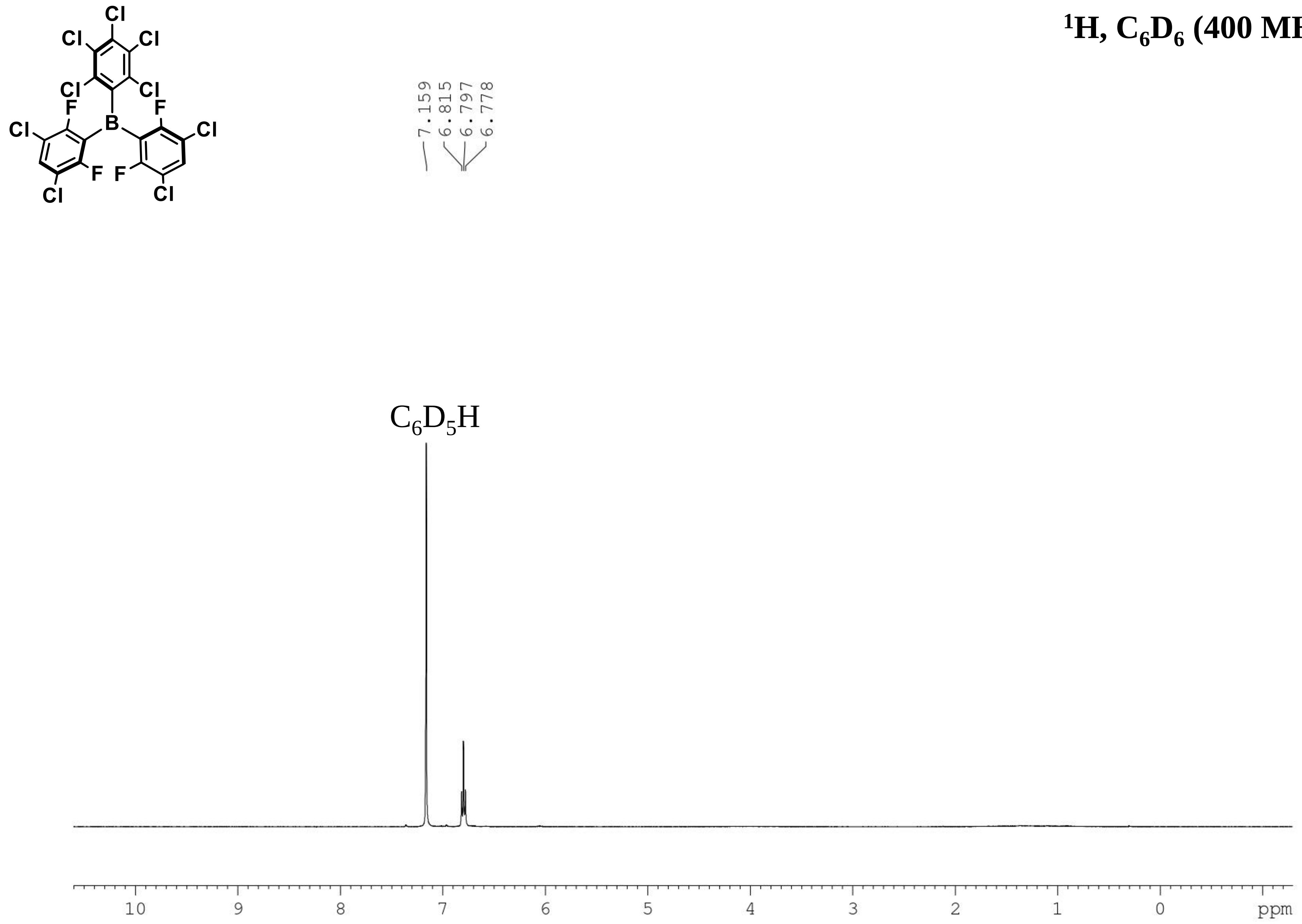

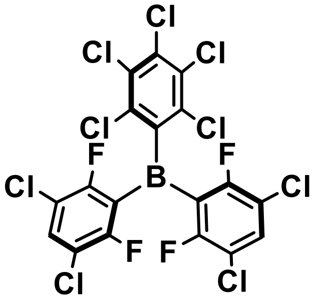

^{11}B, C_6D_6 (128 MHz)

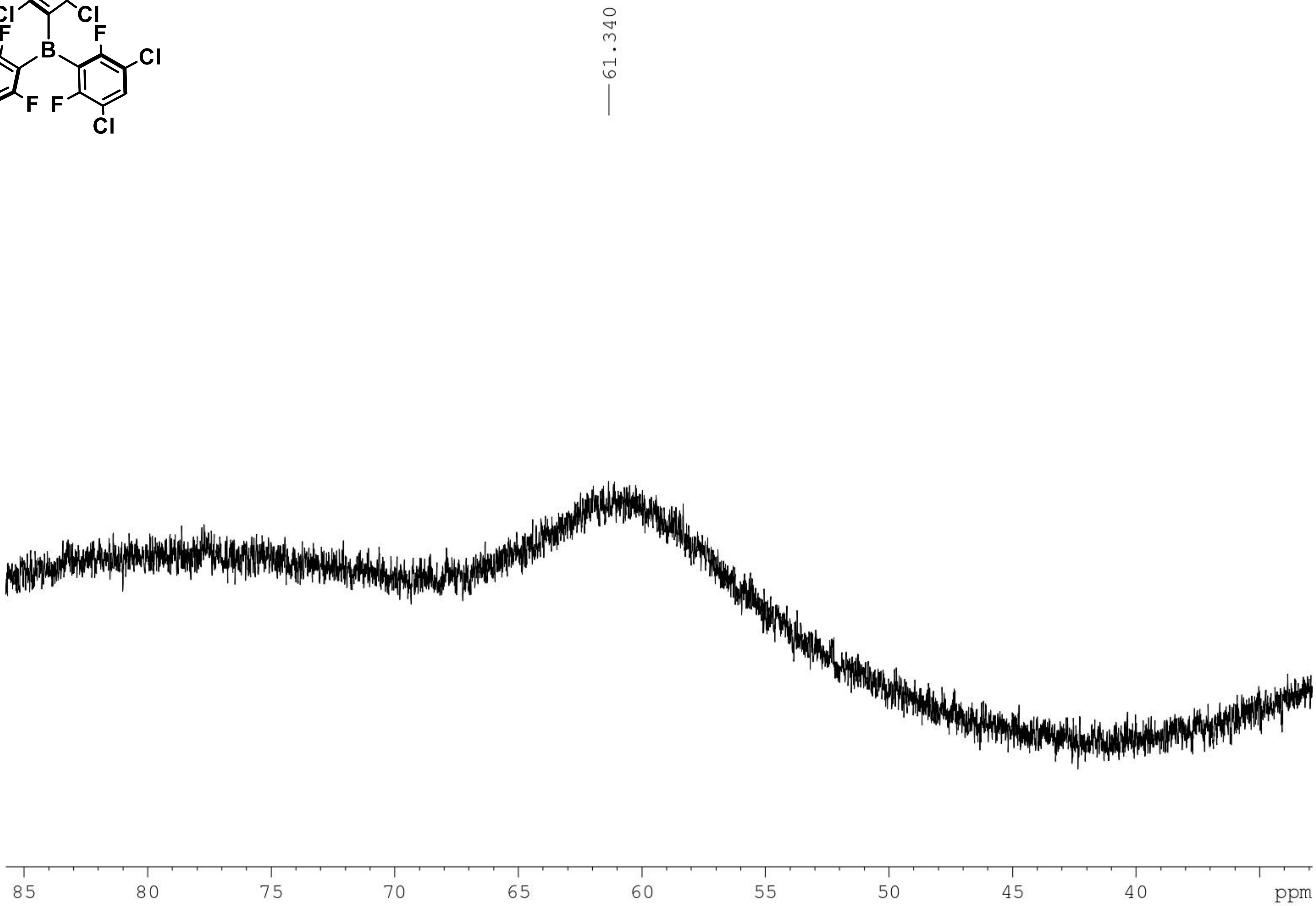

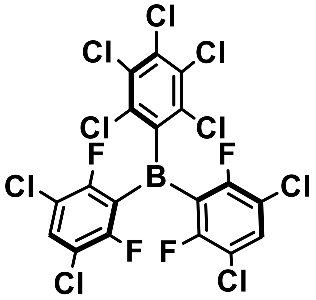

^{13}C, C_6D_6 (100 MHz)

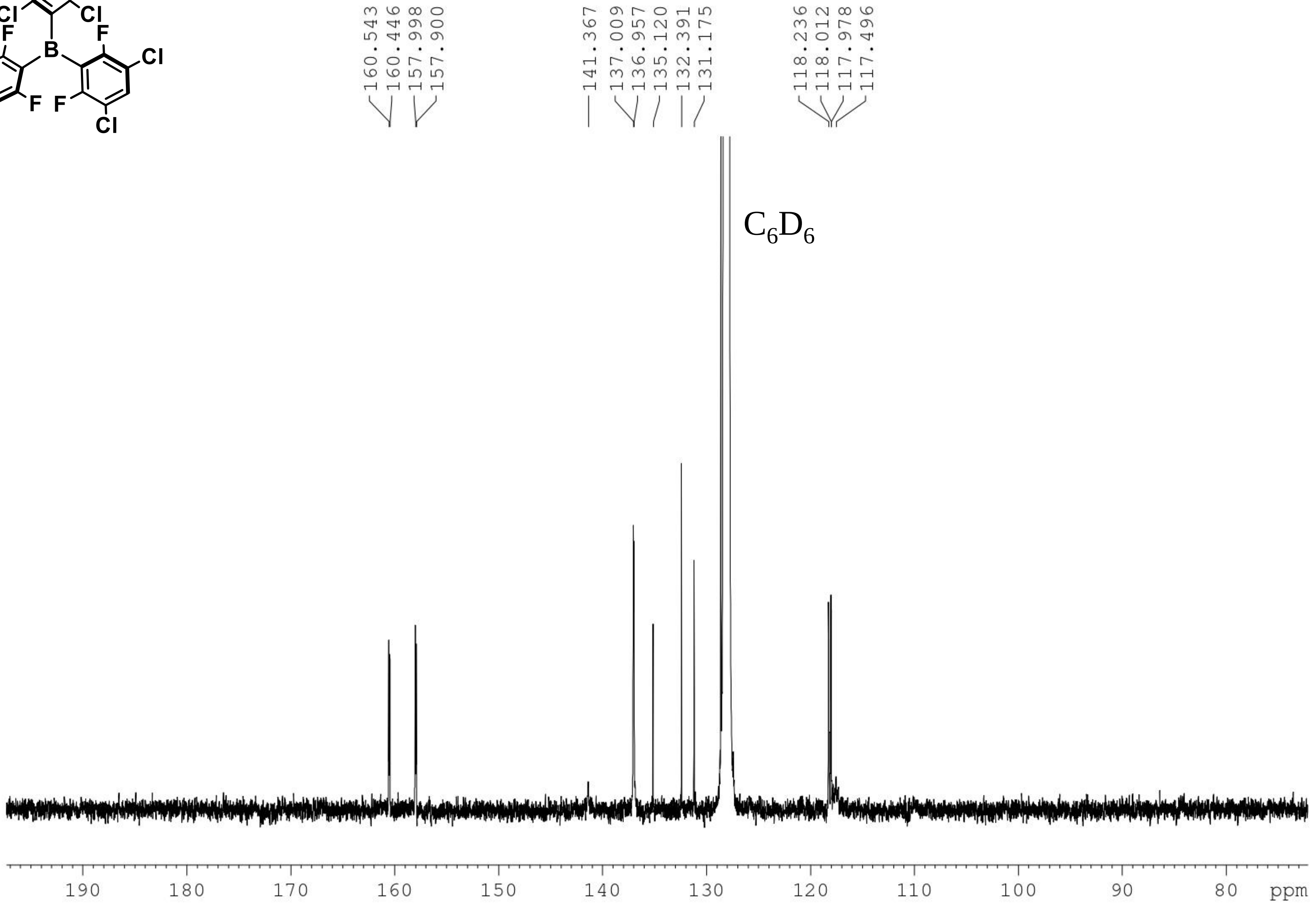

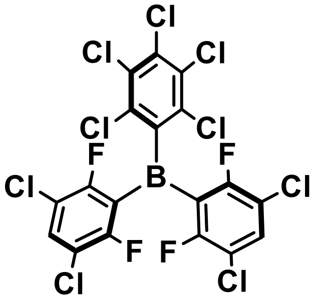

^{19}F, C_6D_6 (376 MHz)

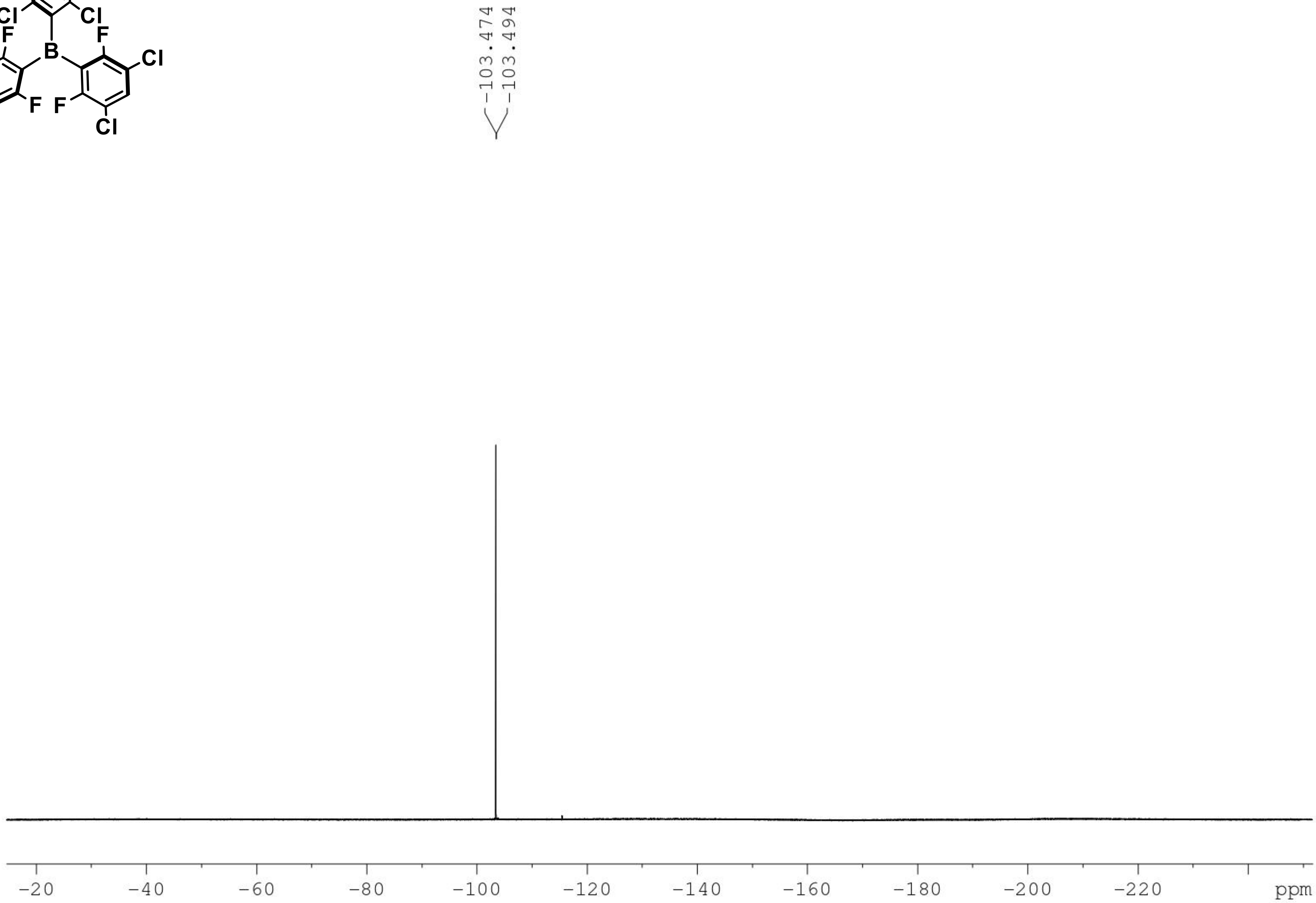

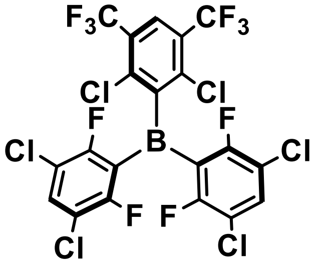

^1H, C_6D_6 (400 MHz)

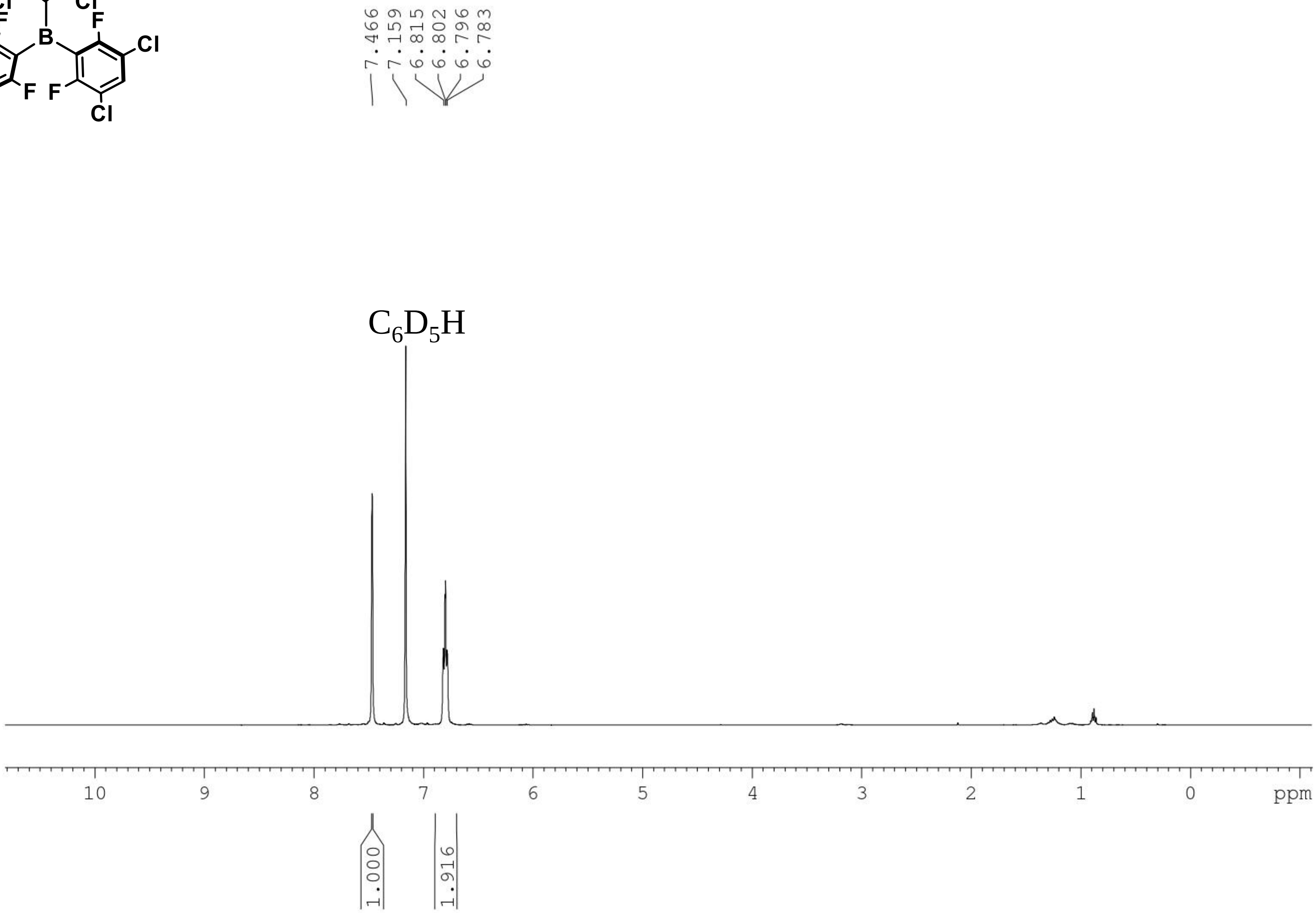

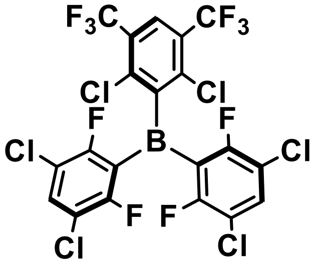

^{11}B, C_6D_6 (128 MHz)

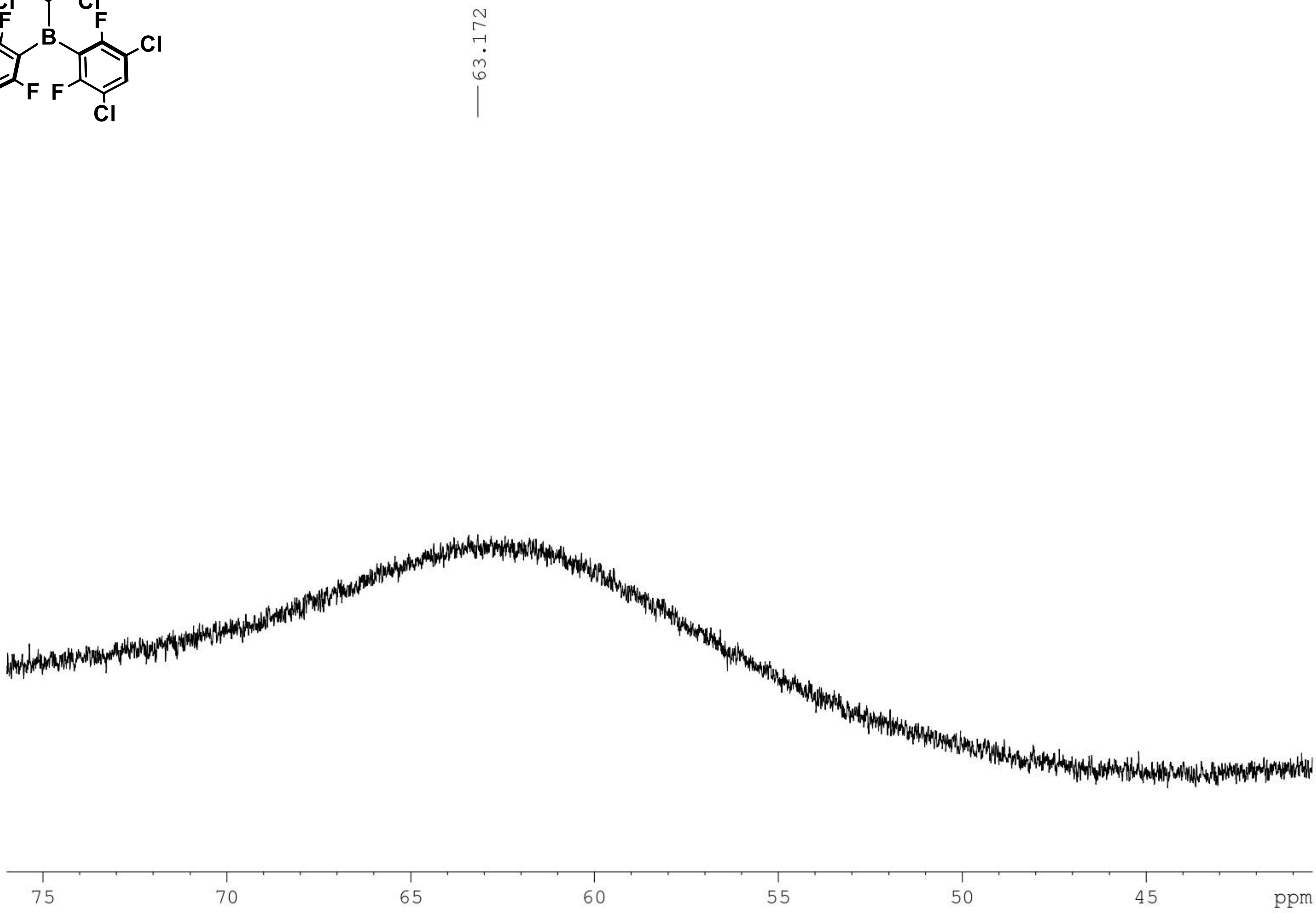

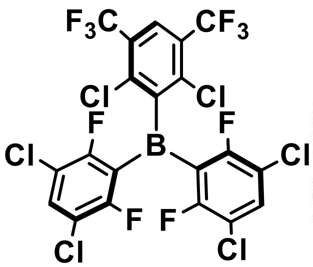

^{13}C, C_6D_6 (100 MHz)

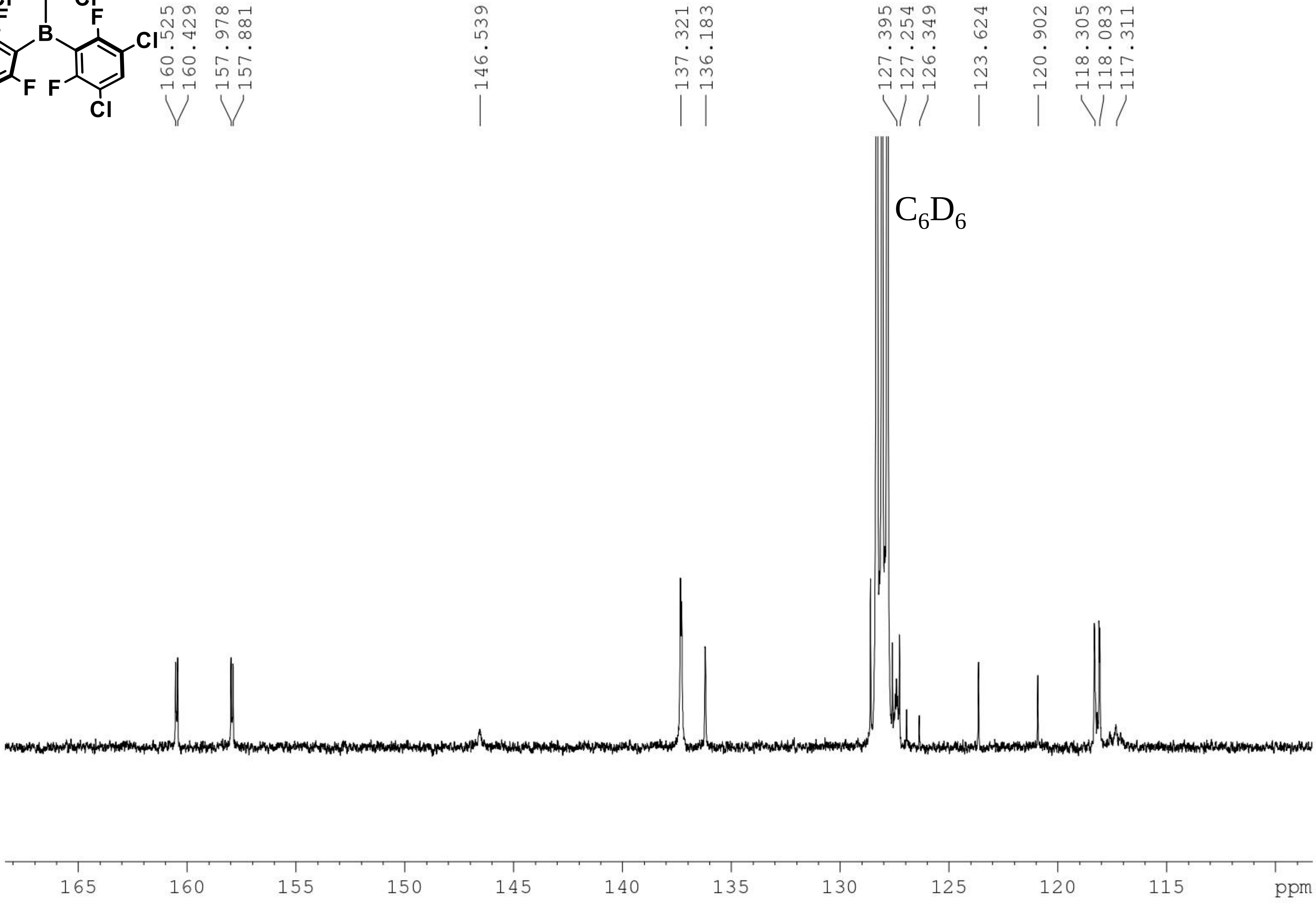

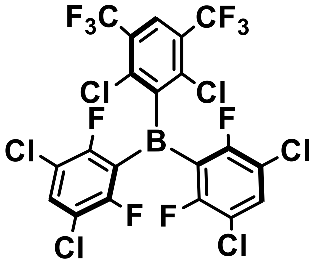

$^{19}F, C_6D_6$ (376 MHz)

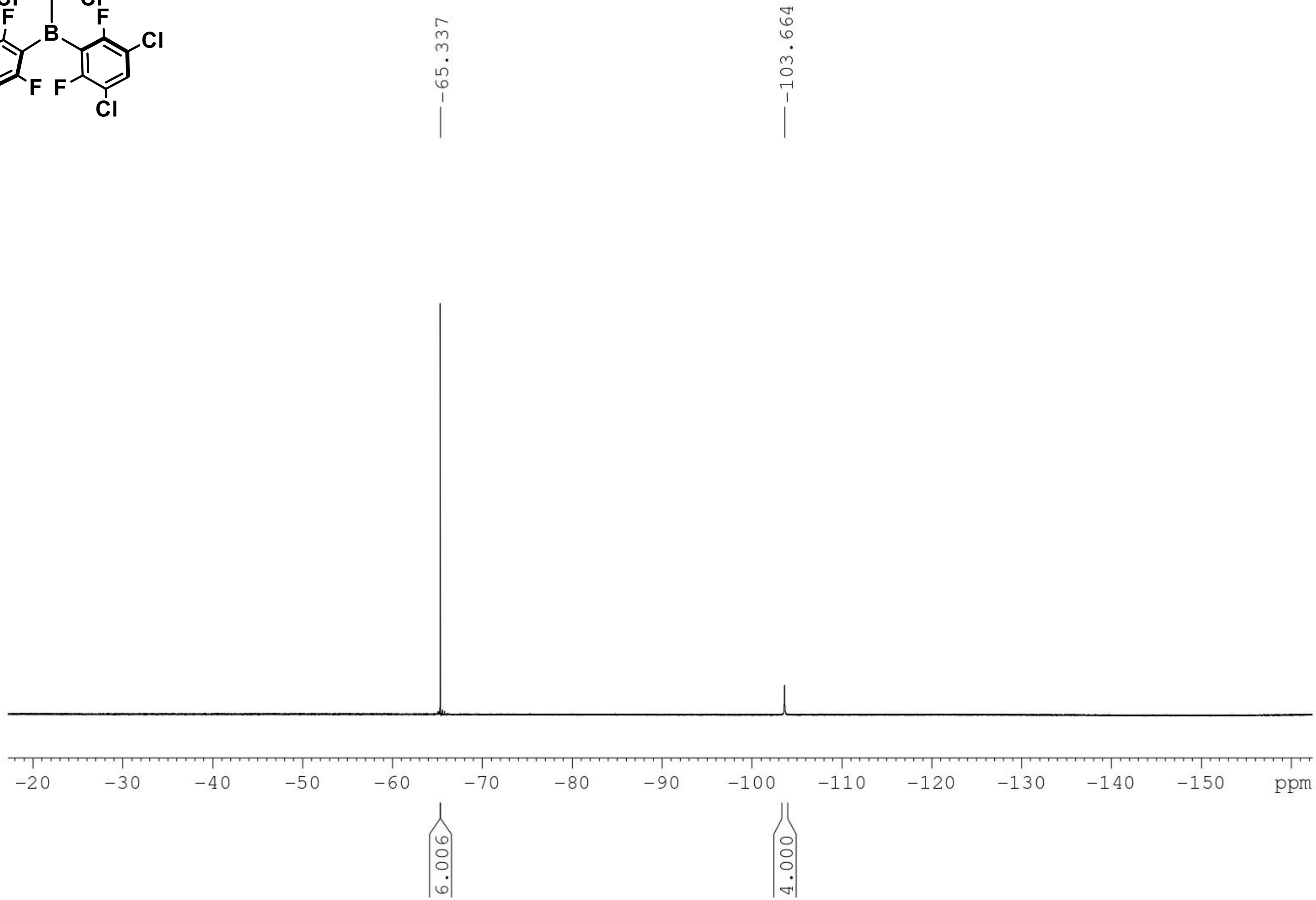

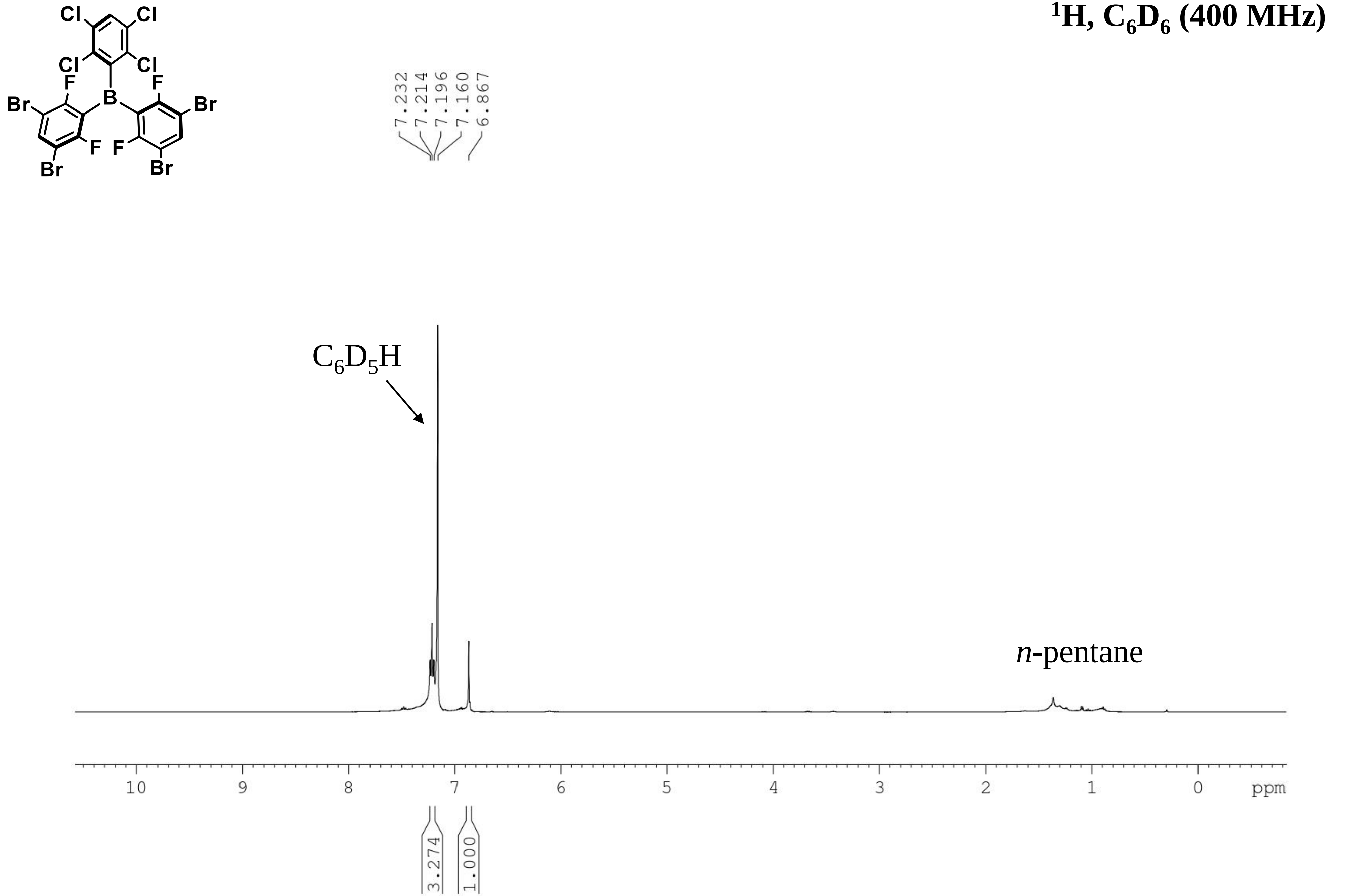

Cl
Cl
Cl
Cl
F
F
Br
B
Br
F
F
Br
Br
¹H, C6D6 (400 MHz)
7.232
7.214
7.196
7.160
6.867
C6D5H
n-pentane
10
9
8
7
6
5
4
3
2
1
0
ppm
3.274
1.000

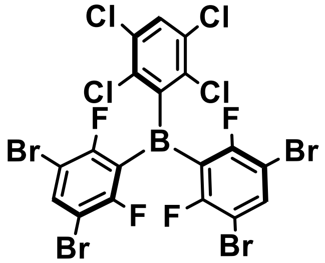

^{11}B, C_6D_6 (128 MHz)

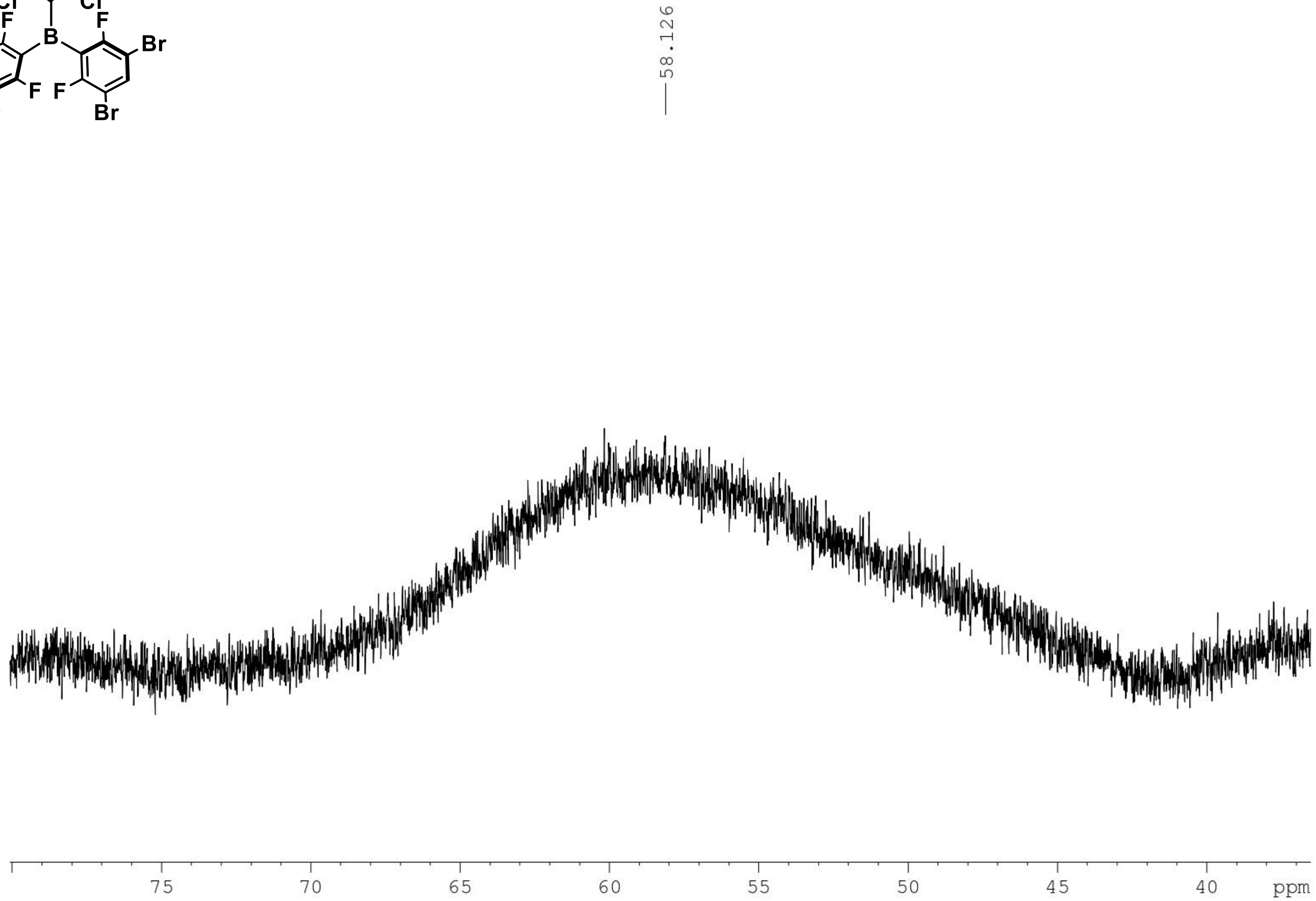

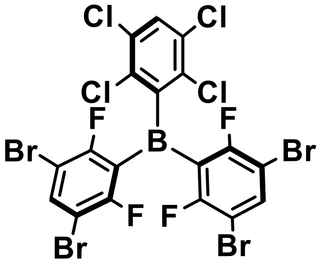

^{13}C, C_6D_6 (100 MHz)

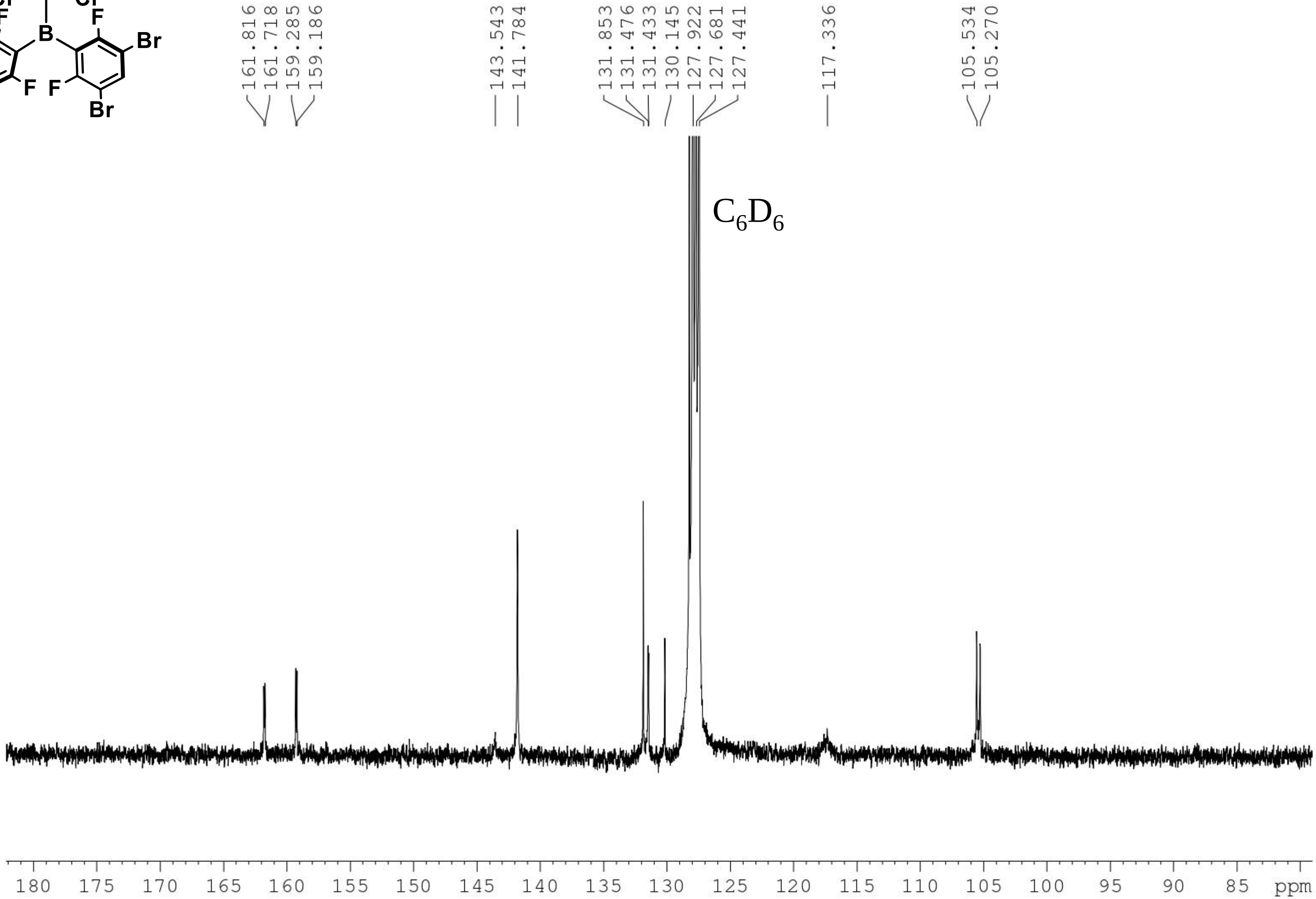

^{19}F, C_6D_6 (376 MHz)

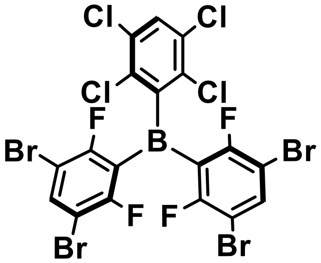

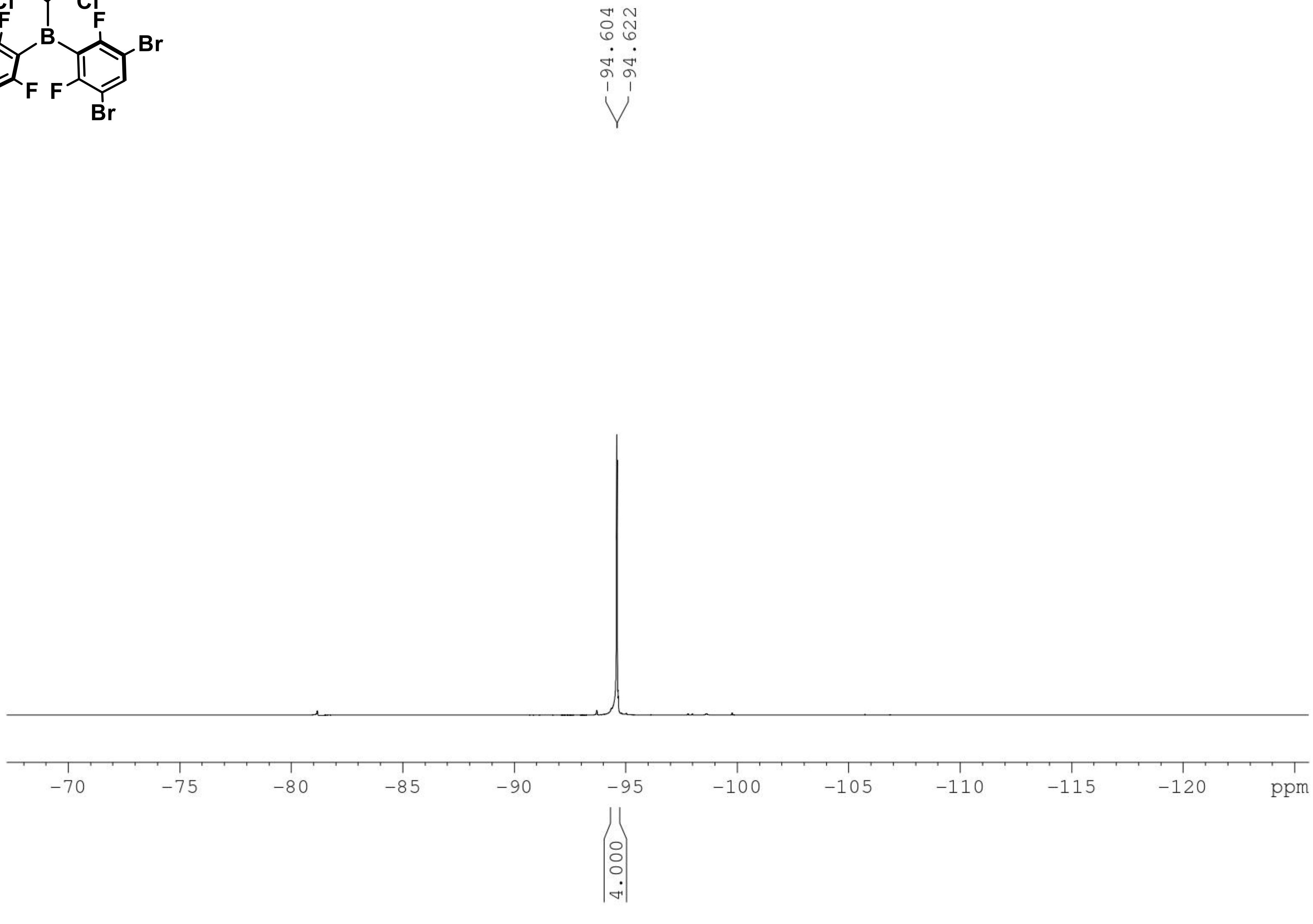

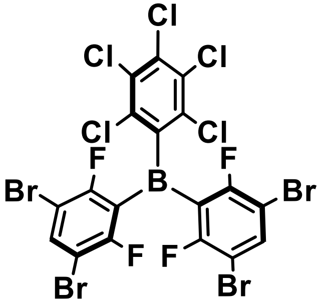

^{1}H, C_6D_6 (400 MHz)

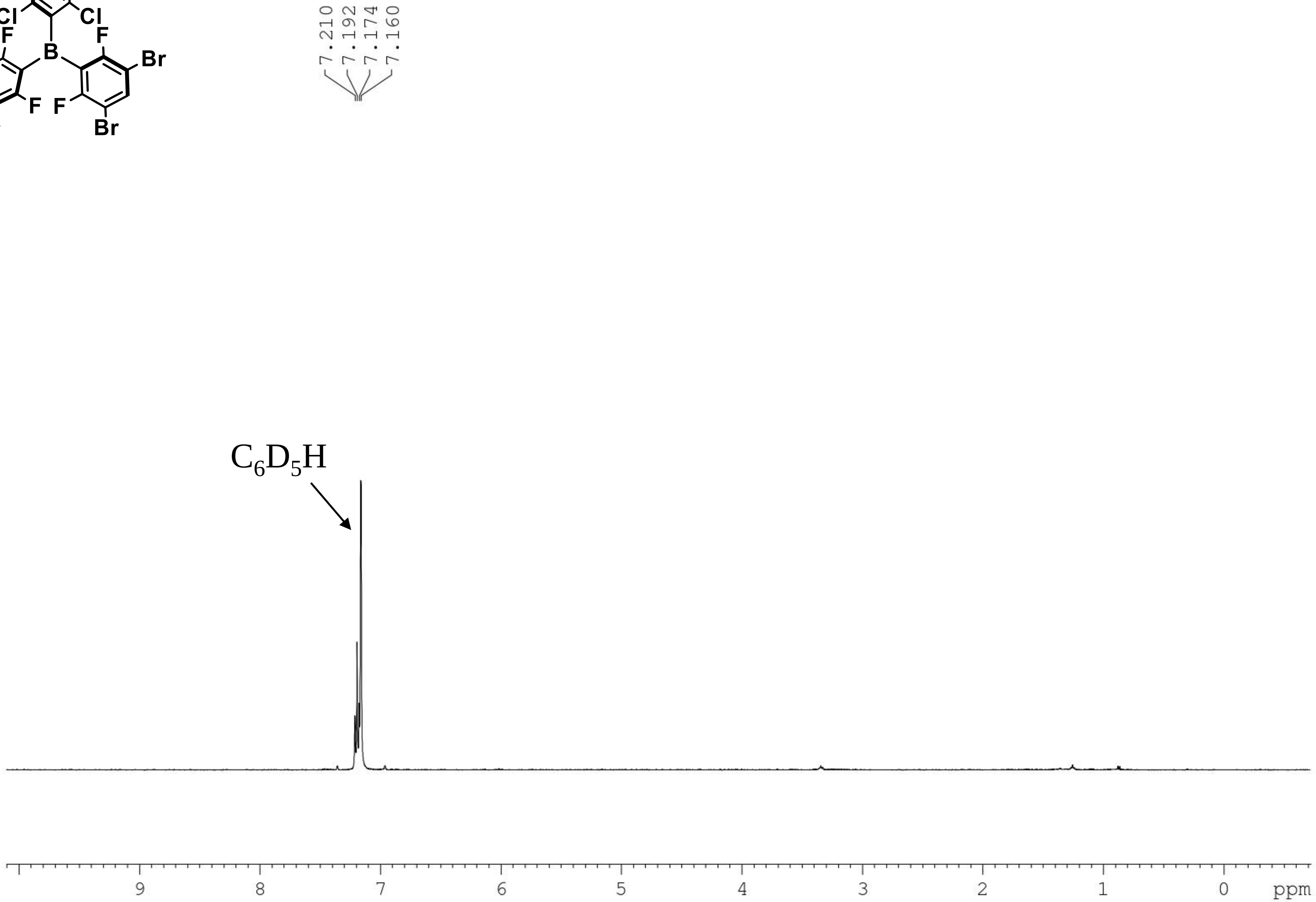

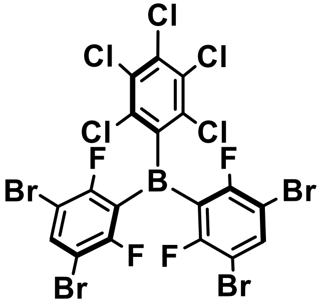

^{11}B, C_6D_6 (128 MHz)

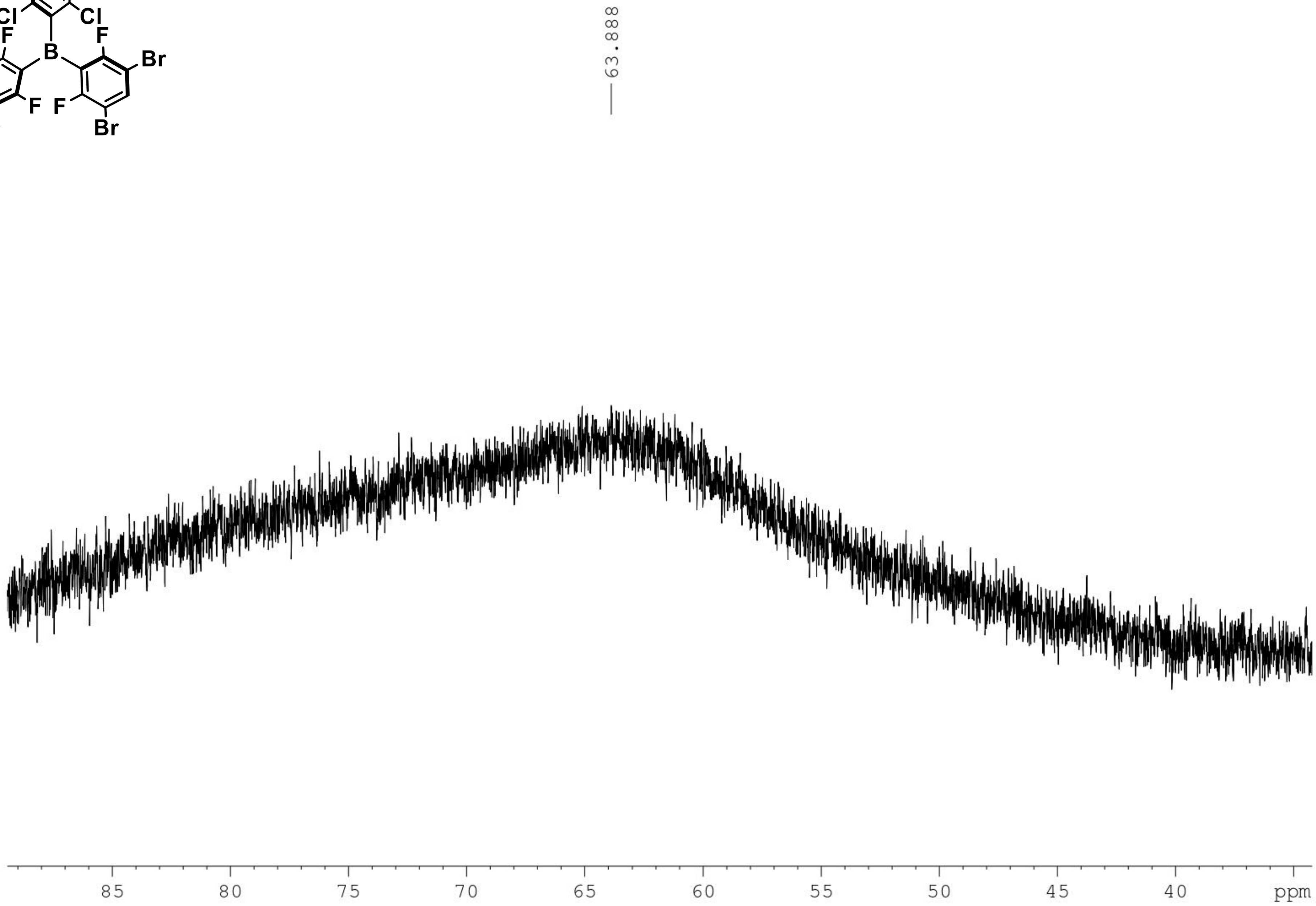

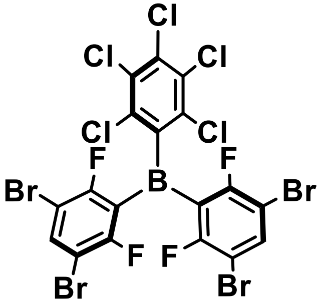

^{13}C, C_6D_6 (100 MHz)

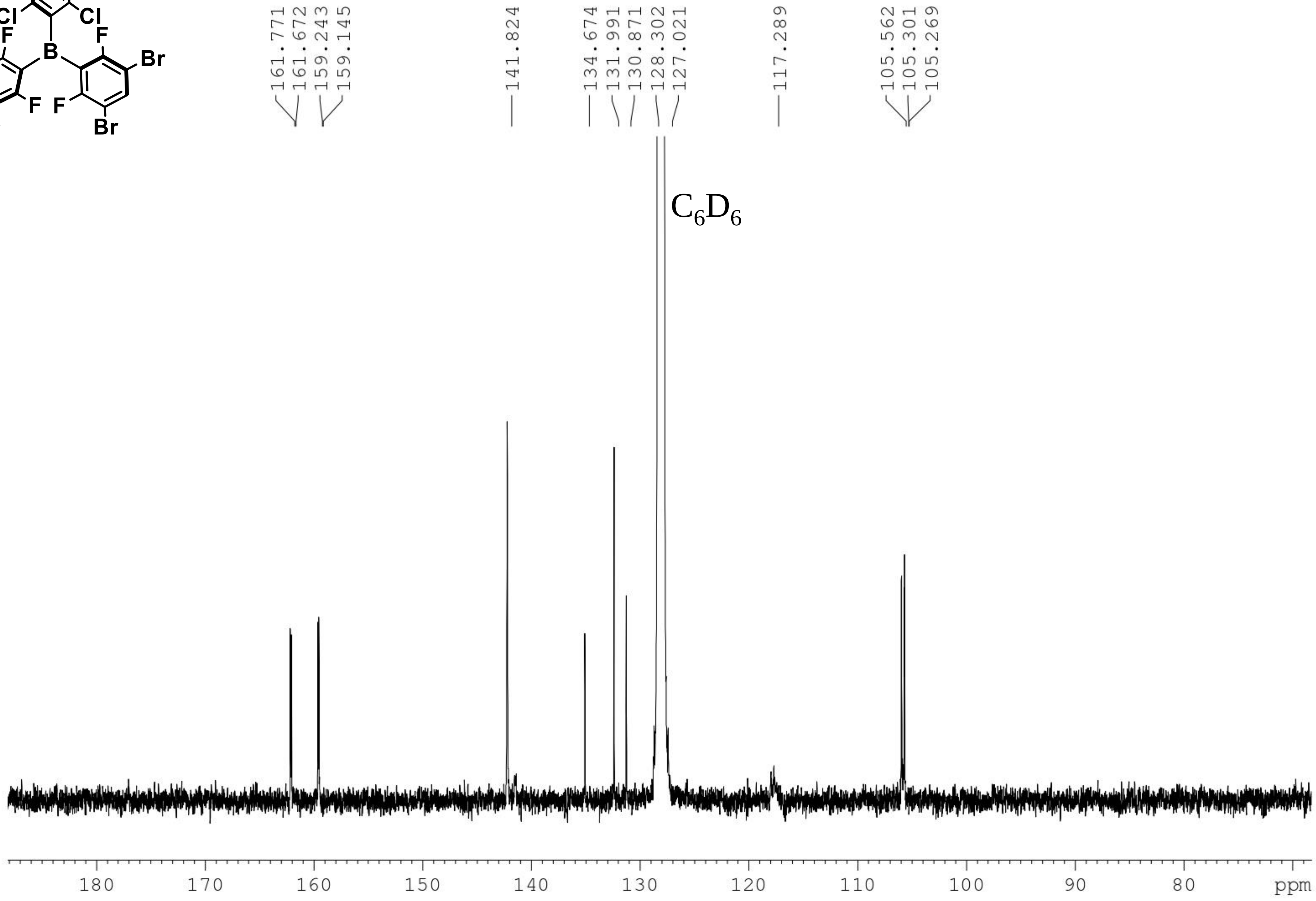

^{19}F, C_6D_6 (376 MHz)

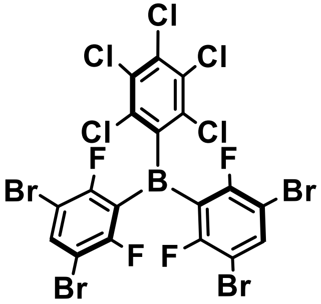

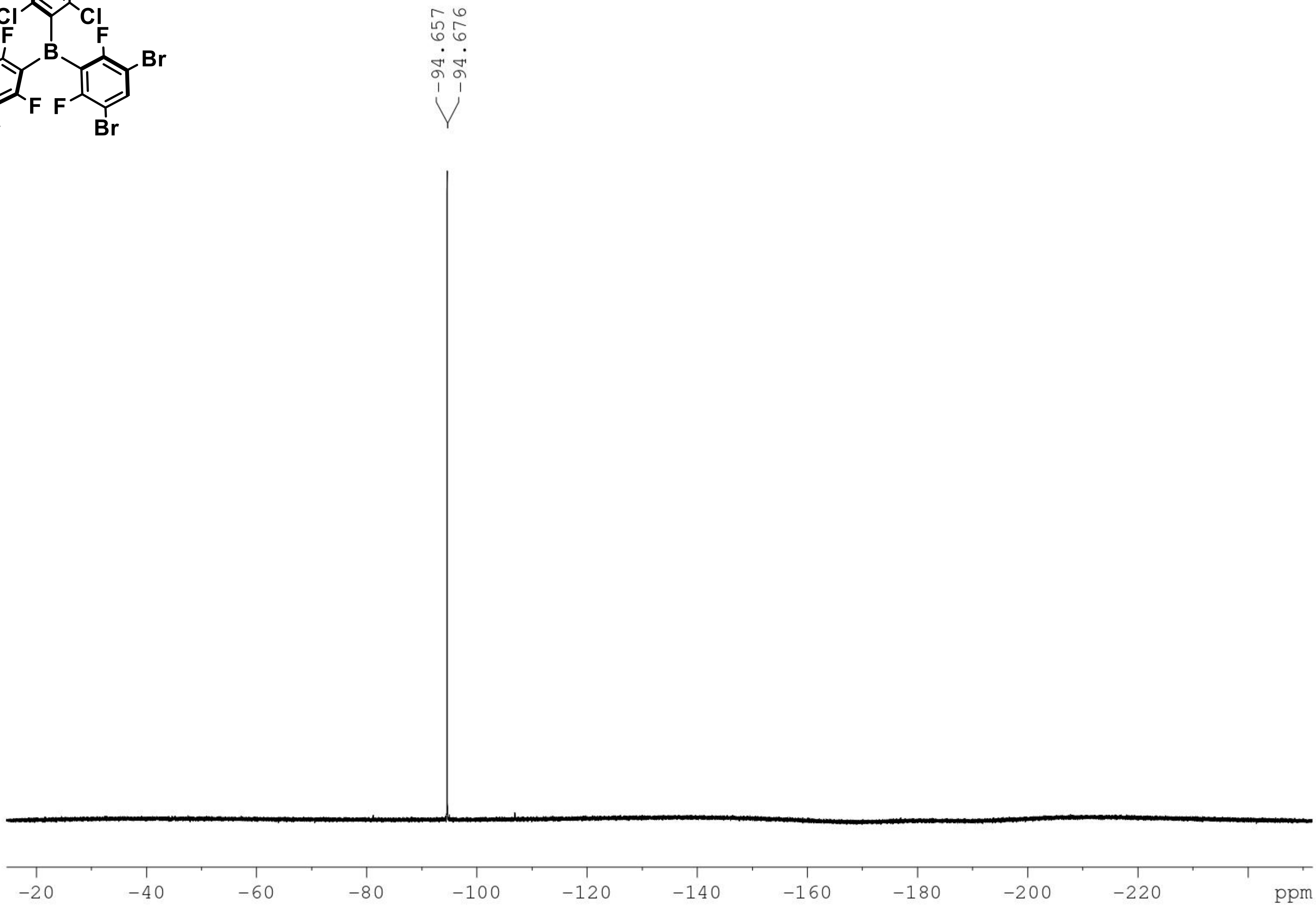

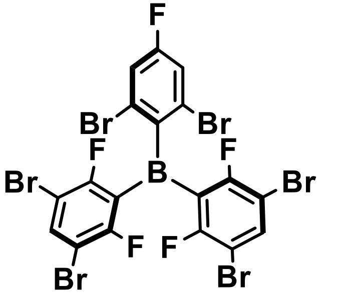

^{1}H, C_6D_6 (400 MHz)

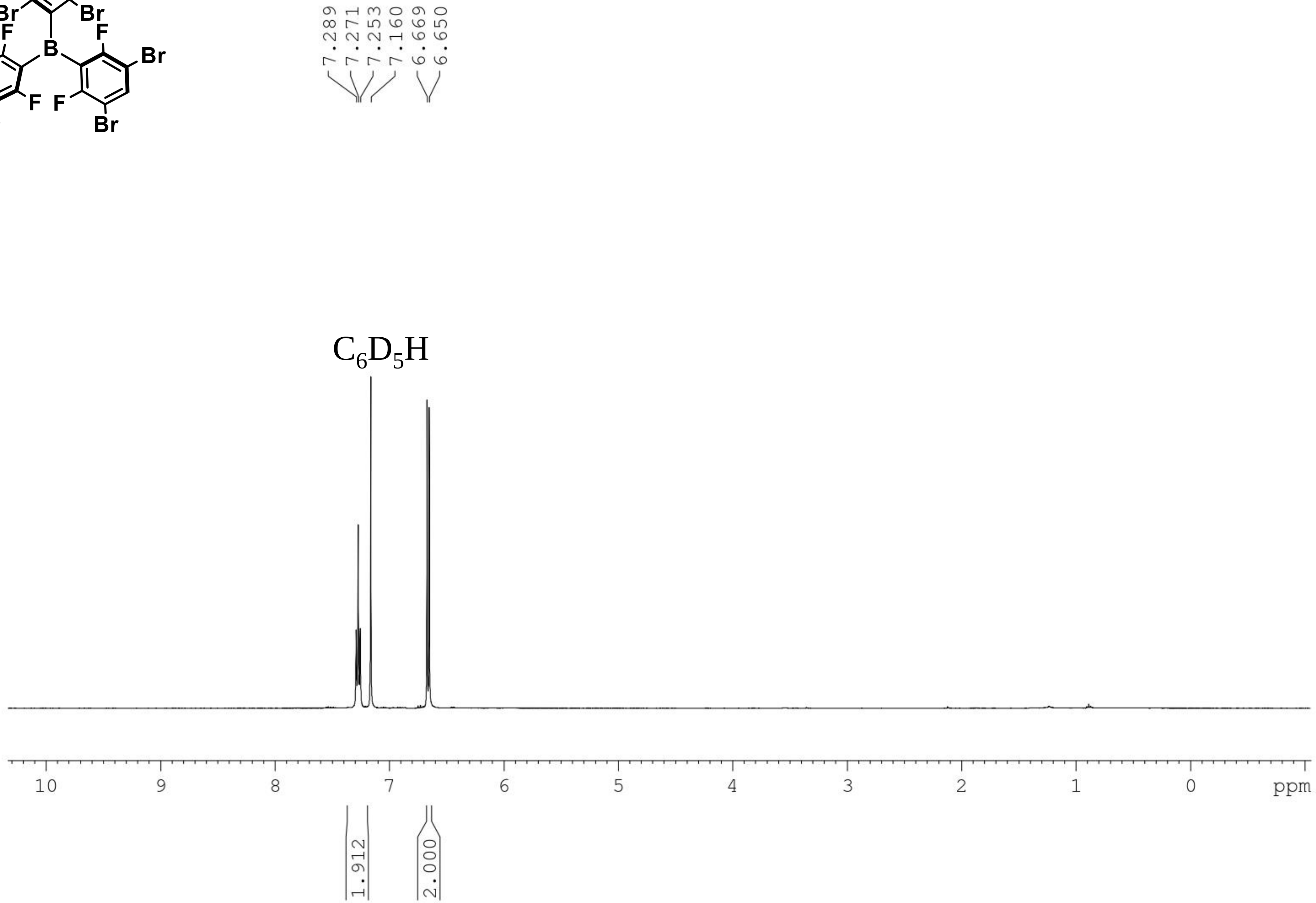

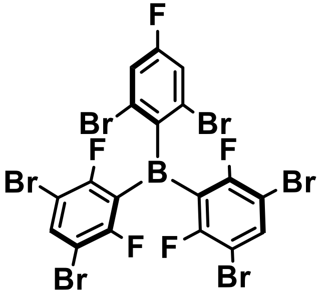

^{11}B, C_6D_6 (128 MHz)

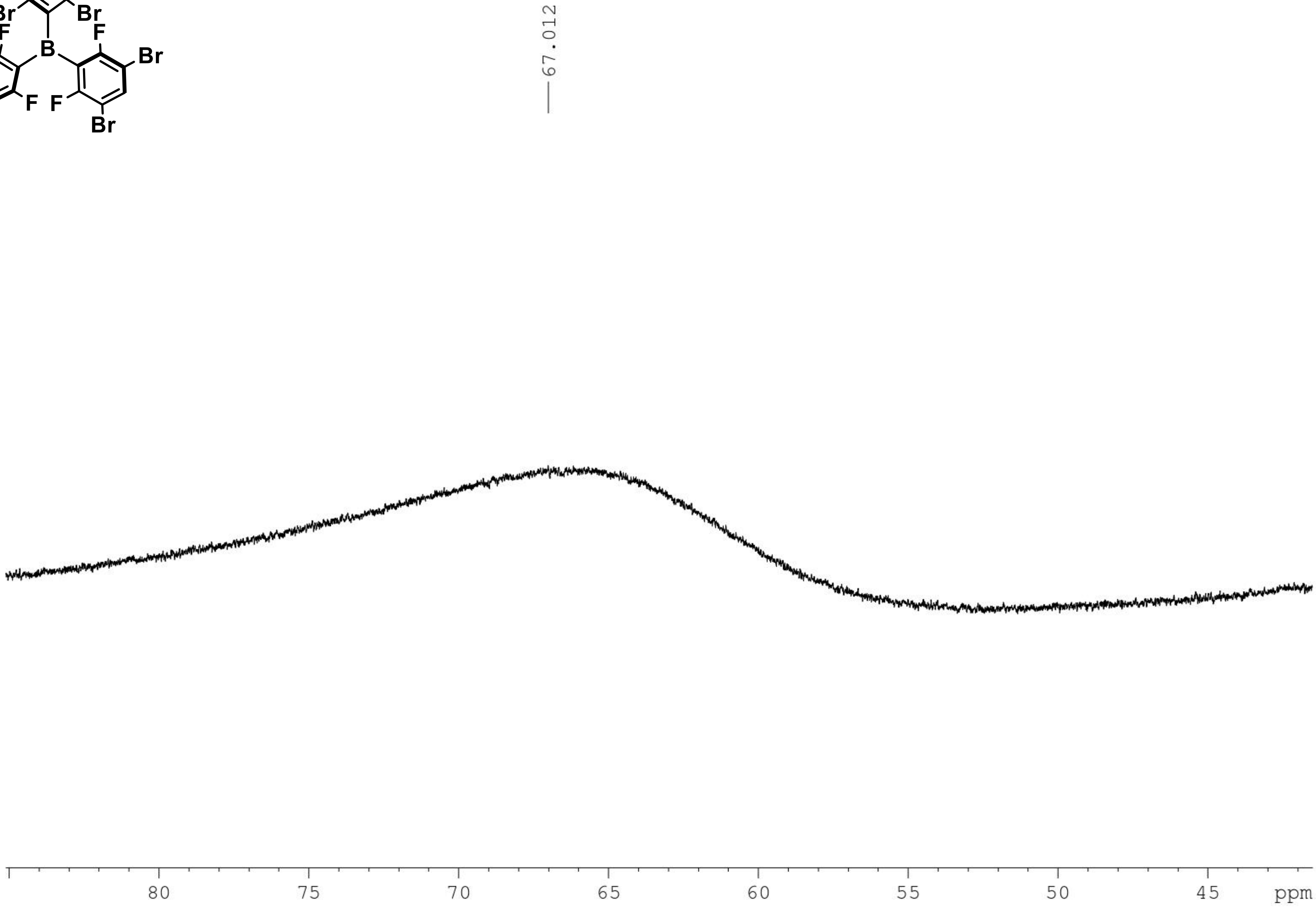

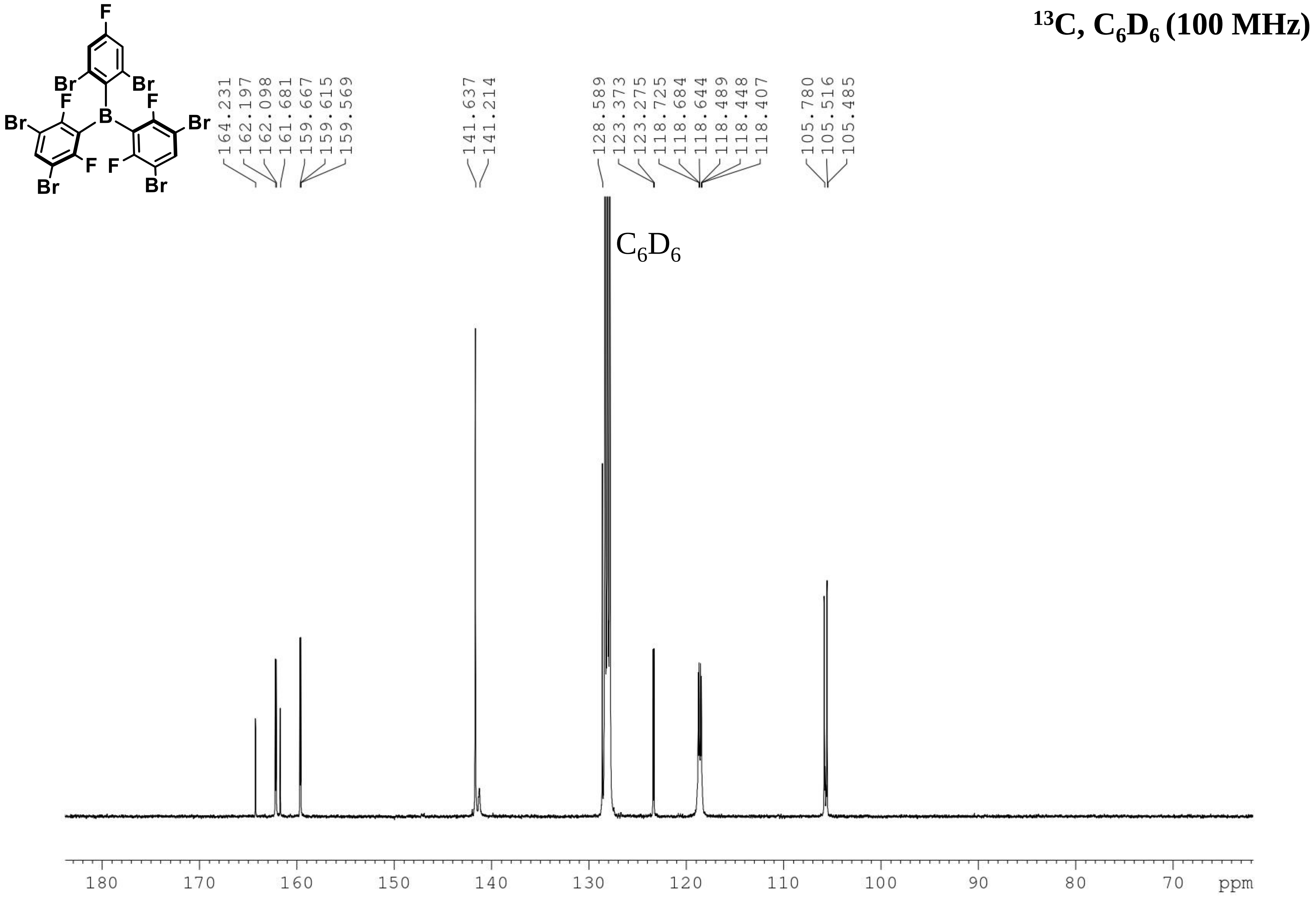
13C, C6D6 (100 MHz)
F
Br
Br
Br
F
B
F
Br
Br
F
F
Br
Br
Br
164.231
162.197
162.098
161.681
159.667
159.615
159.569
141.637
141.214
128.589
123.373
123.275
118.725
118.684
118.644
118.489
118.448
118.407
105.780
105.516
105.485
C6D6
180
170
160
150
140
130
120
110
100
90
80
70
ppm

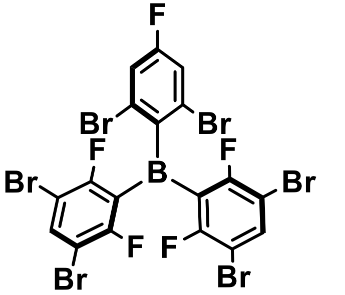

^{19}F, C_6D_6 (376 MHz)

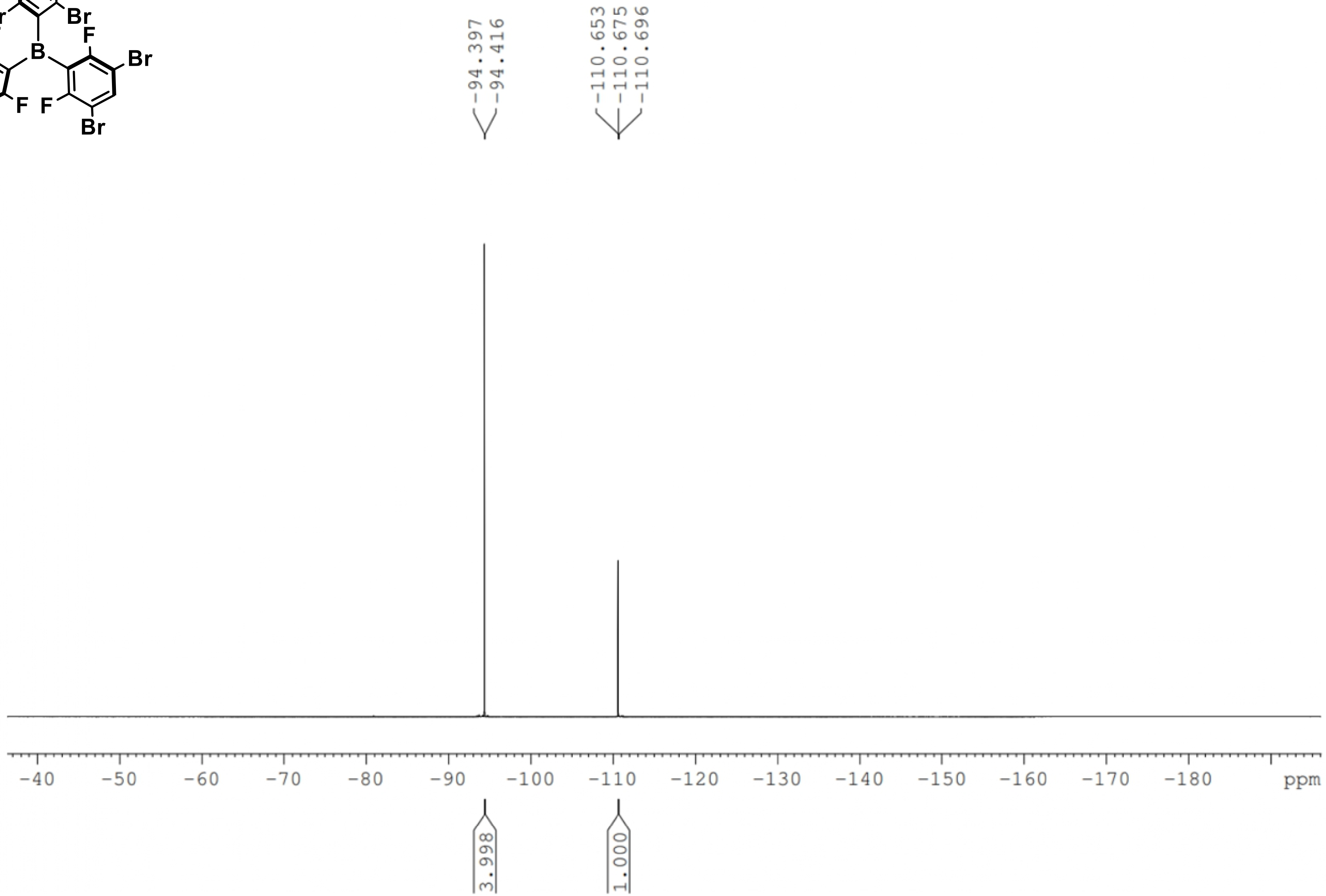

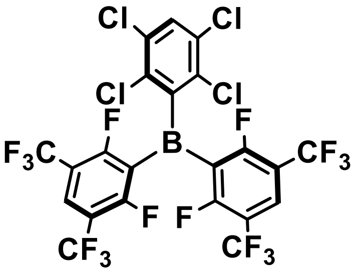

^1H, C_6D_6 (400 MHz)

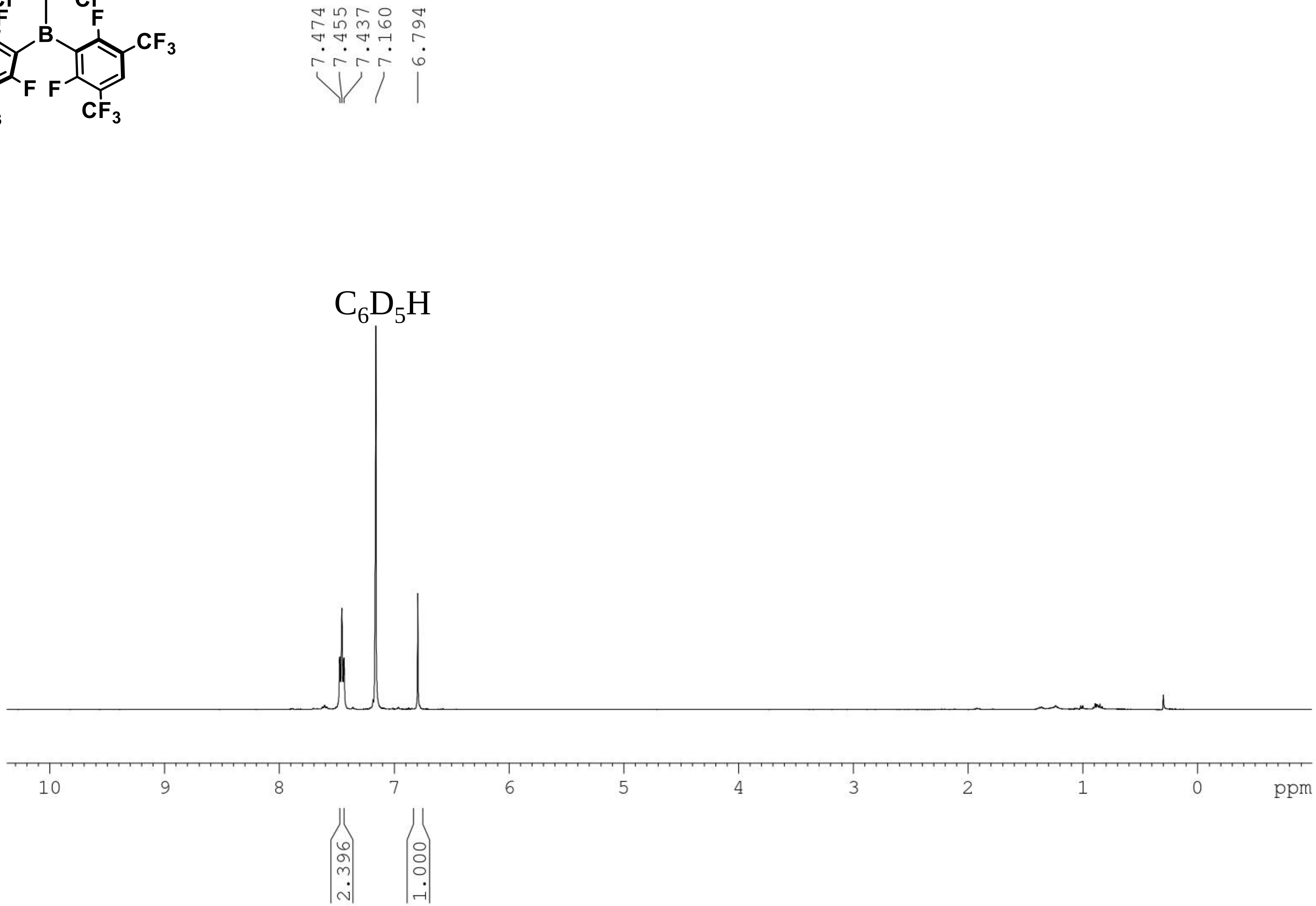

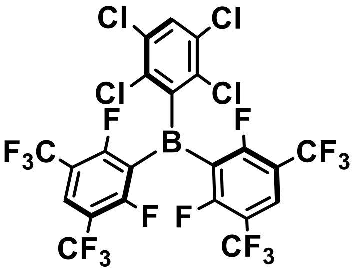

^{11}B, C_6D_6 (128 MHz)

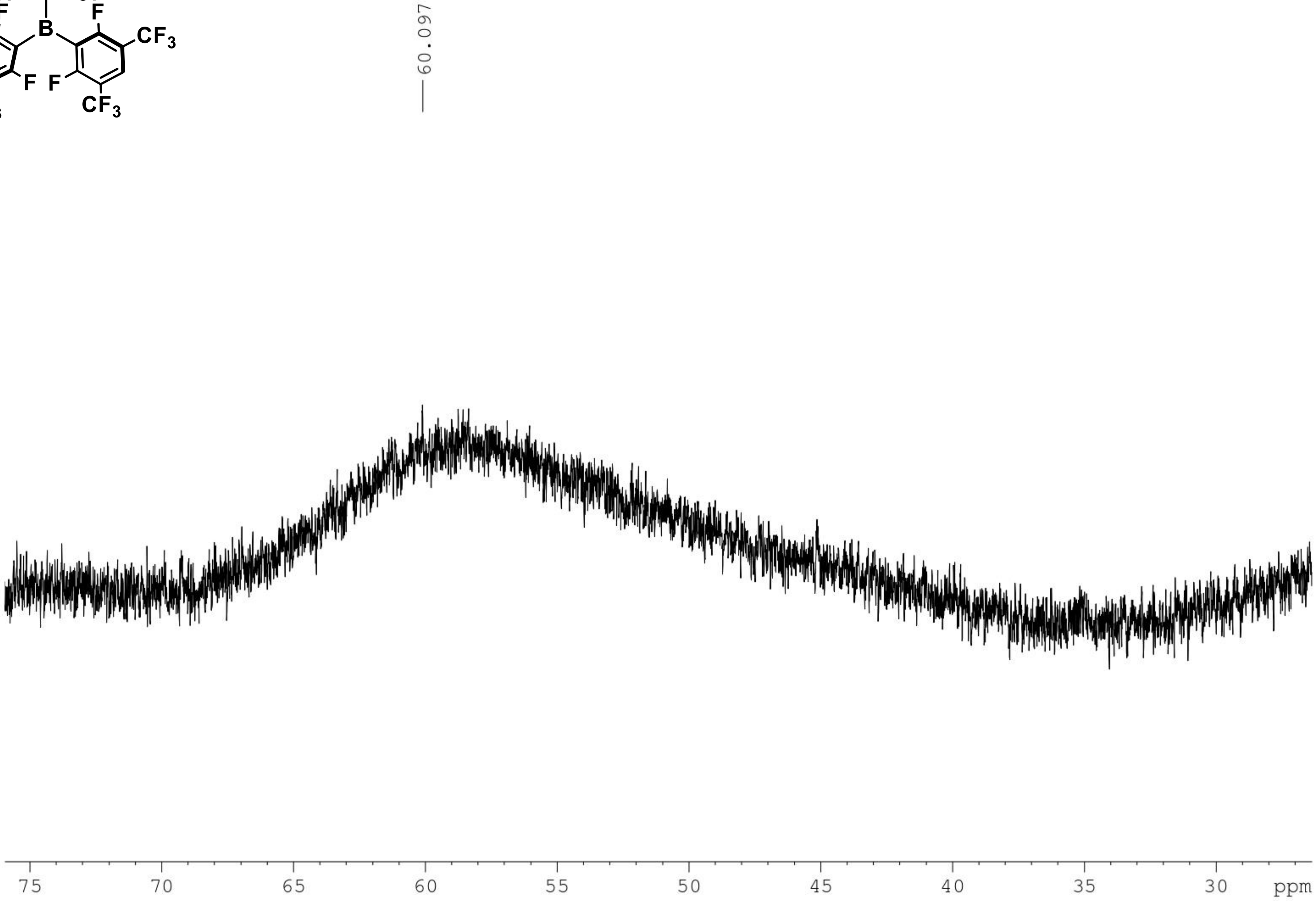

^{13}C, C_6D_6 (100 MHz)

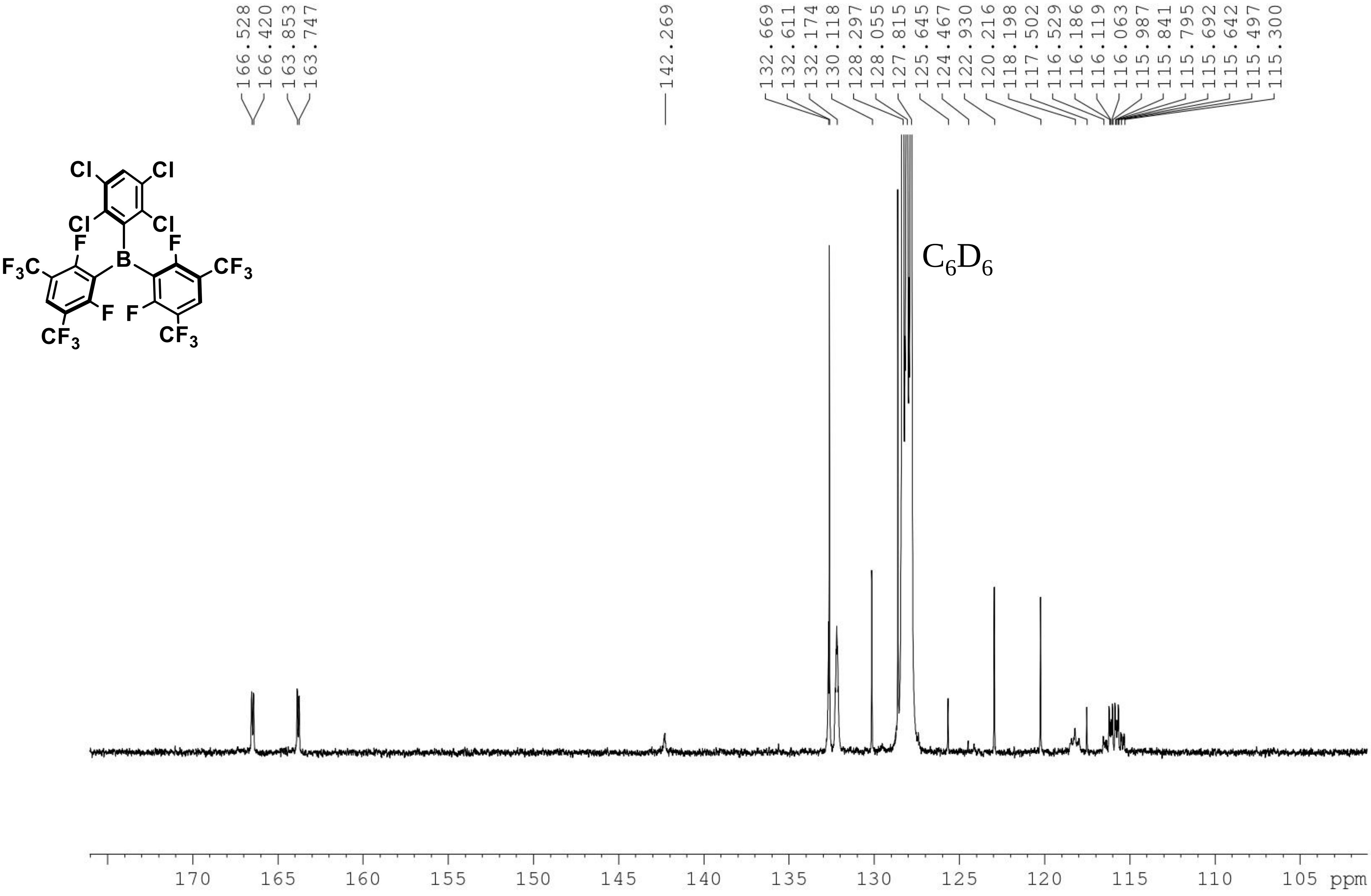

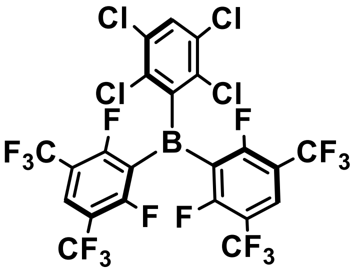

^{19}F, C_6D_6 (376 MHz)

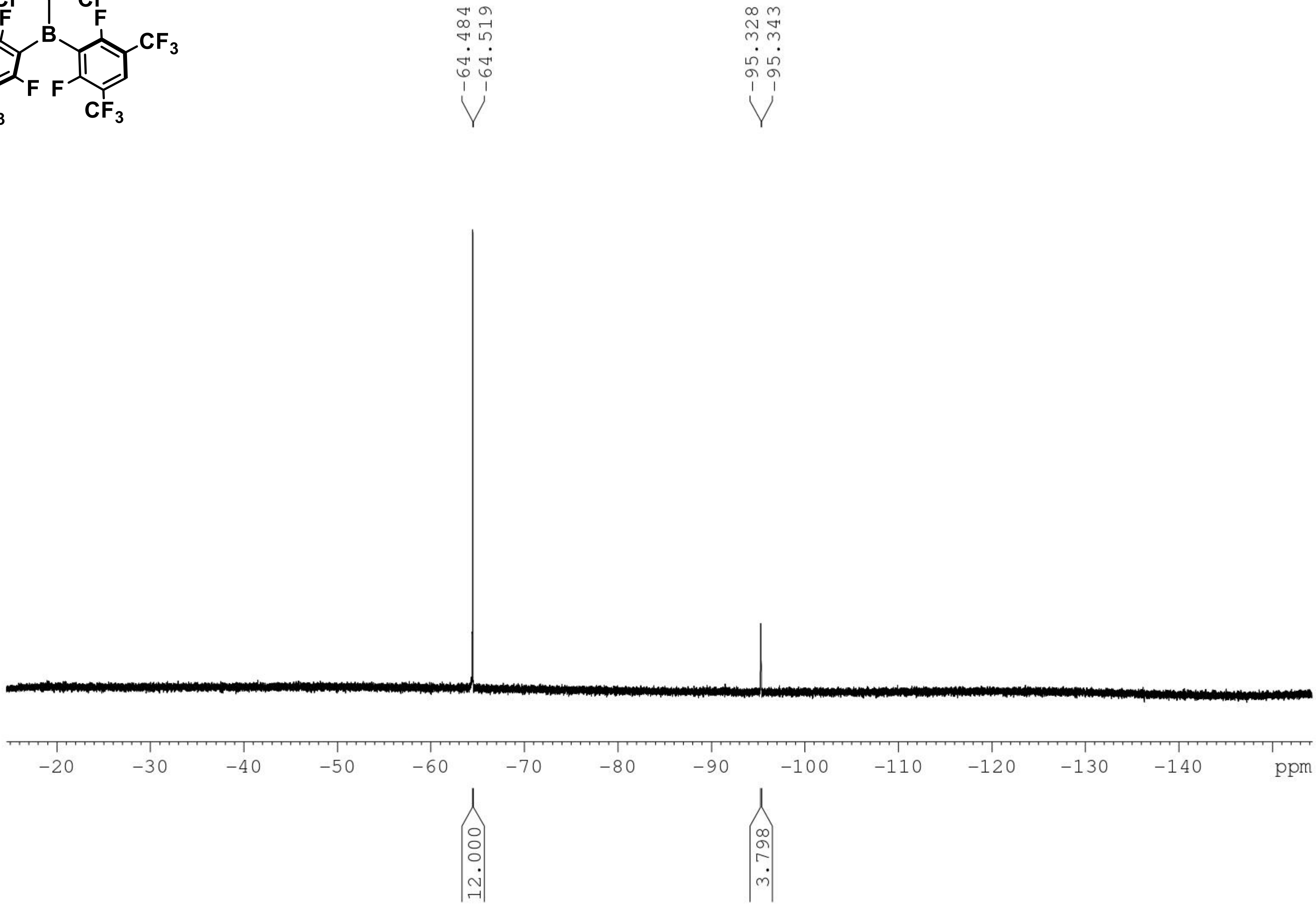

^{1}H, C_6D_6 (400 MHz)

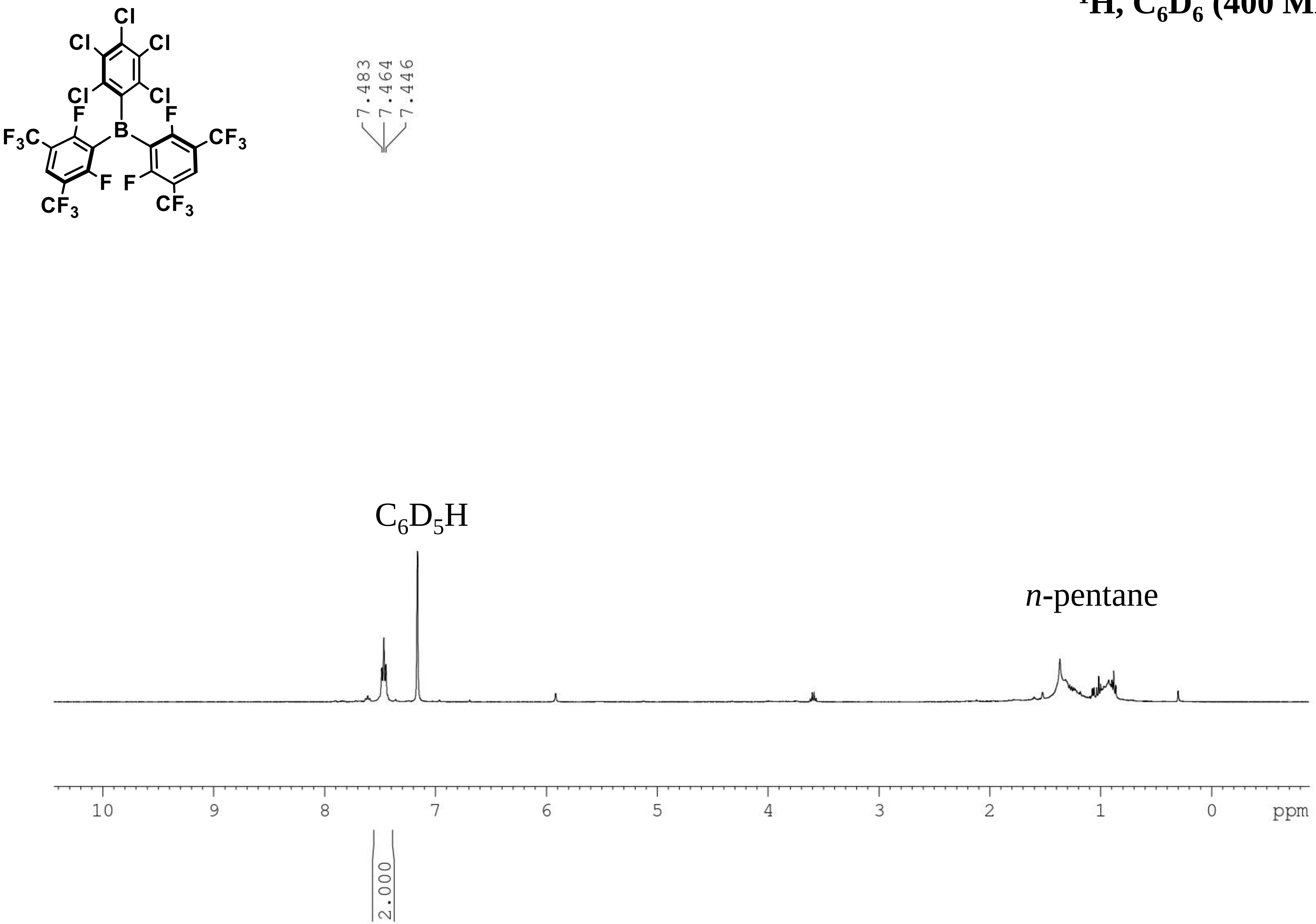

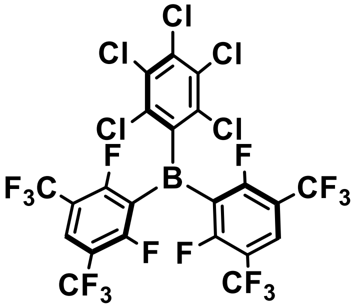

^{11}B, C_6D_6 (128 MHz)

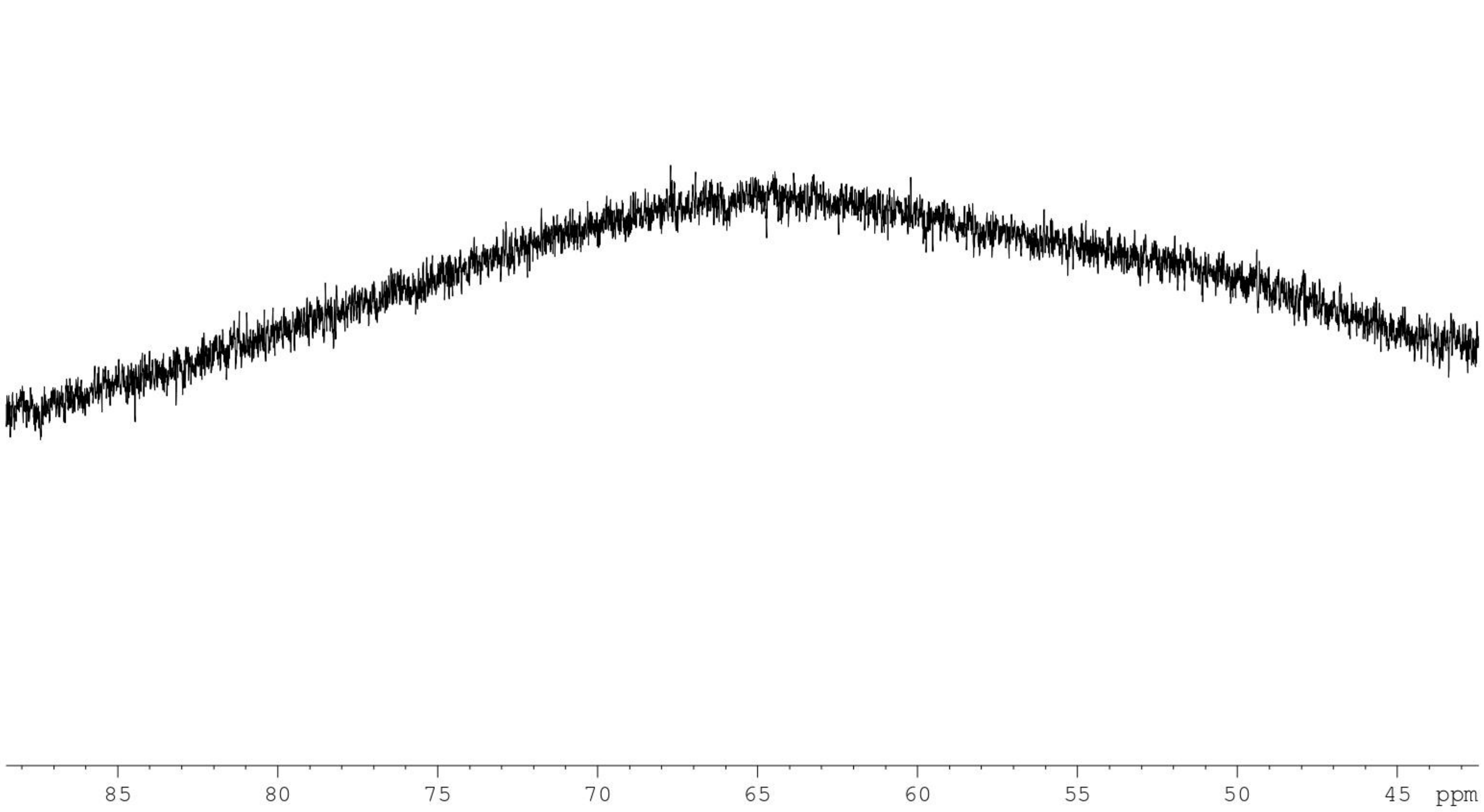

^{13}C, C_6D_6 (100 MHz)

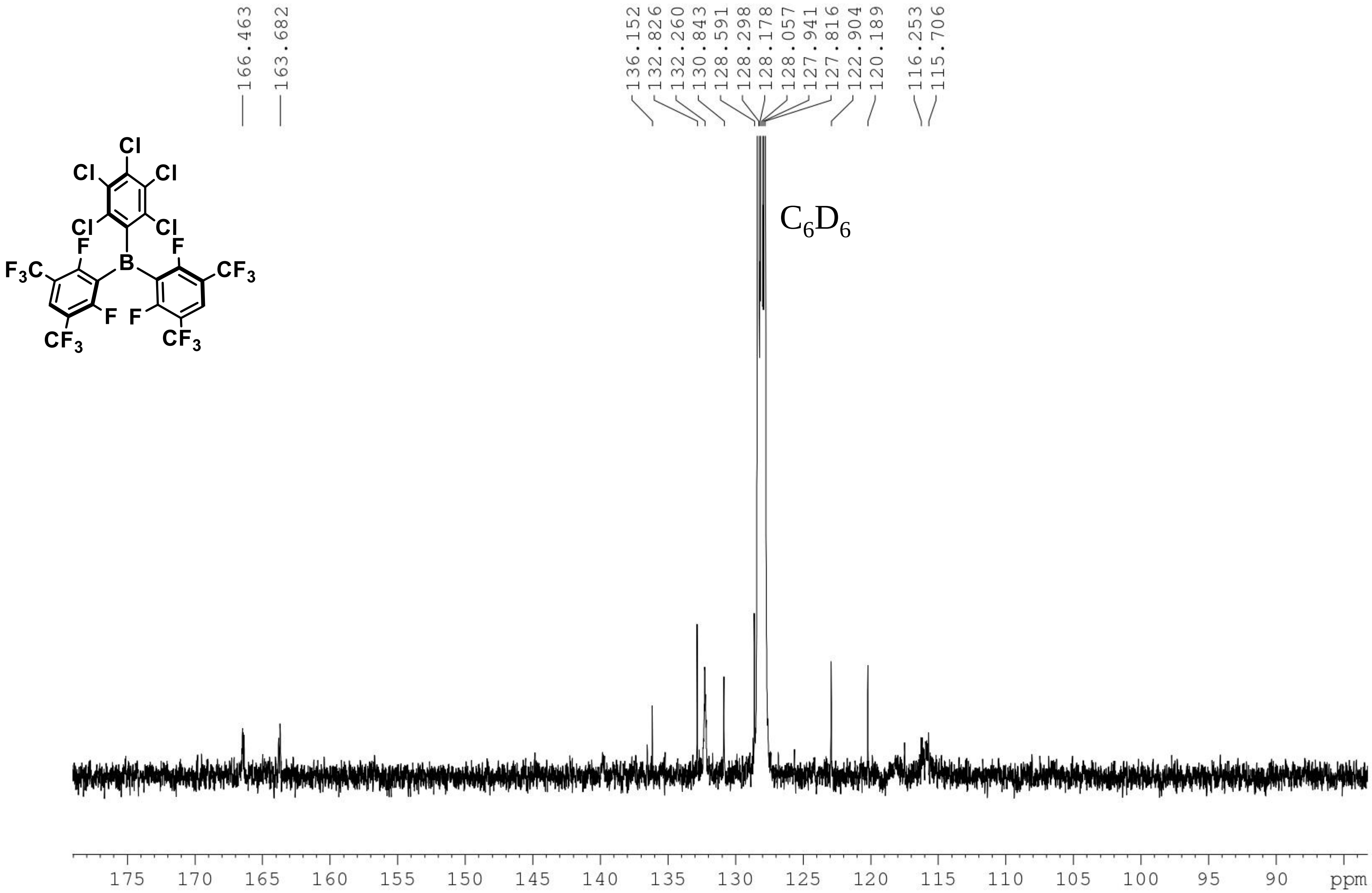

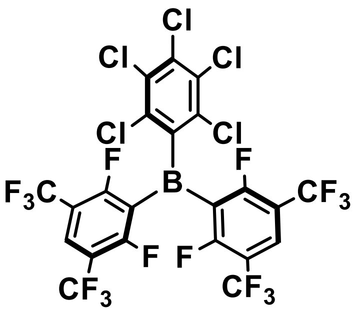

^{19}F, C_6D_6 (376 MHz)

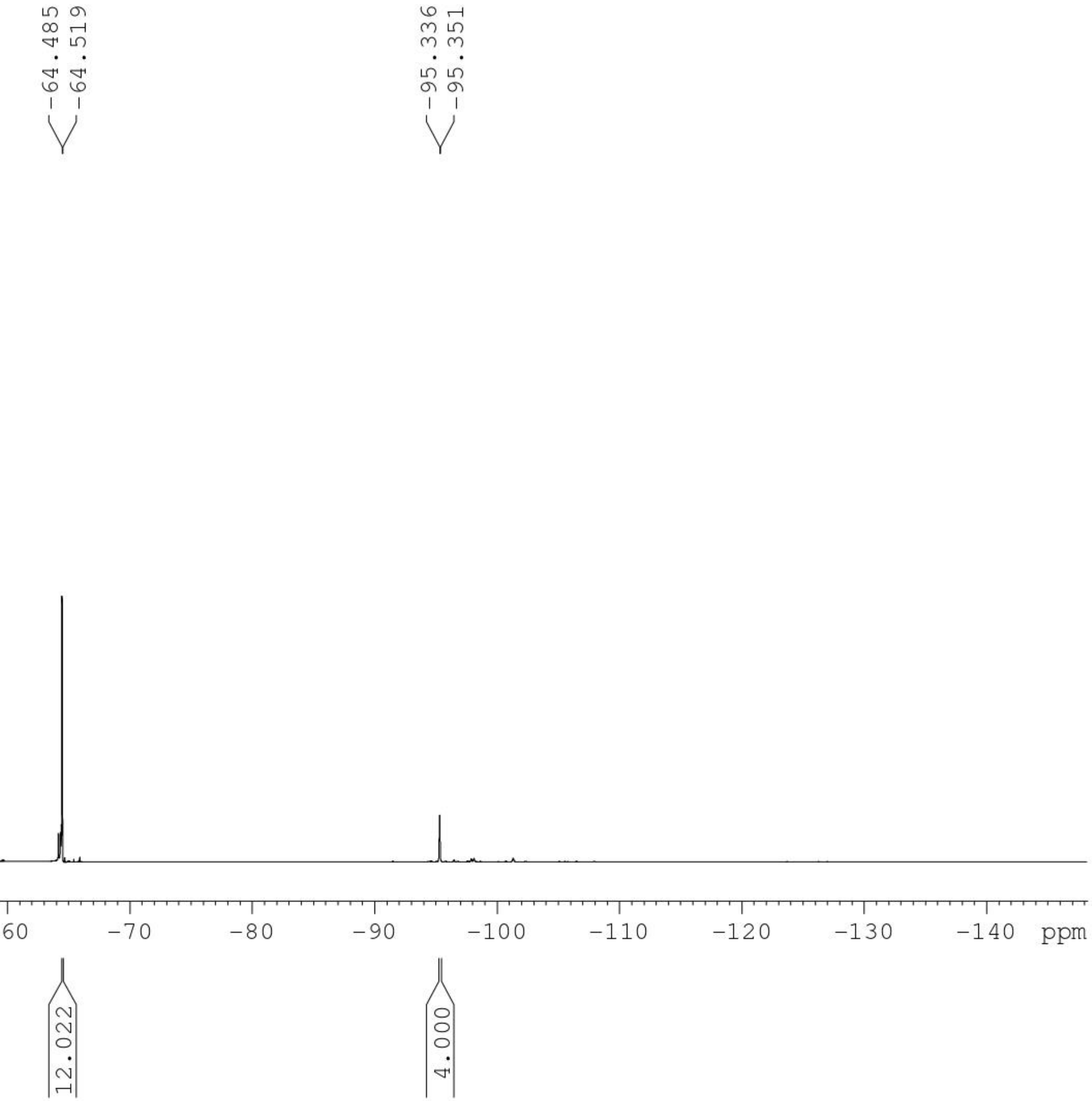

^1H, $CDCl_3$ (400 MHz)

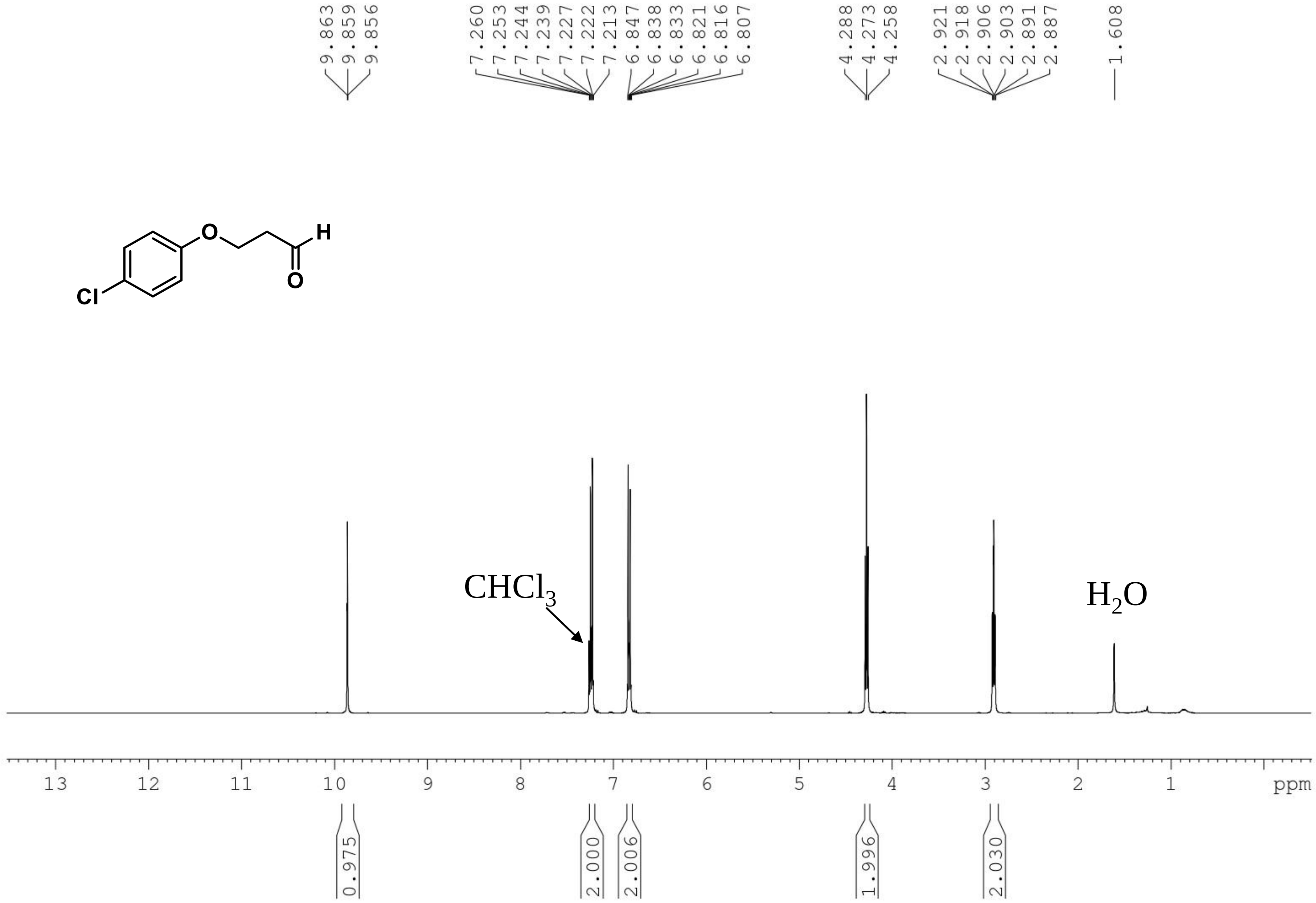

^{13}C, $CDCl_3$ (100 MHz)

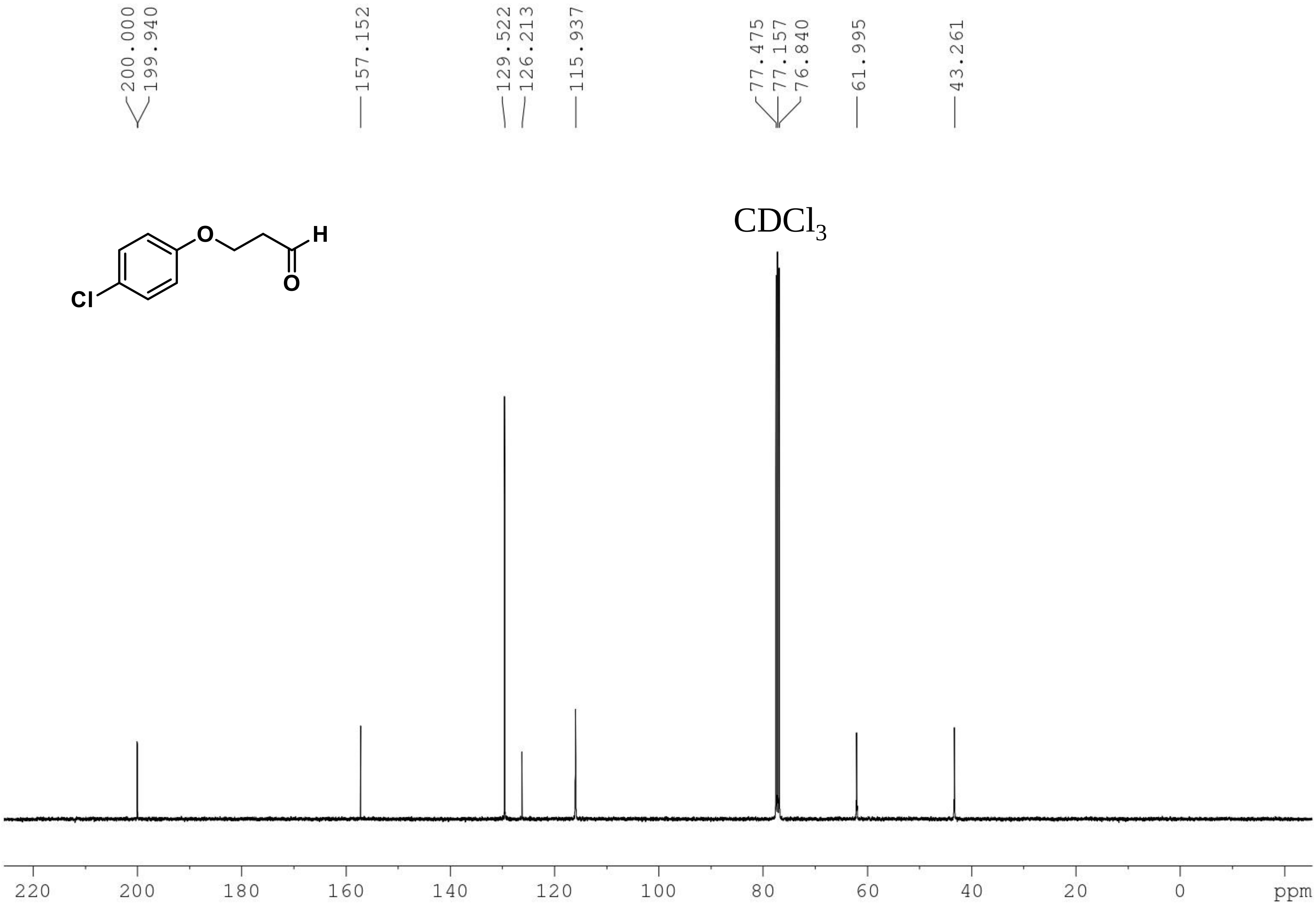

^{1}H, DMSO-d_6 (400 MHz)

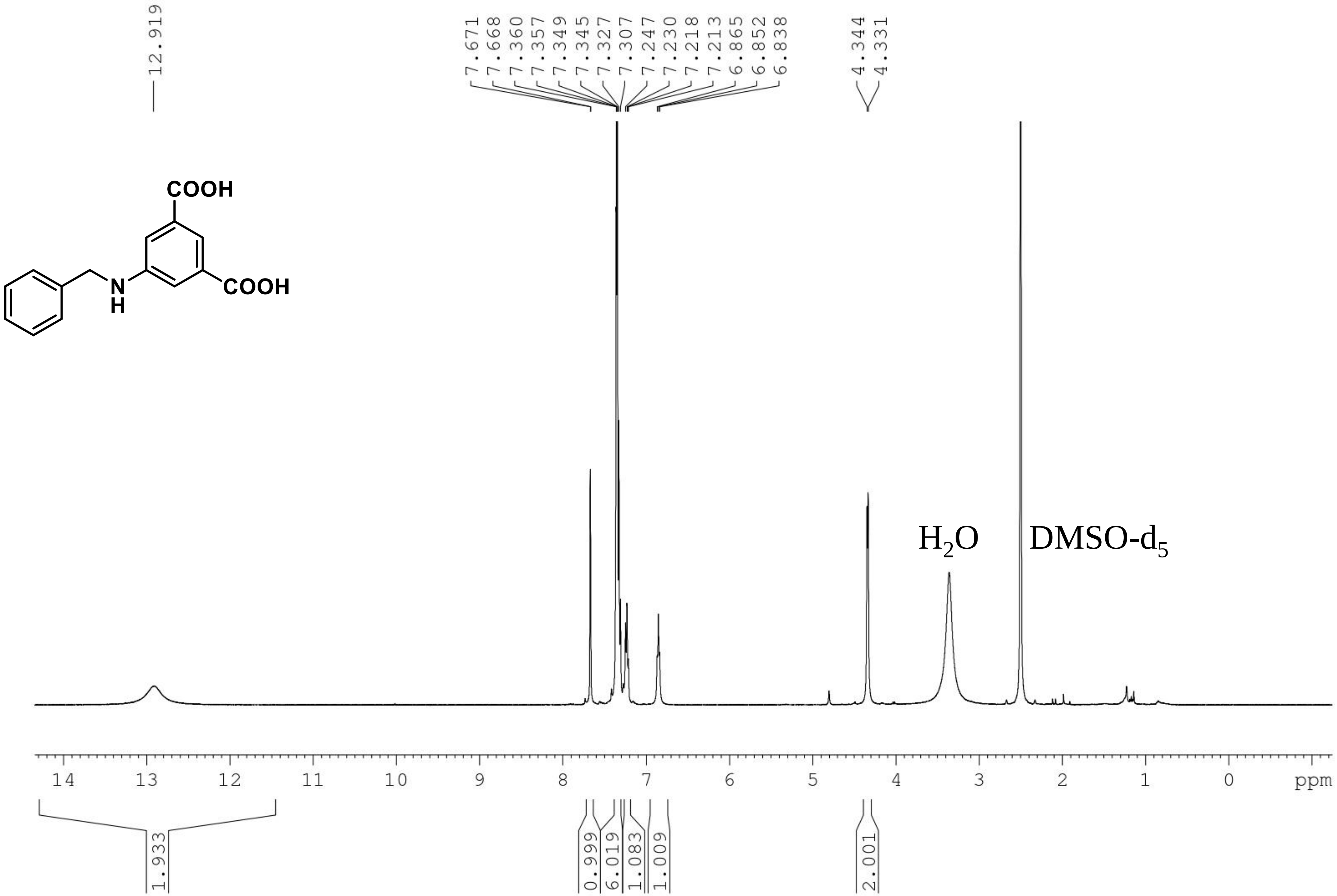

^{13}C, DMSO-d$_6$ (100 MHz)

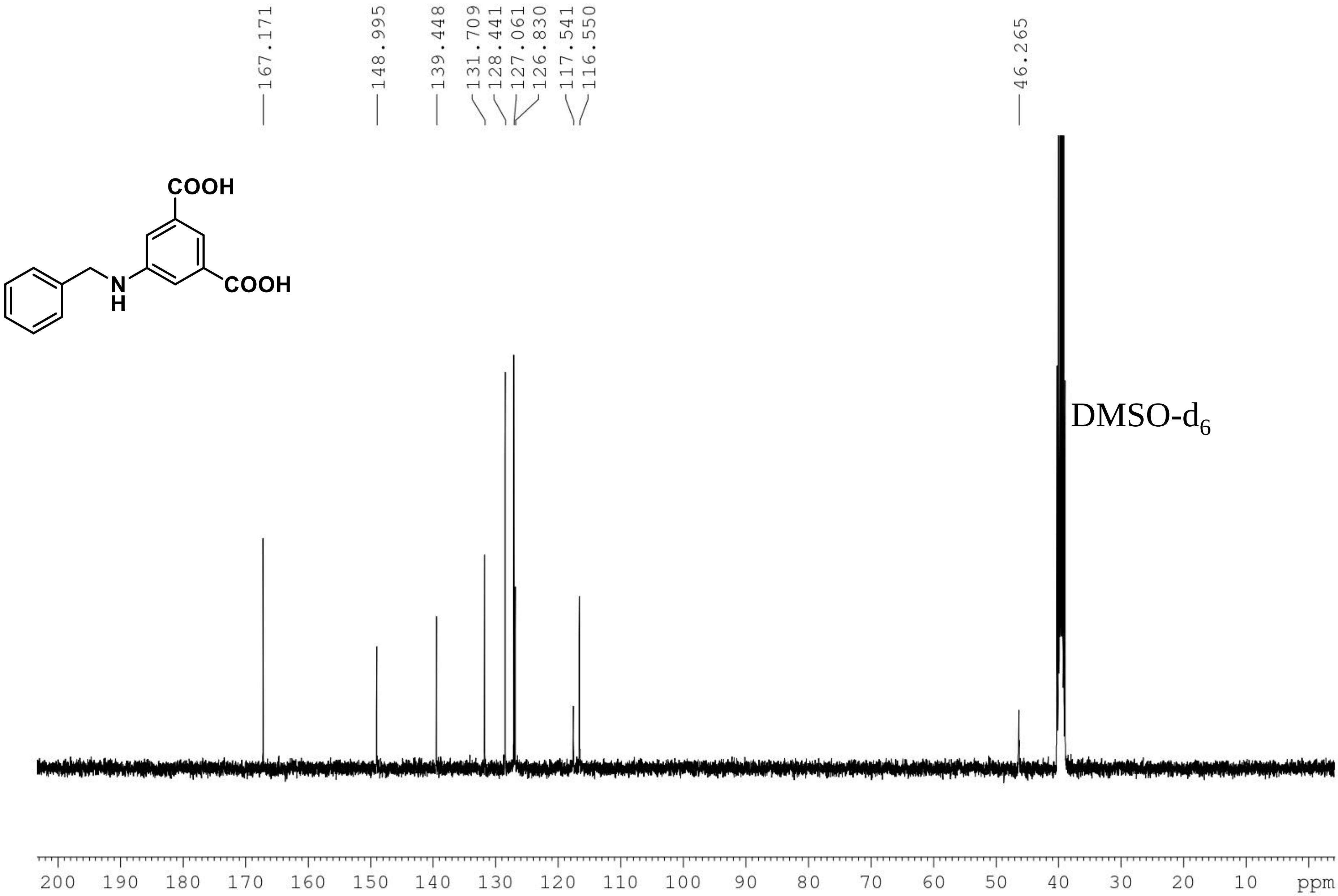

^1H, DMSO-d_6 (400 MHz)

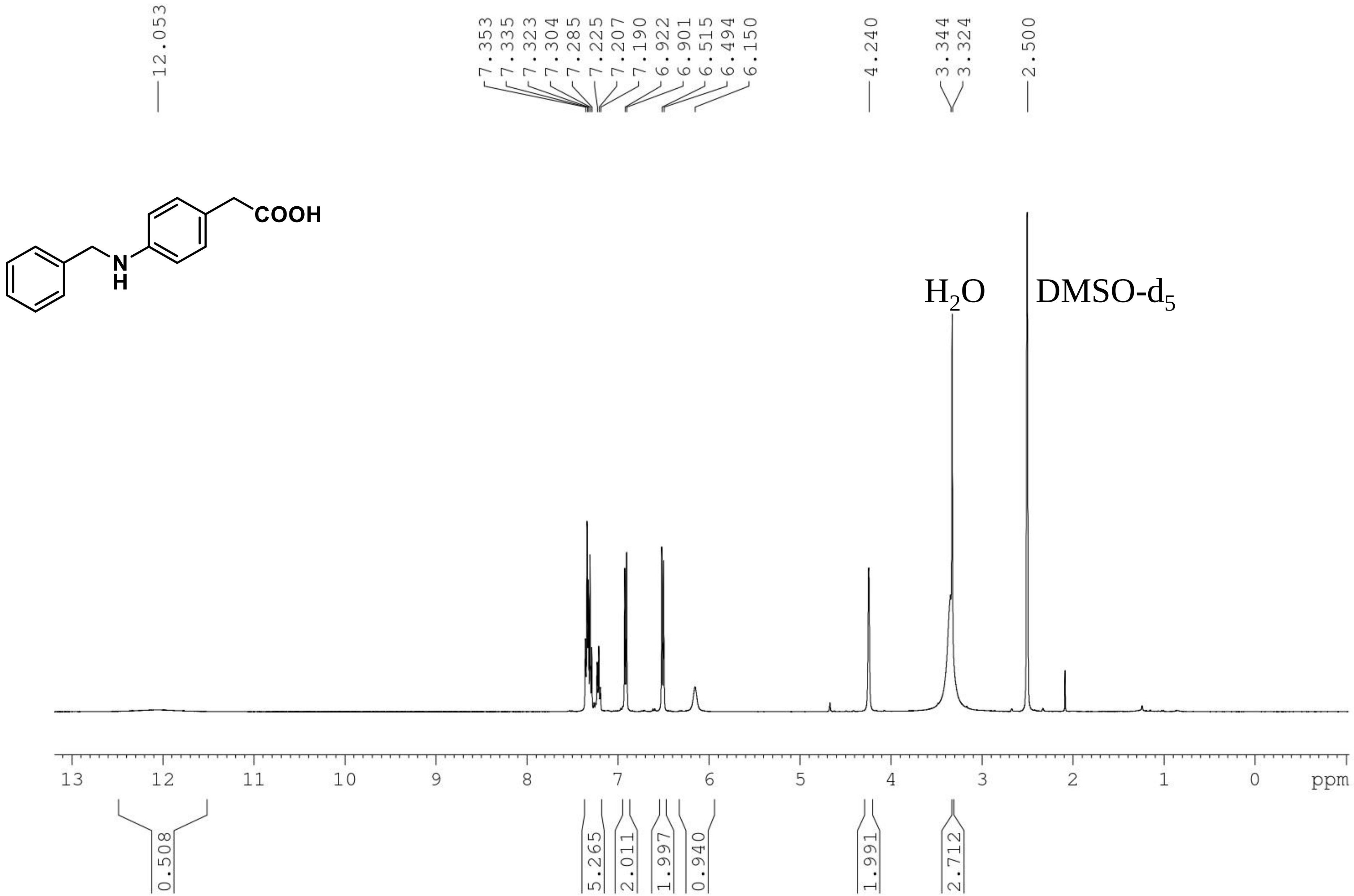

^{13}C, DMSO-d_6 (100 MHz)

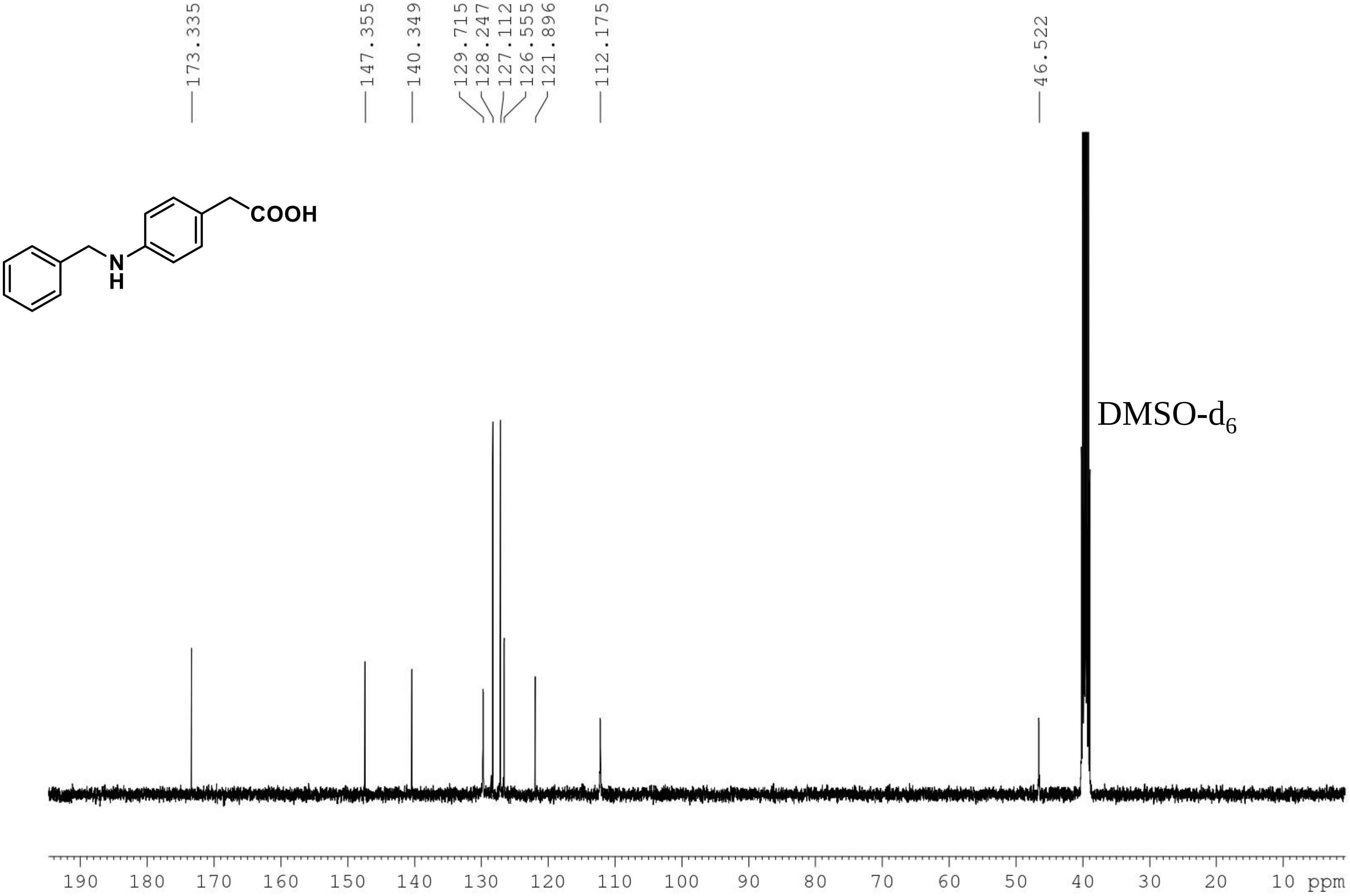

^{1}H, $CDCl_3$ (400 MHz)

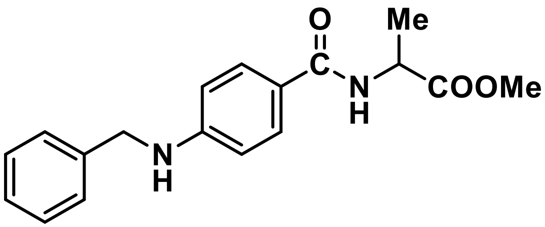

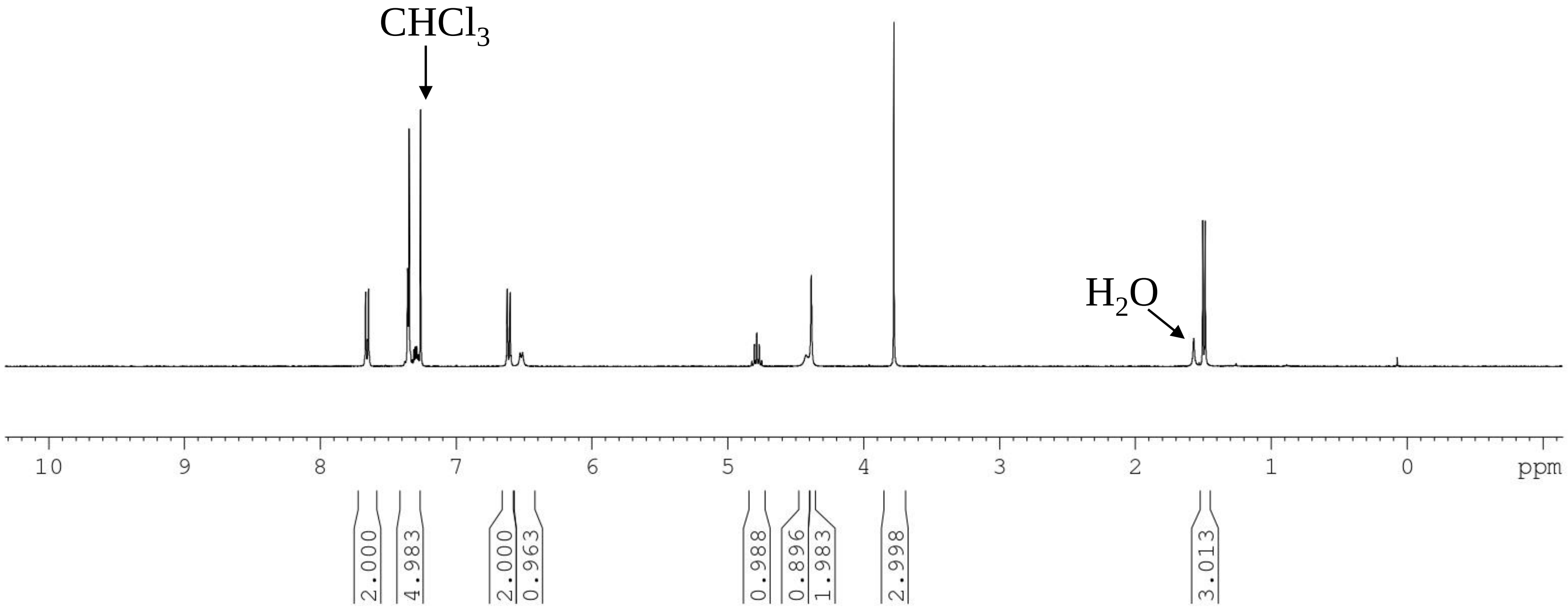

^{13}C, $CDCl_3$ (100 MHz)

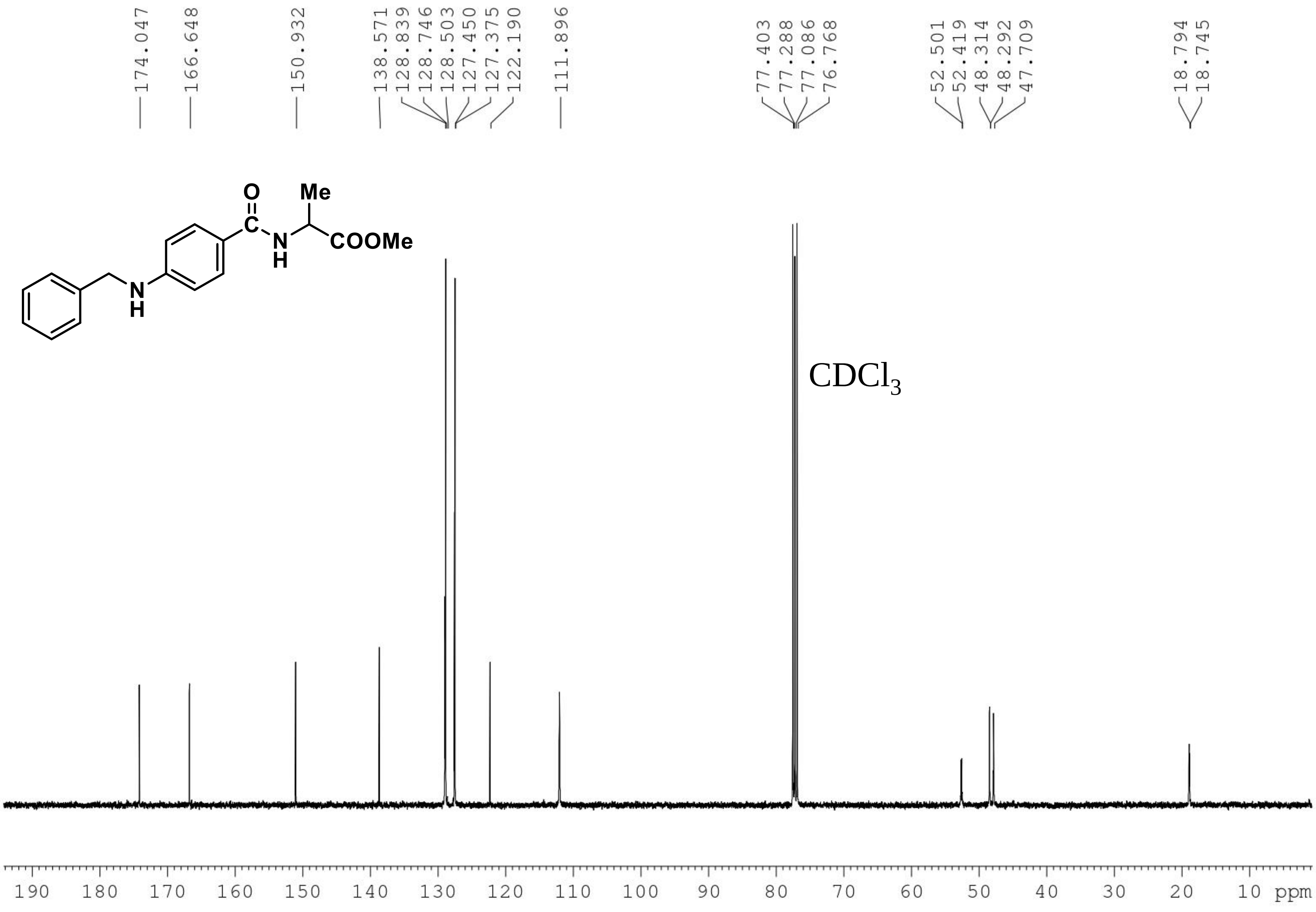

^{1}H, $CDCl_3$ (400 MHz)

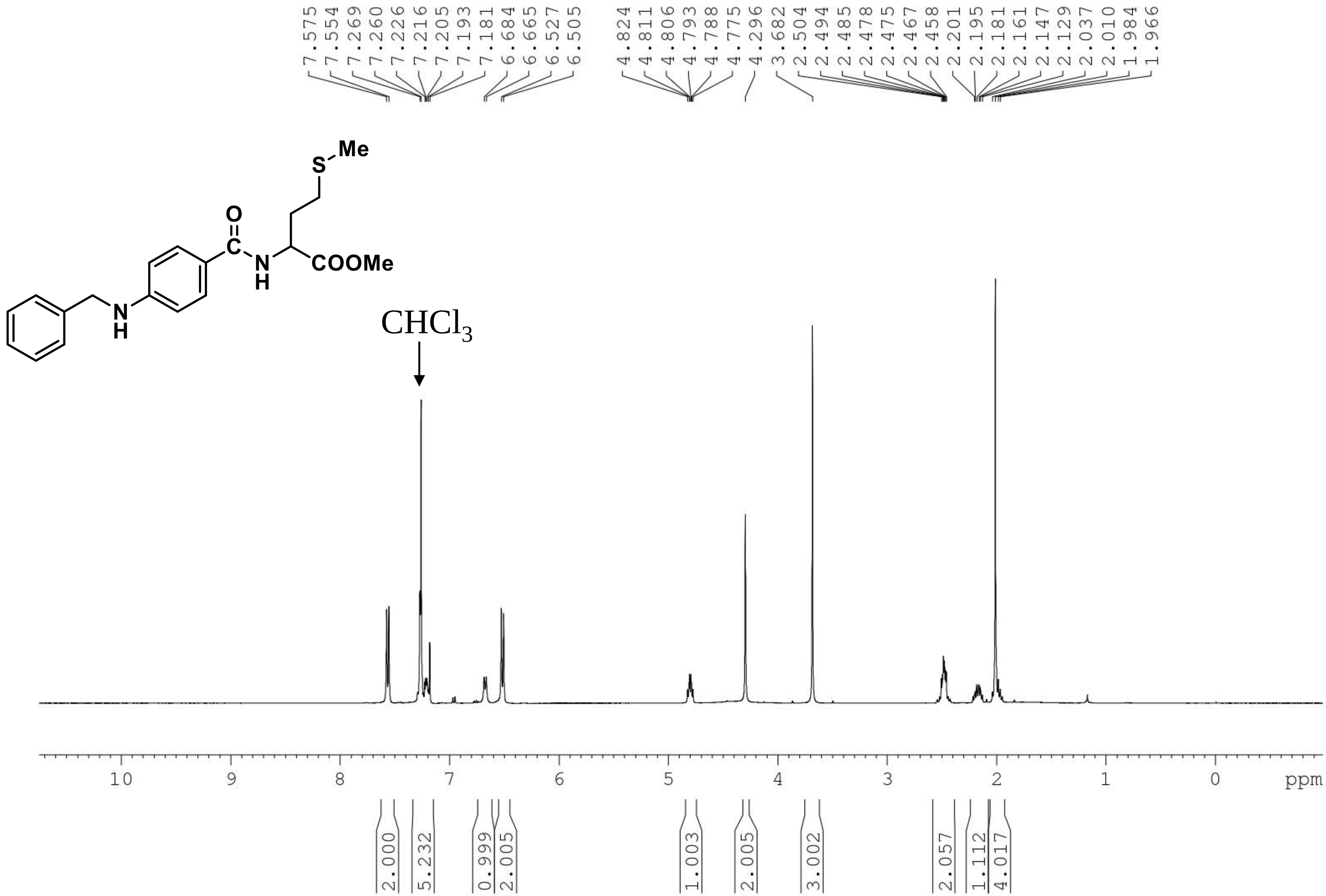

^{13}C, $CDCl_3$ (100 MHz)

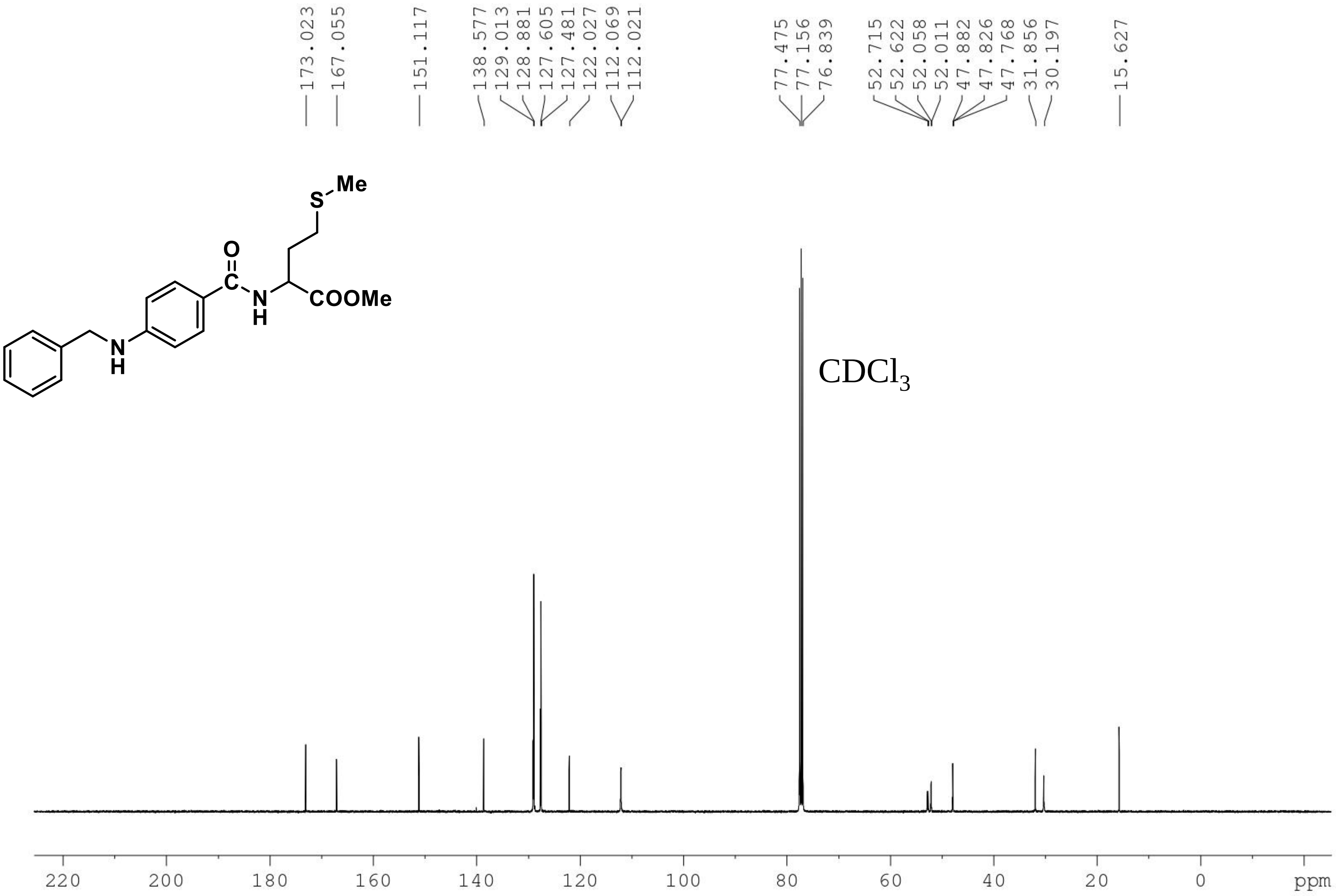

^1H, $CDCl_3$ (400 MHz)

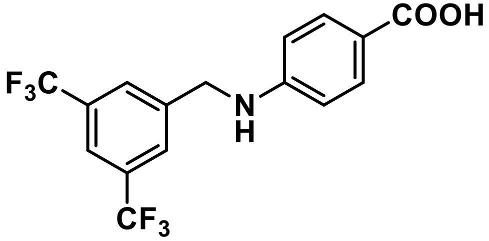

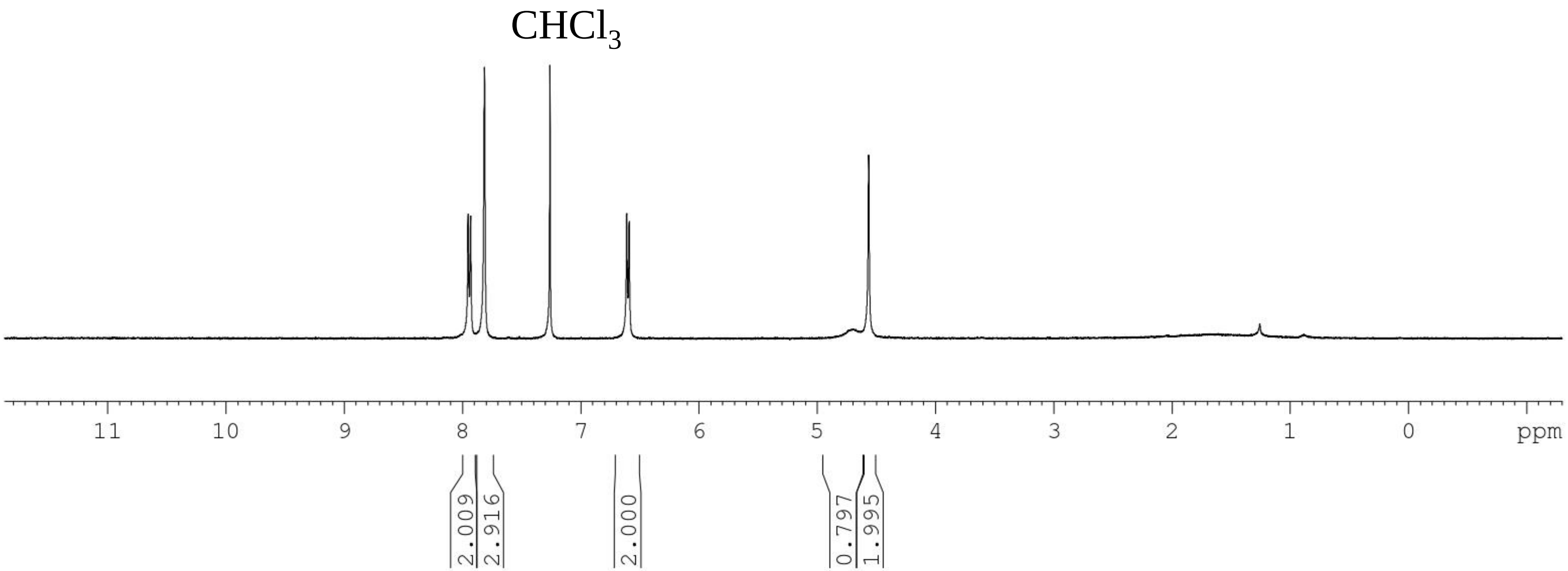

^{13}C, $CDCl_3$ (100 MHz)

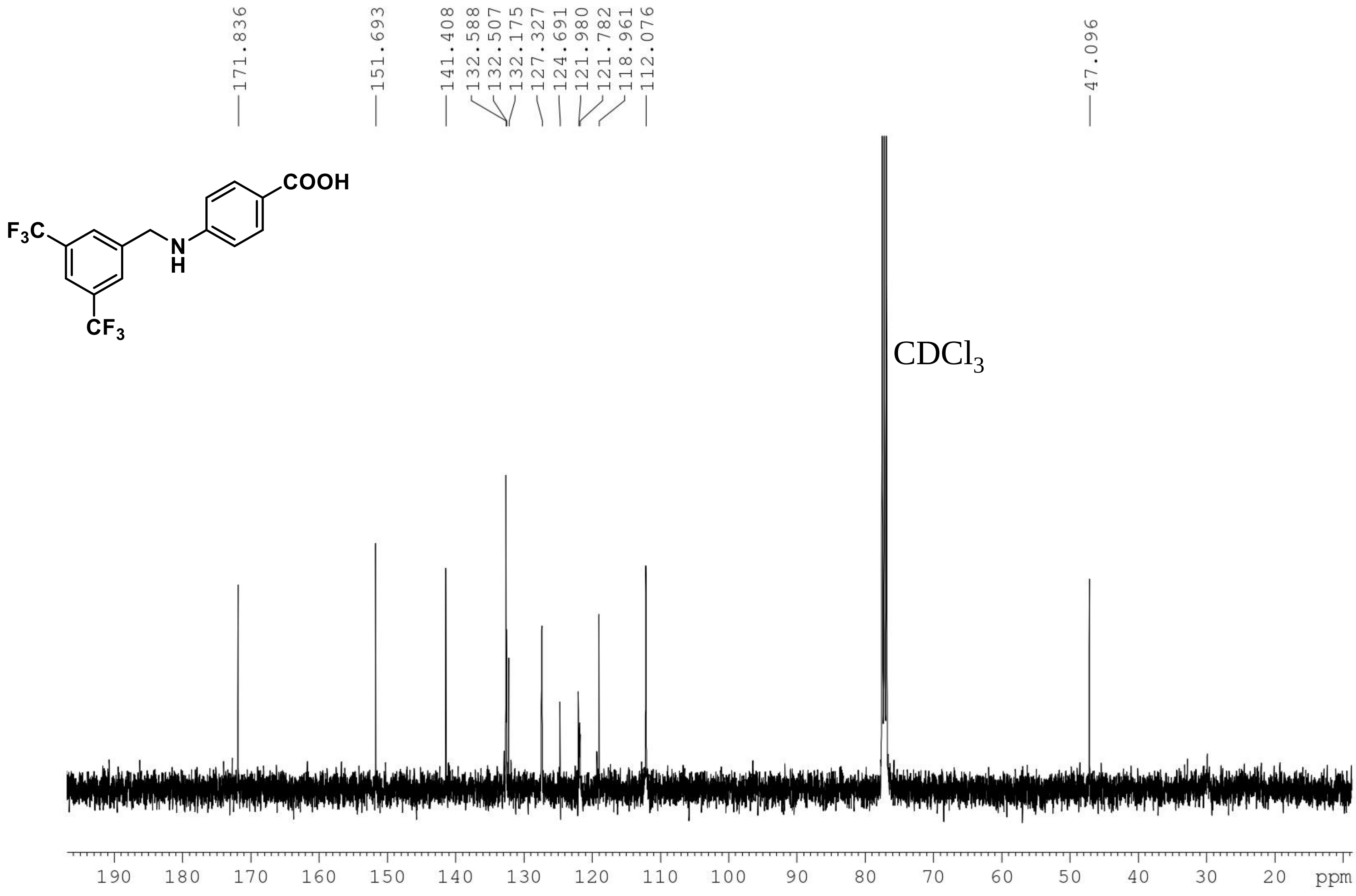

^{19}F, $CDCl_3$ (376 MHz)

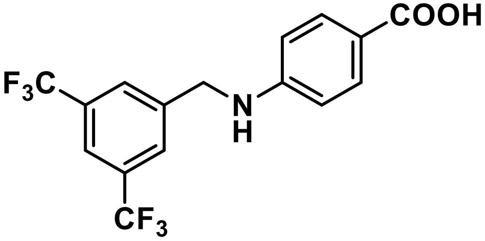

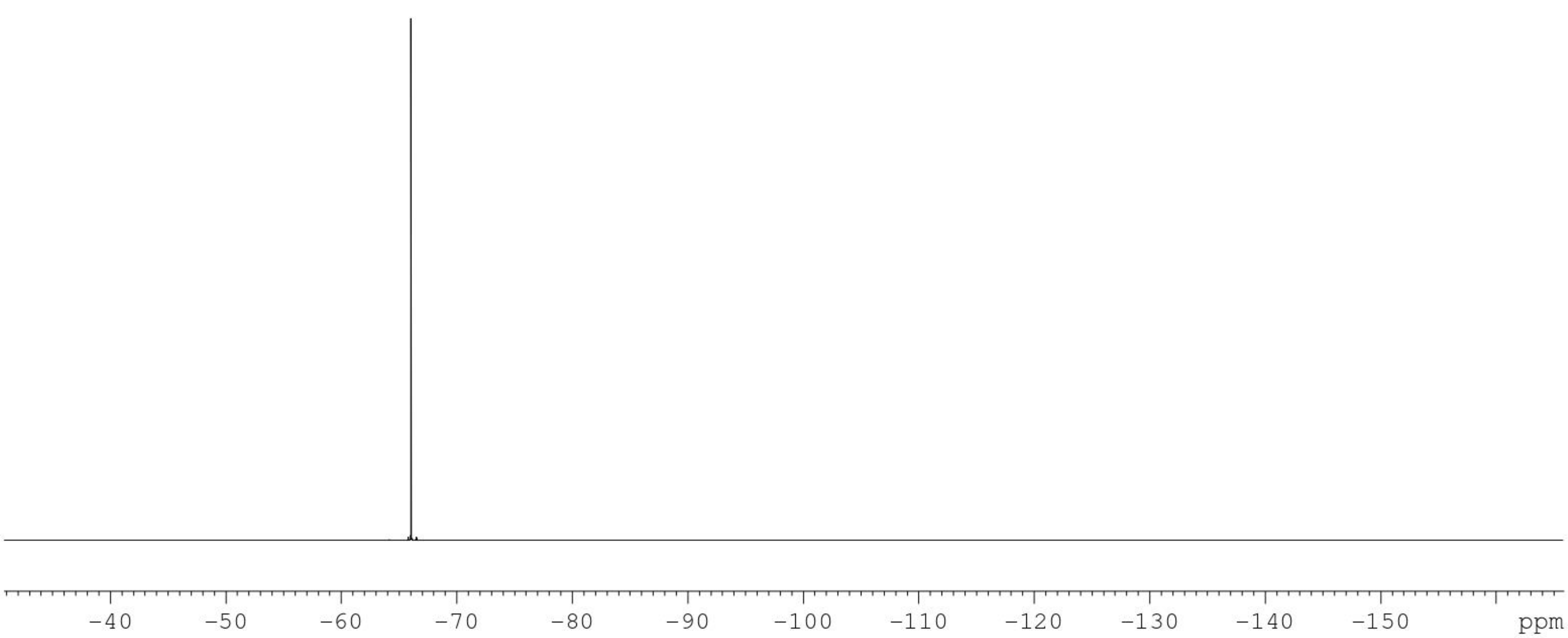

^{1}H, $CDCl_3$ (400 MHz)

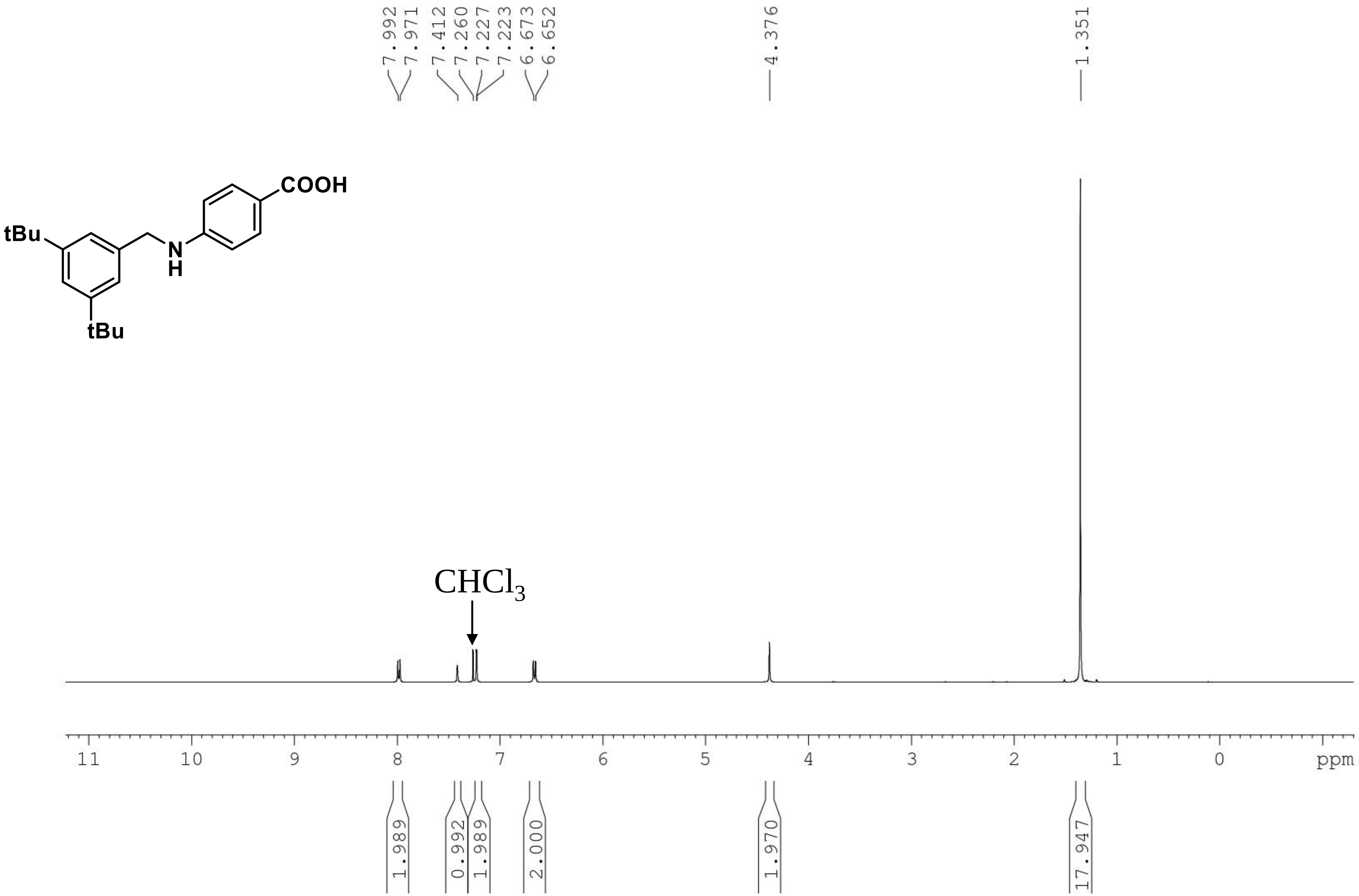

^{13}C, $CDCl_3$ (100 MHz)

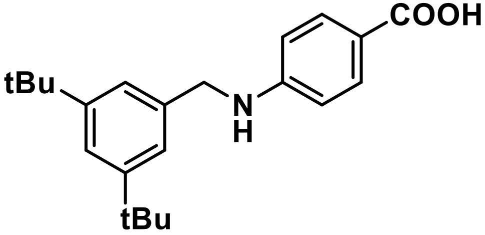

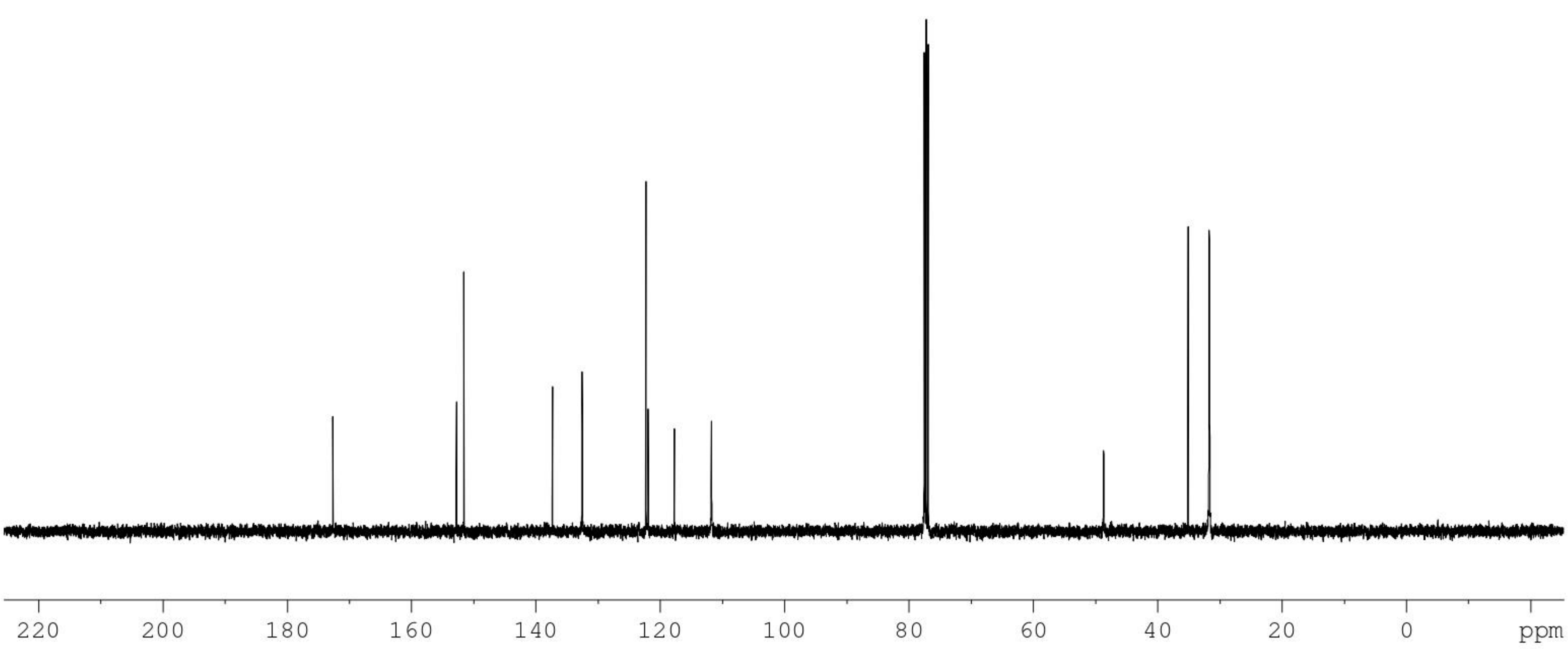

^{1}H, $CDCl_3$ (400 MHz)

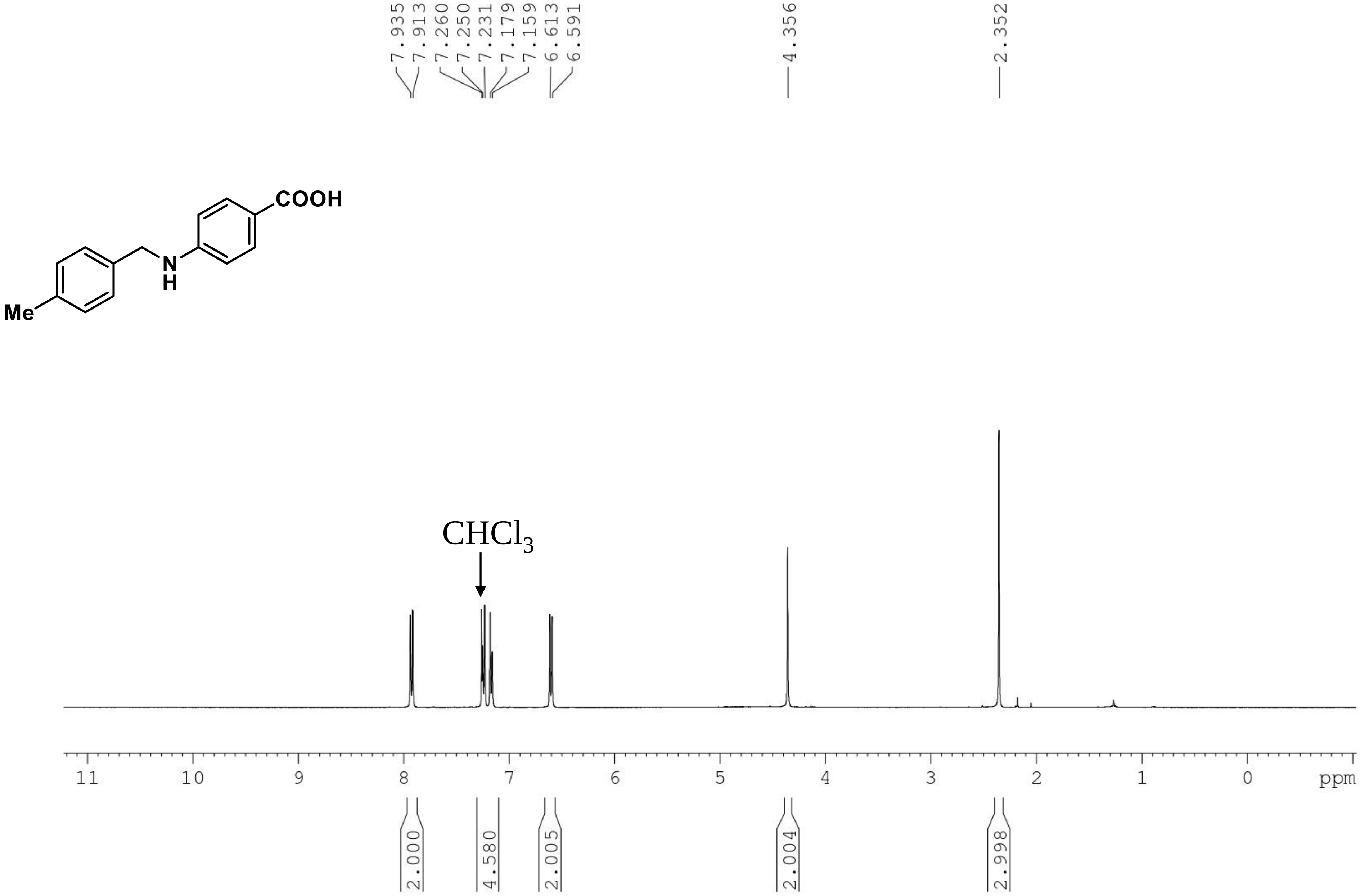

^{13}C, $CDCl_3$ (100 MHz)

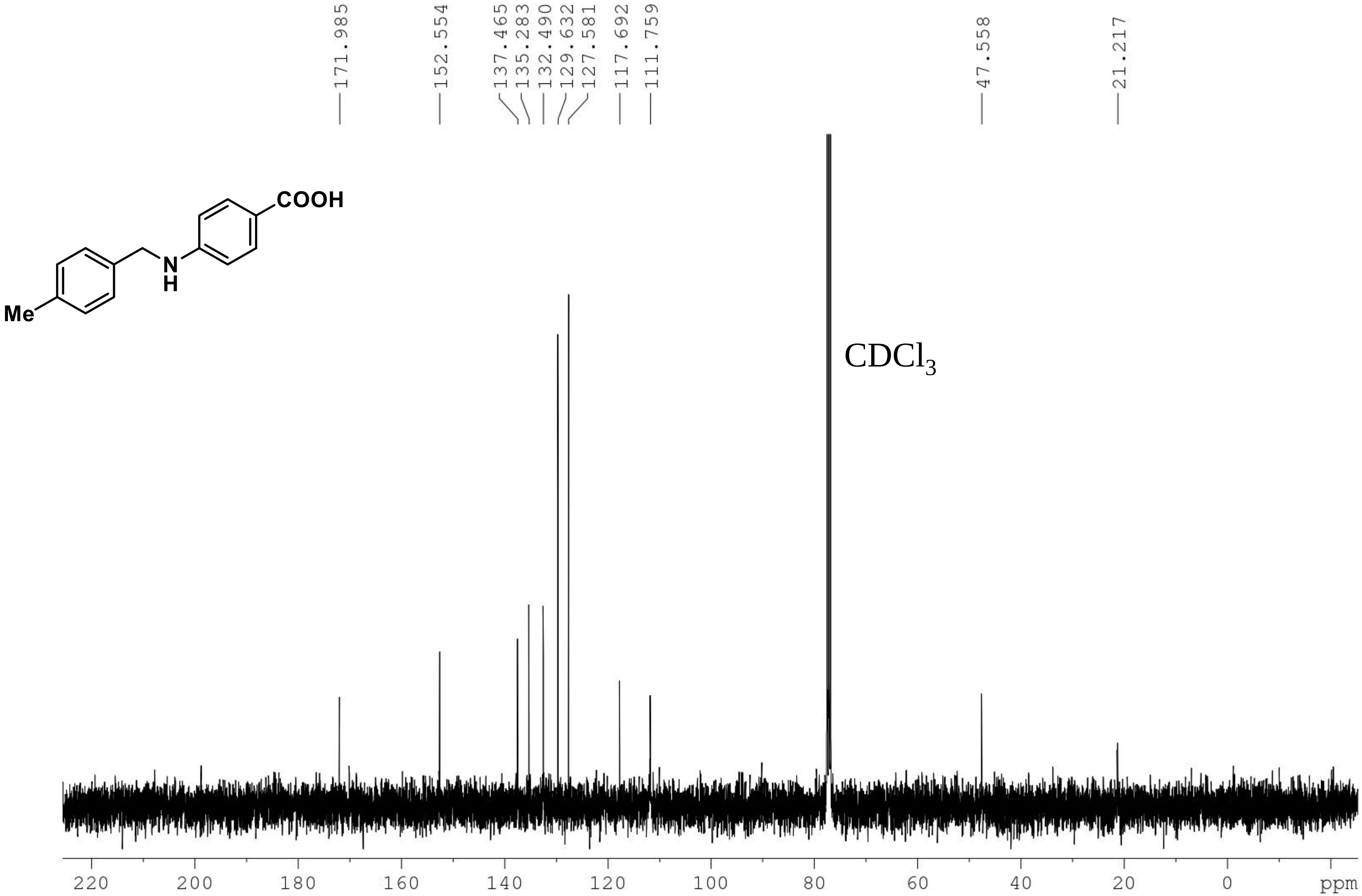

Supplementary References

S1. Saito, N. *et al.* Functional group evaluation kit for digitalization of information on the functional group compatibility and chemoselectivity of organic reactions. *Bull. Chem. Soc. Jpn.* **96**, 465–474 (2021).

S2. Prakash, M. *et al.* Identification of potent and selective inhibitors of fat mass obesity-associated protein using a fragment-merging approach. *J. Med. Chem.* **64**, 15810–15824 (2021).

S3. Godfrey, N. A., Schatz, D. J. & Pronin, S. V. Twelve-step asymmetric synthesis of (−)-nodulisporic acid C. *J. Am. Chem. Soc.* **140**, 12770–12774 (2018).

S4. Pallesen, J. *et al.* N1-substituted quinoxaline-2,3-diones as kainate receptor antagonists: X-ray crystallography, structure-affinity relationships, and in vitro pharmacology. *ACS Chem. Neurosci.* **10**, 1841–1853 (2019).

S5. Benke, B. P. & Madhavan, N. aminobenzoic acid incorporated octapeptides for cation transport. *Bioorganic Med. Chem.* **23**, 1413–1420 (2015).

S6. Zhao, M., Robert, D. & Jung, L. new agents for cutaneous photoprotection: derivatives of α-amino acids, 4-aminobenzoic and 4-methoxycinnamic acids. *Eur. J. Med. Chem.* **28**, 949–954 (1993).

S7. Beckett, M. A., Strickland, G. C., Holland, J. R., & Varma, K. S. A convenient n.m.r. method for the measurement of Lewis acidity at boron centers: Correlation of reaction rates of Lewis acid initiated epoxide polymerizations with Lewis acidity. *Polymer* **37**, 4629−4631 (1996).

S8. Pal, M., Parasuraman, K., & Yeleswarapu, K. R. Palladium-Catalyzed Cleavage of O/N-Propargyl Protecting Groups in Aqueous Media under a Copper-Free Condition[1]. *Org. Lett.* **5**, 349–352 (2003).

S9. Hoshimoto, Y., Kinoshita, T., Hazra, S., Ohashi, M. & Ogoshi, S. Main-group-catalyzed reductive alkylation of multiply substituted amines with aldehydes using H_2. *J. Am. Chem. Soc.* **140**, 7292–7300 (2018).

S10. Kwong, F. Y., Klapars, A. & Buchwald, S. L. Copper-catalyzed coupling of alkylamines and aryl iodides: an efficient system even in an air atmosphere. *Org. Lett.* **4**, 581–584 (2002).

S11. Frisch, M. J. et al. Gaussian 16 Revision C.01 (Gaussian, Inc., Wallingford, CT, 2019).

S12. Chai, J.-D. & Head-Gordon, M. Long-range corrected hybrid density functionals with damped atom–atom dispersion corrections. *Phys. Chem. Chem. Phys.* **10**, 6615–6620 (2008).

S13. Fukui, K. The path of chemical reactions - the IRC approach. *Acc. Chem. Res.* **14**, 363–368 (1981).

S14. Becke, A. D. & Johnson, E. R. A simple effective potential for exchange. *J. Chem. Phys.* **122**, 154104 (2005); Grimme, S., Antony, J., Ehrlich, S. & Krieg, H. A consistent and accurate ab initio parametrization of density functional dispersion correction (DFT-D) for the 94 elements H-Pu. *J. Chem. Phys.* **132**, 154104 (2010); Johnson, E. R. & Becke, A. D. A post-Hartree–Fock model of intermolecular interactions: Inclusion of higher-order corrections. *J. Chem. Phys.* **123**, 024101 (2005); Grimme, S., Ehrlich, S. & Goerigk, L. Effect of the damping function in dispersion corrected density functional theory. *J. Comput. Chem.* **32**, 1456–1465 (2011).

S15. Erdmann, P. & Greb, L. The role of hydrogen bonding in the catalytic hydrogenation of CO_2 to

methanol by a frustrated Lewis pair catalyst system: a computational study. *Angew. Chem., Int Ed* Engl., https://doi.org/10.1002/anie.202114550 (2022).

S16. Reineke, M.H., Sampson, M.D., Rheingold, A.L., Kubiak, C.P., Synthesis and Structural Studies of Nickel(0) Tetracarbene Complexes with the Introduction of a New Four-Coordinate Geometric Index, τ_{δ}. *Inorg. Chem*. 54, 3211–3218 (2015).

S17. Höpfl, H., Synthesis and structure of a novel organometallic polymer containing a Re-Re bond: $[Re_2(CO)_6(\mu\text{-}\eta1\text{: }\eta5\text{-}C_5H_4CH_2)]_n$. *J. Organomet. Chem.* **581**, 129−133 (1999); Toyota, S., Ōki, M., Synthesis of intramolecular boron-amine complexesand proposal of tetrahedral character for correlation between molecular structure and barrier to dissociation of N-B bonds.*Bull Chem Soc Jpn.***65**, 1832−1837 (1992).

S18. Keith, T. A. TK Gristmill Software, Overland Park KS, USA, 2019 (aim.tkgristmill.com).
